# Azapeptide-Based SARS-CoV-2 Main Protease Inhibitors: Design, Synthesis, Enzyme Inhibition, Structural Determination, and Antiviral Activity

**DOI:** 10.1021/acs.jmedchem.5c01520

**Published:** 2025-09-11

**Authors:** Philipp Flury, Jyoti Vishwakarma, Katharina Sylvester, Nobuyo Higashi-Kuwata, Agnieszka K. Dabrowska, Renee Delgado, Ashley Cuell, Rahul Basu, Alexander B. Taylor, Ellen Gonçalves de Oliveira, Mateus Sá Magalhães Serafim, Jingxin Qiao, Yan Chen, Shengyong Yang, Anthony J. O’Donoghue, Hiroaki Mitsuya, Michael Gütschow, Stefan A. Laufer, Christa E. Müller, Reuben S. Harris, Thanigaimalai Pillaiyar

**Affiliations:** † Institute of Pharmaceutical Sciences, Department of Pharmaceutical and Medicinal Chemistry, 9188 Eberhard Karls University Tübingen, Auf der Morgenstelle 8, Tübingen 72076, Germany; ‡ Tübingen Center for Academic Drug Discovery & Development (TüCAD_2_), Eberhard Karls University Tübingen, Auf der Morgenstelle 8, Tübingen 72076, Germany; § Department of Biochemistry and Structural Biology, 14742 University of Texas Health Science Center at San Antonio, San Antonio, Texas 78229, United States; ∥ PharmaCenter Bonn, Pharmaceutical Institute, Pharmaceutical & Medicinal Chemistry, University of Bonn, An der Immenburg 4, Bonn 53121, Germany; ⊥ Department of Refractory Viral Diseases, National Institute of Global Health and Medicine, 9374 Japan Institute for Health Security, 1-21-1 Toyama, Shinjuku-ku, Tokyo 162-8655, Japan; # Greehey Children’s Cancer Research Institute, 350198 University of Texas Health Science Center at San Antonio, San Antonio, Texas 78229, United States; ∇ Department of Microbiology, Institute of Biological Sciences, Federal University of Minas Gerais, Belo Horizonte, Minas Gerais 31270-901, Brazil; ○ Center for Discovery and Innovation in Parasitic Diseases, Skaggs School of Pharmacy and Pharmaceutical Sciences, University of California, 9500 Gilman Drive, San Diego, La Jolla, California 92093-0657, United States; ◆ Department of Biotherapy, Cancer Center and State Key Laboratory of Biotherapy, West China Hospital, Sichuan University, Chengdu, Sichuan 610041, China; ¶ Howard Hughes Medical Institute, 34753 University of Texas Health San Antonio, San Antonio, Texas, 78229, United States

## Abstract

M^pro^ of SARS-CoV-2 plays a vital role in the replication and pathogenesis of virus. Additionally, its high conservation within the *Coronaviridae* family makes it an attractive therapeutic target for developing broad-spectrum agents. This study describes the design, synthesis, and structure−activity relationships of azapeptide-based SARS-CoV-2 M^pro^ inhibitors, leading to several compounds with nanomolar IC_50_ values. Examples include **14r** (IC_50_ = 13.3 nM), **14s** (IC_50_ = 30.6 nM), **20a** (**TPG-20a**, IC_50_ = 28.0 nM), and **20g** (IC_50_ = 30.4 nM). Some compounds inhibit MERS-CoV and SARS-CoV-1 M^pro^ but not the human protease cathepsin L. Several inhibitors, such as **20a** and **20f**, exhibit antiviral activity with potencies comparable to nirmatrelvir and activity against the E166V-carrying SARS-CoV-2 variant (SARS-CoV-2^E166V^). An M^pro^ cocrystal structure with **20a** shows a covalent adduct with the catalytic Cys145. Overall, these new inhibitors are promising chemical tools that may contribute to the identification of future pan-anticoronaviral drugs.

## Graphical abstract

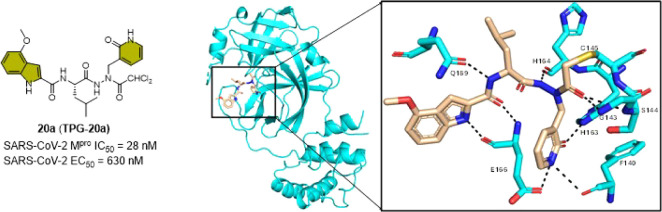

## Introduction

The severe acute respiratory syndrome coronavirus-2 (SARS-CoV-2) is an enveloped, single-stranded RNA virus belonging to the β-coronavirus genus of the *Coronaviridae* family. It is the causative agent of the recent coronavirus disease-19 (COVID-19) pandemic.^1–7^ The SARS-CoV-2 genome encodes polyproteins pp1a and pp1ab, which are a source of nonstructural proteins (nsp1−16) essential to the proper assembly and maturation of the virus. The main protease (M^pro^, also known as nsp5 or 3CL^pro^) is necessary to digest nsps at over 11 distinct sites during viral replication. The homodimeric cysteine protease M^pro^ specifically cleaves polypeptides with glutamine in the P1 position. This primary substrate specificity is conserved across other coronaviruses, including severe acute respiratory syndrome coronavirus-1 (SARS-CoV-1) and Middle East respiratory syndrome coronavirus (MERS-CoV).^8–10^ Given its highly conserved structure across diverse species of coronaviruses, its central role in viral replication, and its distinctive substrate specificity, which is not shared by any of the human cysteine proteases, M^pro^ has been a primary target for developing antiviral drugs to treat COVID-19.^10–14^

Clinical inhibitors of M^pro^ are nirmatrelvir (NMV) and ensitrelvir (ESV). The former needs to be combined with ritonavir, an inhibitor of human cytochrome P450 3A4 (CYP3A4), to increase its plasma half-life. Moreover, the development of drug resistance can undermine their clinical effectiveness. Resistant SARS-CoV-2 strains have emerged against both NMV and ESV.^15–20^ Additionally, NMV and ESV have limited potential for use against other coronavirus species, such as HCoV-NL-63 and HCoV-229E, whose M^pro^ enzymes are less efficiently inhibited.^21,22^ Therefore, it is essential to continue developing M^pro^ inhibitors with unique chemotypes to effectively prevent future coronavirus outbreaks and treat novel emerging coronavirus infections.

Recently, azapeptides have been proposed as next-generation anticoronaviral drugs (Figure 1a) for inhibiting the M^pro^ enzyme of SARS-CoV-2. It has been shown that azapeptide nitriles, such as compound **8**, exhibit good SARS-CoV-2 M^pro^ inhibition and moderate antiviral efficacy. In addition, the reported compounds also showed an inhibitory effect on cathepsin L.^23,24^ The macrocyclization of azapeptide nitriles substantially improved the inhibitory properties.^25^ In a biochemical investigation, Vanhoutte et al. described azapeptides as activity-based probes that enable the detection of active M^pro^ within infected cells. Compound **7d** was highly effective against SARS-CoV-2 M^pro^; however, its cell activity was reduced, most likely due to limited cell permeability.^26^ The chemotype of azapeptide nitriles was also employed to develop an active-site titrant for M^pro^.^27^ Azapeptide inhibitors with a chlorofluoroacetamide (CFA) moiety as a cysteine-reactive warhead strongly inhibited M^pro,^ showing covalent binding. Their antiviral activity varied between different cell lines.^28^ For example, the highly efficient M^pro^ inhibitor **YH-6** had an EC_50_ of 21.2 nM in 293TAT cells, while a 300-fold increased value was observed in VeroE6/transmembrane serine protease 2 (TMPRSS2) cells.

**Figure 1:**
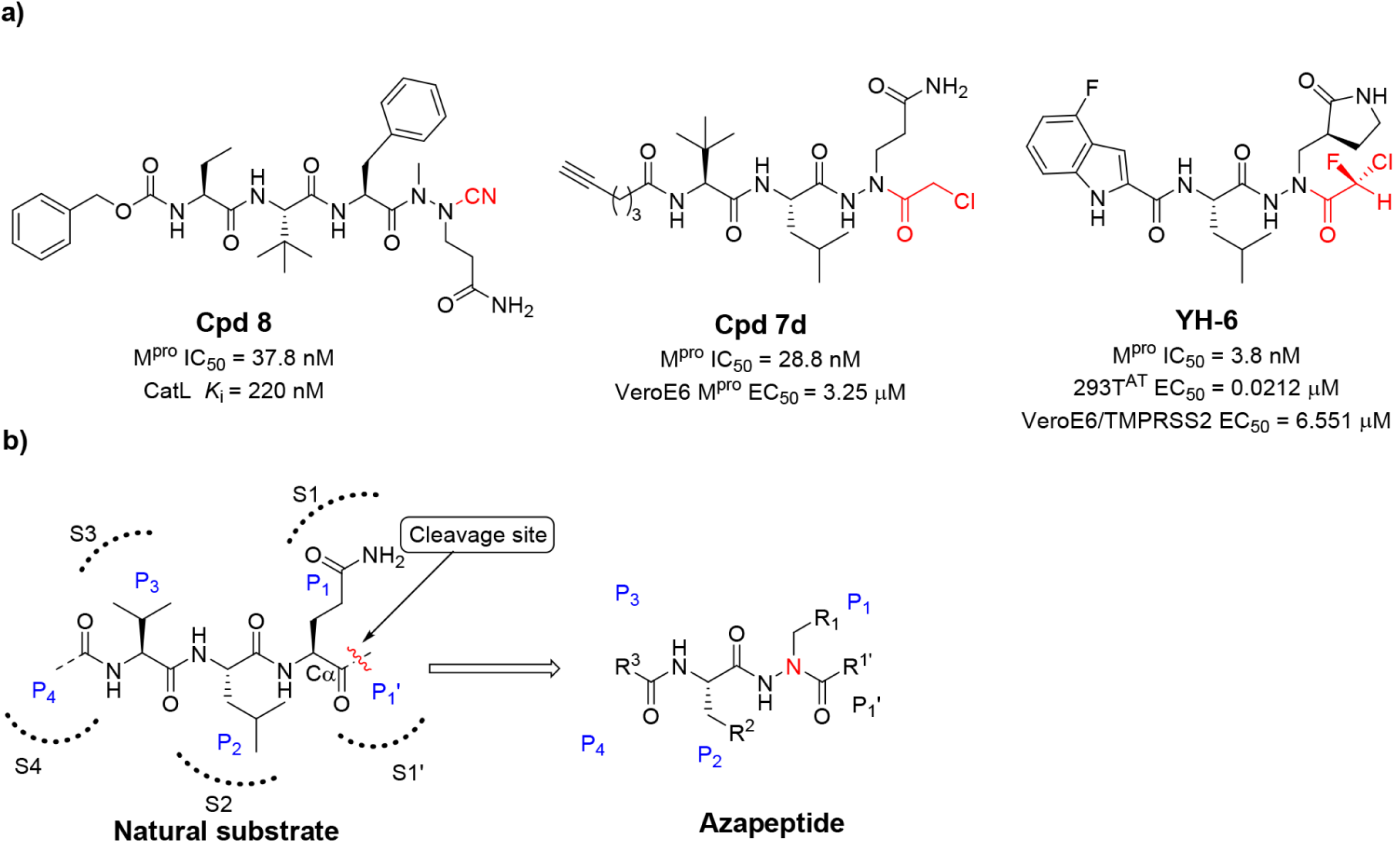
Azapeptides that interact with SARS-CoV-2 M^pro^. Structure and activity of representative azapeptide SARS-CoV-2 M^pro^ inhibitors (a). The warhead group is highlighted in red. (b) Design of azapeptide by replacing the α-carbon of the P1 amino acid with nitrogen in the M^pro^ peptide substrate (b). For R^1^-R^3^ and R^1^’, see Tables 1 and 2.

In the present work, we expanded the chemical space of azapeptide-based inhibitors by reducing the peptide character of the molecules. Based on extensive structure−activity relationship studies, we enhanced M^pro^ inhibitory activity, resulting in compounds that exhibit potent antiviral activity in virus-infected cells without exhibiting cell toxicity. Multiple inhibitors demonstrate antiviral effectiveness with strengths comparable to those of NMV and show high potency against the E166V-carrying SARS-CoV-2 variant (SARS-CoV-2 E166V). The cocrystallization of a selected compound in complex with M^pro^ yielded valuable insights into target−inhibitor interactions. The most promising compounds were investigated for their selectivity toward human cysteine proteases, such as CatL, and their suitability as broad-spectrum agents against further pathogenic coronaviruses.

## Results and Discussion

### Design of New Azapeptide M^pro^ Inhibitors

Azapeptides are a class of molecules containing a nitrogen atom named aza-nitrogen in the original Cα position in the peptide backbone (Figure 1b).^29^ The conversion of carbon into a more nucleophilic nitrogen atom offers several advantages to azapetides compared to peptidomimetics, which are available for M^pro^ inhibition. First, the introduction of aza-nitrogen alters the compounds’ properties, enabling, for example, the straightforward introduction of a wide variety of covalent warheads. Additionally, the aza-nitrogen atom in the peptide backbone enhances potency and selectivity by increasing the bond stability.^30–35^ Azapeptides have been investigated as inhibitors of HIV protease and human cathepsins, yielding encouraging results in biological and preclinical studies.^36,37^ The current study focused on the following modifications: (i) Several cysteine-reactive electrophiles were used to cap the aza nitrogen, allowing for covalent alteration of the M^pro^ active site. (ii) The S1 region plays a significant role in the potency and selectivity of potential inhibitors. Thus, various (hetero)­aromatic groups were introduced to replace the P1 glutamine side chain, thereby achieving selectivity over other proteases. (iii) The P2 residue was investigated, probing a range of substituents and addressing the hydrophobicity and size of the S2 pocket. (iv) Lastly, the solvent-exposed S3−S4 region was examined using several cyclic heteroaromatic scaffolds.

### Synthesis of Compounds

Schemes 1–3 depict the synthesis of 53 final compounds (**5a-h**, **6a-e**, **7a**, **8a-k**, **14a-s**, and **20a-i**), all of which are new. In Scheme 1, the synthesis of compounds **5a-h**, **6a-e**, **7a**, and **8a**-**k** shows the target compounds displaying unique P1 and reactive warhead (P1’) moieties. *O*-(7-Azabenzotriazol-1-yl)-*N*,*N*,*N*′,*N*′-tetramethyluronium hexafluorophosphate (HATU)-mediated coupling of commercially available 2-(2,4-dichlorophenoxy)­acetic acid with *l* -leucine methyl ester hydrochloride in the presence of *N*,*N*-diisopropylethylamine (DIPEA) resulted in the intermediate **1a**. Hydrazine hydrate, dissolved in water at a concentration of 80%, was employed to achieve the hydrazinolysis of the methyl ester. Following this, hydrazide **2a** reacted with a broad range of (hetero)­aromatic carbaldehydes in THF in the presence of a catalytic quantity of glacial acetic acid to generate the hydrazones **3a-l**. The hydrazones were regioselectively reduced to alkylhydrazides (**4a**-**l**) using a dimethylaminoborane complex in the presence of *p*-toluenesulfonic acid in a methylene chloride/methanol (4:1) mixture.^38^ These key intermediates were then linked to several warhead functionalities. Reaction of **4a** with different carboxylic acid chlorides or anhydrides in the presence of sodium hydrogencarbonate in acetone produced compounds **5a-h**. Compounds **6a-e** were obtained by the coupling reaction of **4a** with the readily accessible carboxylic acids or sodium salts, mediated by 2,4,6-tripropyl-1,3,5,2,4,6-trioxatriphosphinane 2,4,6-trioxide (T3P) and hydroxybenzotriazole (HOBt) monohydrate in the presence of DIPEA.^39^ Compound **7a**, which contains a difluoromethylacetyl warhead group, was produced by reacting **4a** with difluoroacetic anhydride in the presence of triethylamine (TEA). Compounds **8a**-**k** were synthesized by coupling **4a**-**l** with chloroacetic acid or sodium chloroacetate, mediated by T3P and HOBt in the presence of DIPEA.

**Scheme 1:**
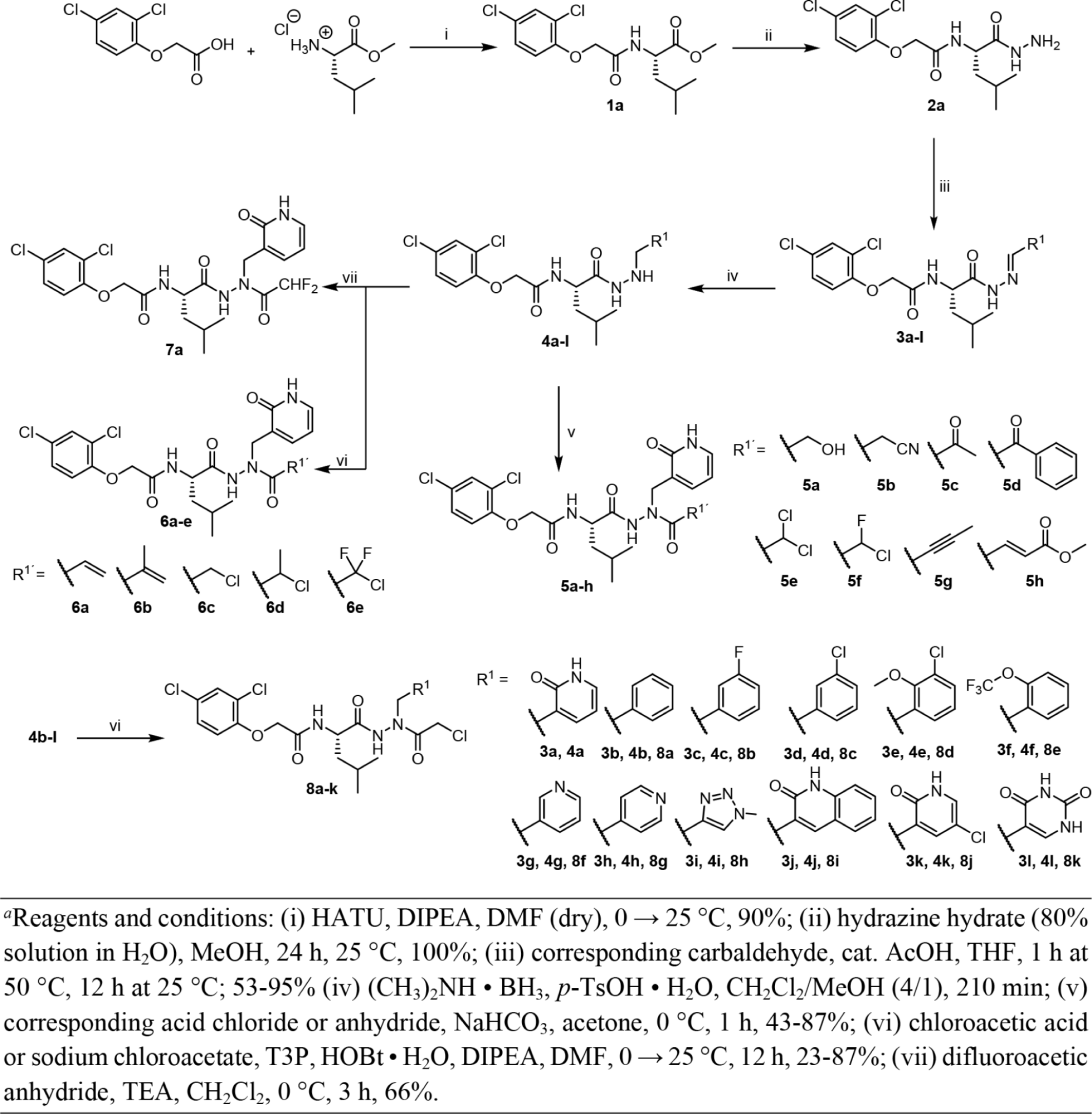
Synthesis of Compounds **5a-h**, **6a-e**, **7a**, and **8a**-**k**

Scheme 2 outlines the synthesis of compounds **14a-s** featuring diverse P2 moieties. A range of commercially available amino acid esters (**9a-s**) were coupled with 2-(2,4-dichlorophenoxy)­acetic acid in the presence of DIPEA to obtain intermediates **10a**-**s**. This reaction was mediated by either T3P or HATU. The hydrazinolysis of esters with hydrazine hydrate resulted in hydrazides (**11a**-**s**), which were subsequently reacted with 2-oxo-1,2-dihydropyridine-3-carbaldehyde or 5-chloro-2-oxo-1,2-dihydropyridine-3-carbaldehyde to generate the hydrazones **12a**-**s**. Further, these hydrazones were reduced and treated with chloroacetyl chloride to afford the products **14a**-**s**. As fixed structural elements, the final compounds mainly contained the pyridin-2­(1*H*)-one group in the P1 side chain and the dichlorophenyl ether as N-terminal capping group.

**Scheme 2:**
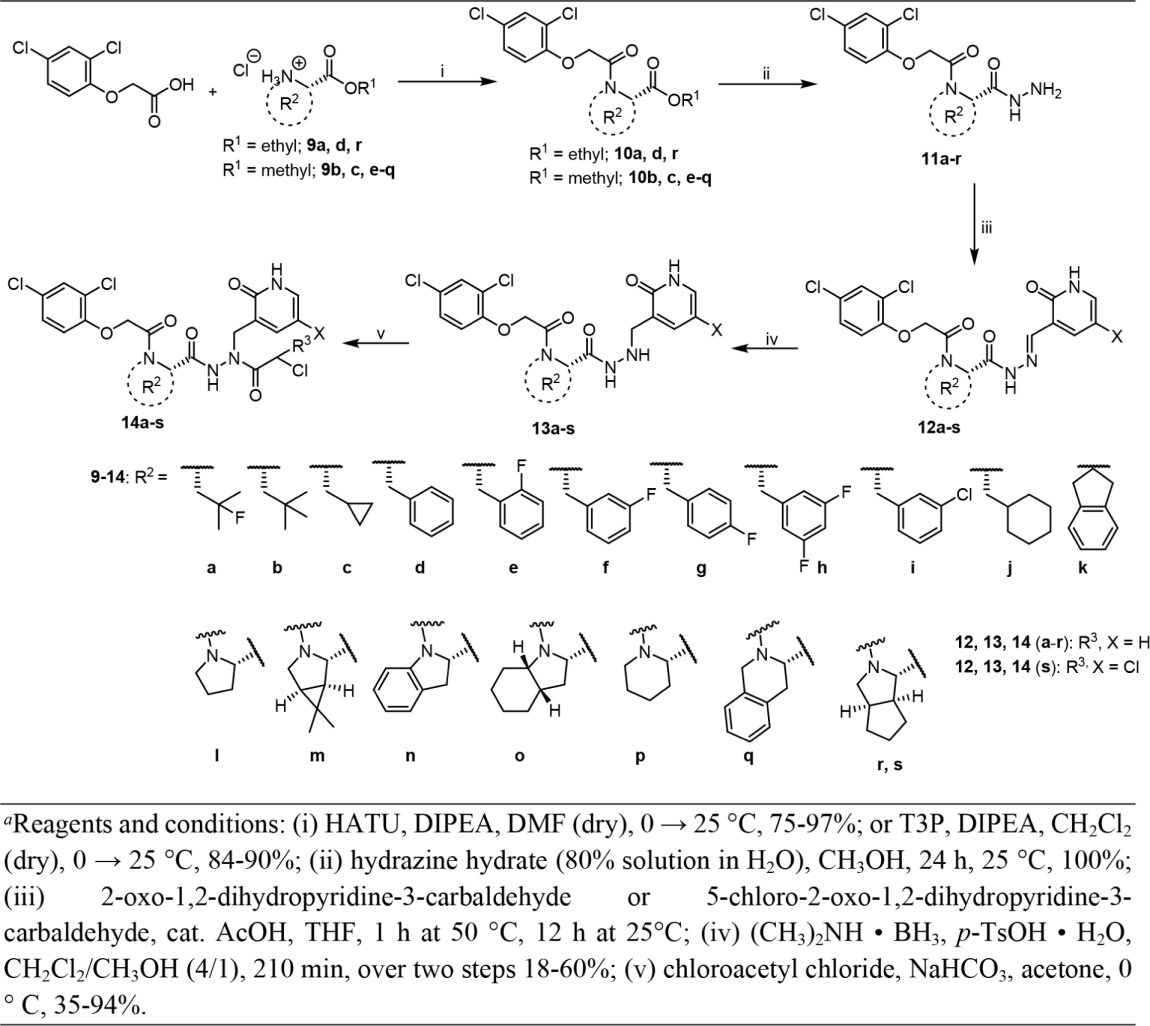
Synthesis of Compounds **14a-s**

Next, as shown in Scheme 3, two further diversity points were introduced in the final compounds **20a**-**i**. We modified the lactam moiety by placing substituents at the pyridin-2­(1*H*)-one group and incorporated various indolyl capping groups in the P3 position. The hydrazides **16a**-**e** were prepared via HATU-supported amide formation from 1*H*-indole-2-carboxylic acid derivatives (4-methoxy-, 4,6-difluoro-, or 4,6-dichloro-) and l-leucine methyl ester hydrochloride, followed by hydrazinolysis of the methyl esters **15a-e**. The condensation of the resulting hydrazides **16a**-**e** with the respective aldehydes **17a**-**e** (except **17a**, which was commercially available), which were prepared through a butyllithium-supported formylation process from the substituted 3-bromopyridin-2-ol derivatives, resulted in the formation of the hydrazones **18a**-**i**. Subsequently, the reduction of the alkyl hydrazones to the corresponding alkyl hydrazides **19a**-**i** was accomplished using a dimethylaminoborane complex as a reducing agent and *p*-toluenesulfonic acid as a proton source. The dichloroacetamide warhead was finally attached by reaction with dichloroacetyl chloride, forming products **20a**-**i**.

**Scheme 3:**
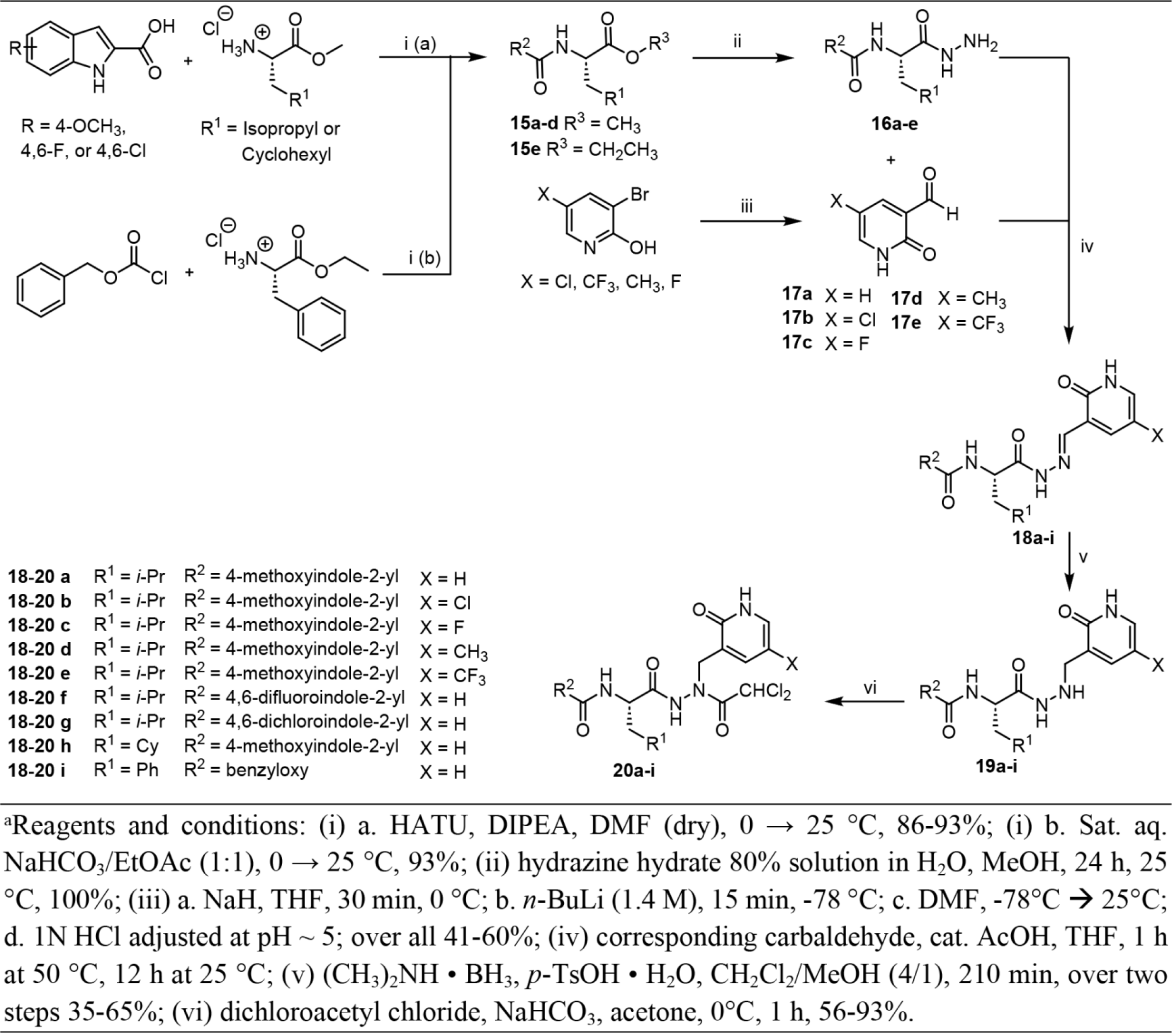
Synthesis of Compounds **20a-i**

The structures of the synthesized compounds were confirmed by ^1^H and ^13^C NMR spectroscopy. In addition, the purity of all final compounds was analyzed by HPLC at wavelengths of 254 and 230 nm. All final compounds have a purity of more than 95%. The mass spectra for all final compounds were obtained using electrospray ionization mass spectrometry (ESI-MS).

### SARS-CoV-2 M^pro^ Inhibition Assays

Following established procedures, SARS-CoV-2 M^pro^ inhibitory activity assays were performed using a fluorogenic substrate (Boc-Abu-Tle-Leu-Gln-AMC).^23,40,41^ Additionally, a well-established quenched fluorescent peptide substrate, DABCYL-KTSAVLQ|SGFRKM-EDANS (DABCYL-EDANS substrate), was used, which produces fluorescence upon M^pro^-mediated cleavage at the Q-S bond.^22,42–44^ A concentration of 10 μM was used for the initial screening of the compounds. Concentration−response curves were determined for compounds resulting in more than 50% M^pro^ inhibition. Furthermore, the effects of several distinct inhibitor concentrations on product formation were assessed over a 60-min period with continuous observation of product formation. Time-dependent inhibition is typical for irreversible inhibitors, which show a delayed potency increase, in contrast to reversible inhibitors.^45^ Thus, the determination of *k*_*inact*_/*K*_*i*_ is important for the characterization of irreversible inhibitors. For inhibitors that demonstrated time-dependent inhibition, *i.e*., an increase in inhibitory effect over the incubation period, the progress curves were analyzed using nonlinear regression, and the second-order rate constants (*k*_inact_/*K*_i_) were determined. NMV was used as a positive control, showing an IC_50_ value of 0.041 μM at SARS-CoV-2 M^pro^ (see Tables 1 and 2).^42^

### Structural Optimization of Azapeptides

An initial effort focused on the warhead of the designed compounds (Table 1). The addition of a hydroxyacetyl group to compound **5a** resulted in only an 11% inhibition of M^pro^ at 10 μM. The hydroxyl-cyano replacement (**5b**) also failed to induce inhibitory activity. Introducing an α-ketoamide-type warhead in compound **5c** resulted in moderate M^pro^ inhibition (IC_50_ = 11.1 μM). A *k*_inact_/*K*_i_ value could not be calculated for **5c** due to considerable M^pro^ reactivation within 1 h. The methyl group was replaced by a phenyl ring (**5d**), resulting in a reduction in inhibitory activity.

Next, we explored different reactive haloacetyl groups and their reactivity, as these might undergo S_N_2 reactions with the nucleophilic M^pro^ active-site Cys145. Decoration with dichloroacetyl caused good M^pro^ inhibition (compound **5e**, IC_50_ = 0.168 μM). An irreversible mode of SARS-CoV-2 M^pro^ inhibition by **5e** was kinetically determined, and the second-order rate constant of inactivation, *k*_inact_/*K*_i_, was 1.33 × 10^4^ M^−1^s^−1^. Compound **7**a, with a difluoroacetyl group, was evaluated to demonstrate the leaving group capability of chloride. In contrast to **5e**, compound **7a** completely lost inhibitory activity against M^pro^. The trihalo-substituted compound **6e** was inactive as well. However, by replacing only one chlorine atom in the α-position of **5e** with fluorine (**5f**, *k*_inact_/*K*_i_ = 3.92 × 10^3^ M^−1^s^−1^), methyl (**6d**, *k*_inact_/*K*_i_ = 7.10 × 10^4^ M^−1^s^−1^), or hydrogen (**6c**), the inhibitory activity was reestablished. As anticipated, the latter compound **6c** with the chloroacetyl exhibited the highest potency among all, with a *k*_inact_/*K*_i_ value of 5.48 × 10^4^ M^−1^s^−1^ and an IC_50_ value of 0.0755 μM.

Next, we employed C−C multiple bonds adjacent to the carbonyl group as reactive warhead groups. The addition of but-2-ynoyl in compound **5g** resulted in moderate inhibitory activity. Expectedly, the Michael acceptor units present in **5h** and **6a** served as better warheads. In particular, the decoration with the fumaric acid methyl ester function in **5h** was advantageous (*k*_inact_/*K*_i_ = 2.70 × 10^4^ M^−1^ s^−1^). Methylation at the α-position of the acryloyl group of **6a** led to the methacyloyl inhibitor **6b** with reduced activity, obviously caused by steric hindrance of the nucleophilic attack of the active-site cysteine.

To investigate the potential of pyridin-2­(1*H*)-one as a P1 group, we used **6c** as a lead and explored various P1 modifications. Initially, the phenyl ring was inserted in place of the lactam, which resulted in compound **8a** with an approximately 3-fold increased IC_50_ value (see **8a** vs **6c**). This implied the pyridin-2­(1*H*)-one group to be involved in advantageous hydrogen bond interactions. A fluoro (**8b**) or chloro (**8c**) substitution at the *m*-position of the phenyl ring was tolerated. Still, the introduction of a bulky substituent, such as methoxy, in addition to chloro **(8d**) or trifluoromethoxy (**8e**), reduced the inhibitory potency. The replacement of pyridin-2­(1*H*)-one with aza-aromatic groups retained the inhibitory activity of the resulting compounds **8f**-**h**. When a quinolin-2­(1*H*)-one (**8i**) was incorporated, the *k*_inact_/*K*_i_ was found to be even higher (1.23 × 10^5^ M^−1^s^−1^) than for **6c** (5.48 × 10^4^ M^−1^s^−1^), indicating more effective inactivation. Interestingly, replacements with the 5-chloropyridin-2­(1*H*)-one and pyrimidine-2,4­(1*H*,3*H*)-dione resulted in compounds **8j** and **8k,** which provoked enhanced M^pro^ inhibition compared to **6c**. In particular, **8j** was equipotent to the approved drug NMV. These results are summarized in Table 1.

**Table 1:**
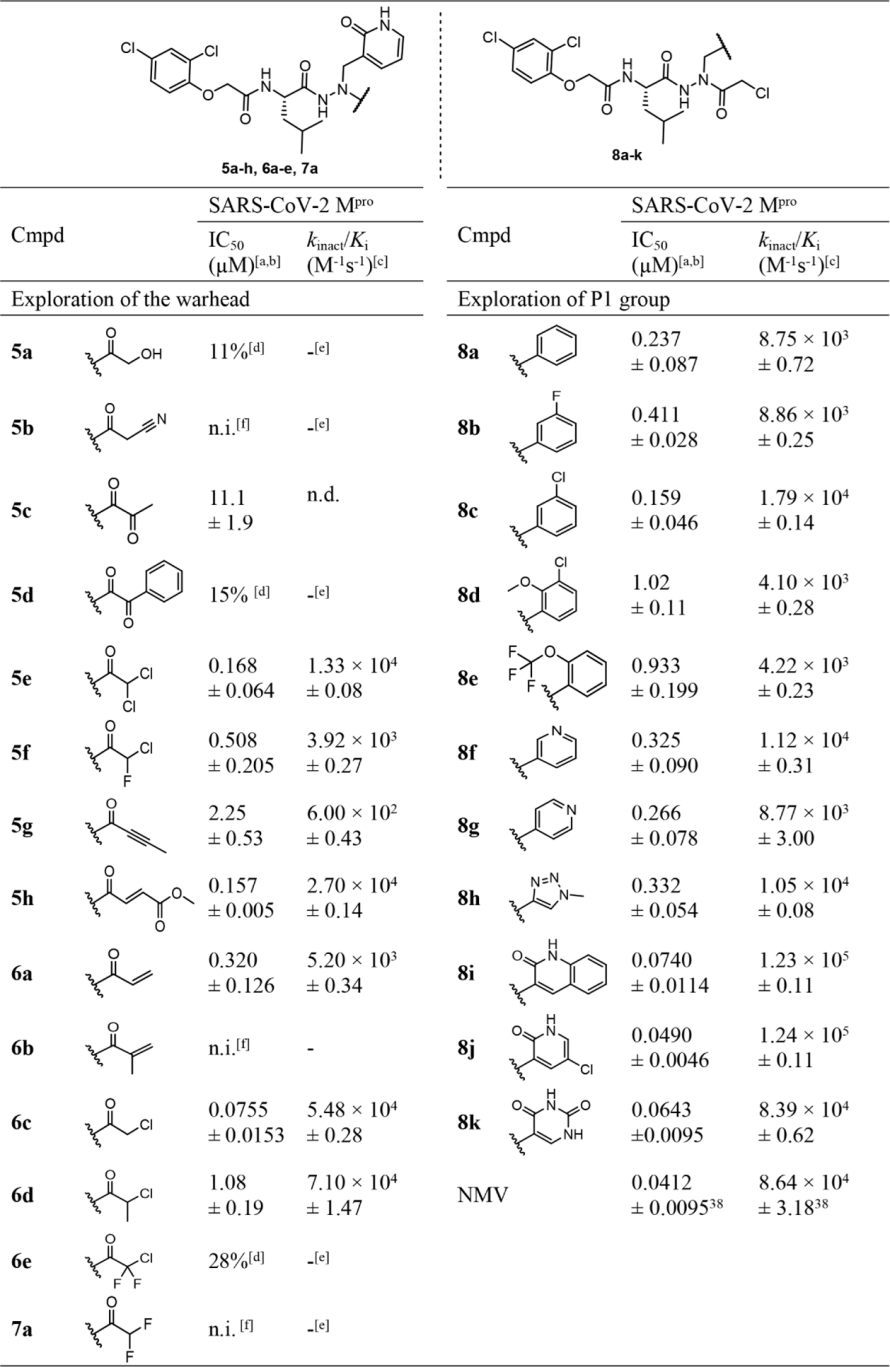
Chemical Structures and M^pro^ Inhibition by Compound **5a**-**h**, **6a**-**e**, **7a**, **8a**-K^a^ ^b^ ^c^ ^d^ ^e^ ^f^ ^g^

a IC_50_ values were obtained from duplicate measurements with at least eight inhibitor concentrations. The equation for nonlinear regression was *v* = *v*_0_/(1 + [I]/IC_50_), where v is the product formation rate at different inhibitor concentrations, *v*_0_ is the uninhibited product formation rate, [I] is the inhibitor concentration, and IC_50_ is the half-maximal inhibitory concentration. The standard errors (SE) refer to the nonlinear regression.

b The final substrate concentration of the fluorogenic substrate Boc-Abu-Tle-Leu-Gln-AMC was 50 μM, and the formation of the product was monitored with excitation and emission wavelengths of 360 and 460 nm, respectively. The reactions were followed for 60 min at 37 °C. Detailed assay conditions are reported elsewhere.^23^

c Inhibitors showed a time-dependent inhibition. Progress curves in the presence of five different inhibitor concentrations were followed over 60 min and analyzed by nonlinear regression using the equation [P] = *v*_i_ × (1−exp­(−*k*_obs_ × t)/*k*_obs_ + d), where [P] is the product, *v*_i_ is the initial rate, *k*_obs_ is the observed first-order rate constant and d is the offset. The second-order rate constant, k_inac_/*K*_i_, was determined by plotting k_obs_ versus [I] and nonlinear regression using the equation *k*_obs_ = (*k*_inac_ × [I])/([I] + *K*_i_ × (1 + [S]/*K*_m_)).^23^

d % inhibition at 10 μM.

e Not tested.

f No inhibition at 10 μM.

g Not determined.

Following the initial structure−activity relationship (SAR) investigations for S1 and S1′ regions, compounds bearing variable substructures capable of interacting with the S2 pocket were investigated (see Table 2). For this purpose, the isopropyl residue of the l-leucine residue in **6c** (IC_50_ = 0.0755 μM) was initially modified by monofluorination (**14a**), methylation (**14b**), and cyclization (**14c**). These changes led to maintaining or increasing the inhibitory potency of **6c**. Notably, an improvement was mainly observed when leucine was replaced with *tert*-butyl-alanine (**14b**, IC_50_ = 0.0494 μM). This result suggested that a slightly larger residue than **6c** was tolerated. Indeed, introducing the phenyl group resulted in a compound, **14d**, that showed activity like that of the tert-butyl derivative, **14b**. An *o-*fluoro (**14e**) and, particularly, an *m*-fluoro (**14f**) substitution on the phenyl ring further improved the inhibitory potency. Thus, we evaluated the di-*m*-fluoro-substituted derivative **14h**, which exhibited potent inhibitory activity with an IC_50_ value of 0.0252 μM. Replacing *m*-fluorine with *m*-chlorine resulted in compound **14i** (IC_50_ = 0.0249 μM), which exhibited a similar potency level. Introducing a bulky cyclohexyl group in compound **14j** perpetuated the inhibitory activity (IC_50_ = 0.0305 μM). These results encouraged us to gain insight into SARs by exploring the S2 region.

Subsequently, several cyclic structures were embedded at the P2 position. Introducing a dihydro-indene moiety (**14k**) resulted in similar inhibitory potency as in the case of **6c**. l-Proline in **14l** seemed unsuitable for this position as the inhibitory activity declined to 1.32 μM. On the other hand, the compound with a dimethyl-3-azabicyclo[3.1.0]­hexanyl residue in the P2 position (**14m**), as present in NMV, showed excellent inhibitory potency with an IC_50_ of 0.0217 μM. The subsequent generation of bicyclic analogs with an indoline residue (**4n**) increased M^pro^ inhibitory potency by 2-fold compared to its pyrrolidine-substituted analog **14l**. However, dearomatizing the indoline to a (3a*S*,7a*S*)-octahydro-1*H*-indole (**14o**) reduced the activity to 1.15 μM, indicating this bicyclic system to be too bulky for the S2 pocket. There was a noticeable increase in inhibitory activity upon replacing proline (**14l**) by homoproline (**14p**) by more than 10-fold or by converting indoline (**14n**) to tetrahydroisoquinoline (**14q**) by more than 20-fold. These results suggested that a tetrahydrosioquinoline ring effectively occupies the S2 pocket. Compound **14r** was identified as the most active M^pro^ inhibitor in our present work, showing increased activity with an IC_50_ of 0.0133 μM. A *k*_inact_/*K*_i_ could not be calculated for **14r** due to M^pro^ reactivation. Compound **14r** was designed during a ring expansion of the potent compound **14m**. A comparison of the saturated bicyclic substructures of **14r** (IC_50_ = 0.0133 μM) and **14o** (IC_50_ = 1.15 μM) revealed a strong influence of subtle structural changes in the P2 position. The analogous compound with the same P2 residue as **14r**, but the dichloroacetyl warhead (**14s**), also displayed remarkable inhibition characteristics, with an IC_50_ of 0.0306 μM and a *k*_inact_/*K*_i_ value of 1.54 × 10^5^ M^−1^s^−1^.

Next, inhibitors with a dichloroacetyl warhead combined with different P3 and P1 residues were explored. The compound with a 4-methoxyindole (**20a** or **TPG-20a**) as a P3-capping group was a highly effective M^pro^ inhibitor, with an IC_50_ value of 0.0278 μM. Substitutions leading to the chloro (**20b**), fluoro (**20c**), methyl (**20d**), and trifluoromethyl (**20e**) on the P1-pyridin-2­(1*H*)-one ring did not result in an improvement in M^pro^ inhibition compared to **20a**. The replacement of the P3-interacting 4-methoxyindole by 4,6-difluoroindole (**20f**), 4,6-dichloroindole (**20g**), or benzyloxy (**20i**) produced compounds with M^pro^ inhibitory activity comparable to that of **20a**. The second-order rate constants of inactivation, *k*_inact_/*K*_i_, for compounds **20a**−**i** could not be calculated because they probably inhibited the enzyme via a noncovalent mechanism.

**Table 2:**
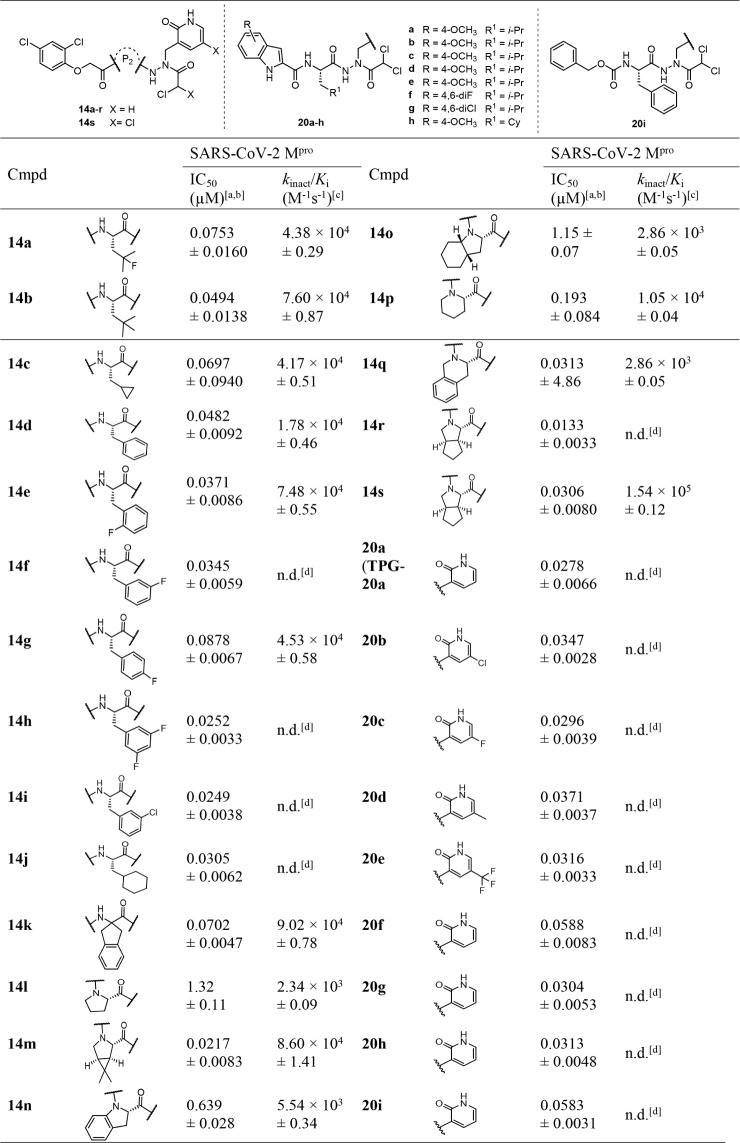
Chemical Structures and Inhibition of M^pro^ by P2-Modified Inhibitors **14a-S** and 20a-I^a^ ^b^ ^c^ ^d^

a IC_50_ values were obtained from duplicate measurements with at least five inhibitor concentrations. The equation for nonlinear regression was *v* = *v*_0_/(1 + [I]/IC_50_), where v is the product formation rate at different inhibitor concentrations, *v*_0_ is the uninhibited product formation rate, [I] is the inhibitor concentration, and IC_50_ is the half-maximal inhibitory concentration. The standard errors (SE) refer to the nonlinear regression.

b The final substrate concentration of the fluorogenic substrate Boc-Abu-Tle-Leu-Gln-AMC was 50 μM and the formation of the product was monitored with excitation and emission wavelengths of 360 and 460 nm, respectively. The reactions were followed for 60 min at 37 °C. Detailed assay conditions are reported elsewhere.^23^

c Inhibitors showed a time-dependent inhibition. Progress curves in the presence of five different inhibitor concentrations were followed over 60 min and analyzed by nonlinear regression using the equation [P] = *v*_i_ × (1−exp­(−*k*_obs_ × t)/*k*_obs_ + d), where [P] is the product, *v*_i_ is the initial rate, *k*_obs_ is the observed first-order rate constant, and d is the offset. The second-order rate constant, k_inac_/*K*_i_, was determined by plotting k_obs_ versus [I] and nonlinear regression using the equation *k*_obs_ = (*k*_inac_ × [I])/([I] + *K*_i_ × (1 + [S]/*K*_m_)).^23^

d n.d. = not determined.

### Cytotoxicity and Antiviral Activity

Selected M^pro^ inhibitors representing a diversity of structures (**6c**, **8j**, **14r**, **14s**, **20a**, **20f**, **20h**, **20g**) were tested at concentrations ranging from 10 to 100 μM for their cytotoxic effects on two different cell lines, VeroE6 and HeLa^ACE2-TMPRSS2^ cells^46^ (Table 3). Most tested compounds showed no significant cytotoxicity up to 100 μM. Subsequently, antiviral activity was tested in multiple cell lines, using different procedures detailed in the methods section. The P-glycoprotein inhibitor CP-100356 was not used in either method. The first method of evaluating EC_50_ was RT-qPCR-based, where the antiviral activity of the tested compounds was found to be better against SARS-CoV-2^B.1.1^ in HeLa ACE2-TMPRSS2 cells than against SARS-CoV-2^WK521^ in VeroE6 cells. The second method used the virus-induced cytopathic effect (CPE) in Vero E6 cells expressing human ACE2 and TMPRSS2 (Vero AT).^47^ To determine the EC_50_, live attenuated SARS-CoV-2 was used to infect Vero AT cells with different concentrations of antiviral compounds (20 μM to 5-fold dilutions, spanning 10 points).^21^ For these experiments, mean values and standard deviation (SD) are shown for individual data points from 2 to 6 replicates (NMV: 4 replicates; ESV: 6 replicates; candidate inhibitors: 2 replicates).

**Table 3:** Antiviral Activity and Cytotoxicity of Selected M^pro^ Inhibitors

	EC_50_ (μM)^a^		EC_50_ (μM)^b^	CC_50_ (μM)^c^	
Cmpd.	SARS-CoV-2^WK521^	SARS-CoV-2^BQ.1.1^	SARS-CoV-2	VeroE6	HeLa^ACE2-TMPRSS2^
**NMV**	1.4 ± 0.2	0.10 ± 0.03	1.14 ± 0.10	>100	>100
**ESV**	-	-	0.06 ± 0.02		
**6c**	32 ± 1.4	1.1 ± 0.5	-	38 ± 1	43 ± 2
**8j**	9.9 ± 0.2	1.8 ± 1.0	-	37 ± 1	29 ± 2
**14r**	3.2 ± 0.4	0.78 ± 0.09	-	33 ± 2	43 ± 3
**14s**	8.6 ± 0.7	0.90 ± 0.07	>20	>100	46 ± 3
**20a**	3.9 ± 0.2	0.46 ± 0.11	0.63 ± 0.06	>100	>100
**20f**	4.0 ± 0.2	0.38 ± 0.09	0.71 ± 0.18	>100	>100
**20h**	2.9 ± 0.4	0.67 ± 0.16	1.08 ± 0.04	>100	>100
**20g**	12 ± 3	0.80 ± 0.21	6.2 ± 10	42 ± 1	45 ± 2
**20i**	-	-	3.2 ± 0.59	-	-

a The cell-based anti-SARS-CoV-2 activity of the compounds was determined using RT-qPCR assays of viral RNA from SARS-CoV-2^WK-521^-exposed VeroE6 cells or SARS-CoV-2^BQ.1.1^-exposed HeLa^ACE2-TMPRSS2^ cells.

b The cell-based anti-SARS-CoV-2 activity of the compounds was determined using the virus-induced cytopathic effect (CPE) in Vero E6 cells expressing human ACE2 and TMPRSS2 (Vero AT).

c The cytotoxicity of the compounds was evaluated in noninfected VeroE6 cells using the water-soluble MTT assay.

The RT-qPCR-based evaluation demonstrated that the compounds **14r** (EC_50_ 3.2 μM), **20a** (EC_50_ 3.9 μM), **20f** (EC_50_ 4.0 μM), and **20h** (EC_50_ 2.9 μM) have comparable antiviral activity to NMV (EC_50_ 1.35 μM). In contrast, compounds **6c**, **8j**, **14s**, and **20g** demonstrated moderate activity against SARS-CoV-2^WK521^ in VeroE6 cells. Interestingly, all tested compounds were identified as more potent antivirals against SARS-CoV-2^BQ.1.1^ in HeLah^ACE2-TMPRSS2^ cells, especially compounds **14r** (EC_50_ = 0.78 μM), **14s** (EC_50_ = 0.90 μM), **20a** (EC_50_ = 0.46 μM), **20f** (EC_50_ = 0.38 μM), **20h** (EC_50_ = 0.67 μM), and **20g** (EC_50_ = 0.80 μM). In the CPE-based experiments, NMV and ESV were used as positive controls (EC_50_ values of 1.1 μM and 0.06 μM, respectively). Compounds **20a** (EC_50_ = 0.63 μM) and **20f** (EC_50_ = 0.71 μM) exhibited high antiviral activity, being somewhat more potent than NMV (EC_50_ = 1.14 μM), and compound **20h** (EC_50_ = 1.1 μM) demonstrated an antiviral potency comparable to NMV. However, compounds **14s**, **20g**, and **20i** displayed only moderate antiviral activity (Figure S2, Panel A). The IC_50_ values of compounds **14s** and **20g** should be interpreted with caution due to compound toxicity at higher concentrations in the CPE assay. The crystal violet-stained cells (5-fold serial dilutions from left to right, starting at 20 μM) reflect the observed antiviral EC_50_ values. As expected, ESV showed the most potent antiviral activity, and NMV antiviral activity was moderate. The compounds **20a**, **20f**, and **20h** exhibited comparable or slightly better antiviral activity than NMV. (Figure S2, Panel B).

### X-ray Cocrystal Structure of the M^pro^ Inhibitor 20a

To further our understanding of the interactions between the target and the inhibitor, we obtained an X-ray crystal structure of M^pro^ in complex with the potent inhibitor **20a** (Figure 2A,B; PDB: 9MDQ). The structure was solved at a resolution of 1.6 Å in the *C2* space group. The asymmetric unit has a single monomer, which forms a dimer as a known biologically active form along the crystallographic 2-fold axis. An apparent electron density of compound **20a** was observed in the structure. The M^pro^-**20a** complex is stabilized by covalent and noncovalent interactions with the residues at the substrate binding pocket (S1’, S1, S2, and S3). The crystal structure reveals that the catalytic C145 nucleophilically attacked the α-carbon of the warhead in **20a**, forming a covalent bond of 1.88 Å. This bond length is identical to the average length of single C−S bonds, 1.88Å,^48^ indicating that a carbon-sulfur single bond was formed, as expected from a nucleophilic attack of the active-site cysteine and the repulsion of one chloride during the nucleophilic substitution. The mechanism of interaction of **20a** with M^pro^ is unique, as the dichloroacetamide warhead has two Cl atoms, with one undergoing a covalent reaction with the C145-thiolate. Ma et al.^49^ and Khatua et al.^50^ reported on the crystal structures of corresponding complexes of M^pro^ with dichloroacetamide-based inhibitors. Interestingly, in the case of **20a**, the electron density for the second Cl atom is not observed in our 1.6 Å structure (Figure S1A). A Polder map^51^ (Figure S1B) shows positive difference density to suggest that the compound may not have undergone the complete transformation cycle (Figure S1C) required to release from the protein.^52^ The loss of the second chlorine might have been attributed to a consecutive reaction of the expected monochloro intermediate CO-CHCl-SCH_2_R. Removal of chloride might have led to a sulfenium ion CO-CH=S^(+)^CH_2_R, or elimination of HCl to a thiocarbonyl ylide CO-CH=S^(+)^CH^(-)^R. However, both constitute high-energy structures^53,54^ with an assumed shorter C=S bond length. To further investigate the mechanism, **20a** was modeled as intermediate 3 covalently bound to C145 (Figure S1B) with an alternate conformation of unbound C145 present. The high-resolution Polder map shows additional alternate conformations for nearby side chains of H41 and M165, consistent with established active-site^55^ flexibility and dynamic disorder for residues 45−49 (Figure S1B), indicative of plasticity in the loop region of the active site.

The residues of the oxyanion hole, *i.e*., the amide backbone of G143 and the amide backbone of S144, interact with the carbonyl group of the halo acetyl moiety by a hydrogen bond to help position the compound. The M^pro^ enzyme has a stringent glutamine requirement in the P1 position, which occupies the S1 subsite. The S1 pocket is occupied by the six-membered lactam unit of **20a**, which mimics the Glu residue. Here, the lactam’s NH forms an H-bond with the Oε1 side chain of E166 and the main chain of F140, and its C­(O) interacts with Nε2 of H163. SAR data support the significance of the lactam group’s interactions with the enzyme, as shown in exchanges **8a**-**h**, which exhibit significantly reduced potency (see Table 1). The isobutyl group of the P2 L-leucine occupies the S2 subsite and interacts with H41 and M165 by hydrophobic interaction (bond length greater than 3.5 Å). The L-Leu NH forms an H-bond with the backbone Glu166, while the 4-methoxyindole extends toward the S3 and S4 subsites. The carbonyl oxygen at the second position of the indole forms an H-bond with the main-chain nitrogen of E166, and the indole NH proton forms an H-bond with the carbonyl oxygen of E166.

**Figure 2:**
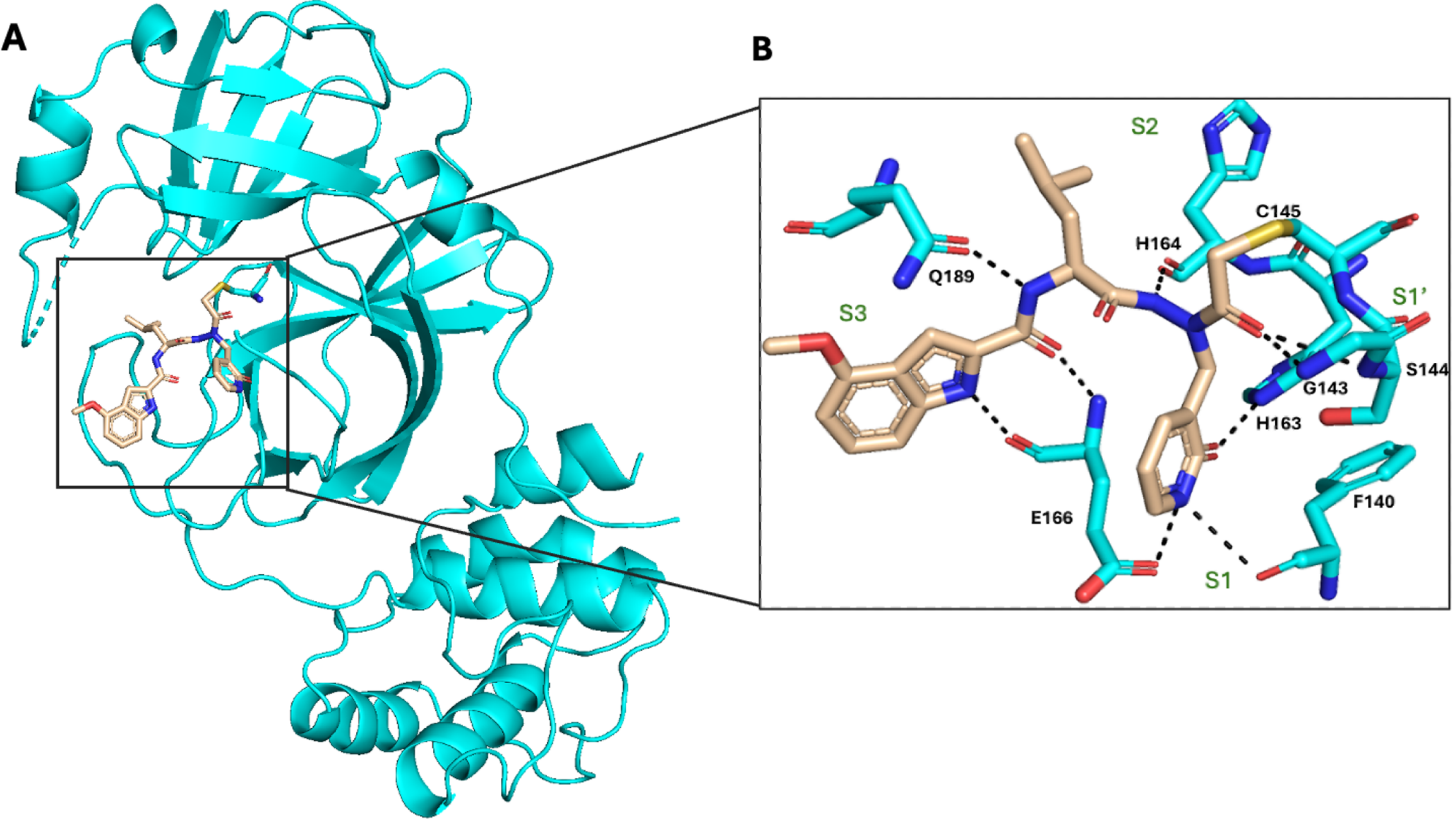
Cocrystal structure of **20a** with SARS-CoV-2 M^pro^ [PDB: 9MDQ]. The M^pro^ (cyan) complex with **20a** (tan) (a). Zoomed-in view of the M^pro^ substrate binding pocket in the presence of the **20a** compound. Subsites (S1’, S1, S2, and S3) are labeled in green. C145 forms a covalent bond (yellow) with the α-carbon of the warhead. Hydrogen bonds are depicted as a black dotted line (b).

The detailed binding interactions between **20a** and the protease were additionally studied by isothermal titration calorimetry (ITC) (Table 4). The covalent inhibitor GC376 and noncovalent ensitrelvir were used as reference compounds. It was observed that the binding affinity of **20a** (*K*_D_ = 43.1 nM) is greater than that of controls GC376 (*K*_D_ = 337 nM) and ensitrelvir (*K*_D_ = 63.5 nM). Additionally, the reaction was exothermic, and the high negative value for the binding enthalpy (ΔH), ΔG, and the positive value for the entropy change (ΔS) indicate a strong interaction between **20a** and M^pro^. The 0.68 value for N [sites] indicates stoichiometry, suggesting that the two active sites in M^pro^ are unequally occupied under experimental conditions (*i.e*., partial occupancy or possible negative cooperativity). The 0.52, *N* value for noncovalent ensitrelvir also supports our data. These values reflect preferential ligand occupancy of one site in the dimer. Albani et al. (2023) reported that the ligand binding can induce asymmetric stabilization and dynamic behavior between the two protomers.^56^ The increasing order of ΔH, GC376 < ensitrelvir < **20a**, underscores the previously acquired biochemical data for **20a**, which exhibits a strong binding capability to the M^pro^ active site.

**Table 4:** Inhibitor Kinetics by Isothermal Titration Calorimetry Assay^a^

	GC376	ensitrelvir	**20a**
*K*_D_ [nM]	337	63.5	43.1
ΔH [kcal/mol]	-4.83 ± 0.338	-14.5 ± 0.387	-25.4 ± 0.473
ΔG [kcal/mol]	−8.83	−9.82	−10.1
-TΔ*S*[kcal/mol]	−4.01	4.72	15.3
N [sites]	0.857 ± 3.8*e^−2^	0.520 ± 9.3*e^−3^	0.680 ± 6.9*e^−3^

a *K*_D_, dissociation constant; ΔH, binding enthalpy; ΔG. Gibbs free energy, ΔS, entropy, and N, reaction stoichiometry.

#### Testing at M^pro^ Mutants

It has been observed that SARS-CoV-2 M^pro^ with several naturally occurring mutations exhibits resistance to approved medications, such as NMV. Notably, the mutations Glu166Val (*K*_i_ > 10 μM), Glu166Asn (resulting in a more than 600-fold increase in IC_50_), the triple mutant Leu50Phe/Glu166Ala/Leu167Phe (72-fold increase in IC_50_ and 51-fold increase in EC_50_), and the Ser144Ala variant (resulting in a more than 90-fold reduction in *K*_i_) raise concerns that the drug may become ineffective in certain instances.^19,57^ A significant problem is the emergence of such mutations in future virus strains. Therefore, we investigated the efficacy of our potent compound, **20a**, at several naturally occurring M^pro^ variants. A panel of 16 mutated M^pro^ enzymes was expressed and assessed for inhibition of their enzymatic activity by **20a** and NMV (Table 5) using the FRET assay with the DABCYL-EDANS substrate.^58^

**Table 5:** IC_50_ Values of NMV and **20a** at Different SARS-CoV-2M^pro^ Mutants^a^

	NMV		**20a**	
M^pro^ variant	IC_50_ (nM)	IC_50_ Fold Increase	IC_50_ (nM)	IC_50_ Fold Increase
Wild type	10.8 ± 0.2	1.0	27.8 ± 6.6	1.0
S144A	45.8 ± 4.4	4.0	59.0 ± 6.6	2.0
S144F	197 ± 9	18.0	367 ± 16	13.0
S144G	215 ± 21	20	364 ± 24	13.0
S144Y	198 ± 45	18.0	631 ± 32	23.0
M165T	162 ± 3	15.0	141 ± 2	5.0
H172F	156 ± 9	14.0	187 ± 4	7.0
H172Q	174 ± 12	16.0	235 ± 20	9.0
Q192S	258 ± 3	24	301 ± 10	11.0
L50F/E166A/L167F	514 ± 34	48.0	291 ± 5	11.0
G143S	138 ± 4	13.0	147 ± 2	5.0
E166V	2470 ± 21	229.0	274 ± 21	10.0
E166N	6690 ± 98	619.0	536 ± 14	19.0
H172Y	275 ± 17	25.0	333 ± 1	12.0
Q189E	214 ± 22	20.0	417 ± 63	15.0
ΔP168	45.3 ± 1	4.0	46.4 ± 1	2.0
A173V	80. 5 ± 3.4	8.0	46.1 ± 4	2.0
		30−100	10−30	>100

a Data are shown as mean ± SD.

NMV showed reduced efficacy against all M^pro^ mutants tested (Table 5). Notably, the Glu166Val and Glu166Asn mutants exhibited the highest resistance, with IC_50_ values of 2.47 and 6.69 μM, respectively, representing a 228- and 619-fold decrease in potency compared to the wild-type enzyme. In contrast to NMV, compound **20a** exhibited more robust activity, with a less pronounced drop-in activity, against all mutations. Compound **20a** exhibited significant potency against the Glu166Val mutant, with an IC_50_ value of 0.274 μM (only 9.9 times lower than the wild-type enzyme), suggesting that **20a** may partially overcome NMV resistance.

### Antiviral Activity of Selected M^pro^ Inhibitors against SARS-CoV-2 ^E166V^

We then examined the antiviral potency of specific M^pro^ inhibitors, including **20a**, against a SARS-CoV-2 variant that contains solely the E166V substitution (Table 6).^17^ To achieve this, we generated a new SARS-CoV-2 variant with a single E166V substitution (rgSARS-CoV-2^E166V^) alongside its parental wild-type strain (rgSARS-CoV-2WT) using reverse genetics technology.^46^ We assessed the susceptibility of this recombinant SARS-CoV-2 to antiviral agents. Compounds **6c**, **8j**, **14r**, and **14s** exhibited low micromolar antiviral activity in VeroE6 cells with 2 μM CP-100356, ranging from 0.29 to 0.033 μM, against the wild type (WT) rgSARS-CoV-2. The maximum reduction in their activities against the rgSARS-CoV-2 ^E166V^ mutant was only 20-fold. In contrast, the EC_50_ value of NMV against rgSARS-CoV-2WT was 0.03 μM in VeroE6 cells with 2 μM CP-100356, which increased to 1.6 μM against rgSARS-CoV-2^E166V^. The fold-difference comparison shows an ∼55-fold decrease in NMV potency. Compounds with a P3-indole cap, such as **20a**, **20f**, **20h**, and **20g**, showed highly potent antiviral activity against rgSARS-CoV-2^WT^, which is 3 to 10 times more powerful than NMV. Interestingly, their efficacy against rgSARS-CoV-2^E166V^ increased several-fold compared to the wild type. Compounds **20a**, **20h**, and **20g** showed 10- and 20-fold improvement, respectively. SARs suggest that the P3-indole cap may favor crucial interactions with the protease, thereby enhancing antiviral activity against rgSARS-CoV-2 ^E166V^.

**Table 6:** Antiviral Activity and Cytotoxicity of Selected M^pro^ Inhibitors against rgSARS-CoV-2^WT^ and rgSARS-CoV-2^E166V^ [^a^]

	EC_50_ (μM)[^b^]		
Cmpd.	rgSARS-CoV-2^WT^	rgSARS-CoV-2^E166V^	CC_50_ (μM)
NMV	0.030 ± 0.001	1.64 ± 0.78	>100
**6c**	0.36 ± 0.02	0.72 ± 0.53	48 ± 2
**8j**	0.61 ± 0.47	0.797 ± 0.010	40 ± 3
**14r**	0.033 ± 0.06	0.296 ± 0.030	40 ± 3
**14s**	0.29 ± 0.03	8.18 ± 1.94	46 ± 2
**20a**	0.002 ± 0.002	0.0005 ± 0.0002	>100
**20f**	0.0041 ± 0.0003	0.0056 ± 0.0011	56 ± 2
**20h**	0.0031 ± 0.0001	0.0003 ± 0.0001	53 ± 2
**20g**	0.01 ± 0.01	0.0005 ± 0.0001	50 ± 1

a A recombinant SARS-CoV-2 variant with only the E166V substitution (rgSARS-CoV-2^E166V^) and a recombinant wild-type virus (rgSARS-CoV-2^WT^) were created through reverse genetics technology. The susceptibility of both strains was determined against NMV. Data are from three independent assays representing arithmetic means (μM) ± 1 SD.

b Each inhibitor was coincubated with 2 μM CP-100356, and the EC_50_ values were calculated. (Multiplicity of Infection (MOI): rgSARS-CoV-2^WT^, 100; rgSARS-CoV-2^E166V^,100.

#### Broad-Spectrum M^pro^ Inhibitory Activities and Off-Target Selectivity

In this study, we employed our well-established SARS-CoV-2 M^pro^ inhibition assays, which used an AMC substrate to test compounds for M^pro^ inhibition. Next, we investigated the M^pro^ inhibitory activity of selected potent SARS-CoV-2 M^pro^ inhibitors (**14s**, **20a**, **20f, 20g**, **20h**, and **20i**) using the commercially available DABCYL-EDANS substrate. The results showed that the potency of the compounds was greatly improved, ranging approximately from 2- to 10-fold in assays using the DABCYL-EDANS substrate (Table 7). Compounds were then tested against recombinant MERS-CoV M^pro^ at a high concentration of 10 μM employing the fluorescence resonance transfer (FRET) assay.^9,59,60^ We identified compounds that were effective inhibitors of the MERS-CoV M^pro^ in the submicromolar to nanomolar range, especially compounds **20a**, **20f,** and **20h**, which were more potent inhibitors than NMV. The rank order of potency at MERS-CoV M^pro^ is as follows: **20a** > **20h** > **20f** > **20g** > **14s**. Most notably, inhibitors did not inhibit at least 50% of the human CatL at 10 μM. Thus, azapeptides represent a novel class of broad-spectrum inhibitors that target M^pro^ of SARS-CoV-2 and MERS-CoV.

**Table 7:** SARS-CoV-2 M^pro^ and MERS-CoV M^pro [a]^ Inhibitory Activity of Selected Compounds

	SARS-CoV-2 M^pro^	MERS-CoV M^pro^	Human CatL
Compd.	IC_50_ (μM)[^a^]	IC_50_ (μM)[^b^]	Inhibition (%) at 10 μM
**14s**	0.00640	0.272 ± 0.090	2
**20a**	0.00710	0.0429 ± 0.0058	5
**20f**	0.00182	0.0577 ± 0.0081	1
**20g**	0.0142	0.118 ± 0.003	4
**20h**	0.00931	0.0498 ± 0.0049	4
**20i**	0.00593	n.t.[^c^]	n.t.[^c^]
NMV	0.0122	0.0881 ± 0.0074	n.t.[^c^]

a MERS-CoV M^pro^ IC_50_ values from two independent assays performed in triplicate (*n* = 6 data points) were calculated by nonlinear regression (r² > 0.9). The analysis considered the standard deviation of each point in each curve, and the deviations were less than 10%. Errors are given by the ratio of the standard deviation to the square root of the number of measurements (*n* = 6).

b The DABCYL-EDANS substrate was used.

c n.t. not tested.

## Conclusions

M^pro^ plays a crucial role in the viral cycle and is highly conserved across human coronaviruses, making it an ideal target for the development of antiviral drugs. Numerous M^pro^ inhibitors have been reported since late 2024.^21,60–68^ However, a lack of efficacy and/or a poor pharmacokinetic profile hinders further development of most of these compounds. Although NMV, a commercially available COVID-19 antiviral drug, demonstrated significant M^pro^ inhibition, it is inefficient against several naturally occurring mutations in M^pro^. Thus, developing new M^pro^ inhibitors with modified chemical structures and altered mechanisms of action is essential. In this work, we described the design, synthesis, and structural optimization of novel azapeptide-based SARS-CoV-2 M^pro^ inhibitors, several of which had low nanomolar IC_50_ values. These azapeptide compounds showed promise as broad-spectrum antiviral candidates by inhibiting the M^pro^ of MERS-CoV. Compounds **20a** and **20f** exhibited potent antiviral activity in Vero E6 cell lines. The covalent adduct involving catalytic Cys145 was experimentally identified via a cocrystal structure of SARS-CoV-2 M^pro^ with **20a**. Compound **20a** also demonstrated efficacy against a panel of naturally occurring NMV-resistant mutants, particularly the Glu166Val M^pro^ mutant, and exhibited potent antiviral activity against rgSARS-CoV-2E166V. To conclude, we introduce M^pro^ inhibitor **20a** (**TPG**-**20a**) as a promising lead compound for future optimization *in vivo* studies and the development of antiviral agents to combat resistant mutants.

## Methods

### General Chemistry

All commercially available starting materials, reagents, and (anhydrous) solvents were used without further purification. Reaction controls were performed by thin-layer chromatography (TLC) on Macherey-Nagel-precoated 60 F254 silica plates. Spots were visualized by ultraviolet (UV) light (254 nm) or staining solutions. Flash column chromatography was carried out using Grace Davison Davisil LC60A (20−45 μm) or Merck Geduran Si60 (mesh 63−200 μm) with a LaFlash automated flash chromatography system. NMR spectra were recorded on a Bruker Avance 400 MHz spectrometer at ambient temperature. Chemical shifts (δ) are reported in parts per million (ppm) relative to the internal control tetramethylsilane (TMS), and the spectra were calibrated against the residual solvent peak of the used deuterated solvent. Coupling constants (*J*) are expressed in Hz. Purities of final compounds were determined by RP-HPLC using an Agilent 1100 Series LC with a Phenomenex Luna C8 analytical column (150 × 4.6 mm, 5 μm) and detected by a UV-DAD detector at 254 and 230 nm wavelengths. The elution was carried out with the following gradient: (A = 0.01 M KH_2_PO_4_, pH 2.30, B = MeOH) 40% B to 85% B in 8 min, 85% B for 5 min, 85% to 40% B in 1 min, 40% B for 2 min, stop time 16 min, flow 1.5 mL/min. Standard mass spectra were obtained from an Advion Expression Compact mass spectrometer (electrospray ionization, ESI) with a TLC plate reader system (using the following settings: ESI voltage 3.50 kV, capillary voltage 187 V, source voltage 44 V, capillary temperature 250 °C, desolvation gas temperature 250 °C, gas flow 5 L/min). High-resolution mass spectra (ESI) of the final compounds were obtained from the Mass Spectrometry Department, Eberhard Karls Universität Tübingen. All final compounds are ≥ 95% pure by HPLC.

### General Procedure A for the HATU-Supported Amide Synthesis

The corresponding carboxylic acid (1 equiv. in DMF, 5 mL) was added to the solution at 0 °C, followed by the sequential addition of HATU (1.2 equiv.). The solution was maintained at 0 °C for 30 min. Subsequently, diisopropylethylamine (DIPEA) (3 equiv.) and the coupling partner amine (1 equiv.) were added in a slow, controlled manner. The mixture was stirred at 0 °C for 1 h and at 25 °C for 12 h. The reaction mixture was quenched by adding water, and the organic phase was extracted with EtOAc (three times, 15 mL each). The combined organic phases were washed with a saturated solution of ammonium chloride (2 × 25 mL), a saturated solution of sodium bicarbonate (2 × 25 mL), and a solution of sodium chloride (2 × 25 mL). The organic layer was dried over anhydrous sodium sulfate, filtered, and concentrated under reduced pressure. The resulting crude material was purified by column chromatography.

### General Procedure B for the Synthesis of Hydrazides

To a stirred suspension of the ester (1.0 equiv. ratio) in 5 mL of methanol, hydrazine monohydrate (10 equiv. ratio, 80% solution in water) was added slowly. The reaction mixture was stirred at 25 °C for 24 h. The mixture was concentrated under reduced pressure, isolating the product as a white solid. The resulting crude material was subjected to the subsequent step without additional purification.

### General Procedure C for the Synthesis of Hydrazones

The hydrazide derivative (1 equiv.) was dissolved in THF (dry, 10 mL), followed by the addition of the aldehyde (1 equiv.) and catalytic acetic acid (1−2 drops). The solution was then permitted to stir at 50 °C for 1 h and at 25 °C overnight. Once the reaction had reached completion, the mixture was concentrated under reduced pressure. The resulting crude material was utilized in the subsequent step without additional purification.

### General Procedure D for the Reduction of Hydrazones

To the solution of the corresponding hydrazone (1 equiv. in CH_2_Cl_2_ (dry, 16 mL) and MeOH (HPLC-grade, 4 mL), *p*-toluenesulfonic acid monohydrate (6 equiv.) and borane dimethylamine complex (1.60 equiv.) were added at 0 °C (ice bath). The reaction mixture was then allowed to reach room temperature and stirred for 90 min. A second addition of the borane dimethylamine complex (1.60 equiv.) was made, and the mixture was stirred for 120 min. The reaction was quenched with an 8 mL solution of 1.5 M NaOH, and the biphasic system was stirred for a period of 30 min. The mixture was diluted with water (10 mL) and CH_2_Cl_2_ (15 mL), and the resulting solution was separated into its constituent phases. The aqueous layer was neutralized and extracted with CH_2_Cl_2_ (three times, 20 mL each). The combined organic layers were subjected to a washing process involving the addition of brine (20 mL), drying with Na_2_SO_4_ filtration, and subsequent removal of the solvent under reduced pressure. The crude material was purified by column chromatography.

### General Procedure E for the Synthesis of Amides

The amine was dissolved in acetone (HPLC-grade, 8 mL) and cooled to 0 °C. Thereafter, sodium bicarbonate (3 equiv.) was added to the solution. The mixture was then stirred for 10 min at the same temperature. Subsequently, the carboxylic acid chloride or anhydride (1.1 equiv. diluted in acetone by approximately 1/20) was added dropwise. The mixture was then stirred for an additional hour at 0 °C. The reaction mixture was concentrated under reduced pressure to afford the crude product. The crude material was purified by column chromatography.

### General Procedure F for the T3P-Supported Amide Synthesis

To a solution of the carboxylic acid or its sodium salt (3 equiv. each), 1-hydroxybenzotriazole hydrate (4 equiv.), and a 50 wt. % solution of 1-propanephosphonic anhydride in EtOAc (4 equiv.) in DMF (dry, 5 mL), the amine (1 equiv.) and DIPEA (5 equiv.) were added at 0 °C. The reaction mixture was diluted with EtOAc and saturated sodium bicarbonate after an overnight stirring period at ambient temperature. The organic layer was subsequently separated, and the aqueous phase was extracted with EtOAc (2 × 15 mL). The combined organic layers were dried over sodium sulfate, filtered, and concentrated under reduced pressure to afford the product. The crude material was purified by column chromatography.

#### Methyl-(3-(2,4-dichlorophenyl)­propanoyl)-l-leucinate (**1a**)

Obtained from the reaction of 3-(2,4-dichlorophenyl)­propanoic acid (220 mg, 1 mmol) and *L-*leucinemethylester • HCl (182 mg, 1 mmol) following general procedure A. Flash purification with petroleum ether/EtOAc (0−43% EtOAc). Yield: 319 mg (92%) of **1a** as a white solid. ^1^H NMR (400 MHz, CDCl_3_) δ 7.32 − 7.38 (m 1H), 7.19 (d, *J* = 8.2 Hz, 1H), 7.16 − 7.13 (m, 1H), 5.75 (d, *J* = 8.2 Hz, 1H), 4.64 − 4.57 (m, 1H), 3.71 (s, 3H), 3.04 (t, *J* = 7.5 Hz, 2H), 2.51 (t, *J* = 7.5 Hz, 2H), 1.66 − 1.60 (m, 1H), 1.51 − 1.41 (m, 2H), 0.90 (d, *J* = 6.5 Hz, 3H), 0.89 (d, *J* = 6.5 Hz, 3H). ESI-MS [M—H]^−^ = 344.3. HPLC t_*R*_ = 9.15 min.

#### (*S*)-2-(2,4-Dichlorophenoxy)-*N*-(1-hydrazineyl-4-methyl-1-oxopentan-2-yl)­acetamide (**2a**)

Obtained from **1a** (348 mg, 1 mmol) following general procedure B. Yield: 348 mg (100%) of **2a** as a white solid. ^1^H NMR (400 MHz, DMSO-*d*_*6*_) δ 9.26 (s, 1H), 8.04 (d, J = 7.1 Hz, 1H), 7.61 − 7.55 (m, 1H), 7.39 − 7.31 (m, 1H), 7.09 − 7.02 (m, 1H), 4.72 − 4.62 (m, 2H), 4.37 − 4.17 (m, 3H), 1.57 − 1.48 (m, 1H), 1.48 − 1.42 (m, 2H), 0.89 − 0.85 (m, 3H), 0.84 − 0.80 (m, 3H). ESI-MS [M + Na]^+^ = 370.0. HPLC t_*R*_ = 8.43 min.

#### (*S*)-2-(2,4-Dichlorophenoxy)-*N*-(4-methyl-1-oxo-1-(2-((2-oxo-1,2-dihydropyridin-3-yl)-methylene)­hydrazineyl)­pentan-2-yl)­acetamide (**3a**)

Obtained from the reaction of **2a** (348 mg, 1 mmol) and 2-oxo-1.2-dihydropyridine-3-carbaldehyde (123 mg, 1 mmol) following general procedure C. The crude product was obtained as an E/Z isomer mixture and used for the next step without further purification.

#### (*S*)-*N*-(1-(2-Benzylidenehydrazineyl)-4-methyl-1-oxopentan-2-yl)-2-(2,4-dichlorophenoxy)­acetamide (**3b**)

Following general procedure C, obtained from the reaction of **2a** (348 mg, 1 mmol) and benzaldehyde (101 μL, 1 mmol). The crude product was obtained as an E/Z isomer mixture and used for the next step without further purification.

#### (*S*)-2-(2,4-Dichlorophenoxy)-*N*-(1-(2-(3-fluorobenzylidene)­hydrazineyl)-4-methyl-1-oxopentan-2-yl)­acetamide (**3c**)

Obtained from the reaction of **2a** (348 mg, 1 mmol) and 3-fluorobenzaldehyde (0.11 mL, 1 mmol) following general procedure C. The crude product was obtained as an E/Z isomer mixture and used for the next step without further purification.

#### (*S*)-*N*-(1-(2-(3-Chlorobenzylidene)­hydrazineyl)-4-methyl-1-oxopentan-2-yl)-2-(2,4-dichlorophenoxy)­acetamide (**3d**)

Obtained from the reaction of **2a** (348 mg, 1 mmol) and 3-chlorobenzaldehyde (0.11 mL, 1 mmol) following general procedure C. The crude product was obtained as an E/Z isomer mixture and used for the next step without further purification.

#### (*S*)-*N*-(1-(2-(3-Chloro-2-methoxybenzylidene)­hydrazineyl)-4-methyl-1-oxopentan-2-yl)-2-(2,4-dichlorophenoxy)­acetamide (**3e**)

Obtained from the reaction of **2a** (348 mg, 1 mmol) and 3-chloro-2-methoxybenzaldehyde (171 mg, 1 mmol) following general procedure C. The crude product was obtained as an E/Z isomer mixture and used for the next step without further purification.

#### (*S*)-2-(2,4-Dichlorophenoxy)-*N*-(4-methyl-1-oxo-1-(2-(2-(trifluoromethoxy)-benzylidene)­hydrazineyl)­pentan-2-yl)­acetamide (**3f**)

Obtained from the reaction of **2a** (348 mg, 1 mmol) and 2-(trifluoromethoxy)­benzaldehyde (0.14 mL, 1 mmol) following general procedure C. The crude product was obtained as an E/Z isomer mixture and used for the next step without further purification.

#### (*S*)-2-(2,4-Dichlorophenoxy)-*N*-(4-methyl-1-oxo-1-(2-(pyridin-3-yl-methylene)­hydrazineyl)­pentan-2-yl)­acetamide (**3g**)

Following general procedure C, obtained from the reaction of **2a** (348 mg, 1 mmol) and 3-pyridinecarboxaldehyde (90 μL, 1 mmol). The crude product was obtained as an E/Z isomer mixture and used for the next step without further purification.

#### (*S*)-2-(2,4-Dichlorophenoxy)-*N*-(4-methyl-1-oxo-1-(2-(pyridin-4-yl-methylene)­hydrazineyl)­pentan-2-yl)­acetamide (**3h**)

Following general procedure C, obtained from the reaction of **2a** (348 mg, 1 mmol) and 4-pyridinecarboxaldehyde (90 μL, 1 mmol). The crude product was obtained as an E/Z isomer mixture and used for the next step without further purification.

#### (*S*)-2-(2,4-Dichlorophenoxy)-*N*-(4-methyl-1-(2-((1-methyl-1*H*-1,2,3-triazol-4-yl)-methylene)­hydrazineyl)-1-oxopentan-2-yl)­acetamide (**3i**)

Following general procedure C, obtained from the reaction of **2a** (348 mg, 1 mmol) and 1-methyl-1H−1,2,3-triazole-4-carbaldehyde (111 mg, 1 mmol). The crude product was obtained as an E/Z isomer mixture and used for the next step without further purification.

#### (*S*)-2-(2,4-Dichlorophenoxy)-*N*-(4-methyl-1-oxo-1-(2-((2-oxo-1,2-dihydroquinolin-3-yl)-methylene)­hydrazineyl)­pentan-2-yl)­acetamide (**3j**)

Following general procedure C, obtained from the reaction of **2a** (348 mg, 1 mmol) and 2-oxo-1,2-dihydroquinoline-3-carbaldehyde (173 mg, 1 mmol). The crude product was obtained as an E/Z isomer mixture and used for the next step without further purification.

#### (*S*)-*N*-(1-(2-((5-Chloro-2-oxo-1,2-dihydropyridin-3-yl)­methylene)­hydrazineyl)-4-methyl-1-oxopentan-2-yl)-2-(2,4-dichlorophenoxy)­acetamide (**3k**)

Obtained from the reaction of **2a** (348 mg, 1 mmol) and 5-chloro-2-oxo-1,2-dihydropyridine-3-carbaldehyde (158 mg, 1 mmol) following General Procedure C. The crude product was obtained as an E/Z isomer mixture and used for the next step without further purification.

#### (*S*)-2-(2,4-Dichlorophenoxy)-*N*-(1-(2-((2,4-dioxo-1,2,3,4-tetrahydropyrimidin-5-yl)-methylene)­hydrazineyl)-4-methyl-1-oxopentan-2-yl)­acetamide (**3l**)

Following general procedure C, obtained from the reaction of 2a (348 mg, 1 mmol) and 2,4-dioxo-1,2,3,4-tetrahydropyrimidine-5-carbaldehyde (140 mg, 1 mmol). The crude product was obtained as an E/Z isomer mixture and used for the next step without further purification.

#### (*S*)-2-(2,4-Dichlorophenoxy)-*N*-(4-methyl-1-oxo-1-(2-((2-oxo-1,2-dihydropyridin-3-yl)-methyl)­hydrazineyl)­pentan-2-yl)­acetamide (**4a**)

Obtained from **3a** (453 mg, 1 mmol) following general procedure D. Flash purification with CH_2_Cl_2_/CH_3_OH (0 − 7.5% CH_3_OH). Yield over 2 steps: 246 mg (54%) of **4a** as a white solid. ^1^H NMR (400 MHz, DMSO-*d*_*6*_) δ 11.54 (s, 1H), 9.59 (s, 1H), 8.03 (d, *J* = 8.4 Hz, 1H), 7.59 (d, *J* = 2.5 Hz, 1H), 7.39 − 7.31 (m, 2H), 7.28 − 7.23 (m, 1H), 7.04 (d, *J* = 8.9 Hz, 1H), 6.14 − 6.07 (m, 1H), 5.40 − 5.27 (m, 1H), 4.72 − 4.61 (m, 2H), 4.32 − 4.22 (m, 1H), 3.67 − 3.55 (m, 2H), 1.48 − 1.36 (m, 3H), 0.84 (d, *J* = 6.3 Hz, 3H), 0.80 (d, *J* = 6.2 Hz, 3H). ESI-MS [M + Na]^+^ = 477.1. HPLC t_*R*_ = 7.98 min.

#### (*S*)-*N*-(1-(2-Benzylhydrazineyl)-4-methyl-1-oxopentan-2-yl)-2-(2,4-dichlorophenoxy)­acetamide (**4b**)

Obtained from **3b** (438 mg, 1 mmol) following general procedure D. Flash purification with CH_2_Cl_2_/CH_3_OH (0 − 2.5% CH_3_OH). Yield over 2 steps: 321 mg (73%) of **4b** as a white solid. ^1^H NMR (400 MHz, DMSO-*d*_*6*_) δ 9.56 (d, *J* = 6.2 Hz, 1H), 8.03 (d, *J* = 8.4 Hz, 1H), 7.59 (d, *J* = 2.6 Hz, 1H), 7.36 − 7.33 (m, 1H), 7.32 − 7.31 (m, 2H), 7.31 − 7.29 (m, 2H), 7.27 − 7.21 (m, 1H), 7.04 (d, *J* = 9.0 Hz, 1H), 5.27 − 5.21 (m, 1H), 4.71 − 4.63 (m, 2H), 4.32 − 4.25 (m, 1H), 3.84 (d, *J* = 5.2 Hz, 2H), 1.48 − 1.36 (m, 3H), 0.82 (d, *J* = 6.3 Hz, 3H), 0.79 (d, *J* = 6.3 Hz, 3H). ESI-MS [M + Na]^+^ = 460.1. HPLC t_*R*_ = 9.96 min.

#### (*S*)-2-(2,4-Dichlorophenoxy)-*N*-(1-(2-(3-fluorobenzyl)­hydrazineyl)-4-methyl-1-oxopentan-2-yl)­acetamide (**4c**)

Obtained from **3c** (494 mg, 1 mmol) following general procedure D. Flash purification with CH_2_Cl_2_/CH_3_OH (0 − 1.5% CH_3_OH). Yield over 2 steps: 246 mg (54%) of **4c** as a white solid. ^1^H NMR (400 MHz, DMSO-*d*_*6*_) δ 9.56 (d, *J* = 6.0 Hz, 1H), 8.04 (d, *J* = 8.4 Hz, 1H), 7.59 (d, *J* = 2.6 Hz, 1H), 7.38 − 7.30 (m, 2H), 7.18 − 7.10 (m, 2H), 7.09 − 7.00 (m, 2H), 5.44 − 5.36 (m, 1H), 4.72 − 4.60 (m, 2H), 4.33 − 4.21 (m, 1H), 3.93 − 3.81 (m, 2H), 1.46 − 1.31 (m, 3H), 0.80 (d, *J* = 6.2 Hz, 3H), 0.78 (d, *J* = 6.2 Hz, 3H). ESI-MS [M + Na]^+^ = 478.2. HPLC t_*R*_ = 9.56 min.

#### (*S*)-*N*-(1-(2-(3-Chlorobenzyl)­hydrazineyl)-4-methyl-1-oxopentan-2-yl)-2-(2,4-dichlorophenoxy)­acetamide (**4d**)

Obtained from **3d** (470 mg, 1 mmol) following general procedure D. Flash purification with CH_2_Cl_2_/CH_3_OH (0 − 1.5% CH_3_OH). Yield over 2 steps: 387 mg (81%) of **4d** as a white solid. ^1^H NMR (400 MHz, DMSO-*d*_*6*_) δ 9.55 (d, *J* = 6.0 Hz, 1H), 8.04 (d, *J* = 8.4 Hz, 1H), 7.59 (d, *J* = 2.6 Hz, 1H), 7.39 − 7.36 (m, 1H), 7.35 − 7.32 (m, 1H), 7.31 − 7.29 (m, 2H), 7.27 − 7.23 (m, 1H), 7.03 (d, *J* = 8.9 Hz, 1H), 5.45 − 5.39 (m, 1H), 4.69 − 4.63 (m, 2H), 4.30 − 4.23 (m, 1H), 3.88 − 3.83 (m, 2H), 1.42 − 1.31 (m, 3H), 0.81 (d, *J* = 6.1 Hz, 3H), 0.78 (d, *J* = 6.2 Hz, 3H). ESI-MS [M + Na]^+^ = 494.2. HPLC t_*R*_ = 10.44 min.

#### (*S*)-*N*-(1-(2-(3-Chloro-2-methoxybenzyl)­hydrazineyl)-4-methyl-1-oxopentan-2-yl)-2-(2,4-dichlorophenoxy)­acetamide (**4e**)

Obtained from **3e** (500 mg, 1 mmol) following general procedure D. Flash purification with CH_2_Cl_2_/CH_3_OH (0 − 7% CH_3_OH). The crude light-yellow solid was used for the next step. ESI-MS [M + Na]^+^ = 523.8. HPLC t_*R*_ = 10.04 min.

#### (*S*)-2-(2,4-Dichlorophenoxy)-*N*-(4-methyl-1-oxo-1-(2-(2-(trifluoromethoxy)­benzyl)­hydrazineyl)­pentan-2-yl)­acetamide (**4f**)

Obtained from **3f** (520 mg, 1 mmol) following general procedure D. Flash purification with CH_2_Cl_2_/CH_3_OH (0 − 3% CH_3_OH). Yield over 2 steps: 110 mg (21%) of **4f** as an off white solid. ^1^H NMR (400 MHz, DMSO-*d*_*6*_) δ 9.66 − 9.56 (m, 1H), 8.06 (d, *J* = 8.3 Hz, 1H), 7.66 − 7.56 (m, 2H), 7.45 − 7.37 (m, 2H), 7.37 − 7.31 (m, 2H), 7.06 (d, *J* = 8.9 Hz, 1H), 5.47 − 5.33 (m, 1H), 4.74 − 4.59 (m, 2H), 4.34 − 4.25 (m, 1H), 4.01 − 3.88 (m, 2H), 1.51 − 1.36 (m, 3H), 0.83 (d, *J* = 6.2 Hz, 3H), 0.80 (d, *J* = 6.2 Hz, 3H). ESI-MS [M + Na]^+^ = 543.9. HPLC t_*R*_ = 10.21 min.

#### (*S*)-2-(2,4-Dichlorophenoxy)-*N*-(4-methyl-1-oxo-1-(2-(pyridin-3-yl-methyl)­hydrazineyl)­pentan-2-yl)­acetamide (**4g**)

Obtained from **3g** (441 mg, 1 mmol) following general procedure D. Flash purification with CH_2_Cl_2_/CH_3_OH (0 − 4% CH_3_OH). Yield over 2 steps: 294 mg (67%) of **4g** as a white solid. ^1^H NMR (400 MHz, DMSO-*d*_*6*_) δ 9.56 (s, 1H), 8.53 − 8.43 (m, 2H), 8.08 − 8.00 (m, 1H), 7.74 − 7.68 (m, H), 7.59 (d, *J* = 2.6 Hz, 1H), 7.38 − 7.28 (m, 2H), 7.04 (d, *J* = 8.9 Hz, 1H), 5.53 − 5.37 (m, 1H), 4.73 − 4.61 (m, 2H), 4.31 − 4.22 (m, 1H), 3.87 (s, 2H), 1.47 − 1.31 (m, 3H), 0.81 (d, *J* = 6.2 Hz, 3H), 0.78 (d, *J* = 6.2 Hz, 3H). ESI-MS [M + Na]^+^ = 461.2. HPLC t_*R*_ = 7.60 min.

#### (*S*)-2-(2,4-Dichlorophenoxy)-*N*-(4-methyl-1-oxo-1-(2-(pyridin-4-yl-methylydrazineyl)­pentan-2-yl)­acetamide (**4h**)

Obtained from **3h** (441 mg, 1 mmol) following general procedure D. Flash purification with CH_2_Cl_2_/CH_3_OH (0 − 4% CH_3_OH). Yield over 2 steps: 355 mg (81%) of **4h** as a white solid. ^1^H NMR (400 MHz, DMSO-*d*_*6*_) δ 9.57 (d, *J* = 5.8 Hz, 1H), 8.54 − 8.44 (m, 2H), 8.05 (d, *J* = 8.4 Hz, 1H), 7.59 (d, *J* = 2.6 Hz, 1H), 7.36 − 7.30 (m, 3H), 7.03 (d, *J* = 8.9 Hz, 1H), 5.57 − 5.47 (m, 1H), 4.72 − 4.61 (m, 2H), 4.31 − 4.21 (m, 1H), 3.93 − 3.84 (m, 2H), 1.46 − 1.31 (m, 3H), 0.80 (d, *J* = 6.1 Hz, 3H), 0.77 (d, *J* = 6.1 Hz, 3H). ESI-MS [M + Na]^+^ = 461.4. HPLC t_*R*_ = 6.78 min.

#### (*S*)-2-(2,4-Dichlorophenoxy)-*N*-(4-methyl-1-(2-((1-methyl-1*H*-1,2,3-triazol-4-yl) methyl)­hydrazineyl)-1-oxopentan-2-yl)­acetamide (**4i**)

Obtained from **3i** (441 mg, 1 mmol) following general procedure D. Flash purification with CH_2_Cl_2_/CH_3_OH (0 − 7% CH_3_OH). Yield over 2 steps: 222 mg (50%) of **4i** as a white solid. ^1^H NMR (400 MHz, DMSO-*d*_*6*_) δ 9.58 (d, *J* = 6.3 Hz, 1H), 8.08 (d, *J* = 8.3 Hz, 1H), 7.91 − 7.83 (m, 1H), 7.59 (d, *J* = 2.6 Hz, 1H), 7.35 (dd, *J* = 8.9, 2.6 Hz, 1H), 7.04 (d, *J* = 8.9 Hz, 1H), 5.28 − 5.17 (m, 1H), 4.74 − 4.61 (m, 2H), 4.34 − 4.21 (m, 1H), 4.01 − 3.97 (m, 3H), 3.89 (d, *J* = 5.4 Hz, 2H), 1.53 − 1.38 (m, 3H), 0.85 (d, *J* = 6.3 Hz, 3H), 0.81 (d, *J* = 6.3 Hz, 3H). ESI-MS [M—H]^−^ = 441.3. HPLC t_*R*_ = 8.50 min.

#### (*S*)-2-(2,4-Dichlorophenoxy)-*N*-(4-methyl-1-oxo-1-(2-((2-oxo-1,2-dihydroquinolin-3-yl)­methyl)­hydrazineyl)­pentan-2-yl)­acetamide (**4j**)

Obtained from **3j** (503 mg, 1 mmol) following general procedure D. Flash purification with CH_2_Cl_2_/CH_3_OH (0 − 7.5% CH_3_OH). Yield over 2 steps: 233 mg (46%) of **4j** as a white solid. ^1^H NMR (400 MHz, DMSO-*d*_*6*_) δ 11.76 (s, 1H), 9.70 − 9.56 (m, 1H), 8.03 (d, *J* = 8.4 Hz, 1H), 7.76 (s, 1H), 7.61 − 7.55 (m, 2H), 7.47 − 7.41 (m, 1H), 7.36 − 7.31 (m, 1H), 7.29 (d, *J* = 8.2 Hz, 1H), 7.18 − 7.11 (m, 1H), 7.01 (d, *J* = 8.9 Hz, 1H), 5.48 − 5.37 (m, 1H), 4.70 − 4.56 (m, 2H), 4.33 − 4.20 (m, 1H), 3.79 (dd, *J* = 14.7, 4.2 Hz, 1H), 3.68 (dd, *J* = 14.8, 6.0 Hz, 1H), 1.47 − 1.28 (m, 3H), 0.77 (d, *J* = 6.3 Hz, 3H), 0.72 (d, *J* = 6.2 Hz, 3H). ESI-MS [M—H]^−^ = 502.9. HPLC t_*R*_ = 8.84 min.

#### (*S*)-*N*-(1-(2-((5-Chloro-2-oxo-1,2-dihydropyridin-3-yl)­methyl)­hydrazineyl)-4-methyl-1-oxopentan-2-yl)-2-(2,4-dichlorophenoxy)­acetamide (**4k**)

Obtained from **3k** (487 mg, 1 mmol) following general procedure D. Flash purification with CH_2_Cl_2_/CH_3_OH (0 − 7.5% CH_3_OH). Yield over 2 steps: 98 mg (20%) of **4k** as a white solid. ^1^H NMR (400 MHz, DMSO-*d*_*6*_) δ 11.91 − 11.43 (m, 1H), 9.69 − 9.55 (m, 1H), 8.05 (d, *J* = 8.4 Hz, 1H), 7.59 (d, *J* = 2.6 Hz, 1H), 7.49 (d, *J* = 2.8 Hz, 1H), 7.40 (d, *J* = 2.8 Hz, 1H), 7.37 − 7.30 (m, 1H), 7.04 (d, *J* = 8.9 Hz, 1H), 5.47 − 5.33 (m, 1H), 4.75 − 4.59 (m, 2H), 4.34 − 4.20 (m, 1H), 3.73 − 3.54 (m, 2H), 1.52 − 1.34 (m, 3H), 0.85 (d, *J* = 6.3 Hz, 3H), 0.81 (d, *J* = 6.3 Hz, 3H). ESI-MS [M—H]^−^ = 486.7. HPLC t_*R*_ = 8.60 min.

#### (*S*)-2-(2,4-Dichlorophenoxy)-*N*-(1-(2-((2,4-dioxo-1,2,3,4-tetrahydropyrimidin-5-yl)­methyl)­hydrazineyl)-4-methyl-1-oxopentan-2-yl)­acetamide (**4l**)

Obtained from **3l** (470 mg, 1 mmol) following general procedure D. Flash purification with CH_2_Cl_2_/CH_3_OH (0 − 9% CH_3_OH). Yield over 2 steps: 217 mg (46%) of **4l** as a white solid. ^1^H NMR (400 MHz, DMSO-*d*_*6*_) δ 11.11 − 10.98 (m, 1H), 10.81 − 10.68 (m, 1H), 9.61 − 9.45 (m, 1H), 8.06 (d, *J* = 8.3 Hz, 1H), 7.59 (d, *J* = 2.6 Hz, 1H), 7.40 − 7.31 (m, 1H), 7.29 − 7.18 (m, 1H), 7.04 (d, *J* = 8.9 Hz, 1H), 5.20 − 5.05 (m, 1H), 4.75 − 4.62 (m, 2H), 4.33 − 4.23 (m, 1H), 3.52 − 3.36 (m, 2H), 1.53 − 1.37 (m, 3H), 0.87 (d, *J* = 6.3 Hz, 3H), 0.82 (d, *J* = 6.3 Hz, 3H). ESI-MS [M + Na]^+^ = 494.1. HPLC t_*R*_ = 8.14 min.

#### (*S*)-2-(2,4-Dichlorophenoxy)-*N*-(1-(2-(2-hydroxyacetyl)-2-((2-oxo-1,2-dihydropyridin-3-yl)­methyl)­hydrazineyl)-4-methyl-1-oxopentan-2-yl)­acetamide (**5a**)

Following general procedure F for the synthesis of amides, compound **5a** was synthesized using **4a** (100 mg, 0.22 mmol, 1 equiv.), 2-Hydroxyacetic acid (50 mg, 0.66 mmol, 3 equiv.), T_3_P (50% in EtOAc, 0.52 mL, 0.88 mmol, 4 equiv.), 1-hydroxybenzotriazole hydrate (135 mg, 0.88 mmol, 4 equiv.), and DIPEA (0.19 mL, 1.10 mmol, 5 equiv.) in DMF (5 mL). After column chromatography (NP, CH_2_Cl_2_/CH_3_OH, v/v, 100:0−92:8), the pure product was isolated as a white solid (65 mg, 58%). ^1^H NMR (400 MHz, DMSO-*d*_*6*_) δ 11.70 (s, 1H), 10.69 − 10.31 (m, 1H), 8.41 − 8.10 (m, 1H), 7.68 − 7.48 (m, 1H), 7.45 − 7.20 (m, 3H), 7.16 − 6.94 (m, 1H), 6.24 − 6.00 (m, 1H), 4.90 − 4.71 (m, 2H), 4.74 − 4.63 (m, 2H), 4.44 − 4.16 (m, 2H), 4.11 − 3.84 (m, 1H), 1.90 − 1.71 (m, 1H), 1.57 − 1.47 (m, 2H), 1.47 − 1.30 (m, 1H), 0.95 − 0.84 (m, 3H), 0.82 − 0.76 (m, 3H). ^13^C NMR (101 MHz, DMSO-*d*_*6*_) δ 171.48, 168.70, 167.20, 161.89, 152.49, 134.41, 129.30, 128.13, 127.90, 125.49, 125.02, 122.40, 115.27, 104.64, 67.42, 61.87, 57.63, 49.75, 46.87, 24.14, 22.79, 21.34. TLC-MS (ESI) *m*/*z* for (C_22_H_26_Cl_2_N_4_O_6_ [M—H]^−^) calcd 511.12, found 511.0. HPLC t_*R*_ = 10.30 min.

#### (*S*)-*N*-(1-(2-(2-Cyanoacetyl)-2-((2-oxo-1,2-dihydropyridin-3-yl)­methyl)­hydrazineyl)-4-methyl-1-oxopentan-2-yl)-2-(2,4-dichlorophenoxy)­acetamide (**5b**)

Following general procedure F for the synthesis of amides, compound **5b** was synthesized using **4a** (100 mg, 0.22 mmol, 1 equiv.), 2-cyanoacetic acid (56 mg, 0.66 mmol, 3 equiv.), T_3_P (50% in EtOAc, 0.52 mL, 0.88 mmol, 4 equiv.), 1-hydroxybenzotriazole hydrate (135 mg, 0.88 mmol, 4 equiv.), and DIPEA (0.19 mL, 1.10 mmol, 5 equiv.) in DMF (5 mL). After column chromatography (NP, CH_2_Cl_2_/CH_3_OH, v/v, 100:0−93.4:6.6), the pure product was isolated as an off-white solid (75 mg, 65%). ^1^H NMR (400 MHz, DMSO-*d*_*6*_) δ 11.73 (s, 1H), 10.80 − 10.42 (m, 1H), 8.44 − 8.19 (m, 1H), 7.58 (d, *J* = 2.6 Hz, 1H), 7.40 − 7.28 (m, 3H), 7.08 − 6.97 (m, 1H), 6.16 (t, *J* = 6.6 Hz, 1H), 4.77 − 4.68 (m, 2H), 4.68 − 4.61 (m, 1H), 4.29 − 4.10 (m, 1H), 4.06 − 3.79 (m, 2H), 3.65 − 3.49 (m, 1H), 1.60 − 1.44 (m, 2H), 1.45 − 1.30 (m, 1H), 0.87 (d, *J* = 6.6 Hz, 3H), 0.82 (d, *J* = 6.3 Hz, 3H). ^13^C NMR (101 MHz, DMSO-*d*_*6*_) δ 171.50, 167.52, 165.07, 161.90, 152.50, 139.55, 134.66, 129.36, 127.89, 125.27, 125.05, 122.39, 115.50, 115.24, 104.61, 67.31, 58.46, 49.86, 47.19, 30.70, 24.12, 24.06, 22.84. TLC-MS (ESI) *m*/*z* for (C_23_H_25_Cl_2_N_5_O_5_ [M—H]^−^) calcd 520.12, found 519.9. HPLC t_*R*_ = 8.29 min.

#### (*S*)-2-(2,4-Dichlorophenoxy)-*N*-(4-methyl-1-oxo-1-(2-((2-oxo-1,2-dihydropyridin-3-yl)­methyl)-2-(2-oxopropanoyl)­hydrazineyl)­pentan-2-yl)­acetamide (**5c**)

Following general procedure F for the synthesis of amides, compound **5c** was synthesized using **4a** (100 mg, 0.22 mmol, 1 equiv.), sodium 2-oxopropanoate (73 mg, 0.66 mmol, 3 equiv.), T_3_P (50% in EtOAc, 0.52 mL, 0.88 mmol, 4 equiv.), 1-hydroxybenzotriazole hydrate (135 mg, 0.88 mmol, 4 equiv.), and DIPEA (0.19 mL, 1.10 mmol, 5 equiv.) in DMF (5 mL). After column chromatography (NP, CH_2_Cl_2_/CH_3_OH, v/v, 100:0−95.2:4.8), the pure product was isolated as a white solid (82 mg, 71%). ^1^H NMR (400 MHz, DMSO-*d*_*6*_) δ 11.75 (s, 1H), 10.82 − 10.34 (m, 1H), 8.27 − 8.10 (m, 1H), 7.61 − 7.56 (m, 1H), 7.37 − 7.27 (m, 3H), 7.05 − 6.98 (m, 1H), 6.19 − 6.09 (m, 1H), 4.78 − 4.57 (m, 3H), 4.47 − 4.18 (m, 2H), 2.17 (s, 3H), 1.53 − 1.40 (m, 2H), 1.35 − 1.25 (m, 1H), 0.84 (d, *J* = 6.4 Hz, 3H), 0.78 (d, *J* = 6.4 Hz, 3H). ^13^C NMR (101 MHz, DMSO-*d*_*6*_) δ 198.86, 172.68, 168.98, 167.46, 162.27, 152.95, 140.30, 135.38, 129.80, 128.37, 125.47, 125.08, 122.86, 115.70, 105.09, 67.77, 49.88, 46.53, 40.33, 27.49, 26.80, 24.54, 23.32. TLC-MS (ESI) *m*/*z* for (C_23_H_26_Cl_2_N_4_O_6_ [M + Na]^−^) calcd 547.12, found 546.9. HPLC t_*R*_ = 8.29 min.

#### (*S*)-2-(2,4-Dichlorophenoxy)-*N*-(4-methyl-1-oxo-1-(2-((2-oxo-1,2-dihydropyridin-3-yl)­methyl)-2-(2-oxo-2-phenylacetyl)­hydrazineyl)­pentan-2-yl)­acetamide (**5d**)

Following general procedure F for the synthesis of amides, compound **5d** was synthesized using **4a** (100 mg, 0.22 mmol, 1 equiv.), 2-oxo-2-phenylacetic acid (99 mg, 0.66 mmol, 3 equiv.), T_3_P (50% in EtOAc, 0.52 mL, 0.88 mmol, 4 equiv.), 1-hydroxybenzotriazole hydrate (135 mg, 0.88 mmol, 4 equiv.), and DIPEA (0.19 mL, 1.10 mmol, 5 equiv.) in DMF (5 mL). After column chromatography (NP, CH_2_Cl_2_/CH_3_OH, v/v, 100:0−94.8:5.2), the pure product was isolated as a white solid (112 mg, 87%). ^1^H NMR (400 MHz, DMSO-*d*_*6*_) δ 11.76 (s, 1H), 10.70 (s, 1H), 8.18 − 7.96 (m, 1H), 7.87 (d, *J* = 7.3 Hz, 2H), 7.78 − 7.66 (m, 1H), 7.60 − 7.51 (m, 3H), 7.41 − 7.36 (m, 1H), 7.36 − 7.26 (m, 2H), 6.97 (d, *J* = 8.8 Hz, 1H), 6.21 − 6.13 (m, 1H), 5.16 − 4.95 (m, 1H), 4.67 − 4.46 (m, 2H), 4.15 − 4.08 (m, 1H), 4.06 − 3.92 (m, 1H), 1.34 − 1.19 (m, 1H), 1.00 − 0.79 (m, 2H), 0.74 − 0.54 (m, 6H). ^13^C NMR (101 MHz, DMSO-*d*_*6*_) δ 193.70, 167.71, 166.70, 166.60, 161.81, 152.42, 134.93, 134.82, 131.87, 129.28 (2C), 129.26 (2C), 128.92, 128.86, 127.86, 125.00, 124.53, 122.40, 115.26, 104.63, 67.28, 49.74, 45.67, 40.07, 23.80, 22.60, 21.32. TLC-MS (ESI) *m*/*z* for (C_28_H_28_Cl_2_N_4_O_6_ [M—H]^−^) calcd 585.14, found 585.1. HPLC t_*R*_ = 9.16 min.

#### (*S*)-*N*-(1-(2-(2,2-Dichloroacetyl)-2-((2-oxo-1,2-dihydropyridin-3-yl)­methyl)­hydrazineyl)-4-methyl-1-oxopentan-2-yl)-2-(2,4-dichlorophenoxy)­acetamide (**5e**)

Following general procedure F for the synthesis of amides, compound **5e** was synthesized using **4a** (100 mg, 0.22 mmol, 1 equiv.), 2,2-dichloroacetic acid (54 mg, 0.66 mmol, 3 equiv.), T_3_P (50% in EtOAc, 0.52 mL, 0.88 mmol, 4 equiv.), 1-hydroxybenzotriazole hydrate (135 mg, 0.88 mmol, 4 equiv.), and DIPEA (0.19 mL, 1.10 mmol, 5 equiv.) in DMF (5 mL). After column chromatography (NP, CH_2_Cl_2_/CH_3_OH, v/v, 100:0−93:7), the pure product was isolated as a white solid (94 mg, 76%). ^1^H NMR (400 MHz, DMSO-*d*_*6*_) δ 11.76 (s, 1H), 10.92 − 10.55 (m, 1H), 8.45 − 8.24 (m, 1H), 7.58 (d, *J* = 2.6 Hz, 1H), 7.39 − 7.34 (m, 1H), 7.34 − 7.28 (m, 2H), 7.12 − 6.94 (m, 1H), 6.76 − 6.58 (m, 1H), 6.17 (t, *J* = 6.6 Hz, 1H), 4.69 (m, 2H), 4.69 − 4.62 (m, 1H), 4.31 − 4.15 (m, 1H), 4.10 − 3.96 (m, 1H), 1.59 − 1.46 (m, 2H), 1.46 − 1.31 (m, 1H), 0.91 (d, *J* = 6.3 Hz, 3H), 0.82 (d, *J* = 6.3 Hz, 3H). ^13^C NMR (101 MHz, DMSO-*d*_*6*_) δ 171.74, 167.73, 165.36, 161.89, 152.52, 139.64, 134.88, 129.32, 127.93, 125.03, 124.83, 122.41, 115.34, 104.65, 67.41, 64.32, 50.00, 47.79, 39.84, 24.12, 22.90, 21.37. TLC-MS (ESI) *m*/*z* for (C_22_H_24_Cl_4_N_4_O_5_ [M + H]^+^) calcd 565.05, found 564.8. HPLC t_*R*_ = 8.57 min.

#### *N*-((2*S*)-1-(2-(2-Chloro-2-fluoroacetyl)-2-((2-oxo-1,2-dihydropyridin-3-yl)­methyl)­hydrazineyl)-4-methyl-1-oxopentan-2-yl)-2-(2,4-dichlorophenoxy)­acetamide­(**5f**)

Following general procedure F for the synthesis of amides, compound **5f** was synthesized using **4a** (100 mg, 0.22 mmol, 1 equiv.), sodium 2-chloro-2-fluoroacetate (89 mg, 0.66 mmol, 3 equiv.), T_3_P (50% in EtOAc, 0.52 mL, 0.88 mmol, 4 equiv.), 1-hydroxybenzotriazole hydrate (135 mg, 0.88 mmol, 4 equiv.), and DIPEA (0.19 mL, 1.10 mmol, 5 equiv.) in DMF (5 mL). After column chromatography (NP, CH_2_Cl_2_/CH_3_OH, v/v, 100:0−94:6), the pure product was isolated as a white solid (28 mg, 23%). ^1^H NMR (400 MHz, DMSO-*d*_*6*_) δ 11.75 (s, 1H), 10.96 − 10.39 (m, 1H), 8.47 − 8.19 (m, 1H), 7.64 − 7.53 (m, 1H), 7.43 − 7.26 (m, 3H), 7.14 − 6.91 (m, 1H), 6.88 − 6.51 (m, 1H), 6.25 − 6.10 (m, 1H), 4.88 − 4.74 (m, 1H), 4.75 − 4.63 (m, 2H), 4.34 − 3.93 (m, 2H), 1.63 − 1.46 (m, 2H), 1.45 − 1.28 (m, 1H), 0.86 (d, *J* = 6.1 Hz, 3H), 0.81 (d, *J* = 6.1 Hz, 3H). ^13^C NMR (101 MHz, DMSO-*d*_*6*_) δ 172.86, 170.81, 162.38, 162.32, 152.96, 140.11, 135.41, 129.80, 128.41, 128.35, 125.51, 122.89, 117.01 (d, *J* = 246.8 Hz), 115.75, 105.09, 67.85, 50.54, 47.58, 39.44, 24.59, 23.44, 21.74. TLC-MS (ESI) *m*/*z* for (C_22_H_24_Cl_3_FN_4_O_5_ [M—H]^−^) calcd 547.08, found 546.8. HPLC t_*R*_ = 8.38 min.

#### (*S*)-*N*-(1-(2-(But-2-ynoyl)-2-((2-oxo-1,2-dihydropyridin-3-yl)­methyl)­hydrazineyl)-4-methyl-1-oxopentan-2-yl)-2-(2,4-dichlorophenoxy)­acetamide (**5g**)

Following general procedure F for the synthesis of amides, compound **5g** was synthesized using **4a** (100 mg, 0.22 mmol, 1 equiv.) but-2-ynoic acid (55 mg, 0.66 mmol, 3 equiv.), T_3_P (50% in EtOAc, 0.52 mL, 0.88 mmol, 4 equiv.), 1-hydroxybenzotriazole hydrate (135 mg, 0.88 mmol, 4 equiv.), and DIPEA (0.19 mL, 1.10 mmol, 5 equiv.) in DMF (5 mL). After column chromatography (NP, CH_2_Cl_2_/CH_3_OH, v/v, 100:0−92:8), the pure product was isolated as an off-white solid (70 mg, 61%). ^1^H NMR (400 MHz, DMSO-*d*_*6*_) δ 11.68 (s, 1H), 10.83 − 10.71 (m, 1H), 8.23 (d, *J* = 8.2 Hz, 1H), 7.58 (d, *J* = 2.6 Hz, 1H), 7.34 − 7.30 (m, 2H), 7.29 − 7.23 (m, 1H), 7.07 − 7.00 (m, 1H), 6.19 − 6.12 (m, 1H), 4.74 − 4.66 (m, 2H), 4.67 − 4.56 (m, 1H), 4.55 − 4.25 (m, 2H), 4.55 − 4.25 (m, 2H), 1.90 (s, 3H), 1.64 − 1.53 (m, 1H), 1.52 − 1.43 (m, 2H), 0.87 (d, *J* = 6.4 Hz, 3H), 0.83 (d, *J* = 6.4 Hz, 3H). ^13^C NMR (101 MHz, DMSO-*d*_*6*_) δ 171.70, 167.23, 162.17, 155.89, 152.95, 139.23, 134.88, 129.79, 128.37, 125.88, 125.48, 122.87, 115.74, 105.03, 89.17, 73.50, 67.86, 49.77, 47.03, 41.11, 24.59, 23.40, 21.90, 3.76. TLC-MS (ESI) *m*/*z* for (C_24_H_26_Cl_2_N_4_O_5_ [M—H]^−^) calcd 519.13, found 519.1. HPLC t_*R*_ = 8.13 min.

#### Methyl-(*E*)-4-(2-((2-(2,4-dichlorophenoxy)­acetyl)-l-leucyl)-1-((2-oxo-1,2-dihydropyridin-3-yl)­methyl)­hydrazineyl)-4-oxobut-2-enoate (**5h**)

Following general procedure F for the synthesis of amides, compound **5h** was synthesized using **4a** (100 mg, 0.22 mmol, 1 equiv.) (*E*)-4-methoxy-4-oxobut-2-enoic acid (86 mg, 0.66 mmol, 3 equiv.), T_3_P (50% in EtOAc, 0.52 mL, 0.88 mmol, 4 equiv.), 1-hydroxybenzotriazole hydrate (135 mg, 0.88 mmol, 4 equiv.), and DIPEA (0.19 mL, 1.10 mmol, 5 equiv.) in DMF (5 mL). After column chromatography (NP, CH_2_Cl_2_/CH_3_OH, v/v, 100:0−93.5:6.5), the pure product was isolated as a white solid (52 mg, 42%). ^1^H NMR (400 MHz, DMSO-*d*_*6*_) δ 11.72 (s, 1H), 10.85 (s, 1H), 8.31 (d, *J* = 7.7 Hz, 1H), 7.58 (d, *J* = 2.6 Hz, 1H), 7.37 − 7.26 (m, 3H), 7.19 (d, *J* = 18.0 Hz, 1H), 7.02 (d, *J* = 8.2 Hz, 1H), 6.62 (d, *J* = 17.8 Hz, 1H), 6.19 − 6.11 (m, 1H), 4.98 − 4.78 (m, 1H), 4.75 − 4.61 (m, 2H), 4.42 − 4.27 (m, 1H), 4.05 − 3.88 (m, 1H), 3.70 (s, 3H), 1.60 − 1.50 (m, 1H), 1.50 − 1.41 (m, 2H), 0.94 − 0.85 (m, 3H), 0.84 − 0.78 (m, 3H). ^13^C NMR (101 MHz, DMSO-*d*_*6*_) δ 171.38, 166.99, 165.38, 165.12, 161.92, 152.51, 134.53, 134.52, 133.20, 129.35, 129.30, 127.87, 125.41, 125.01, 122.41, 115.28, 104.64, 67.38, 52.01, 49.53, 47.18, 38.97, 24.18, 22.64, 21.55. TLC-MS (ESI) *m*/*z* for (C_25_H_28_Cl_2_N_4_O_7_ [M—H]^−^) calcd 565.13, found 565.3. HPLC t_*R*_ = 8.37 min.

#### (*S*)-*N*-(1-(2-Acryloyl-2-((2-oxo-1,2-dihydropyridin-3-yl)­methyl)­hydrazineyl)-4-methyl-1-oxopentan-2-yl)-2-(2,4-dichlorophenoxy)­acetamide (**6a**)

Following the general procedure E for the synthesis of amides, compound **6a** was synthesized using **4a** (100 mg, 0.22 mmol, 1 equiv.), acryloyl chloride (20 μL, 0.24 mmol, 1.1 equiv.), and NaHCO_3_ (55 mg, 0.66 mmol, 3 equiv.) in acetone (8 mL). After column chromatography (NP, CH_2_Cl_2_/CH_3_OH, v/v, 100:0−94:6), the pure product was isolated as a white solid (48 mg, 43%). ^1^H NMR (400 MHz, DMSO-*d*_*6*_) δ 11.68 (s, 1H), 10.74 − 10.57 (m, 1H), 8.37 − 8.21 (m, 1H), 7.58 (d, *J* = 2.6 Hz, 1H), 7.38 − 7.24 (m, 3H), 7.08 − 6.97 (m, 1H), 6.65 − 6.41 (m, 1H), 6.25 − 6.10 (m, 2H), 5.78 − 5.56 (m, 1H), 4.93 − 4.76 (m, 1H), 4.77 − 4.62 (m, 2H), 4.36 − 4.21 (m, 1H), 4.03 − 3.87 (m, 1H), 1.59 − 1.46 (m, 2H), 1.44 − 1.33 (m, 1H), 0.93 − 0.84 (m, 3H), 0.83 − 0.77 (m, 3H). ^13^C NMR (101 MHz, DMSO-*d*_*6*_) δ 171.20, 167.15, 166.63, 161.92, 152.50, 138.85, 134.23, 129.31, 128.67, 127.89, 126.70, 126.04, 124.99, 122.40, 115.27, 104.61, 67.39, 49.69, 46.85, 39.41, 24.14, 22.78, 21.41. TLC-MS (ESI) *m*/*z* for (C_23_H_26_Cl_2_N_4_O_5_ [M—H]^−^) calcd 507.13, found 507.1. HPLC t_*R*_ = 8.09 min.

#### (*S*)-2-(2,4-Dichlorophenoxy)-N-(1-(2-methacryloyl-2-((2-oxo-1,2-dihydropyridin-3-yl) methyl)­hydrazineyl)-4-methyl-1-oxopentan-2-yl)­acetamide (**6b**)

Following general procedure E for the synthesis of amides, compound **6b** was synthesized using **4a** (100 mg, 0.22 mmol, 1 equiv.), methacryloyl chloride (25 μL, 0.24 mmol, 1.1 equiv.), and NaHCO_3_ (55 mg, 0.66 mmol, 3 equiv.) in acetone (8 mL). After column chromatography (NP, CH_2_Cl_2_/CH_3_OH, v/v, 100:0−94:6), the pure product was isolated as a white solid (67 mg, 56%). ^1^H NMR (400 MHz, DMSO-*d*_*6*_) δ 11.68 (s, 1H), 10.72 − 10.52 (m, 1H), 8.23 − 8.11 (m, 1H), 7.59 (d, *J* = 2.5 Hz, 1H), 7.35 − 7.27 (m, 3H), 7.01 (d, *J* = 8.9 Hz, 1H), 6.19 − 6.13 (t, *J* = 6.6 Hz, 1H), 5.16 − 5.06 (m, 2H), 4.97 − 4.76 (m, 1H), 4.72 − 4.62 (m, 2H), 4.36 − 4.27 (m, 1H), 4.06 − 3.71 (m, 1H), 1.80 (s, 3H), 1.56 − 1.48 (m, 1H), 1.44 − 1.35 (m, 2H), 0.84 (d, *J* = 6.4 Hz, 3H), 0.80 (d, *J* = 6.4 Hz, 3H). ^13^C NMR (101 MHz, DMSO-*d*_*6*_) δ 170.66, 166.71, 166.66, 161.80, 152.46, 138.33, 134.15, 129.37, 129.33, 127.90, 126.14, 125.03, 122.41, 116.26, 115.24, 104.61, 67.40, 49.13, 47.01, 40.23, 24.07, 22.78, 21.58, 19.49. TLC-MS (ESI) *m*/*z* for (C_24_H_28_Cl_2_N_4_O_5_ [M—H]^−^) calcd 521.14, found 520.9. HPLC t_*R*_ = 8.85 min.

#### (*S*)-*N*-(1-(2-(2-Chloroacetyl)-2-((2-oxo-1,2-dihydropyridin-3-yl)­methyl)­hydrazineyl)-4-methyl-1-oxopentan-2-yl)-2-(2,4-dichlorophenoxy)­acetamide (**6c**)

Following general procedure E for the synthesis of amides, compound **6c** was synthesized using **4a** (100 mg, 0.22 mmol, 1 equiv.), 2-chloroacetyl chloride (19 μL, 0.24 mmol, 1.1 equiv.), and NaHCO_3_ (55 mg, 0.66 mmol, 3 equiv.) in acetone (8 mL). After column chromatography (NP, CH_2_Cl_2_/CH_3_OH, v/v, 100:0−94:6), the pure product was isolated as a white solid (66 mg, 56%). ^1^H NMR (400 MHz, DMSO-*d*_*6*_) δ 11.71 (s, 1H), 10.76 − 10.45 (m, 1H), 8.41 − 8.21 (m, 1H), 7.58 (d, *J* = 2.5 Hz, 1H), 7.38 − 7.27 (m, 3H), 7.07 − 6.96 (m, 1H), 6.16 (t, *J* = 6.6 Hz, 1H), 4.91 − 4.74 (m, 1H), 4.75 − 4.61 (m, 2H), 4.41 − 4.19 (m, 2H), 4.15 − 3.86 (m, 2H), 1.62 − 1.46 (m, 2H), 1.45 − 1.31 (m, 1H), 0.91 − 0.83 (m, 3H), 0.83 − 0.77 (m, 3H). ^13^C NMR (101 MHz, DMSO-*d*_*6*_) δ 171.52, 167.40 (2C), 161.92, 152.50, 139.33, 134.55, 129.35, 127.90, 125.47, 125.05, 122.41, 115.23, 104.64, 67.35, 49.80, 47.34, 42.19, 42.15, 24.16, 22.83, 21.37. TLC-MS (ESI) *m*/*z* for (C_22_H_25_Cl_3_N_4_O_5_ [M—H]^−^) calcd. 529.09, found 529.1. HPLC t_*R*_ = 8.27 min.

#### *N*-((2*S*)-1-(2-(2-Chloropropanoyl)-2-((2-oxo-1,2-dihydropyridin-3-yl)­methyl)­hydrazineyl)-4-methyl-1-oxopentan-2-yl)-2-(2,4-dichlorophenoxy)­acetamide (**6d**)

Following general procedure E for the synthesis of amides, compound **6d** was synthesized using **4a** (100 mg, 0.22 mmol, 1 equiv.), 2-chloropropanoyl chloride (23 μL, 0.24 mmol, 1.1 equiv.), and NaHCO_3_ (55 mg, 0.66 mmol, 3 equiv.) in acetone (8 mL). After column chromatography (NP, CH_2_Cl_2_/CH_3_OH, v/v, 100:0−94.5:5.5) the pure product was isolated as a white solid (97 mg, 81%). ^1^H NMR (400 MHz, DMSO-*d*_*6*_) δ 11.72 (s, 1H), 10.91 − 10.59 (m, 1H), 8.42 − 8.22 (m, 1H), 7.62 − 7.56 (m, 1H), 7.36 − 7.30 (m, 2H), 7.30 − 7.27 (m, 1H), 7.09 − 6.96 (m, 1H), 6.21 − 6.11 (m, 1H), 4.88 − 4.78 (m, 1H), 4.76 − 4.63 (m, 3H), 4.34 − 4.17 (m, 1H), 4.02 − 3.88 (m, 1H), 1.63 − 1.54 (m, 2H), 1.53 − 1.43 (m, 3H), 1.35 − 1.27 (m, 1H), 0.85 (d, *J* = 6.1 Hz, 3H), 0.83 − 0.79 (d, *J* = 6.1 Hz, 3H). ^13^C NMR (101 MHz, DMSO-*d*_*6*_) δ 170.61, 170.57, 167.33, 161.89, 152.51, 138.80, 134.46, 129.33, 127.88, 125.58, 125.03, 122.41, 115.25, 104.64, 67.30, 53.48, 49.88, 47.25, 40.06, 24.17, 22.86, 21.46, 21.36. TLC-MS (ESI) *m*/*z* for (C_23_H_27_Cl_3_N_4_O_5_ [M—H]^−^) calcd. 543.10, found 542.9. HPLC t_*R*_ = 8.60 min.

#### (*S*)-*N*-(1-(2-(2-Chloro-2,2-difluoroacetyl)-2-((2-oxo-1,2-dihydropyridin-3-yl)­methyl)­hydrazineyl)-4-methyl-1-oxopentan-2-yl)-2-(2,4-dichlorophenoxy)­acetamide (**6e**)

Following general procedure E for the synthesis of amides, compound **6e** was synthesized using **4a** (100 mg, 0.22 mmol, 1 equiv.), chlorodifluoroacetic anhydride (42 μL, 0.24 mmol, 1.1 equiv.), and NaHCO_3_ (55 mg, 0.66 mmol, 3 equiv.) in acetone (8 mL). After column chromatography (NP, CH_2_Cl_2_/CH_3_OH, v/v, 100:0−95:5), the pure product was isolated as a white solid (103 mg, 83%). ^1^H NMR (400 MHz, DMSO-*d*_*6*_) δ 11.77 (s, 1H), 10.88 − 10.73 (m, 1H), 8.30 − 8.20 (m, 1H), 7.64 − 7.55 (m, 1H), 7.44 − 7.37 (m, 1H), 7.37 − 7.33 (m, 1H), 7.32 − 7.25 (m, 1H), 7.10 − 6.96 (m, 1H), 6.23 − 6.09 (m, 1H), 5.01 − 4.84 (m, 1H), 4.80 − 4.60 (m, 2H), 4.48 − 4.34 (m, 1H), 4.09 − 3.90 (m, 1H), 1.66 − 1.46 (m, 2H), 1.45 − 1.31 (m, 1H), 0.91 − 0.84 (m, 3H), 0.84 − 0.78 (m, 3H). ^13^C NMR (101 MHz, DMSO-*d*_*6*_) δ 171.82, 166.84, 161.76, 158.87, 152.47, 140.64, 135.33, 129.32, 127.83, 125.04, 123.94, 122.47, 115.41 (t, *J* = 268.0 Hz), 115.16, 104.66, 67.45, 49.42, 48.61, 40.08, 24.05, 23.01, 21.16. TLC-MS (ESI) *m*/*z* for (C_22_H_23_Cl_3_F_2_N_4_O_5_ [M—H]^−^) calcd. 565.07, found 565.0. HPLC t_*R*_ = 9.04 min.

#### (*S*)-2-(2,4-Dichlorophenoxy)-*N*-(1-(2-(2,2-difluoroacetyl)-2-((2-oxo-1,2-dihydropyridin-3-yl)­methyl)­hydrazineyl)-4-methyl-1-oxopentan-2-yl)­acetamide (**7a**)

To an ice-cold solution of **4a** (100 mg, 0.22 mmol, 1 equiv.) and triethylamine (454 μL, 0.33 mmol, 1.5 equiv.) in CH_2_Cl_2_ (dry, 10 mL) was added 2,2-difluoroacetic anhydride (25 μL, 0.198 mmol, 0.9 equiv.). The mixture was stirred at room temperature for 1 − 3 h. Then, sat. NaHCO_3_ was added to the reaction mixture, followed by extraction with EtOAc (3 × 20 mL). The organic layer was washed with brine, dried over anhydrous NaSO_4_, filtered, and concentrated under reduced pressure. The pure product **7a** yielded after column chromatography (NP, CH_2_Cl_2_/CH_3_OH, v/v, 100:0−96:4) as a white solid (78 mg, 66%). ^1^H NMR (400 MHz, DMSO-*d*_*6*_) δ 11.76 (s, 1H), 10.98 − 10.52 (m, 1H), 8.46 − 8.21 (m, 1H), 7.58 (s, 1H), 7.42 − 7.24 (m, 3H), 7.11 − 6.89 (m, 1H), 6.43 − 6.10 (m, 2H), 4.88 − 4.74 (m, 1H), 4.74 − 4.60 (m, 2H), 4.34 − 4.15 (m, 1H), 4.07 − 3.91 (m, 1H), 1.59 − 1.45 (m, 2H), 1.43 − 1.24 (m, 1H), 0.92 (d, *J* = 6.8 Hz, 3H), 0.82 (d, *J* = 6.8 Hz, 3H). ^13^C NMR (101 MHz, DMSO-*d*_*6*_) δ 172.56, 168.07, 167.88, 162.35, 152.96, 140.68, 135.50, 129.80, 128.33, 125.50, 124.95, 122.87, 115.74, 105.21 (t, *J* = 237.5 Hz)., 105.10, 67.78, 50.37, 47.62, 39.66, 24.59, 23.35, 21.80. TLC-MS (ESI) *m*/*z* for (C_22_H_24_Cl_2_F_2_N_4_O_5_ [M + Na]^+^) calcd. 555.11, found 554.7. HPLC t_*R*_ = 8.64 min.

#### (*S*)-*N*-(1-(2-Benzyl-2-(2-chloroacetyl)­hydrazineyl)-4-methyl-1-oxopentan-2-yl)-2-(2,4-dichlorophenoxy)­acetamide (**8a**)

Following general procedure E for the synthesis of amides, compound **8a** was synthesized using **4b** (100 mg, 0.23 mmol, 1 equiv.), 2-chloroacetyl chloride (20 μL, 0.25 mmol, 1.1 equiv.), and NaHCO_3_ (58 mg, 0.69 mmol, 3 equiv.) in acetone (8 mL). After column chromatography (NP, petroleum ether/EtOAc, v/v, 100:0−65:35), the pure product was isolated as a white solid (91 mg, 77%). ^1^H NMR (400 MHz, CDCl_3_) δ 8.47 (s, 1H), 7.43 (d, *J* = 2.4 Hz, 1H), 7.32 − 7.27 (m, 2H), 7.26 (s, 1H), 7.25 − 7.21 (m, 3H), 7.02 − 6.94 (m, 1H), 6.78 (d, *J* = 8.8 Hz, 1H), 5.58 − 5.03 (m, 1H), 4.40 − 4.33 (m, 2H), 4.33 − 4.14 (m, 2H), 4.13 − 3.98 (m, 2H), 1.74 − 1.65 (m, 1H), 1.61 − 1.51 (m, 2H), 0.91 (d, *J* = 6.1 Hz, 3H), 0.89 (d, *J* = 6.3 Hz, 3H). ^13^C NMR (101 MHz, CDCl_3_) δ 170.36, 168.35, 168.25, 151.22, 134.87, 130.39, 128.99 (2C), 128.83 (2C), 128.20, 128.14, 128.01, 123.89, 114.84, 67.89, 50.79, 49.94, 41.26, 39.34, 24.77, 22.66, 22.03. TLC-MS (ESI) *m*/*z* for (C_23_H_26_Cl_3_N_3_O_4_ [M—H]^−^) calcd 512.10, found 512.9. HPLC t_*R*_ = 9.91 min.

#### (*S*)-*N*-(1-(2-(2-Chloroacetyl)-2-(3-fluorobenzyl)­hydrazineyl)-4-methyl-1-oxopentan-2-yl)-2-(2,4-dichlorophenoxy)­acetamide (**8b**)

Following general procedure E for the synthesis of amides, compound **8b** was synthesized using **4c** (100 mg, 0.22 mmol, 1 equiv.), 2-chloroacetyl chloride (19 μL, 0.24 mmol, 1.1 equiv.), and NaHCO_3_ (55 mg, 0.66 mmol, 3 equiv.) in acetone (8 mL). After column chromatography (NP, petroleum ether/EtOAc, v/v, 100:0−65:35), the pure product was isolated as a white solid (110 mg, 94%). ^1^H NMR (400 MHz, DMSO-*d*_*6*_) δ 10.87 − 10.58 (m, 1H), 8.34 (s, 1H), 7.58 (d, *J* = 2.6 Hz, 1H), 7.42 − 7.34 (m, 1H), 7.34 − 7.29 (m, 1H), 7.15 − 7.06 (m, 3H), 7.05 − 6.95 (m, 1H), 5.17 − 4.90 (m, 1H), 4.76 − 4.62 (m, 2H), 4.39 − 4.22 (m, 2H), 4.19 − 4.01 (m, 2H), 1.54 − 1.39 (m, 2H), 1.37 − 1.20 (m, 1H) 0.89 − 0.82 (m, 3H), 0.82 − 0.77 (m, 3H). ^13^C NMR (101 MHz, DMSO-*d*_*6*_) δ 171.34 (2C), 167.42, 162.11 (d, *J* = 243.5 Hz), 152.48, 138.57, 130.18, 129.34, 127.88, 125.03, 124.59, 122.40, 115.27, 115.06, 114.32, 67.31, 50.90, 49.80, 41.96, 41.48, 24.11, 22.67, 21.47. TLC-MS (ESI) *m*/*z* for (C_23_H_25_Cl_3_FN_3_O_4_ [M—H]^−^) calcd 530.09, found 530.2. HPLC t_*R*_ = 9.55 min.

#### (*S*)-*N*-(1-(2-(2-Chloroacetyl)-2-(3-chlorobenzyl)­hydrazineyl)-4-methyl-1-oxopentan-2-yl)-2-(2,4-dichlorophenoxy)­acetamide (**8c**)

Following general procedure E for the synthesis of amides, compound **8c** was synthesized using **4d** (100 mg, 0.21 mmol, 1 equiv.), 2-chloroacetyl chloride (18 μL, 0.23 mmol, 1.1 equiv.), and NaHCO_3_ (52 mg, 0.63 mmol, 3 equiv.) in acetone (8 mL). After column chromatography (NP, petroleum ether/EtOAc, v/v, 100:0−65:35), the pure product was isolated as a white solid (110 mg, 95%). ^1^H NMR (400 MHz, DMSO-*d*_*6*_) δ 10.87 − 10.57 (m, 1H), 8.43 − 8.24 (m, 1H), 7.58 (d, *J* = 2.6 Hz, 1H), 7.37 − 7.33 (m, 2H), 7.34 − 7.29 (m, 2H), 7.25 − 7.19 (m, 1H), 7.08 − 6.96 (m, 1H), 5.08 − 4.87 (m, 1H), 4.77 − 4.62 (m, 2H), 4.38 − 4.20 (m, 2H), 4.19 − 4.03 (m, 2H), 1.55 − 1.39 (m, 2H), 1.37 − 1.22 (m, 1H), 0.91 − 0.82 (m, 3H), 0.82 − 0.77 (m, 3H). ^13^C NMR (101 MHz, DMSO-*d*_*6*_) δ 171.84 (2C), 167.91, 152.96, 138.65, 133.45, 130.68, 129.82, 128.84, 128.36, 128.02, 127.78, 125.51, 122.87, 115.67, 67.77, 51.36, 50.22, 42.44, 41.98, 24.59, 23.16, 21.98. TLC-MS (ESI) *m*/*z* for (C_23_H_25_Cl_4_N_3_O_4_ [M—H]^−^) calcd 547.06, found 547.2. HPLC t_*R*_ = 9.93 min.

#### (*S*)-*N*-(1-(2-(3-Chloro-2-methoxybenzyl)-2-(2-chloroacetyl)­hydrazineyl)-4-methyl-1-oxopentan-2-yl)-2-(2,4-dichlorophenoxy)­acetamide (**8d**)

Following general procedure E for the synthesis of amides, compound **8d** was synthesized using **4e** (100 mg, 0.20 mmol, 1 equiv.), 2-chloroacetyl chloride (17 μL, 0.22 mmol, 1.1 equiv.), and NaHCO_3_ (50 mg, 0.60 mmol, 3 equiv.) in acetone (8 mL). After column chromatography (NP, petroleum ether/EtOAc, v/v, 100:0−65:35), the pure product was isolated as a white solid (68 mg, 59%). ^1^H NMR (400 MHz, DMSO-*d*_*6*_) δ 10.79 − 10.54 (m, 1H), 8.41 − 8.24 (m, 1H), 7.59 (d, *J* = 2.6 Hz, 1H), 7.49 − 7.40 (m, 1H), 7.37 − 7.29 (m, 1H), 7.27 − 7.19 (m, 1H), 7.13 (t, *J* = 7.8 Hz, 1H), 7.09 − 6.95 (m, 1H), 5.19 − 4.98 (m, 1H), 4.81 − 4.62 (m, 2H), 4.42 − 4.31 (m, 1H), 4.30 − 4.14 (m, 2H), 4.14 − 4.04 (m, 1H), 3.75 (s, 3H), 1.56 − 1.41 (m, 2H), 1.34 − 1.27 (m, 1H), 0.86 (d, *J* = 6.6 Hz, 3H), 0.83 (d, *J* = 6.5 Hz, 3H). ^13^C NMR (101 MHz, DMSO-*d*_*6*_) δ 171.90, 168.26, 167.85, 154.54, 152.96, 131.39, 130.40, 129.98, 129.81, 128.35, 127.25, 125.55, 125.51, 122.87, 115.68, 67.79, 61.17, 50.22, 46.32, 42.52, 38.86, 24.59, 23.23, 21.85. TLC-MS (ESI) *m*/*z* for (C_24_H_27_Cl_4_N_3_O [M + Na]^+^) calcd 600.07, found 599.7. HPLC t_*R*_ = 10.00 min.

#### (*S*)-*N*-(1-(2-(2-Chloroacetyl)-2-(2-(trifluoromethoxy)­benzyl)­hydrazineyl)-4-methyl-1-oxopentan-2-yl)-2-(2,4-dichlorophenoxy)­acetamide (**8e**)

Following general procedure E for the synthesis of amides, compound **8e** was synthesized using **4f** (100 mg, 0.19 mmol, 1 equiv.), 2-chloroacetyl chloride (16 μL, 0.21 mmol, 1.1 equiv.), and NaHCO_3_ (48 mg, 0.57 mmol, 3 equiv.) in acetone (8 mL). After column chromatography (NP, petroleum ether/EtOAc, v/v, 100:0−65:35) the pure product was isolated as a white solid (110 mg, 95%). ^1^H NMR (400 MHz, DMSO-*d*_*6*_) δ 10.89 − 10.51 (m, 1H), 8.44 − 8.18 (m, 1H), 7.58 (d, *J* = 2.6 Hz, 1H), 7.49 − 7.41 (m, 2H), 7.40 − 7.34 (m, 2H), 7.34 − 7.29 (m, 1H), 7.11 − 6.91 (m, 1H), 5.25 − 5.00 (m, 1H), 4.77 − 4.59 (m, 2H), 4.41 − 4.15 (m, 3H), 4.14 − 4.02 (m, 1H), 1.58 − 1.42 (m, 2H), 1.37 − 1.18 (m, 1H), 0.91 − 0.81 (m, 3H), 0.81 − 0.75 (m, 3H). ^13^C NMR (101 MHz, DMSO-*d*_*6*_) δ 171.97, 171.94, 167.89, 152.96, 147.33, 131.76, 130.24, 129.81, 128.54, 128.34, 127.91, 125.51, 122.87, 121.04, 120.56 (d, *J* = 257.0 Hz), 115.68, 67.79, 45.93, 45.85, 42.46, 42.40, 24.56, 23.21, 21.78. TLC-MS (ESI) *m*/*z* for (C_24_H_25_Cl_3_F_3_N_3_O_5_ [M + Na]^+^) calcd 620.08, found 619.7. HPLC t_*R*_ = 10.24 min.

#### (*S*)-*N*-(1-(2-(2-Chloroacetyl)-2-(pyridin-3-ylmethyl)­hydrazineyl)-4-methyl-1-oxopentan-2-yl)-2-(2,4-dichlorophenoxy)­acetamide (**8f**)

Following general procedure E for the synthesis of amides, compound **8f** was synthesized using **4g** (100 mg, 0.23 mmol, 1 equiv.), 2-chloroacetyl chloride (20 μL, 0.25 mmol, 1.1 equiv.), and NaHCO_3_ (58 mg, 0.69 mmol, 3 equiv.) in acetone (8 mL). After column chromatography (NP, CH_2_Cl_2_/CH_3_OH, v/v, 100:0−97.5:2.5), the pure product was isolated as a white solid (78 mg, 66%). ^1^H NMR (400 MHz, DMSO-*d*_*6*_) δ 10.88 − 10.56 (m, 1H), 8.52 − 8.49 (m, 1H), 8.49 − 8.46 (m, 1H), 8.40 − 8.32 (m, 1H), 7.72 − 7.66 (m, 1H), 7.59 (d, *J* = 2.6 Hz, 1H), 7.41 − 7.35 (m, 1H), 7.35 − 7.31 (m, 1H), 7.11 − 6.96 (m, 1H), 5.09 − 4.89 (m, 1H), 4.76 − 4.65 (m, 2H), 4.41 − 4.23 (m, 2H), 4.22 − 4.06 (m, 2H), 1.55 − 1.43 (m, 2H), 1.41 − 1.22 (m, 1H), 0.90 − 0.83 (m, 3H), 0.83 − 0.79 (m, 3H). ^13^C NMR (101 MHz, DMSO-*d*_*6*_) δ 171.86, 167.94 (2C), 152.97, 150.25, 149.28, 136.96, 131.83, 129.82 (2C), 128.27, 125.50, 123.92, 122.86, 115.68, 67.77, 53.81, 49.55, 42.48, 42.45, 24.59, 23.17. TLC-MS (ESI) *m*/*z* for (C_22_H_25_Cl_3_N_4_O_4_ [M—H]^−^) calcd 513.09, found 513.2. HPLC t_*R*_ = 8.80 min.

#### (*S*)-*N*-(1-(2-(2-Chloroacetyl)-2-(pyridin-4-ylmethyl)­hydrazineyl)-4-methyl-1-oxopentan-2-yl)-2-(2,4-dichlorophenoxy)­acetamide (**8g**)

Following general procedure E for the synthesis of amides, compound **8g** was synthesized using **4h** (100 mg, 0.23 mmol, 1 equiv.), 2-chloroacetyl chloride (20 μL, 0.25 mmol, 1.1 equiv.), and NaHCO_3_ (58 mg, 0.69 mmol, 3 equiv.) in acetone (8 mL). After column chromatography (NP, CH_2_Cl_2_/CH_3_OH, v/v, 100:0−97:3), the pure product was isolated as a white solid (71 mg, 60%). ^1^H NMR (400 MHz, DMSO-*d*_*6*_) δ 10.96 − 10.68 (m, 1H), 8.52 (d, *J* = 5.8 Hz, 2H), 8.42 − 8.32 (m, 1H), 7.59 (d, *J* = 2.5 Hz, 1H), 7.34 − 7.30 (m, 1H), 7.28 (d, *J* = 5.6 Hz, 2H), 7.06 − 6.98 (m, 1H), 5.14 − 4.91 (m, 1H), 4.75 − 4.64 (m, 2H), 4.47 − 4.26 (m, 2H), 4.22 − 4.07 (m, 2H), 1.60 − 1.46 (m, 2H), 1.44 − 1.31 (m, 1H), 0.88 − 0.82 (m, 3H), 0.82 − 0.78 (m, 3H).^13^C NMR (101 MHz, DMSO-*d*_*6*_) δ 171.89, 167.93 (2C), 152.96, 150.02 (2C), 145.27, 129.82, 128.36, 125.50, 123.60 (2C), 122.86, 115.67, 67.77, 51.28, 50.35, 42.37, 41.98, 24.59, 23.12, 21.92. TLC-MS (ESI) *m*/*z* for (C_22_H_25_Cl_3_N_4_O_4_ [M—H]^−^) calcd 513.09, found 513.3. HPLC t_*R*_ = 8.47 min.

#### (*S*)-*N*-(1-(2-(2-Chloroacetyl)-2-((1-methyl-1*H*-1,2,3-triazol-4-yl)­methyl)­hydrazineyl)-4-methyl-1-oxopentan-2-yl)-2-(2,4-dichlorophenoxy)­acetamide (**8h**)

Following general procedure E for the synthesis of amides, compound **8h** was synthesized using **4i** (100 mg, 0.23 mmol, 1 equiv.), 2-chloroacetyl chloride (20 μL, 0.25 mmol, 1.1 equiv.), and NaHCO_3_ (58 mg, 0.69 mmol, 3 equiv.) in acetone (8 mL). After column chromatography (NP, CH_2_Cl_2_/CH_3_OH, v/v, 100:0−95.4:4.6), the pure product was isolated as a white solid (90 mg, 75%). ^1^H NMR (400 MHz, DMSO-*d*_*6*_) δ 10.79 − 10.47 (m, 1H), 8.40 − 8.24 (m, 1H), 8.03 − 7.82 (m, 1H), 7.60 (d, *J* = 2.5 Hz, 1H), 7.34 (d, *J* = 7.9 Hz, 1H), 7.11 − 6.96 (m, 1H), 5.18 − 4.92 (m, 1H), 4.82 − 4.57 (m, 2H), 4.36 − 4.27 (m, 1H), 4.27 − 4.15 (m, 2H), 4.10 − 4.03 (m, 1H), 4.01 (s, 3H), 1.61 − 1.47 (m, 2H), 1.46 − 1.33 (m, 1H), 0.90 (d, *J* = 6.1 Hz, 3H), 0.83 (d, *J* = 6.1 Hz, 3H). ^13^C NMR (101 MHz, DMSO-*d*_*6*_) δ 171.91 (2C), 167.92, 152.97, 141.55, 129.81, 128.43, 128.38, 125.49, 122.84, 115.67, 67.76, 50.20, 46.63, 42.89, 42.55, 36.64, 24.59, 23.32, 21.85. TLC-MS (ESI) *m*/*z* for (C_20_H_25_Cl_3_N_6_O_4_ [M—H]^−^) calcd 517.10, found 517.2. HPLC t_*R*_ = 8.79 min.

#### (*S*)-*N*-(1-(2-(2-Chloroacetyl)-2-((2-oxo-1,2-dihydroquinolin-3-yl)­methyl)­hydrazineyl)-4-methyl-1-oxopentan-2-yl)-2-(2,4-dichlorophenoxy)­acetamide (**8i**)

Following general procedure E for the synthesis of amides, compound **8i** was synthesized using **4j** (100 mg, 0.20 mmol, 1 equiv.), 2-chloroacetyl chloride (17 μL, 0.22 mmol, 1.1 equiv.), and NaHCO_3_ (50 mg, 0.6 mmol, 3 equiv.) in acetone (8 mL). After column chromatography (NP, CH_2_Cl_2_/CH_3_OH, v/v, 100:0−96:4), the pure product was isolated as a white solid (106 mg, 91%). ^1^H NMR (400 MHz, DMSO-*d*_*6*_) δ 13.19 (s, 1H), 10.80 − 10.42 (m, 1H), 8.38 − 8.22 (m, 1H), 7.78 (s, 1H), 7.64 − 7.58 (m, 1H), 7.57 (d, *J* = 2.6 Hz, 1H), 7.52 − 7.45 (m, 1H), 7.36 − 7.27 (m, 2H), 7.22 − 7.13 (m, 1H), 7.06 − 6.93 (m, 1H), 5.03 − 4.81 (m, 1H), 4.74 − 4.61 (m, 2H), 4.42 − 4.30 (m, 1H), 4.30 − 4.25 (m, 2H), 4.21 − 4.14 (m, 1H), 1.56 − 1.36 (m, 2H), 1.34 − 1.21 (m, 1H), 0.89 − 0.74 (m, 3H), 0.75 − 0.64 (m, 3H). ^13^C NMR (101 MHz, DMSO-*d*_*6*_) δ 172.10, 169.06, 167.87, 161.88, 152.96, 138.96, 130.65, 129.80, 128.34, 128.26, 127.07, 127.04, 125.50, 122.87, 122.30, 119.36, 115.67, 115.39, 67.76, 47.30, 47.28, 42.72, 41.96, 24.55, 23.23, 21.74. TLC-MS (ESI) *m*/*z* for (C_26_H_27_Cl_3_N_4_O_5_ [M—H]^−^) calcd 579.10, found 579.1. HRMS (ESI-TOF) *m*/*z* for (C_26_H_27_Cl_3_N_4_O_5_ [M+H]^+^ calcd. 581.1125, found 581.1127. HPLC t_*R*_ = 8.99 min.

#### (*S*)-*N*-(1-(2-((5-Chloro-2-oxo-1,2-dihydropyridin-3-yl)­methyl)-2-(2-chloroacetyl) hydrazineyl)-4-methyl-1-oxopentan-2-yl)-2-(2,4-dichlorophenoxy)­acetamide (**8j**)

Following general procedure E for the synthesis of amides, compound **8j** was synthesized using **4k** (100 mg, 0.20 mmol, 1 equiv.), 2-chloroacetyl chloride (18 μL, 0.22 mmol, 1.1 equiv.), and NaHCO_3_ (50 mg, 0.6 mmol, 3 equiv.) in acetone (8 mL). After column chromatography (NP, CH_2_Cl_2_/CH_3_OH, v/v, 100:0−95.7:4.3), the pure product was isolated as a white solid (60 mg, 53%). ^1^H NMR (400 MHz, DMSO-*d*_*6*_) δ 12.04 (s, 1H), 10.81 − 10.47 (m, 1H), 8.38 − 8.23 (m, 1H), 7.58 (d, *J* = 2.6 Hz, 2H), 7.38 (d, *J* = 2.7 Hz, 1H), 7.35 − 7.30 (m, 1H), 7.07 − 7.00 (m, 1H), 4.88 − 4.60 (m, 3H), 4.50 − 4.30 (m, 1H), 4.31 − 4.05 (m, 3H), 1.61 − 1.48 (m, 2H), 1.48 − 1.35 (m, 1H), 0.92 (d, *J* = 5.8 Hz, 3H), 0.83 (d, *J* = 5.8 Hz, 3H). ^13^C NMR (101 MHz, DMSO-*d*_*6*_) δ 172.01, 168.19, 167.89, 160.89, 152.98, 139.38, 133.49, 129.81 (2C), 128.35 (2C), 125.54, 122.92, 115.75, 67.88, 50.39, 47.53, 42.52, 41.97, 24.63, 23.24, 21.86. TLC-MS (ESI) *m*/*z* for (C_22_H_24_Cl_4_N_4_O_5_ [M + H]^+^) calcd 565.05, found 565.2. HRMS (ESI-TOF) *m*/*z* for (C_22_H_24_Cl_4_N_4_O_5_ [M+H]^+^ calcd. 565.0579, found 565.0585. HPLC t_*R*_ = 8.72 min.

#### (*S*)-*N*-(1-(2-(2-Chloroacetyl)-2-((2,4-dioxo-1,2,3,4-tetrahydropyrimidin-5-yl)­methyl) hydrazineyl)-4-methyl-1-oxopentan-2-yl)-2-(2,4-dichlorophenoxy)­acetamide (**8k**)

Following general procedure E for the synthesis of amides, compound **8k** was synthesized using **4l** (100 mg, 0.21 mmol, 1 equiv.), 2-chloroacetyl chloride (19 μL, 0.23 mmol, 1.1 equiv.), and NaHCO_3_ (50 mg, 0.59 mmol, 3 equiv.) in acetone (8 mL). After column chromatography (NP, CH_2_Cl_2_/CH_3_OH, v/v, 100:0−94:6), the pure product was isolated as a white solid (102 mg, 89%). ^1^H NMR (400 MHz, DMSO-*d*_*6*_) δ 11.15 (s, 1H), 11.00 − 10.85 (m, 1H), 10.65 − 10.35 (m, 1H), 8.39 − 8.22 (m, 1H), 7.62 − 7.55 (m, 1H), 7.39 − 7.30 (m, 1H), 7.30 − 7.23 (m, 1H), 7.12 − 6.96 (m, 1H), 4.86 − 4.66 (m, 2H), 4.65 − 4.51 (m, 1H), 4.34 − 4.16 (m, 2H), 4.11 − 3.97 (m, 1H), 3.92 − 3.71 (m, 1H), 1.63 − 1.48 (m, 2H), 1.48 − 1.35 (m, 1H), 0.88 (d, *J* = 5.2 Hz, 3H), 0.84 (d, *J* = 6.4 Hz, 3H). ^13^C NMR (101 MHz, DMSO-*d*_*6*_) δ 172.04, 167.83, 164.28, 152.96, 151.81, 142.41, 142.22, 129.81, 128.38, 125.51, 122.86, 115.66, 106.28, 67.84, 50.91, 50.14, 43.91, 42.69, 24.65, 23.30, 21.91. TLC-MS (ESI) *m*/*z* for (C_21_H_24_Cl_3_N_5_O_6_ [M—H]^−^) calcd 546.08, found 546.6. HRMS (ESI-TOF) *m*/*z* for (C_21_H_24_Cl_3_N_5_O_6_ [M+H]^+^) calcd. 548.0870, found 548.0871. HPLC t_*R*_ = 1 8.11 min.

#### Ethyl (*S*)-2-(2-(2,4-dichlorophenoxy)­acetamido)-4-fluoro-4-methylpentanoate (**10a**)

Obtained from the reaction of (2,4-dichlorphenoxy)­acetic acid (221 mg, 1 mmol) and ethyl (*S*)-2-amino-4-fluoro-4-methylpentanoate • HCl (**9a**, 214 mg, 1 mmol) following general procedure A. Flash purification with petroleum ether/EtOAc (0 − 40% EtOAc). Yield: 342 mg (90%) of **10a** as a light-yellow sticky oil. ^1^H NMR (400 MHz, CDCl_3_) δ 7.45 − 7.37 (m, 2H), 7.24 − 7.18 (m, 1H), 6.83 (d, *J* = 8.8 Hz, 1H), 4.78 − 4.70 (m, 1H), 4.59 − 4.46 (m, 2H), 4.27 − 4.16 (m, 2H), 2.25 − 2.09 (m, 2H), 1.45 (d, *J* = 4.9 Hz, 3H), 1.40 (d, *J* = 4.9 Hz, 3H), 1.29 (t, *J* = 7.1 Hz, 3H). ESI-MS [M—H]^−^ = 377.8. HPLC t_*R*_ = 9.18 min.

#### Methyl (*S*)-2-(2-(2,4-dichlorophenoxy)­acetamido)-4,4-dimethylpentanoate (**10b**)

Obtained from the reaction of (2,4-dichlorphenoxy)­acetic acid (221 mg, 1 mmol) and methyl (*S*)-2-amino-4,4-dimethylpentanoate • HCl (**9b**, 159 mg, 1 mmol) following general procedure A. Flash purification with petroleum ether/EtOAc (0 − 40% EtOAc). Yield: 312 mg (86%) of **10a** as a light-yellow oil. ^1^H NMR (400 MHz, CDCl_3_) δ 7.42 (d, *J* = 2.5 Hz, 1H), 7.25 − 7.19 (m, 1H), 7.07 − 6.98 (m, 1H), 6.84 (d, *J* = 8.8 Hz, 1H), 4.74 − 4.65 (m, 1H), 4.58 − 4.46 (m, 2H), 3.74 (s, 3H), 1.86 (dd, *J* = 14.5, 3.5 Hz, 1H), 1.58 (dd, *J* = 14.5, 9.1 Hz, 1H), 0.97 (s, 9H). ESI-MS [M + Na]^+^ = 383.6. HPLC t_*R*_ = 9.84 min.

#### Methyl (*S*)-3-cyclopropyl-2-(2-(2,4-dichlorophenoxy)­acetamido)­propanoate (**10c**)

Obtained from the reaction of (2,4-dichlorphenoxy)­acetic acid (221 mg, 1 mmol) and methyl (*S*)-2-amino-3-cyclopropylpropanoate • HCl (**9c**, 180 mg, 1 mmol) following general procedure A. Flash purification with petroleum ether/EtOAc (0−50% EtOAc). Yield: 297 mg (86%) of **10c** as a white solid. ^1^H NMR (400 MHz, CDCl_3_) δ 7.47 − 7.39 (m, 2H), 7.24 − 7.20 (m, 1H), 6.87 − 6.83 (m, 1H), 4.79 − 4.72 (m, 1H), 4.54 (s, 2H), 3.77 (s, 3H), 1.81 − 1.74 (m, 2H), 0.73 − 0.63 (m, 1H), 0.53 − 0.43 (m, 2H), 0.13 − 0.03 (m, 2H). ESI-MS [M + Na]^+^ = 368.2. HPLC t_*R*_ = 9.00 min.

#### Ethyl (2-(2,4-dichlorophenoxy)­acetyl)-l-phenylalaninate (**10d**)

Obtained from the reaction of (2,4-dichlorphenoxy)­acetic acid (221 mg, 1 mmol) and ethyl *L*-phenylalaninate • HCl (**9d**, 230 mg, 1 mmol) following general procedure A. Flash purification with petroleum ether/EtOAc (0−45% EtOAc). Yield: 340 mg (86%) of **10d** as a white solid. ^1^H NMR (400 MHz, CDCl_3_) δ 7.32 (d, *J* = 2.5 Hz, 1H), 7.19 − 7.16 (m, 3H), 7.14 − 7.09 (m, 2H), 7.07 − 7.04 (m, 2H), 6.71 − 6.67 (m, 1H), 4.89 − 4.83 (m, 1H), 4.47 − 4.37 (m, 2H), 4.15 − 4.08 (m, 2H), 3.14 − 3.04 (m, 2H), 1.17 (t, *J* = 7.1 Hz, 3H). ESI-MS [M—H]^−^ = 394.1. HPLC t_*R*_ = 10.72 min.

#### Methyl (*S*)-2-(2-(2,4-dichlorophenoxy)­acetamido)-3-(2-fluorophenyl)­propanoate (**10e**)

Obtained from the reaction of (2,4-dichlorphenoxy)­acetic acid (221 mg, 1 mmol) and methyl (*S*)-2-amino-3-(2-fluorophenyl)­propanoate • HCl (**9e**, 234 mg, 1 mmol) following general procedure A. Flash purification with petroleum ether/EtOAc (0−45% EtOAc). Yield: 380 mg (95%) of **10e** as a light-yellow solid. ^1^H NMR (400 MHz, CDCl_3_) δ 7.32 (d, *J* = 2.5 Hz, 1H), 7.19 − 7.13 (m, 2H), 7.14 − 7.09 (m, 1H), 7.09 − 7.03 (m, 1H), 7.00 − 6.95 (m, 1H), 6.95 − 6.89 (m, 1H), 6.70 (d, *J* = 8.8 Hz, 1H), 4.91 − 4.83 (m, 1H), 4.47 − 4.35 (m, 2H), 3.69 (s, 3H), 3.21 (dd, *J* = 14.1, 5.6 Hz, 1H), 3.10 (dd, *J* = 14.0, 6.8 Hz, 1H). ESI-MS [M + Na]^+^ = 421.6. HPLC t_*R*_ = 9.33 min.

#### Methyl (*S*)-2-(2-(2,4-dichlorophenoxy)­acetamido)-3-(3-fluorophenyl)­propanoate (**10f**)

Obtained from the reaction of (2,4-dichlorphenoxy)­acetic acid (221 mg, 1 mmol) and methyl (*S*)-2-amino-3-(3-fluorophenyl)­propanoate • HCl (**9f**, 234 mg, 1 mmol) following general procedure A. Flash purification with petroleum ether/EtOAc (0−45% EtOAc). Yield: 343 mg (86%) of **10f** as a white solid. ^1^H NMR (400 MHz, CDCl_3_) δ 7.39 (d, *J* = 2.5 Hz, 1H), 7.25 − 7.21 (m, 1H), 7.21 − 7.16 (m, 2H), 6.97 − 6.91 (m, 1H), 6.91 − 6.87 (m, 1H), 6.85 − 6.81 (m, 1H), 6.78 (d, *J* = 8.8 Hz, 1H), 4.99 − 4.92 (m, 1H), 4.56 − 4.45 (m, 2H), 3.76 (s, 3H), 3.23 − 3.10 (m, 2H). ESI-MS [M—H]^−^ = 398.2. HPLC t_*R*_ = 9.09 min.

#### Methyl (*S*)-2-(2-(2,4-dichlorophenoxy)­acetamido)-3-(4-fluorophenyl)­propanoate (**10g**)

Obtained from the reaction of (2,4-dichlorphenoxy)­acetic acid (221 mg, 1 mmol) and methyl (*S*)-2-amino-3-(4-fluorophenyl)­propanoate • HCl (**9g**, 234 mg, 1 mmol) following general procedure A. Flash purification with petroleum ether/EtOAc (0−50% EtOAc). Yield: 303 mg (76%) of **10g** as a white solid. ^1^H NMR (400 MHz, CDCl_3_) δ 7.40 (d, *J* = 2.5 Hz, 1H), 7.22 − 7.15 (m, 2H), 7.10 − 7.04 (m, 2H), 6.98 − 6.92 (m, 2H), 6.78 (d, *J* = 8.8 Hz, 1H), 4.98 − 4.88 (m, 1H), 4.56 − 4.41 (m, 2H), 3.74 (s, 3H), 3.17 (dd, *J* = 14.1, 5.6 Hz, 1H), 3.11 (dd, *J* = 14.1, 6.4 Hz, 1H). ESI-MS [M + Na]^+^ = 422.5. HPLC t_*R*_ = 9.10 min.

#### Methyl (*S*)-2-(2-(2,4-dichlorophenoxy)­acetamido)-3-(3,5-difluorophenyl)­propanoate (**10h**)

Obtained from the reaction of (2,4-dichlorphenoxy)­acetic acid (221 mg, 1 mmol) and methyl (*S*)-2-amino-3-(3,5-difluorophenyl)­propanoate • HCl (**9h**, 252 mg, 1 mmol) following general procedure A. Flash purification with petroleum ether/EtOAc (0−50% EtOAc). Yield: 355 mg (85%) of **10h** as an off white solid. ^1^H NMR (400 MHz, CDCl_3_) δ 7.40 (d, *J* = 2.5 Hz, 1H), 7.25 − 7.22 (m, 1H), 7.22 − 7.18 (m, 1H), 6.81 − 6.77 (m, 1H), 6.73 − 6.67 (m, 1H), 6.67 − 6.63 (m, 2H), 4.98 − 4.91 (m, 1H), 4.58 − 4.44 (m, 2H), 3.77 (s, 3H), 3.19 (dd, *J* = 14.0, 5.6 Hz, 1H), 3.12 (dd, *J* = 14.0, 6.3 Hz, 1H). ESI-MS [M + Na]^+^ = 440.5. HPLC t_*R*_ = 9.26 min.

#### Methyl (*S*)-2-(2-(2,4-dichlorophenoxy)­acetamido)-3-(3-chlororophenyl)­propanoate (**10i**)

Obtained from the reaction of (2,4-dichlorphenoxy)­acetic acid (221 mg, 1 mmol) and methyl (*S*)-2-amino-3-(3-chlororophenyl)­propanoate • HCl (**9i**, 250 mg, 1 mmol) following general procedure A. Flash purification with petroleum ether/EtOAc (0−45% EtOAc). Yield: 396 mg (95%) of **10i** as a light-yellow solid. ^1^H NMR (400 MHz, CDCl_3_) δ 7.33 (d, *J* = 2.5 Hz, 1H), 7.18 − 7.14 (m, 2H), 7.14 − 7.10 (m, 2H), 7.08 − 7.03 (m, 1H), 6.96 − 6.92 (m, 1H), 6.71 (d, *J* = 8.8 Hz, 1H), 4.92 − 4.82 (m, 1H), 4.50 − 4.36 (m, 2H), 3.69 (s, 3H), 3.11 (dd, *J* = 14.0, 5.6 Hz, 1H), 3.04 (dd, *J* = 14.0, 6.6 Hz, 1H). ESI-MS [M + Na]^+^ = 437.6. HPLC t_*R*_ = 9.77 min.

#### Methyl (*S*)-3-cyclohexyl-2-(2-(2,4-dichlorophenoxy)­acetamido)­propanoate (**10j**)

Obtained from the reaction of (2,4-dichlorphenoxy)­acetic acid (221 mg, 1 mmol) and methyl (*S*)-2-amino-3-ccyclohexylpropanoate • HCl (**9j**, 222 mg, 1 mmol) following general procedure A. Flash purification with petroleum ether/EtOAc (0−40% EtOAc). Yield: 346 mg (89%) of **10j** as a white solid. ^1^H NMR (400 MHz, CDCl_3_) δ 7.42 (d, *J* = 2.5 Hz, 1H), 7.25 − 7.20 (m, 1H), 7.12 − 7.08 (m, 1H), 6.85 (d, *J* = 8.8 Hz, 1H), 4.73 − 4.67 (m, 1H), 4.54 (s, 2H), 3.74 (s, 3H), 1.73 − 1.59 (m, 7H), 1.35 − 1.26 (m, 1H), 1.22 − 1.10 (m, 3H), 0.99 − 0.86 (m, 2H). ESI-MS [M + Na]^+^ = 410.3. HPLC t_*R*_ = 10.79 min.

#### Methyl 2-(2-(2,4-dichlorophenoxy)­acetamido)-2,3-dihydro-1*H*-indene-2-carboxylate (**10k**)

Obtained from the reaction of (2,4-dichlorphenoxy)­acetic acid (221 mg, 1 mmol) and methyl 2-amino-2,3-dihydro-1*H*-indene-2-carboxylate • HCl (**9k**, 228 mg, 1 mmol) following general procedure A. Flash purification with petroleum ether/EtOAc (0−40% EtOAc). Yield: 369 mg (94%) of **10k** as a white solid. ^1^H NMR (400 MHz, CDCl_3_) δ 7.36 − 7.33 (m, 1H), 7.29 − 7.26 (m, 1H), 7.23 − 7.20 (m, 4H), 7.20 − 7.16 (m, 1H), 6.81 − 6.77 (m, 1H), 4.47 (s, 2H), 3.77 (s, 3H), 3.72 (s, 1H), 3.68 (s, 1H), 3.35 (s, 1H), 3.31 (s, 1H). ESI-MS [M + Na]^+^ = 416.5. HPLC t_*R*_ = 9.18 min.

#### Methyl (2-(2,4-dichlorophenoxy)­acetyl)-l-prolinate (**10l**)

Obtained from the reaction of (2,4-dichlorphenoxy)­acetic acid (221 mg, 1 mmol) and methyl *L*-prolinate • HCl (**9l**, 166 mg, 1 mmol) following general procedure B. Flash purification with petroleum ether/EtOAc (0−60% EtOAc). Yield: 305 mg (92%) of **10l** as a white solid. ^1^H NMR (400 MHz, CDCl_3_) δ 7.38 − 7.35 (m, 1H), 7.19 − 7.14 (m, 1H), 6.93 − 6.87 (m, 1H), 4.80 − 4.66 (m, 2H), 4.55 − 4.48 (m, 1H), 3.77 − 3.65 (m, 5H), 2.24 − 1.91 (m, 4H). ESI-MS [M + Na]^+^ = 354.4. HPLC t_*R*_ = 7.31 min.

#### Methyl (1*R*,2*S*,5*S*)-3-(2-(2,4-dichlorophenoxy)­acetyl)-6,6-dimethyl-3-azabicyclo*[3.1.0]*hexane-2-carboxylate (**10m**)

Obtained from the reaction of (2,4-dichlorphenoxy)­acetic acid (221 mg, 1 mmol) and (1*R*,2*S*,5*S*)-methyl 6,6-dimethyl-3-azabicyclo*[3.1.0]*hexane-2-carboxylate • HCl (**9m**, 206 mg, 1 mmol) following general procedure B. Yield: 334 mg (90%) of **10m** as a light yellow oil. ^1^H NMR (400 MHz, CDCl_3_) δ 7.36 (d, J = 2.5 Hz, 1H), 7.19 − 7.14 (m, 1H), 6.92 − 6.88 (m, 1H), 4.79 − 4.66 (m, 2H), 4.66 − 4.56 (m, 1H), 4.35 (d, J = 3.4 Hz, 1H), 4.20 − 4.10 (m, 2H), 3.89 − 3.83 (m, 1H), 3.66 − 3.55 (m, 1H), 2.87 − 2.74 (m, 1H), 2.67 − 2.59 (m, 1H), 2.05 − 1.93 (m, 1H), 1.92 − 1.83 (m, 1H), 1.76 − 1.65 (m, 1H), 1.63 − 1.55 (m, 2H), 1.48 − 1.39 (m, 1H). ESI-MS [M + Na]^+^ = 394.0. HPLC t_*R*_ = 8.83 min.

#### Methyl (*S*)-1-(2-(2,4-dichlorophenoxy)­acetyl)­indoline-2-carboxylate (**10n**)

Obtained from the reaction of (2,4-dichlorphenoxy)­acetic acid (221 mg, 1 mmol) and methyl (*S*)-indoline-2-carboxylate • HCl (**9n**, 214 mg, 1 mmol) following general procedure B. Flash purification with petroleum ether/EtOAc (0−40% EtOAc). Yield: 307 mg (81%) of **10n** as a white solid. ^1^H NMR (400 MHz, CDCl_3_) δ 8.40 − 8.13 (m, 1H), 7.38 (d, *J* = 2.5 Hz, 1H), 7.26 − 7.16 (m, 3H), 7.13 − 7.06 (m, 1H), 7.03 − 6.90 (m, 1H), 5.42 − 5.21 (m, 1H), 5.14 − 4.71 (m, 2H), 3.73 (s, 3H), 3.68 − 3.51 (m, 1H), 3.47 − 3.15 (m, 1H). ESI-MS [M + Na]^+^ = 402.5. HPLC t_*R*_ = 8.62 min.

#### Methyl (2*s*,3*as*,7*as*)-1-(2-(2,4-dichlorophenoxy)­acetyl)­octahydro-1*H*-indole-2-carboxylate (**10o**)

Obtained from the reaction of (2,4-dichlorphenoxy)­acetic acid (221 mg, 1 mmol) and methyl (2*S*,3*aS*,7*aS*)-octahydro-1*H*-indole-2-carboxylate • HCl (**9o**, 220 mg, 1 mmol) following general procedure B. Flash purification with petroleum ether/EtOAc (0−40% EtOAc). Yield: 369 mg (96%) of **10o** as a white solid. ^1^H NMR (400 MHz, CDCl_3_) δ 7.38 − 7.34 (m, 1H), 7.19 − 7.14 (m, 1H), 6.92 (d, *J* = 8.8 Hz, 1H), 4.82 − 4.75 (m, 1H), 4.72 − 4.66 (m, 1H), 4.51 − 4.43 (m, 1H), 4.07 − 3.99 (m, 1H), 3.73 (s, 3H), 2.20 − 2.10 (m, 1H), 2.05 − 1.93 (m, 2H), 1.79 − 1.71 (m, 2H), 1.70 − 1.63 (m, 2H), 1.63 − 1.47 (m, 2H), 1.31 − 1.18 (m, 2H). ESI-MS [M + Na]^+^ = 408.5. HPLC t_*R*_ = 9.18 min.

#### Methyl (*S*)-1-(2-(2,4-dichlorophenoxy)­acetyl)­piperidine-2-carboxylate (**10p**)

Obtained from the reaction of (2,4-dichlorphenoxy)­acetic acid (221 mg, 1 mmol) and methyl (*S*)-piperidine-2-carboxylate • HCl (**9p**,180 mg, 1 mmol) following general procedure B. Flash purification with petroleum ether/EtOAc (0−50% EtOAc). Yield: 304 mg (88%) of **10p** as a colorless oil. ^1^H NMR (400 MHz, CDCl_3_) δ 7.39 − 7.35 (m, 1H), 7.20 − 7.14 (m, 1H), 6.99 − 6.90 (m, 1H), 5.31 − 5.24 (m, 1H), 4.91 − 4.74 (m, 2H), 3.96 − 3.87 (m, 1H), 3.73 − 3.66 (m, 3H), 3.33 − 3.23 (m, 1H), 2.36 − 2.21 (m, 1H), 1.78 − 1.69 (m, 2H), 1.53 − 1.41 (m, 1H), 1.39 − 1.25 (m, 2H). ESI-MS [M + Na]^+^ = 368.2. HPLC t_*R*_ = 8.36 min.

#### Methyl (*S*)-2-(2-(2,4-dichlorophenoxy)­acetyl)-1,2,3,4-tetrahydroisoquinoline-3-carboxylate (**10q**)

Obtained from the reaction of (2,4-dichlorphenoxy)­acetic acid (221 mg, 1 mmol) and methyl (*S*)-1,2,3,4-tetrahydroisoquinoline-3-carboxylate • HCl (**9q**, 228 mg, 1 mmol) following general procedure B. Flash purification with petroleum ether/EtOAc (0−40% EtOAc). Yield: 287 mg (73%) of **10q** as a white solid. ^1^H NMR (400 MHz, CDCl_3_) δ 7.39 − 7.36 (m, 1H), 7.25 − 7.19 (m, 2H), 7.18 − 7.11 (m, 3H), 7.02 − 6.93 (m, 1H), 5.38 − 5.27 (m, 1H), 4.98 − 4.87 (m, 2H), 4.86 − 4.75 (m, 2H), 3.61 − 3.56 (m, 3H), 3.30 − 3.10 (m, 2H). ESI-MS [M + Na]^+^ = 416.5. HPLC t_*R*_ = 8.79 min.

#### Ethyl (1*s*,3*ar*,6*as*)-2-(2-(2,4-dichlorophenoxy)­acetyl)­octahydrocyclopenta*[c]*pyrrole-1-carboxylate (**10r**)

Obtained from the reaction of (2,4-dichlorphenoxy)­acetic acid (221 mg, 1 mmol) and (1*S*,3*aR*,6*aS*)-ethyl octahydrocyclopenta*[c]*pyrrole-1-carboxylate • HCl (**9r**, 220 mg, 1 mmol) following general procedure B. Yield: 335 mg (87%) of **10r** as a light yellow oil. ^1^H NMR (400 MHz, CDCl_3_) δ 7.36 (d, *J* = 2.5 Hz, 1H), 7.18 − 7.14 (m, 1H), 6.88 − 6.84 (m, 1H), 4.71 − 4.62 (m, 2H), 4.62 − 4.50 (m, 1H), 4.44 (s, 1H), 3.89 (dd, *J* = 10.5, 5.3 Hz, 1H), 3.75 − 3.73 (m, 1H), 3.73 − 3.71 (m, 3H), 1.62 − 1.41 (m, 2H), 1.08 − 1.02 (m, 4H), 0.90 (s, 2H), 0.82 (s, 1H). ESI-MS [M + Na]^+^ = 408.0. HPLC t_*R*_ = 9.66 min.

#### (*S*)-2-(2,4-Dichlorophenoxy)-*N*-(4-fluoro-1-hydrazineyl-4-methyl-1-oxopentan-2-yl)­acetamide (**11a**)

Obtained from **10a** (380 mg, 1 mmol) following general procedure B. Yield: mg (100%) of **11a** as a white solid. ^1^H NMR (400 MHz, DMSO-*d*_*6*_) δ 9.53 − 9.23 (m, 1H), 8.26 (d, *J* = 8.4 Hz, 1H), 7.65 (d, *J* = 2.6 Hz, 1H), 7.45 − 7.37 (m, 1H), 7.11 (d, *J* = 8.9 Hz, 1H), 4.73 (s, 2H), 4.59 − 4.45 (m, 1H), 4.44 − 4.00 (m, 2H), 2.19 − 2.07 (m, 1H), 2.05 − 1.90 (m, 1H), 1.40 (d, *J* = 3.1 Hz, 3H), 1.34 (d, *J* = 3.2 Hz, 3H). ESI-MS [M + Na]^+^ = 387.7. HPLC t_*R*_ = 7.65 min.

#### (*S*)-2-(2,4-Dichlorophenoxy)-*N*-(1-hydrazineyl-4,4-dimethyl-1-oxopentan-2-yl)­acetamide (**11b**)

Obtained from **10b** (362 mg, 1 mmol) following general procedure B. Yield: 362 mg (100%) of **11b** as a white solid. ^1^H NMR (400 MHz, DMSO-*d*_*6*_) δ 9.28 (s, 1H), 8.06 (d, *J* = 8.7 Hz, 1H), 7.59 (d, *J* = 2.6 Hz, 1H), 7.39 − 7.30 (m, 1H), 7.05 (d, *J* = 8.9 Hz, 1H), 4.65 (s, 2H), 4.40 − 4.31 (m, 1H), 4.28 − 4.09 (m, 2H), 1.63 (dd, *J* = 14.1, 4.2 Hz, 1H), 1.48 (dd, *J* = 14.1, 8.6 Hz, 1H), 0.87 (s, 9H). ESI-MS [M + Na]^+^ = 383.6. HPLC t_*R*_ = 8.85 min.

#### (*S*)-*N*-(3-Cyclopropyl-1-hydrazineyl-1-oxopropan-2-yl)-2-(2,4-dichlorophenoxy)­acetamide (**11c**)

Obtained from **10c** (346 mg, 1 mmol) following general procedure B. Yield: 346 mg (100%) of **11c** as a white solid. ^1^H NMR (400 MHz, DMSO-*d*_*6*_) δ 9.24 − 9.13 (m, 1H), 8.01 (d, *J* = 8.3 Hz, 1H), 7.52 (d, *J* = 2.6 Hz, 1H), 7.31 − 7.27 (m, 1H), 7.01 (d, *J* = 8.9 Hz, 1H), 4.67 − 4.57 (m, 2H), 4.34 − 4.27 (m, 1H), 4.27 − 3.95 (m, 2H), 1.57 − 1.47 (m, 1H), 1.40 − 1.31 (m, 1H), 0.64 − 0.52 (m, 1H), 0.36 − 0.22 (m, 2H), 0.05 − 0.09 (m, 2H). ESI-MS [M—H]^−^ = 344.2. HPLC t_*R*_ = 7.57 min.

#### (*S*)-2-(2,4-Dichlorophenoxy)-*N*-(1-hydrazineyl-1-oxo-3-phenylpropan-2-yl)­acetamide (**11d**)

Obtained from **10c** (382 mg, 1 mmol) following general procedure B. Yield: 382 mg (100%) of **11d** as a white solid. ^1^H NMR (400 MHz, DMSO-*d*_*6*_) δ 9.31 (s, 1H), 8.17 (d, *J* = 8.6 Hz, 1H), 7.56 (d, *J* = 2.5 Hz, 1H), 7.28 − 7.18 (m, 6H), 6.76 (d, *J* = 8.9 Hz, 1H), 4.65 − 4.50 (m, 3H), 4.33 − 4.19 (m, 2H), 3.00 (dd, *J* = 13.6, 4.7 Hz, 1H), 2.82 (dd, *J* = 13.6, 9.5 Hz, 1H). ESI-MS [M + Na]^+^ = 404.2. HPLC t_*R*_ = 8.65 min.

#### (*S*)-2-(2,4-Dichlorophenoxy)-*N*-(3-(2-fluorophenyl)-1-hydrazineyl-1-oxopropan-2-yl)­acetamide (**11e**)

Obtained from **10e** (400 mg, 1 mmol) following general procedure B. Yield: 400 mg (100%) of **11e** as a white solid. ^1^H NMR (400 MHz, DMSO-*d*_*6*_) δ 9.35 (s, 1H), 8.18 (d, *J* = 8.8 Hz, 1H), 7.58 (d, *J* = 2.6 Hz, 1H), 7.30 − 7.24 (m, 3H), 7.15 − 7.05 (m, 2H), 6.79 (d, *J* = 8.9 Hz, 1H), 4.68 − 4.56 (m, 3H), 4.35 − 4.17 (m, 2H), 3.09 (dd, *J* = 13.9, 5.0 Hz, 1H), 2.86 (dd, *J* = 13.8, 9.4 Hz, 1H). ESI-MS [M + Na]^+^ = 421.6. HPLC t_*R*_ = 8.27 min.

#### (*S*)-2-(2,4-Dichlorophenoxy)-*N*-(3-(3-fluorophenyl)-1-hydrazineyl-1-oxopropan-2-yl)­acetamide (**11f**)

Obtained from **10f** (400 mg, 1 mmol) following general procedure B. Yield: 400 mg (100%) of **11f** as a white solid. ^1^H NMR (400 MHz, DMSO-*d*_*6*_) δ 9.44 − 9.19 (m, 1H), 8.25 (d, *J* = 8.6 Hz, 1H), 7.56 (d, *J* = 2.6 Hz, 1H), 7.33 − 7.26 (m, 1H), 7.25 − 7.20 (m, 1H), 7.06 − 7.02 (m, 3H), 6.78 (d, *J* = 8.9 Hz, 1H), 4.60 − 4.52 (m, 3H), 4.34 − 4.21 (m, 2H), 3.02 (dd, *J* = 13.7, 4.7 Hz, 1H), 2.84 (dd, *J* = 13.7, 9.7 Hz, 1H). ESI-MS [M + Na]^+^ = 422.2. HPLC t_*R*_ = 8.13 min.

#### (*S*)-2-(2,4-Dichlorophenoxy)-*N*-(3-(4-fluorophenyl)-1-hydrazineyl-1-oxopropan-2-yl)­acetamide (**11g**)

Obtained from **10g** (400 mg, 1 mmol) following general procedure B. Yield: 400 mg (100%) of **11g** as a white solid. ^1^H NMR (400 MHz, DMSO-*d*_*6*_) δ 9.29 (s, 1H), 8.17 (d, *J* = 8.6 Hz, 1H), 7.60 − 7.54 (m, 1H), 7.28 − 7.18 (m, 3H), 7.07 (t, *J* = 8.8 Hz, 2H), 6.78 (d, *J* = 8.9 Hz, 1H), 4.66 − 4.56 (m, 2H), 4.55 − 4.46 (m, 1H), 4.33 − 4.21 (m, 2H), 2.98 (dd, *J* = 13.7, 4.7 Hz, 1H), 2.81 (dd, *J* = 13.6, 9.5 Hz, 1H). ESI-MS [M + Na]^+^ = 422.5. HPLC t_*R*_ = 8.19 min.

#### (*S*)-2-(2,4-Dichlorophenoxy)-*N*-(3-(3,5-difluorophenyl)-1-hydrazineyl-1-oxopropan-2-yl)­acetamide (**11h**)

Obtained from **10h** (418 mg, 1 mmol) following general procedure B. Yield: 418 mg (100%) of **11h** as a white solid. ^1^H NMR (400 MHz, DMSO-*d*_*6*_) δ 9.29 (s, 1H), 8.21 (d, *J* = 8.6 Hz, 1H), 7.57 (d, *J* = 2.6 Hz, 1H), 7.25 − 7.18 (m, 1H), 7.11 − 7.02 (m, 1H), 6.98 − 6.89 (m, 2H), 6.82 (d, *J* = 8.9 Hz, 1H), 4.64 − 4.51 (m, 3H), 4.37 − 4.20 (m, 2H), 3.03 (dd, *J* = 13.7, 4.7 Hz, 1H), 2.87 (dd, *J* = 13.7, 9.7 Hz, 1H). ESI-MS [M + Na]^+^ = 440.8. HPLC t_*R*_ = 8.41 min.

#### (*S*)-2-(2,4-Dichlorophenoxy)-*N*-(3-(3-chlorophenyl)-1-hydrazineyl-1-oxopropan-2-yl)­acetamide (**11i**)

Obtained from **10i** (417 mg, 1 mmol) following general procedure B. Yield: 417 mg (100%) of **10i** as a white solid. ^1^H NMR (400 MHz, DMSO-*d*_*6*_) δ 9.32 (s, 1H), 8.24 (d, *J* = 8.7 Hz, 1H), 7.57 (d, *J* = 2.6 Hz, 1H), 7.32 − 7.27 (m, 3H), 7.26 − 7.21 (m, 1H), 7.21 − 7.15 (m, 1H), 6.77 (d, *J* = 8.9 Hz, 1H), 4.66 − 4.57 (m, 2H), 4.58 − 4.51 (m, 1H), 4.35 − 4.21 (m, 2H), 3.01 (dd, *J* = 13.7, 4.6 Hz, 1H), 2.83 (dd, *J* = 13.7, 9.8 Hz, 1H). ESI-MS [M + Na]^+^ = 437.6. HPLC t_*R*_ = 8.94 min.

#### (*S*)-*N*-(3-Cyclohexyl-1-hydrazineyl-1-oxopropan-2-yl)-2-(2,4-dichlorophenoxy)­acetamide (**11j**)

Obtained from **10j** (388 mg, 1 mmol) following general procedure B. Yield: 388 mg (100%) of **11j** as a white solid. ^1^H NMR (400 MHz, DMSO-*d*_*6*_) δ 9.27 − 9.16 (m, 1H), 8.03 (d, *J* = 8.5 Hz, 1H), 7.62 − 7.59 (m, 1H), 7.38 − 7.33 (m, 1H), 7.06 (d, *J* = 8.9 Hz, 1H), 4.74 − 4.63 (m, 2H), 4.38 − 4.31 (m, 1H), 4.28 − 4.19 (m, 2H), 1.68 − 1.56 (m, 5H), 1.50 − 1.43 (m, 2H), 1.21 − 1.04 (m, 4H), 0.91 − 0.75 (m, 2H). ESI-MS [M—H]^−^ = 386.3. HPLC t_*R*_ = 9.32 min.

#### 2-(2,4-Dichlorophenoxy)-*N*-(2-(hydrazinecarbonyl)-2,3-dihydro-1*H*-inden-2-yl)­acetamide (**11k**)

Obtained from **10k** (394 mg, 1 mmol) following general procedure B. The crude product was used without further purification for the next step. ESI-MS [M + Na]^+^ = 416.3.

#### (*S*)-1-(2-(2,4-Dichlorophenoxy)­acetyl)­pyrrolidine-2-carbohydrazide (**11l**)

Obtained from **10l** (332 mg, 1 mmol) following general procedure B. Yield: 332 mg (100%) of **11l** as a white solid. ^1^H NMR (400 MHz, DMSO-*d*_*6*_) δ 9.11 (s, 1H), 7.65 − 7.58 (m, 1H), 7.39 − 7.33 (m, 1H), 7.15 (d, *J* = 9.0 Hz, 1H), 5.06 − 4.93 (m, 2H), 4.36 − 4.28 (m, 1H), 4.29 − 4.12 (m, 2H), 3.69 − 3.61 (m, 1H), 3.59 − 3.51 (m, 1H), 2.12 − 2.03 (m, 1H), 2.00 − 1.92 (m, 2H), 1.90 − 1.83 (m, 1H). ESI-MS [M + Na]^+^ = 354.3. HPLC t_*R*_ = 5.58 min.

#### (1*R*,2*S*,5*S*)-3-(2-(2,4-Dichlorophenoxy)­acetyl)-6,6-dimethyl-3-azabicyclo*[3.1.0]*hexane-2-carbohydrazide (**11m**)

Obtained from **10m** (372 mg, 1 mmol) following general procedure B. Yield: 372 mg (100%) of **11m** as a white solid. ^1^H NMR (400 MHz, DMSO-*d*_*6*_) δ 9.14 (s, 1H), 7.57 − 7.54 (m, 1H), 7.34 − 7.30 (m, 1H), 7.04 − 6.88 (m, 1H), 5.02 − 4.77 (m, 2H), 4.26 − 4.14 (m, 2H), 4.10 (s, 1H), 3.82 (dd, *J* = 10.5, 5.4 Hz, 1H), 3.56 − 3.46 (m, 1H), 1.53 − 1.42 (m, 1H), 1.30 (d, *J* = 7.6 Hz, 1H), 1.01 (s, 3H), 0.88 (s, 3H). ESI-MS [M + Na]^+^ = 396.1. HPLC t_*R*_ = 7.68 min.

#### (*S*)-1-(2-(2,4-Dichlorophenoxy)­acetyl)­indoline-2-carbohydrazide (**11n**)

Obtained from **10n** (380 mg, 1 mmol) following general procedure B. Yield: 380 mg (100%) of **11n** as a white solid. ^1^H NMR (400 MHz, DMSO-*d*_*6*_) δ 8.00 (d, *J* = 7.9 Hz, 1H), 7.59 (d, *J* = 2.4 Hz, 1H), 7.36 − 7.29 (m, 1H), 7.28 − 7.13 (m, 3H), 7.13 − 7.07 (m, 1H), 7.07 − 7.01 (m, 1H), 5.21 − 5.12 (m, 1H), 5.11 − 5.04 (m, 1H), 4.68 − 4.58 (m, 1H), 3.63 − 3.54 (m, 2H), 3.09 (s, 2H). ESI-MS [M + Na]^+^ = 402.3. HPLC t_*R*_ = 7.16 min.

#### (2*S*,3*aS*,7*aS*)-1-(2-(2,4-Dichlorophenoxy)­acetyl)­octahydro-1*H*-indole-2-carbohydrazide (**11o**)

Obtained from **10o** (386 mg, 1 mmol) following general procedure B. The crude product was used without further purification for the next step. ESI-MS [M + Na]^+^ = 408.4.

#### (*S*)-1-(2-(2,4-Dichlorophenoxy)­acetyl)­piperidine-2-carbohydrazide (**11p**)

Obtained from **10p** (346 mg, 1 mmol) following general procedure B. Yield: 346 mg (100%) of **11p** as a white solid. ^1^H NMR (400 MHz, DMSO-*d*_*6*_) δ 9.32 − 8.99 (m, 1H), 7.57 − 7.53 (m, 1H), 7.36 − 7.30 (m, 1H), 7.16 − 7.05 (m, 1H), 5.17 − 5.07 (m, 1H), 5.02 − 4.95 (m, 1H), 4.94 − 4.89 (m, 1H), 4.32 − 4.13 (m, 2H), 3.70 − 3.59 (m, 1H), 3.22 − 3.10 (m, 1H), 2.22 − 2.12 (m, 1H), 1.64 − 1.56 (m, 2H), 1.52 − 1.36 (m, 2H), 1.33 − 1.23 (m, 1H). ESI-MS [M + Na]^+^ = 360.0. HPLC t_*R*_ = 7.32 min.

#### (*S*)-2-(2-(2,4-Dichlorophenoxy)­acetyl)-1,2,3,4-tetrahydroisoquinoline-3-carbohydrazide (**11q**)

Obtained from **10q** (394 mg, 1 mmol) following general procedure B. Yield: 394 mg (100%) of **11q** as a white solid. ^1^H NMR (400 MHz, DMSO-*d*_*6*_) δ 9.08 (s, 1H), 7.57 (d, *J* = 2.5 Hz, 1H), 7.36 − 7.30 (m, 1H), 7.24 − 7.19 (m, 3H), 7.19 − 7.17 (m, 2H), 7.17 − 7.08 (m, 1H), 5.26 − 5.15 (m, 2H), 5.04 − 4.98 (m, 1H), 4.84 − 4.77 (m, 1H), 4.76 − 4.61 (m, 2H), 3.26 − 3.19 (m, 1H), 3.03 − 2.95 (m, 1H). ESI-MS [M + Na]^+^ = 416.3. HPLC t_*R*_ = 7.49 min.

#### (1*S*,3*aR*,6*aS*)-2-(2-(2,4-Dichlorophenoxy)­acetyl)­octahydrocyclopenta*[c]*pyrrole-1-carbohydrazide (**11r**)

Obtained from **10r** (386 mg, 1 mmol) following general procedure B. Yield: 372 mg (100%) of **11r** as a white solid. ^1^H NMR (400 MHz, DMSO-*d*_*6*_) δ 9.12 (s, 1H), 7.59 − 7.54 (m, 1H), 7.38 − 7.28 (m, 1H), 7.11 − 6.94 (m, 1H), 5.06 − 4.82 (m, 2H), 4.28 − 4.14 (m, 2H), 4.10 − 4.04 (m, 1H), 3.81 − 3.69 (m, 1H), 3.42 − 3.36 (m, 1H), 2.77 − 2.66 (m, 1H), 2.49 − 2.38 (m, 1H), 1.85 − 1.73 (m, 2H), 1.69 − 1.63 (m, 1H), 1.56 − 1.45 (m, 3H). ESI-MS [M + Na]^+^ = 396.0. HPLC t_*R*_ = 7.36 min.

#### (*S*)-2-(2,4-Dichlorophenoxy)-*N*-(4-fluoro-4-methyl-1-oxo-1-(2-((2-oxo-1,2-dihydropyridin-3-yl)­methylene)­hydrazineyl)­pentan-2-yl)­acetamide (**12a**)

Obtained from the reaction of **11a** (340 mg, 1 mmol) and 2-oxo-1.2-dihydropyridine-3-carbaldehyde (123 mg, 1 mmol) following general procedure C. The crude product was obtained as an E/Z isomer mixture and used for the next step without further purification.

#### (*S*)-2-(2,4-Dichlorophenoxy)-*N*-(4,4-dimethyl-1-oxo-1-(2-((2-oxo-1,2-dihydropyridin-3-yl)­methylene)­hydrazineyl)­pentan-2-yl)­acetamide (**12b**)

Obtained from the reaction of **11b** 34i mg, 1 mmol) and 2-oxo-1.2-dihydropyridine-3-carbaldehyde (123 mg, 1 mmol) following general procedure C. The crude product was obtained as an E/Z isomer mixture and used for the next step without further purification.

#### (*S*)-*N*-(3-Cyclopropyl-1-oxo-1-(2-((2-oxo-1,2-dihydropyridin-3-yl)­methylene)­hydrazineyl)­propan-2-yl)-2-(2,4-dichlorophenoxy)­acetamide (**12c**)

Obtained from the reaction of **11c** (345 mg, 1 mmol) and 2-oxo-1.2-dihydropyridine-3-carbaldehyde (123 mg, 1 mmol) following general procedure C. The crude product was obtained as an E/Z isomer mixture and used for the next step without further purification.

#### (*S*)-2-(2,4-Dichlorophenoxy)-*N*-(1-oxo-1-(2-((2-oxo-1,2-dihydropyridin-3-yl) methylene)­hydrazineyl)-3-phenylpropan-2-yl)­acetamide (**12d**)

Obtained from the reaction of **11d** (381 mg, 1 mmol) and 2-oxo-1.2-dihydropyridine-3-carbaldehyde (123 mg, 1 mmol) following general procedure C. The crude product was obtained as an E/Z isomer mixture and used for the next step without further purification.

#### (*S*)-2-(2,4-Dichlorophenoxy)-*N*-(3-(2-fluorophenyl)-1-oxo-1-(2-((2-oxo-1,2-dihydropyridin-3-yl)­methylene)­hydrazineyl)­propan-2-yl)­acetamide (**12e**)

Obtained from the reaction of **11e** (399 mg, 1 mmol) and 2-oxo-1.2-dihydropyridine-3-carbaldehyde (123 mg, 1 mmol) following general procedure C. The crude product was obtained as an E/Z isomer mixture and used for the next step without further purification.

#### (*S*)-2-(2,4-Dichlorophenoxy)-*N*-(3-(3-fluorophenyl)-1-oxo-1-(2-((2-oxo-1,2-dihydropyridin-3-yl)­methylene)­hydrazineyl)­propan-2-yl)­acetamide (**12f**)

Obtained from the reaction of **11f** (399 mg, 1 mmol) and 2-oxo-1.2-dihydropyridine-3-carbaldehyde (123 mg, 1 mmol) following general procedure C. The crude product was obtained as an E/Z isomer mixture and used for the next step without further purification.

#### (*S*)-2-(2,4-Dichlorophenoxy)-*N*-(3-(4-fluorophenyl)-1-oxo-1-(2-((2-oxo-1,2-dihydropyridin-3-yl)­methylene)­hydrazineyl)­propan-2-yl)­acetamide (**12g**)

Obtained from the reaction of **11g** (399 mg, 1 mmol) and 2-oxo-1,2-dihydropyridine-3-carbaldehyde (123 mg, 1 mmol) following general procedure C. The crude product was obtained as an E/Z isomer mixture and used for the next step without further purification.

#### (*S*)-2-(2,4-Dichlorophenoxy)-*N*-(3-(3,5-difluorophenyl)-1-oxo-1-(2-((2-oxo-1,2-dihydropyridin-3-yl)­methylene)­hydrazineyl)­propan-2-yl)­acetamide (**12h**)

Obtained from the reaction of **11h** (417 mg, 1 mmol) and 2-oxo-1.2-dihydropyridine-3-carbaldehyde (123 mg, 1 mmol) following general procedure C. The crude product was obtained as an E/Z isomer mixture and used for the next step without further purification.

#### (*S*)-2-(2,4-Dichlorophenoxy)-*N*-(3-(3-chlorophenyl)-1-oxo-1-(2-((2-oxo-1,2-dihydropyridin-3-yl)­methylene)­hydrazineyl)­propan-2-yl)­acetamide (**12i**)

Obtained from the reaction of **11i** (mg, 1 mmol) and 2-oxo-1.2-dihydropyridine-3-carbaldehyde (123 mg, 1 mmol) following general procedure C. The crude product was obtained as an E/Z isomer mixture and used for the next step without further purification.

#### (*S*)-*N*-(3-Cyclohexyl-1-oxo-1-(2-((2-oxo-1,2-dihydropyridin-3-yl)­methylene) hydrazineyl)­propan-2-yl)-2-(2,4-dichlorophenoxy) acetamide (**12j**)

Obtained from the reaction of **11j** (387 mg, 1 mmol) and 2-oxo-1.2-dihydropyridine-3-carbaldehyde (123 mg, 1 mmol) following general procedure C. The crude product was obtained as an E/Z isomer mixture and used for the next step without further purification.

#### 2-(2,4-Dichlorophenoxy)-*N*-(2-(2-((2-oxo-1,2-dihydropyridin-3-yl)­methylene)­hydrazine-1-carbonyl)-2,3-dihydro-1*H*-inden-2-yl) acetamide (**12k**)

Obtained from the reaction of **11k** (393 mg, 1 mmol) and 2-oxo-1.2-dihydropyridine-3-carbaldehyde (123 mg, 1 mmol) following general procedure C. The crude product was obtained as an E/Z isomer mixture and used for the next step without further purification.

#### (*S*)-1-(2-(2,4-Dichlorophenoxy)­acetyl)-*N’*-((2-Oxo-1,2-dihydropyridin-3-yl)­methylene)­pyrrolidine-2-carbohydrazide (**12l**)

Obtained from the reaction of **11l** (331 mg, 1 mmol) and 2-oxo-1.2-dihydropyridine-3-carbaldehyde (123 mg, 1 mmol) following general procedure C. The crude product was obtained as an E/Z isomer mixture and used for the next step without further purification.

#### (1*R*,2*S*,5*S*)-3-(2-(2,4-Dichlorophenoxy)­acetyl)-6,6-dimethyl-*N’*-((2-oxo-1,2-dihydropyridin-3-yl)­methylene)-3-azabicyclo*[3.1.0]*hexane-2-carbohydrazid (**12m**)

Obtained from the reaction of **11m** (371 mg, 1 mmol) and 2-oxo-1.2-dihydropyridine-3-carbaldehyde (123 mg, 1 mmol) following general procedure C. The crude product was obtained as an E/Z isomer mixture and used for the next step without further purification.

#### (*S*)-1-(2-(2,4-Dichlorophenoxy)­acetyl)-*N’*-((2-oxo-1,2-dihydropyridin-3-yl)­methylene)­indoline-2-carbohydrazide (**12n**)

Obtained from the reaction of **11n** (379 mg, 1 mmol) and 2-oxo-1.2-dihydropyridine-3-carbaldehyde (123 mg, 1 mmol) following general procedure C. The crude product was obtained as an E/Z isomer mixture and used for the next step without further purification.

#### (2*S*,3*aS*,7*aS*)-1-(2-(2,4-Dichlorophenoxy)­acetyl)-*N’*-((2-oxo-1,2-dihydropyridin-3-yl) methylene)­octahydro-1*H*-indole-2-carbohydrazide (**12o**)

Obtained from the reaction of **11o** (385 mg, 1 mmol) and 2-oxo-1.2-dihydropyridine-3-carbaldehyde (123 mg, 1 mmol) following general procedure C. The crude product was obtained as an E/Z isomer mixture and used for the next step without further purification.

#### (*S*)-1-(2-(2,4-Dichlorophenoxy)­acetyl)-*N’*-((2-oxo-1,2-dihydropyridin-3-yl)­methylene)­piperidine-2-carbohydrazide (**12p**)

Obtained from the reaction of **11p** (345 mg, 1 mmol) and 2-oxo-1.2-dihydropyridine-3-carbaldehyde (123 mg, 1 mmol) following general procedure C. The crude product was obtained as an E/Z isomer mixture and used for the next step without further purification.

#### (*S*)-2-(2-(2,4-Dichlorophenoxy)­acetyl)-*N’*-((2-oxo-1,2-dihydropyridin-3-yl)­methylene)-1,2,3,4-tetrahydroisoquinoline-3-carbohydrazide (**12q**)

Obtained from the reaction of **11p** (393 mg, 1 mmol) and 2-oxo-1.2-dihydropyridine-3-carbaldehyde (123 mg, 1 mmol) following general procedure C. The crude product was obtained as an E/Z isomer mixture and used for the next step without further purification.

#### (1*S*,3*aR*,6*aS*)-2-(2-(2,4-Dichlorophenoxy)­acetyl)-*N’*-((2-oxo-1,2-dihydropyridin-3-yl) methylene)­octahydrocyclopenta*[c]*pyrrole-1-carbohydrazide (**12r**)

Obtained from the reaction of **11r** (371 mg, 1 mmol) and 2-oxo-1.2-dihydropyridine-3-carbaldehyde (123 mg, 1 mmol) following general procedure C. The crude product was obtained as an E/Z isomer mixture and used for the next step without further purification.

#### (1*S*,3*aR*,6*aS*)-*N’*-(-(5-Chloro-2-oxo-1,2-dihydropyridin-3-yl)­methylene)-2-(2-(2,4-dichlorophenoxy)­acetyl)­octahydrocyclopenta*[c]*pyrrole-1-carbohydrazide (**12s**)

Obtained from the reaction of **11r** (372 mg, 1 mmol) and 5-chloro-2-oxo-1,2-dihydropyridine-3-carbaldehyde (157 mg, 1 mmol) following general procedure C. The crude product was obtained as an E/Z isomer mixture and used for the next step without further purification.

#### (*S*)-2-(2,4-Dichlorophenoxy)-*N*-(4-fluoro-4-methyl-1-oxo-1-(2-((2-oxo-1,2-dihydropyridin-3-yl)­methyl)­hydrazineyl)­pentan-2-yl) acetamide (**13a**)

Obtained from **12a** (366 mg, 1 mmol) following general procedure D. Flash purification with CH_2_Cl_2_/CH_3_OH (0 − 7% CH_3_OH). Yield over 2 steps: 114 mg (24%) of **13a** as a white solid. ^1^H NMR (400 MHz, DMSO-*d*_*6*_) δ 11.56 (s, 1H), 9.72 − 9.48 (m, 1H), 8.16 (d, *J* = 8.4 Hz, 1H), 7.60 (d, *J* = 2.6 Hz, 1H), 7.39 − 7.33 (m, 2H), 7.29 − 7.24 (m, 1H), 7.05 (d, *J* = 8.9 Hz, 1H), 6.12 (t, *J* = 6.6 Hz, 1H), 5.43 − 5.19 (m, 1H), 4.67 (s, 2H), 4.50 − 4.34 (m, 1H), 3.70 − 3.53 (m, 2H), 2.07 − 1.95 (m, 1H), 1.95 − 1.80 (m, 1H), 1.32 (d, *J* = 4.9 Hz, 3H), 1.27 (d, *J* = 5.0 Hz, 3H). ESI-MS [M—H]^−^ = 470.8. HPLC t_*R*_ = 7.75 min.

#### (*S*)-2-(2,4-Dichlorophenoxy)-*N*-(4,4-dimethyl-1-oxo-1-(2-((2-oxo-1,2-dihydropyridin-3-yl)­methyl)­hydrazineyl)­pentan-2-yl)­acetamide (**13b**)

Obtained from **12b** (362 mg, 1 mmol) following general procedure D. Flash purification with CH_2_Cl_2_/CH_3_OH (0 − 7% CH_3_OH). Yield over 2 steps: 230 mg (49%) of **13b** as a white solid. ^1^H NMR (400 MHz, DMSO-*d*_*6*_) δ 11.55 (s, 1H), 9.66 − 9.54 (m, 1H), 8.07 (d, *J* = 8.6 Hz, 1H), 7.60 (d, *J* = 2.6 Hz, 1H), 7.42 − 7.31 (m, 2H), 7.29 − 7.23 (m, 1H), 7.04 (d, *J* = 8.9 Hz, 1H), 6.11 (t, *J* = 6.6 Hz, 1H), 5.36 − 5.23 (m, 1H), 4.72 − 4.58 (m, 2H), 4.36 − 4.24 (m, 1H), 3.72 − 3.55 (m, 2H), 1.56 (dd, *J* = 14.2, 4.2 Hz, 1H), 1.44 (dd, *J* = 14.2, 8.4 Hz, 1H), 0.86 (s, 9H). ESI-MS [M—H]^−^ = 466.8. HPLC t_*R*_ = 8.81 min.

#### (*S*)-*N*-(3-Cyclopropyl-1-oxo-1-(2-((2-oxo-1,2-dihydropyridin-3-yl)­methyl)­hydrazineyl) propan-2-yl)-2-(2,4-dichlorophenoxy)­acetamide (**13c**)

Obtained from **12c** (451 mg, 1 mmol) following general procedure D. Flash purification with CH_2_Cl_2_/CH_3_OH (0 − 7% CH_3_OH). Yield over 2 steps: 132 mg (29%) of **13c** as a white solid. ^1^H NMR (400 MHz, DMSO-*d*_*6*_) δ 11.65 − 11.42 (m, 1H), 9.66 − 9.46 (m, 1H), 8.11 − 7.97 (m, 1H), 7.62 − 7.51 (m, 1H), 7.38 − 7.29 (m, 2H), 7.26 − 7.19 (m, 1H), 7.10 − 6.99 (m, 1H), 6.16 − 6.01 (m, 1H), 5.37 − 5.23 (m, 1H), 4.72 − 4.60 (m, 2H), 4.34 − 4.24 (m, 1H), 3.69 − 3.52 (m, 2H), 1.54 − 1.44 (m, 1H), 1.42 − 1.33 (m, 1H), 0.60 − 0.51 (m, 1H), 0.37 − 0.25 (m, 2H), 0.07 − −0.08 (m, 2H). ESI-MS [M—H]^−^ = 451.5. HPLC t_*R*_ = 7.57 min.

#### (*S*)-2-(2,4-Dichlorophenoxy)-*N*-(1-oxo-1-(2-((2-oxo-1,2-dihydropyridin-3-yl)­methyl) hydrazineyl)-3-phenylpropan-2-yl)­acetamide (**13d**)

Obtained from **12d** (487 mg, 1 mmol) following general procedure D. Flash purification with CH_2_Cl_2_/CH_3_OH (0 − 7% CH_3_OH). Yield over 2 steps: 254 mg (52%) of **13d** as a white solid. ^1^H NMR (400 MHz, DMSO-*d*_*6*_) δ 8.63 − 8.29 (m, 1H), 7.56 (d, *J* = 2.0 Hz, 1H), 7.52 − 7.45 (m, 1H), 7.40 − 7.34 (m, 1H), 7.29 − 7.19 (m, 7H), 7.12 (d, *J* = 7.8 Hz, 1H), 6.85 − 6.78 (m, 1H), 6.18 − 6.05 (m, 1H), 4.71 − 4.43 (m, 4H), 3.69 − 3.60 (m, 2H), 2.97 (dd, *J* = 13.6, 4.4 Hz, 1H), 2.79 (dd, *J* = 13.4, 9.8 Hz, 1H). ESI-MS [M—H]^−^ = 487.3. HPLC t_*R*_ = 8.52 min.

#### (*S*)-2-(2,4-Dichlorophenoxy)-*N*-(3-(2-fluorophenyl)-1-oxo-1-(2-((2-oxo-1,2-dihydropyridin-3-yl)­methyl)­hydrazineyl)­propan-2-yl) acetamide (**13e**)

Obtained from **12e** (505 mg, 1 mmol) following general procedure D. Flash purification with CH_2_Cl_2_/CH_3_OH (0 − 7% CH_3_OH). Yield over 2 steps: 416 mg (82%) of **13e** as a white solid. ^1^H NMR (400 MHz, DMSO-*d*_*6*_) δ 11.57 (s, 1H), 9.74 − 9.58 (m, 1H), 8.17 (d, *J* = 8.7 Hz, 1H), 7.58 (d, *J* = 2.6 Hz, 1H), 7.37 − 7.30 (m, 1H), 7.30 − 7.19 (m, 4H), 7.14 − 7.03 (m, 2H), 6.80 (d, *J* = 8.9 Hz, 1H), 6.12 (t, *J* = 6.6 Hz, 1H), 5.38 − 5.26 (m, 1H), 4.65 − 4.52 (m, 3H), 3.67 − 3.54 (m, 2H), 3.03 (dd, *J* = 13.8, 5.4 Hz, 1H), 2.82 (dd, *J* = 13.8, 9.0 Hz, 1H). ESI-MS [M—H]^−^ = 504.8. HPLC t_*R*_ = 8.30 min.

#### (*S*)-2-(2,4-Dichlorophenoxy)-*N*-(3-(3-fluorophenyl)-1-oxo-1-(2-((2-oxo-1,2-dihydropyridin-3-yl)­methyl)­hydrazineyl)­propan-2-yl) acetamide (**13f**)

Obtained from **12f** (505 mg, 1 mmol) following general procedure D. Flash purification with CH_2_Cl_2_/CH_3_OH (0 − 7% CH_3_OH). Yield over 2 steps: 254 mg (50%) of **13f** as a white solid. ^1^H NMR (400 MHz, DMSO-*d*_*6*_) δ 11.56 (s, 1H), 9.71 − 9.51 (m, 1H), 8.20 (d, *J* = 8.6 Hz, 1H), 7.57 (d, *J* = 2.6 Hz, 1H), 7.37 − 7.18 (m, 4H), 7.08 − 6.95 (m, 3H), 6.79 (d, *J* = 8.9 Hz, 1H), 6.16 − 6.06 (m, 1H), 5.40 − 5.24 (m, 1H), 4.65 − 4.55 (m, 2H), 4.58 − 4.45 (m, 1H), 3.70 − 3.56 (m, 2H), 3.03 − 2.91 (m, 1H), 2.86 − 2.76 (m, 1H). ESI-MS [M—H]^−^ = 505.5. HPLC t_*R*_ = 8.12 min.

#### (*S*)-2-(2,4-Dichlorophenoxy)-*N*-(3-(4-fluorophenyl)-1-oxo-1-(2-((2-oxo-1,2-dihydropyridin-3-yl)­methyl)­hydrazineyl)­propan-2-yl) acetamide (**13g**)

Obtained from **12g** (505 mg, 1 mmol) following general procedure D. Flash purification with CH_2_Cl_2_/CH_3_OH (0 − 7% CH_3_OH). Yield over 2 steps: 462 mg (91%) of **13g** as a white solid. ^1^H NMR (400 MHz, DMSO-*d*_*6*_) δ 11.56 (s, 1H), 9.69 − 9.49 (m, 1H), 8.15 (d, *J* = 8.5 Hz, 1H), 7.60 − 7.54 (m, 1H), 7.38 − 7.30 (m, 1H), 7.28 − 7.22 (m, 2H), 7.21 − 7.16 (m, 2H), 7.10 − 6.99 (m, 2H), 6.79 (d, *J* = 8.9 Hz, 1H), 6.16 − 6.08 (m, 1H), 5.39 − 5.25 (m, 1H), 4.66 − 4.54 (m, 2H), 4.52 − 4.42 (m, 1H), 3.70 − 3.55 (m, 2H), 2.93 (dd, *J* = 13.7, 5.1 Hz, 1H), 2.76 (dd, *J* = 13.7, 9.1 Hz, 1H). ESI-MS [M—H]^−^ = 505.5. HPLC t_*R*_ = 8.19 min.

#### (*S*)-2-(2,4-Dichlorophenoxy)-*N*-(3-(3,5-difluorophenyl)-1-oxo-1-(2-((2-oxo-1,2-dihydropyridin-3-yl)­methyl)­hydrazineyl)­propan-2-yl) Acetamide (**13h**)

Obtained from **12h** (523 mg, 1 mmol) following general procedure D. Flash purification with CH_2_Cl_2_/CH_3_OH (0 − 7% CH_3_OH). Yield over 2 steps: 499 mg (95%) of **13h** as a white solid. ^1^H NMR (400 MHz, DMSO-*d*_*6*_) δ 11.56 (s, 1H), 9.68 − 9.52 (m, 1H), 8.21 (d, *J* = 8.6 Hz, 1H), 7.59 − 7.55 (m, 1H), 7.38 − 7.33 (m, 1H), 7.30 − 7.25 (m, 1H), 7.25 − 7.21 (m, 1H), 7.09 − 7.01 (m, 1H), 6.95 − 6.88 (m, 2H), 6.83 (d, *J* = 8.9 Hz, 1H), 6.15 − 6.09 (m, 1H), 5.39 − 5.29 (m, 1H), 4.65 − 4.56 (m, 2H), 4.56 − 4.49 (m, 1H), 3.66 − 3.59 (m, 2H), 2.98 (dd, *J* = 13.8, 4.9 Hz, 1H), 2.84 (dd, *J* = 13.7, 9.5 Hz, 1H). ESI-MS [M—H]^−^ = 523.5. HPLC t_*R*_ = 8.41 min.

#### (*S*)-2-(2,4-Dichlorophenoxy)-*N*-(3-(3-chlorophenyl)-1-oxo-1-(2-((2-oxo-1,2-dihydropyridin-3-yl)­methyl)­hydrazineyl)­propan-2-yl)­acetamide (**13i**)

Obtained from **12i** (417 mg, 1 mmol) following general procedure D. Flash purification with CH_2_Cl_2_/CH_3_OH (0 − 7% CH_3_OH). Yield over 2 steps: 356 mg (68%) of **13i** as a white solid. ^1^H NMR (400 MHz, DMSO-*d*_*6*_) δ 11.57 (s, 1H), 9.70 − 9.53 (m, 1H), 8.23 (d, *J* = 8.6 Hz, 1H), 7.58 (d, *J* = 2.6 Hz, 1H), 7.38 − 7.32 (m, 1H), 7.32 − 7.26 (m, 4H), 7.26 − 7.22 (m, 1H), 7.21 − 7.10 (m, 1H), 6.78 (d, *J* = 8.9 Hz, 1H), 6.13 (t, *J* = 6.6 Hz, 1H), 5.42 − 5.24 (m, 1H), 4.66 − 4.56 (m, 2H), 4.56 − 4.48 (m, 1H), 3.67 − 3.54 (m, 2H), 2.96 (dd, *J* = 13.7, 4.9 Hz, 1H), 2.80 (dd, *J* = 13.7, 9.5 Hz, 1H). ESI-MS [M—H]^−^ = 520.7. HPLC t_*R*_ = 8.90 min.

#### (*S*)-*N*-(3-Cyclohexyl-1-oxo-1-(2-((2-oxo-1,2-dihydropyridin-3-yl)­methyl)­hydrazineyl) propan-2-yl)-2-(2,4-dichlorophenoxy)­acetamide (**13j**)

Obtained from **12j** (493 mg, 1 mmol) following general procedure D. Flash purification with CH_2_Cl_2_/CH_3_OH (0 − 7% CH_3_OH). Yield over 2 steps: 272 mg (55%) of **13j** as a white solid. ^1^H NMR (400 MHz, DMSO-*d*_*6*_) δ 11.61 − 11.51 (m, 1H), 9.60 − 9.52 (m, 1H), 8.02 (d, *J* = 8.4 Hz, 1H), 7.62 − 7.58 (m, 1H), 7.39 − 7.33 (m, 2H), 7.28 − 7.24 (m, 1H), 7.07 − 7.02 (m, 1H), 6.14 − 6.09 (m, 1H), 5.36 − 5.29 (m, 1H), 4.73 − 4.63 (m, 2H), 4.33 − 4.26 (m, 1H), 3.67 − 3.57 (m, 2H), 1.64 − 1.56 (m, 5H), 1.43 − 1.36 (m, 2H), 1.15 − 1.03 (m, 4H), 0.87 − 0.75 (m, 2H). ESI-MS [M—H]^−^ = 493.5. HPLC t_*R*_ = 9.19 min.

#### 2-(2,4-Dichlorophenoxy)-*N*-(2-(2-((2-oxo-1,2-dihydropyridin-3-yl)­methyl)­hydrazine-1-carbonyl)-2,3-dihydro-1*H*-inden-2-yl)­acetamide *(13k)*

Obtained from **12k** (499 mg, 1 mmol) following general procedure D. Flash purification with CH_2_Cl_2_/CH_3_OH (0 − 7.1% CH_3_OH). Yield over 2 steps: 175 mg (35%) of **13k** as a white solid. ^1^H NMR (400 MHz, DMSO-*d*_*6*_) δ 11.54 (s, 1H), 9.58 − 9.40 (m, 1H), 8.39 − 8.25 (m, 1H), 7.54 (d, *J* = 2.6 Hz, 1H), 7.38 − 7.32 (m, 1H), 7.30 − 7.24 (m, 2H), 7.23 − 7.13 (m, 4H), 7.00 (d, *J* = 9.0 Hz, 1H), 6.20 − 6.02 (m, 1H), 5.32 − 5.13 (m, 1H), 4.63 (s, 2H), 3.61 (s, 2H), 3.45 (d, *J* = 16.7 Hz, 2H), 3.16 (d, *J* = 16.7 Hz, 2H). ESI-MS [M—H]^−^ = 499.6. HPLC t_*R*_ = 8.23 min.

#### (*S*)-1-(2-(2,4-Dichlorophenoxy)­acetyl)-*N’*-((2-oxo-1,2-dihydropyridin-3-yl)­methyl)­pyrrolidine-2-carbohydrazide (**13l**)

Obtained from **12l** (437 mg, 1 mmol) following general procedure D. Flash purification with CH_2_Cl_2_/CH_3_OH (0 − 8% CH_3_OH). Yield over 2 steps: 268 mg (61%) of **13l** as an off white solid. ^1^H NMR (400 MHz, DMSO-*d*_*6*_) δ 11.53 (s, 1H), 9.46 − 9.26 (m, 1H), 7.57 − 7.54 (m, 1H), 7.38 − 7.33 (m, 1H), 7.33 − 7.29 (m, 1H), 7.28 − 7.23 (m, 1H), 7.09 (d, *J* = 9.0 Hz, 1H), 6.13 − 6.05 (m, 1H), 5.29 − 5.17 (m, 1H), 5.02 − 4.86 (m, 2H), 4.36 − 4.17 (m, 1H), 3.71 − 3.62 (m, 1H), 3.62 − 3.56 (m, 2H), 3.53 − 3.42 (m, 1H), 2.03 − 1.94 (m, 1H), 1.92 − 1.83 (m, 2H), 1.77 − 1.69 (m, 1H). ESI-MS [M—H]^−^ = 437.5. HPLC t_*R*_ = 5.89 min.

#### (1*R*,2*S*,5*S*)-3-(2-(2,4-Dichlorophenoxy)­acetyl)-6,6-dimethyl-*N’*-((2-oxo-1,2-dihydropyridin-3-yl)­methyl)-3-azabicyclo*[3.1.0]*hexane-2-carbohydrazide (**13m**)

Obtained from **12m** (477 mg, 1 mmol) following general procedure D. Flash purification with CH_2_Cl_2_/CH_3_OH (0 − 8% CH_3_OH). Yield over 2 steps: 149 mg (31%) of **13m** as a white solid. ^1^H NMR (400 MHz, DMSO-*d*_*6*_) δ 11.56 (s, 1H), 9.77 − 9.33 (m, 1H), 7.61 − 7.50 (m, 1H), 7.42 − 7.16 (m, 3H), 7.07 − 6.79 (m, 1H), 6.17 − 6.02 (m, 1H), 5.48 − 5.21 (m, 1H), 5.10 − 4.91 (m, 1H), 4.88 − 4.75 (m, 1H), 4.29 − 4.02 (m, 1H), 3.85 − 3.74 (m, 1H), 3.70 − 3.44 (m, 3H), 1.52 − 1.37 (m, 1H), 1.25 − 1.15 (m, 1H), 0.99 (s, 3H), 0.88 (s, 3H). ESI-MS [M + Na]^+^ = 501.0. HPLC t_*R*_ = 8.150 min.

#### (*S*)-1-(2-(2,4-Dichlorophenoxy)­acetyl)-*N’*-((2-oxo-1,2-dihydropyridin-3-yl)­methyl)­indoline-2-carbohydrazide (**13n**)

Obtained from **12n** (485 mg, 1 mmol) following general procedure D. Flash purification with CH_2_Cl_2_/CH_3_OH (0 − 7% CH_3_OH). Yield over 2 steps: 244 mg (50%) of **13n** as a white solid. ^1^H NMR (400 MHz, DMSO-*d*_*6*_) δ 11.56 (s, 1H), 10.09 − 9.53 (m, 1H), 8.10 − 7.92 (m, 1H), 7.63 − 7.54 (m, 1H), 7.39 − 7.28 (m, 2H), 7.28 − 7.15 (m, 3H), 7.08 − 6.96 (m, 2H), 6.17 − 6.00 (m, 1H), 5.61 − 5.27 (m, 1H), 5.26 − 4.98 (m, 2H), 4.60 − 4.33 (m, 1H), 3.81 − 3.50 (m, 3H), 3.13 − 3.01 (m, 1H). ESI-MS [M—H]^−^ = 485.5. HPLC t_*R*_ = 7.13 min.

#### (2*S*,3*aS*,7*aS*)-1-(2-(2,4-Dichlorophenoxy)­acetyl)-*N’*-((2-oxo-1,2-dihydropyridin-3-yl) methyl)­octahydro-1*H*-indole-2-carbohydrazide (**13o**)

Obtained from **12o** (491 mg, 1 mmol) following general procedure D. Flash purification with CH_2_Cl_2_/CH_3_OH (0 − 7.1% CH_3_OH). Yield over 2 steps: 212 mg (43%) of **13o** as a white solid. ^1^H NMR (400 MHz, DMSO-*d*_*6*_) δ 11.52 (s, 1H), 9.66 − 9.28 (m, 1H), 7.58 − 7.53 (m, 1H), 7.38 − 7.34 (m, 1H), 7.34 − 7.28 (m, 1H), 7.28 − 7.14 (m, 1H), 7.11 − 7.04 (m, 1H), 6.19 − 5.98 (m, 1H), 5.29 − 5.17 (m, 1H), 5.12 − 4.99 (m, 1H), 4.86 − 4.69 (m, 1H), 4.36 − 4.13 (m, 1H), 4.03 − 3.84 (m, 1H), 3.69 − 3.50 (m, 2H), 2.32 − 2.16 (m, 1H), 2.00 − 1.85 (m, 2H), 1.77 − 1.56 (m, 4H), 1.48 − 1.38 (m, 1H), 1.31 − 1.04 (m, 3H). ESI-MS [M—H]^−^ = 491.6. HPLC t_*R*_ = 7.87 min.

#### (*S*)-1-(2-(2,4-Dichlorophenoxy)­acetyl)-*N’*-((2-oxo-1,2-dihydropyridin-3-yl)­methyl)­piperidine-2-carbohydrazide (**13p**)

Obtained from **12p** (451 mg, 1 mmol) following general procedure D. Flash purification with CH_2_Cl_2_/CH_3_OH (0 − 8% CH_3_OH). Yield over 2 steps: 129 mg (29%) of **13p** as a white solid. ^1^H NMR (400 MHz, DMSO-*d*_*6*_) δ 11.56 (s, 1H), 9.69 − 9.32 (m, 1H), 7.55 (d, *J* = 2.5 Hz, 1H), 7.39 − 7.30 (m, 2H), 7.29 − 7.22 (m, 1H), 7.14 − 7.00 (m, 1H), 6.14 − 6.06 (m, 1H), 5.50 − 5.36 (m, 1H), 5.16 − 4.91 (m, 2H), 4.89 − 4.83 (m, 1H), 3.74 − 3.58 (m, 3H), 3.19 − 3.08 (m, 1H), 2.16 − 2.03 (m, 1H), 1.65 − 1.51 (m, 2H), 1.49 − 1.31 (m, 2H), 1.27 − 1.19 (m, 1H). ESI-MS [M + Na]^+^ = 475.3. HPLC t_*R*_ = 7.44 min.

#### (*S*)-2-(2-(2,4-Dichlorophenoxy)­acetyl)-*N’*-((2-oxo-1,2-dihydropyridin-3-yl)­methyl)-1,2,3,4-tetrahydroisoquinoline-3-carbohydrazide (**13q**)

Obtained from **12q** (499 mg, 1 mmol) following general procedure D. Flash purification with CH_2_Cl_2_/CH_3_OH (0 − 7% CH_3_OH). Yield over 2 steps: 251 mg (50%) of **13q** as an off white solid. ^1^H NMR (400 MHz, DMSO-*d*_*6*_) δ 11.49 (s, 1H), 9.69 − 9.26 (m, 1H), 7.57 (d, *J* = 2.6 Hz, 1H), 7.36 − 7.30 (m, 1H), 7.25 − 7.19 (m, 3H), 7.19 − 7.14 (m, 2H), 7.12 − 7.03 (m, 1H), 7.03 − 6.96 (m, 1H), 6.01 − 5.94 (m, 1H), 5.33 − 5.07 (m, 3H), 4.97 − 4.91 (m, 1H), 4.77 − 4.58 (m, 2H), 3.52 − 3.32 (m, 2H), 3.16 (dd, *J* = 15.6, 3.4 Hz, 1H), 2.98 (dd, *J* = 15.4, 6.0 Hz, 1H). ESI-MS [M—H]^−^ = 499.6. HPLC t_*R*_ = 7.57 min.

#### (1*S*,3*aR*,6*aS*)-2-(2-(2,4-Dichlorophenoxy)­acetyl)-*N’*-((2-oxo-1,2dihydropyridin-3-yl)­methyl)­octahydrocyclopenta*[c]*pyrrole-1-carbohydrazide (**13r**)

Obtained from **12r** (477 mg, 1 mmol) following general procedure D. Flash purification with CH_2_Cl_2_/CH_3_OH (0 − 8% CH_3_OH). Yield over 2 steps: 192 mg (40%) of **13r** as a white solid. ^1^H NMR (400 MHz, DMSO-*d*_*6*_) δ 11.56 (s, 1H), 9.74 − 9.35 (m, 1H), 7.56 (d, *J* = 2.6 Hz, 1H), 7.41 − 7.18 (m, 3H), 7.11 − 6.85 (m, 1H), 6.16 − 6.01 (m, 1H), 5.47 − 5.16 (m, 1H), 5.09 − 4.97 (m, 1H), 4.93 − 4.79 (m, 1H), 4.11 − 3.97 (m, 1H), 3.79 − 3.48 (m, 3H), 3.45 − 3.35 (m, 1H), 2.73 − 2.55 (m, 1H), 2.45 − 2.29 (m, 1H), 1.94 − 1.73 (m, 2H), 1.71 − 1.61 (m, 1H), 1.58 − 1.38 (m, 3H). ESI-MS [M + Na]^+^ = 501.0. HPLC t_*R*_ = 7.88 min.

#### (1*S*,3*aR*,6*aS*)-*N’*-((5-Chloro-2-oxo-1,2-dihydropyridin-3-yl)­methyl)-2-(2-(2,4-dichlorophenoxy)­acetyl)­octahydrocyclopenta*[c]*pyrrole-1-carbohydrazide (**13s**)

Obtained from **12s** (511 mg, 1 mmol) following general procedure D. Flash purification with CH_2_Cl_2_/CH_3_OH (0 − 6% CH_3_OH). Yield over 2 steps: 136 mg (29%) of **13s** as a white solid. ^1^H NMR (400 MHz, DMSO-*d*_*6*_) δ 11.93 − 11.36 (m, 1H), 9.71 − 9.34 (m, 1H), 7.56 (d, *J* = 2.6 Hz, 1H), 7.51 − 7.43 (m, 1H), 7.43 − 7.37 (m, 1H), 7.35 − 7.29 (m, 1H), 7.06 (d, *J* = 9.0 Hz, 1H), 5.39 − 5.19 (m, 1H), 5.05 − 4.94 (m, 1H), 4.94 − 4.80 (m, 1H), 3.79 − 3.69 (m, 1H), 3.69 − 3.52 (m, 2H), 3.43 − 3.35 (m, 1H), 2.74 − 2.62 (m, 1H), 2.60 − 2.52 (m, 1H), 2.41 − 2.29 (m, 1H), 1.92 − 1.74 (m, 2H), 1.73 − 1.63 (m, 1H), 1.59 − 1.44 (m, 3H). ESI-MS [M—H]^−^ = 510.8. HPLC t_*R*_ = 8.50 min.

#### (*S*)-*N*-(1-(2-(2-Chloroacetyl)-2-((2-oxo-1,2-dihydropyridin-3-yl)­methyl)­hydrazineyl)-4-fluoro-4-methyl-1-oxopentan-2-yl)-2-(2,4-dichlorophenoxy)­acetamide (**14a**)

Following general procedure E for the synthesis of amides, compound **14a** was synthesized using **13a** (100 mg, 0.23 mmol, 1 equiv.), 2-chloroacetyl chloride (20 μL, 0.26 mmol, 1.1 equiv.), and NaHCO_3_ (58 mg, 0.69 mmol, 3 equiv.) in acetone (8 mL). After column chromatography (NP, CH_2_Cl_2_/CH_3_OH, v/v, 100:0−95.6:4.4), the pure product **14a** was isolated as a white solid (103 mg, 81%). ^1^H NMR (400 MHz, DMSO-*d*_*6*_) δ11.86 − 11.62 (m, 1H), 10.80 − 10.48 (m, 1H), 8.44 − 8.23 (m, 1H), 7.59 (d, *J* = 2.5 Hz, 1H), 7.42 − 7.29 (m, 3H), 7.04 (d, *J* = 8.8 Hz, 1H), 6.17 (t, *J* = 6.6 Hz, 1H), 4.93 − 4.74 (m, 1H), 4.74 − 4.64 (m, 2H), 4.46 − 4.28 (m, 2H), 4.15 − 4.06 (m, 1H), 4.01 − 3.90 (m, 1H), 2.18 − 2.01 (m, 1H), 1.99 − 1.87 (m, 1H), 1.40 − 1.32 (m, 3H), 1.32 − 1.25 (m, 3H). ^13^C NMR (101 MHz, DMSO-*d*_*6*_) δ 171.26, 167.66, 167.65, 162.39, 152.82, 139.69, 134.98, 129.82, 128.39, 126.08, 125.57, 122.86, 115.70, 105.11, 84.11 (d, *J* = 242.5 Hz), 67.88, 48.90, 47.81, 42.67, 42.02, 27.13, 26.89. TLC-MS (ESI) *m*/*z* for (C_22_H_24_Cl_3_FN_4_O_5_ [M—H]^−^) calcd. 547.08, found 547.2. HPLC t_*R*_ = 8.10 min. FT-IR (ATR) [cm^−1^] 3167, 2977, 2926, 1646, 1607, 1478, 1226, 1106, 1070, 763.

#### (*S*)-*N*-(1-(2-(2-Chloroacetyl)-2-((2-oxo-1,2-dihydropyridin-3-yl)­methyl)­hydrazineyl)-4, 4-dimethyl-1-oxopentan-2-yl)-2-(2,4-dichlorophenoxy)­acetamide (**14b**)

Following general procedure E for the synthesis of amides, compound **14b** was synthesized using **13b** (100 mg, 0.21 mmol, 1 equiv.), 2-chloroacetyl chloride (18 μL, 0.23 mmol, 1.1 equiv.), and NaHCO_3_ (53 mg, 0.63 mmol, 3 equiv.) in acetone (8 mL). After column chromatography (NP, CH_2_Cl_2_/CH_3_OH, v/v, 100:0−95.3:4.7), the pure product **14b** was isolated as a white solid (109 mg, 95%). ^1^H NMR (400 MHz, DMSO-*d*_*6*_) δ 11.87 − 11.60 (m, 1H), 10.74 − 10.39 (m, 1H), 8.39 − 8.17 (m, 1H), 7.59 (d, *J* = 2.6 Hz, 1H), 7.41 − 7.25 (m, 3H), 7.10 − 6.93 (m, 1H), 6.16 (t, *J* = 6.6 Hz, 1H), 4.92 − 4.74 (m, 1H), 4.74 − 4.63 (m, 2H), 4.40 − 4.28 (m, 1H), 4.27 − 4.20 (m, 1H), 4.12 − 4.03 (m, 1H), 4.01 − 3.92 (m, 1H), 1.61 − 1.47 (m, 2H), 0.86 (s, 9H). ^13^C NMR (101 MHz, DMSO-*d*_*6*_) δ 172.25, 169.07, 167.53, 162.38, 152.88, 139.93, 135.05, 129.82, 128.36, 125.93, 125.51, 122.84, 115.64, 105.08, 67.88, 49.45, 47.70, 42.65, 41.99, 30.69, 29.82 (3C). TLC-MS (ESI) *m*/*z* for (C_23_H_27_Cl_3_N_4_O_5_ [M—H]^−^) calcd. 543.10, found 543.2. HRMS (ESI-TOF) *m*/*z* for (C_23_H_27_Cl_3_N_4_O_5_ [M+Na]^+^) calcd. 567.0942, found 567.0950. HPLC t_*R*_ = 8.64 min. FT-IR (ATR) [cm^−1^] 3159, 2957, 1642, 1607, 1475, 1226, 1106, 763.

#### (*S*)-*N*-(1-(2-(2-Chloroacetyl)-2-((2-oxo-1,2-dihydropyridin-3-yl)­methyl)­hydrazineyl)-3-cyclopropyl-1-oxopropan-2-yl)-2-(2,4-dichlorophenoxy)­acetamide **(14c**)

Following general procedure E for the synthesis of amides, compound **14c** was synthesized using **13c** (100 mg, 0.22 mmol, 1 equiv.), 2-chloroacetyl chloride (19 μL, 0.24 mmol, 1.1 equiv.), and NaHCO_3_ (58 mg, 0.69 mmol, 3 equiv.) in acetone (8 mL). After column chromatography (NP, CH_2_Cl_2_/CH_3_OH, v/v, 100:0−93.5:6.5), the pure product **14c** was isolated as a white solid (112 mg, 96%). ^1^H NMR (400 MHz, DMSO-*d*_*6*_) δ 11.70 (s, 1H), 10.83 − 10.36 (m, 1H), 8.46 − 8.13 (m, 1H), 7.59 (d, *J* = 2.6 Hz, 1H), 7.39 − 7.29 (m, 3H), 7.13 − 6.99 (m, 1H), 6.20 − 6.11 (m, 1H), 4.91 − 4.62 (m, 3H), 4.46 − 4.30 (m, 1H), 4.31 − 4.18 (m, 1H), 4.16 − 3.98 (m, 2H), 1.69 − 1.54 (m, 1H), 1.52 − 1.32 (m, 1H), 0.75 − 0.58 (m, 1H), 0.44 − 0.26 (m, 2H), 0.16 − −0.04 (m, 2H). ^13^C NMR (101 MHz, DMSO-*d*_*6*_) δ 171.07, 167.33 (2C), 161.85, 152.44, 138.90, 134.42, 129.32, 127.93, 125.57, 125.04, 122.37, 115.25, 104.59, 67.36, 52.22, 47.30, 42.16, 35.58, 7.54, 4.54, 4.02. TLC-MS (ESI) *m*/*z* for (C_22_H_23_Cl_3_N_4_O_5_ [M—H]^−^) calcd. 527.07, found 527.5. HPLC t_*R*_ = 7.95 min. HRMS (ESI-TOF) *m*/*z* for (C_22_H_23_Cl_3_N_4_O_5_ [M+H]+) calcd. 529.0812, found 529.0811. FT-IR (ATR) [cm^−1^] 3162, 2998, 2921, 1647, 1608, 1477, 1242, 1067, 762.

#### (*S*)-*N*-(1-(2-(2-Chloroacetyl)-2-((2-oxo-1,2-dihydropyridin-3-yl)­methyl)­hydrazineyl)-1-oxo-3-phenylpropan-2-yl)-2-(2,4-dichlorophenoxy)­acetamide (**14d**)

Following the general procedure E for the synthesis of amides, compound **14d** was synthesized using **13d** (100 mg, 0.20 mmol, 1 equiv.), 2-chloroacetyl chloride (18 μL, 0.22 mmol, 1.1 equiv.), and NaHCO_3_ (50 mg, 0.60 mmol, 3 equiv.) in acetone (8 mL). After column chromatography (NP, CH_2_Cl_2_/CH_3_OH, v/v, 100:0−95:5), the pure product **14d** was isolated as a white solid (64 mg, 57%). ^1^H NMR (400 MHz, DMSO-*d*_*6*_) δ 11.71 (s, 1H), 10.80 − 10.53 (m, 1H), 8.40 (d, *J* = 7.4 Hz, 1H), 7.57 (d, *J* = 2.6 Hz, 1H), 7.36 − 7.31 (m, 1H), 7.31 − 7.24 (m, 3H), 7.24 − 7.13 (m, 4H), 6.84 (d, *J* = 8.9 Hz, 1H), 6.14 (t, *J* = 6.6 Hz, 1H), 4.94 − 4.69 (m, 1H), 4.69 − 4.58 (m, 2H), 4.58 − 4.30 (m, 2H), 4.15 − 4.02 (m, 1H), 4.00 − 3.63 (m, 1H), 3.10 − 2.95 (m, 1H), 2.94 − 2.79 (m, 1H). ^13^C NMR (101 MHz, DMSO-*d*_*6*_) δ 170.51, 167.32, 167.27, 161.85, 152.33, 138.81, 136.88, 134.41, 129.30, 129.10 (2C), 128.25 (2C), 127.86, 126.55, 125.60, 125.01, 122.35, 115.07, 104.61, 67.27, 52.70, 52.67, 47.31, 42.13. TLC-MS (ESI) *m*/*z* for (C_25_H_23_Cl_3_N_4_O_5_ [M—H]^−^) calcd. 563.07, found 563.2. HRMS (ESI-TOF) *m*/*z* for (C_25_H_23_Cl_3_N_4_O_5_ [M+H]^+^) calcd. 566.0812, found 565.0814. HPLC t_*R*_ = 8.73 min. FT-IR (ATR) [cm^−1^] 3163, 3021, 2922, 1653, 1477, 1223, 1101, 1067, 762, 698.

#### (*S*)-*N*-(1-(2-(2-Chloroacetyl)-2-((2-oxo-1,2-dihydropyridin-3-yl)­methyl)­hydrazineyl)-3-(2-fluorophenyl)-1-oxopropan-2-yl)-2-(2,4-dichlorophenoxy)­acetamide (**14e**)

Following the general procedure E for the synthesis of amides, compound **14e** was synthesized using **13e** (100 mg, 0.20 mmol, 1 equiv.), 2-chloroacetyl chloride (18 μL, 0.22 mmol, 1.1 equiv.), and NaHCO_3_ (50 mg, 0.60 mmol, 3 equiv.) in acetone (8 mL). After column chromatography (NP, CH_2_Cl_2_/CH_3_OH, v/v, 100:0−95.6:4.4), the pure product **14e** was isolated as a light yellow solid (111 mg, 95%). ^1^H NMR (400 MHz, DMSO-*d*_*6*_) δ 11.72 (s, 1H), 10.86 − 10.53 (m, 1H), 8.43 (d, *J* = 7.3 Hz, 1H), 7.58 (d, *J* = 2.5 Hz, 1H), 7.42 − 7.18 (m, 5H), 7.18 − 6.98 (m, 2H), 6.85 (d, *J* = 8.9 Hz, 1H), 6.14 (t, *J* = 6.5 Hz, 1H), 4.97 − 4.69 (m, 1H), 4.70 − 4.61 (m, 2H), 4.59 − 4.45 (m, 1H), 4.43 − 4.26 (m, 1H), 4.17 − 4.06 (m, 1H), 4.00 − 3.68 (m, 1H), 3.21 − 3.00 (m, 1H), 3.00 − 2.85 (m, 1H). ^13^C NMR (101 MHz, DMSO-*d*_*6*_) δ 170.58, 169.07, 167.87, 164.62 (d, *J* = 261.6 Hz), 162.33, 152.81, 134.82, 132.14, 132.01, 129.79, 129.23, 128.35, 126.09, 125.51, 124.73, 123.95, 122.82, 115.69, 115.52, 105.08, 67.72, 51.74, 47.81, 42.70, 41.95. TLC-MS (ESI) *m*/*z* for (C_25_H_22_Cl_3_FN_4_O_5_ [M—H]^−^) calcd. 582.06, found 581.2. HPLC t_*R*_ = 8.56 min. FT-IR (ATR) [cm^−1^] 3163, 2922, 1650, 1607, 1478, 1226, 1106, 759.

#### (*S*)-*N*-(1-(2-(2-Chloroacetyl)-2-((2-oxo-1,2-dihydropyridin-3-yl)­methyl)­hydrazineyl)-3-(3-fluorophenyl)-1-oxopropan-2-yl)-2-(2,4-dichlorophenoxy)­acetamide (**14f**)

Following the general procedure E for the synthesis of amides, compound **14f** was synthesized using **13f** (100 mg, 0.20 mmol, 1 equiv.), 2-chloroacetyl chloride (18 μL, 0.22 mmol, 1.1 equiv.), and NaHCO_3_ (50 mg, 0.60 mmol, 3 equiv.) in acetone (8 mL). After column chromatography (NP, CH_2_Cl_2_/CH_3_OH, v/v, 100:0−95.7:4.3), the pure product **14f** was isolated as a white solid (89 mg, 76%). ^1^H NMR (400 MHz, DMSO-*d*_*6*_) δ 11.71 (s, 1H), 10.77 − 10.55 (m, 1H), 8.43 (d, *J* = 7.4 Hz, 1H), 7.56 (d, *J* = 2.6 Hz, 1H), 7.36 − 7.32 (m, 1H), 7.32 − 7.25 (m, 2H), 7.24 − 7.20 (m, 1H), 7.14 − 6.96 (m, 3H), 6.84 (d, *J* = 8.9 Hz, 1H), 6.15 (t, *J* = 6.6 Hz, 1H), 4.88 − 4.68 (m, 1H), 4.69 − 4.51 (m, 3H), 4.50 − 4.30 (m, 1H), 4.18 − 4.06 (m, 1H), 3.99 − 3.74 (m, 1H), 3.13 − 2.97 (m, 1H), 2.96 − 2.83 (m, 1H). ^13^C NMR (101 MHz, DMSO-*d*_*6*_) δ 170.38, 169.28, 167.41, 162.01 (d, *J* = 243.5 Hz), 161.88, 152.32, 139.96, 138.94, 134.47, 130.09, 129.31, 127.80, 125.60, 125.24, 125.04, 122.38, 115.85, 115.03, 104.61, 67.32, 52.50, 52.39, 47.34, 42.07. TLC-MS (ESI) *m*/*z* for (C_25_H_22_Cl_3_FN_4_O_5_ [M—H]^−^) calcd. 582.06, found 581.4. HPLC t_*R*_ = 8.31 min. HRMS (ESI-TOF) *m*/*z* for (C_25_H_22_Cl_3_FN_4_O_5_ [M+Na]^+^) calcd. 605.0538, found 605.0543. FT-IR (ATR) [cm^−1^] 3168, 3021, 2927, 1641, 1477, 1244, 1226, 765.

#### (*S*)-*N*-(1-(2-(2-Chloroacetyl)-2-((2-oxo-1,2-dihydropyridin-3-yl)­methyl)­hydrazineyl)-3-(4-fluorophenyl)-1-oxopropan-2-yl)-2-(2,4-dichlorophenoxy)­acetamide (**14g**)

Following general procedure E for the synthesis of amides, compound **14g** was synthesized using **13g** (100 mg, 0.20 mmol, 1 equiv.), 2-chloroacetyl chloride (18 μL, 0.22 mmol, 1.1 equiv.), and NaHCO_3_ (50 mg, 0.60 mmol, 3 equiv.) in acetone (8 mL). After column chromatography (NP, CH_2_Cl_2_/CH_3_OH, v/v, 100:0−95.5:4.5), the pure product **14g** was isolated as a white solid (94 mg, 81%). ^1^H NMR (400 MHz, DMSO-*d*_*6*_) δ 11.71 (s, 1H), 10.80 − 10.47 (m, 1H), 8.41 (d, *J* = 7.4 Hz, 1H), 7.57 (d, *J* = 2.6 Hz, 1H), 7.37 − 7.32 (m, 1H), 7.31 − 7.20 (m, 4H), 7.14 − 6.97 (m, 2H), 6.85 (d, *J* = 8.9 Hz, 1H), 6.23 − 6.08 (m, 1H), 4.97 − 4.69 (m, 1H), 4.68 − 4.58 (m, 2H), 4.58 − 4.28 (m, 2H), 4.14 − 4.08 (m, 1H), 4.00 − 3.73 (m, 1H), 3.09 − 2.94 (m, 1H), 2.93 − 2.78 (m, 1H). ^13^C NMR (101 MHz, DMSO-*d*_*6*_) δ 170.40, 168.70, 167.24, 161.83, 161.06 (d, *J* = 241.9 Hz), 152.32, 138.76, 134.40, 133.04, 130.98, 130.96, 129.29, 127.79, 125.59, 125.02, 122.37, 115.05 (2C), 114.82, 104.57, 67.29, 52.69, 52.65, 47.27, 42.02. TLC-MS (ESI) *m*/*z* for (C_25_H_22_Cl_3_FN_4_O_5_ [M—H]^−^) calcd. 582.06, found 581.5. HPLC t_*R*_ = 8.31 min. FT-IR (ATR) [cm^−1^] 3182, 2921, 1653, 1507, 1477, 1220, 1068, 761.

#### (*S*)-*N*-(1-(2-(2-Chloroacetyl)-2-((2-oxo-1,2-dihydropyridin-3-yl)­methyl)­hydrazineyl)-3-(3,5-difluorophenyl)-1-oxopropan-2-yl)-2-(2,4-dichlorophenoxy)­acetamide (**14h**)

Following general procedure E for the synthesis of amides, compound **14h** was synthesized using **13h** (100 mg, 0.19 mmol, 1 equiv.), 2-chloroacetyl chloride (17 μL, 0.21 mmol, 1.1 equiv.), and NaHCO_3_ (48 mg, 0.57 mmol, 3 equiv.) in acetone (8 mL). After column chromatography (NP, CH_2_Cl_2_/CH_3_OH, v/v, 100:0−95.5:4.5), the pure product **14h** was isolated as a light yellow solid (67 mg, 59%). ^1^H NMR (400 MHz, DMSO-*d*_*6*_) δ 11.72 (s, 1H), 10.77 − 10.54 (m, 1H), 8.44 (d, *J* = 7.5 Hz, 1H), 7.57 (d, *J* = 2.6 Hz, 1H), 7.42 − 7.30 (m, 2H), 7.27 − 7.20 (m, 1H), 7.12 − 6.95 (m, 3H), 6.86 (d, *J* = 8.9 Hz, 1H), 6.22 − 6.09 (m, 1H), 4.95 − 4.69 (m, 1H), 4.72 − 4.47 (m, 3H), 4.40 − 4.03 (m, 2H), 4.01 − 3.88 (m, 1H), 3.18 − 3.00 (m, 1H), 3.00 − 2.86 (m, 1H). ^13^C NMR (101 MHz, DMSO-*d*_*6*_) δ 170.24, 170.22, 167.43, 162.18 (d, *J* = 245.7 Hz), 162.05 (d, *J* = 245.9 Hz), 161.89, 152.30, 141.73, 139.08, 134.51, 129.30, 127.72, 125.59, 125.07, 122.40, 115.01, 112.39, 112.15, 104.61, 102.08, 67.41, 52.29, 52.27, 47.35, 42.03. TLC-MS (ESI) *m*/*z* for (C_25_H_21_Cl_3_F_2_N_4_O_5_ [M—H]^−^) calcd. 599.06, found 599.6. HRMS (ESI-TOF) *m*/*z* for (C_25_H_21_Cl_3_F_2_N_4_O_5_ [M+H]^+^) calcd. 601.0624, found 601.0628. HPLC t_*R*_ = 8.83 min. FT-IR (ATR) [cm^−1^] 3171, 1950, 2920, 1646, 1595, 1477, 1242, 1116, 849, 764.

#### (*S*)-*N*-(1-(2-(2-Chloroacetyl)-2-((2-oxo-1,2-dihydropyridin-3-yl)­methyl)­hydrazineyl)-3-(3-chlorophenyl)-1-oxopropan-2-yl)-2-(2,4-dichlorophenoxy)­acetamide (**14i**)

Following general procedure E for the synthesis of amides, compound **14i** was synthesized using **13i** (100 mg, 0.19 mmol, 1 equiv.), 2-chloroacetyl chloride (17 μL, 0.21 mmol, 1.1 equiv.), and NaHCO_3_ (88 mg, 0.57 mmol, 3 equiv.) in acetone (8 mL). After column chromatography (NP, CH_2_Cl_2_/CH_3_OH, v/v, 100:0−95.4:4.6), the pure product **14i** was isolated as a white solid (108 mg, 95%). ^1^H NMR (400 MHz, DMSO-*d*_*6*_) δ 11.88 − 11.54 (m, 1H), 10.84 − 10.55 (m, 1H), 8.46 (d, *J* = 7.5 Hz, 1H), 7.57 (d, *J* = 2.6 Hz, 1H), 7.41 − 7.32 (m, 2H), 7.32 − 7.25 (m, 3H), 7.25 − 7.22 (m, 1H), 7.22 − 7.16 (m, 1H), 6.83 (d, *J* = 8.9 Hz, 1H), 6.16 (t, *J* = 6.6 Hz, 1H), 4.89 − 4.70 (m, 1H), 4.70 − 4.49 (m, 3H), 4.48 − 4.32 (m, 1H), 4.21 − 4.06 (m, 1H), 4.00 − 3.77 (m, 1H), 3.13 − 2.95 (m, 1H), 2.94 − 2.81 (m, 1H). ^13^C NMR (101 MHz, DMSO-*d*_*6*_) δ 170.89, 167.92, 167.19, 162.37, 152.80, 140.14, 139.54, 134.99, 133.36, 130.51, 129.83, 129.80, 129.44, 128.33, 128.29, 127.06, 125.51, 122.86, 115.47, 105.11, 67.77, 53.04, 47.66, 42.60, 40.52. TLC-MS (ESI) *m*/*z* for (C_25_H_22_Cl_4_N_4_O_5_ [M + H]^+^) calcd. 599.03, found 598.9. HPLC t_*R*_ = 8.76 min. FT-IR (ATR) [cm^−1^] 3159, 2934, 1642, 1603, 1475, 1428, 1226, 1101, 763.

#### (*S*)-*N*-(1-(2-(2-Chloroacetyl)-2-((2-oxo-1,2-dihydropyridin-3-yl)­methyl)­hydrazineyl)-3-cyclohexyl-1-oxopropan-2-yl)-2-(2,4-dichlorophenoxy)­acetamide (**14j**)

Following the general procedure O for the synthesis of amides, compound **14j** was synthesized using **13j** (100 mg, 0.20 mmol, 1 equiv.), 2-chloroacetyl chloride (18 μL, 0.22 mmol, 1.1 equiv.), and NaHCO_3_ (50 mg, 0.60 mmol, 3 equiv.) in acetone (8 mL). After column chromatography (NP, CH_2_Cl_2_/CH_3_OH, v/v, 100:0−93.8:6.2), the pure product **14j** was isolated as a white solid (90 mg, 79%). ^1^H NMR (400 MHz, DMSO-*d*_*6*_) δ 11.72 (s, 1H), 10.78 − 10.42 (m, 1H), 8.40 − 8.12 (m, 1H), 7.60 (d, *J* = 2.6 Hz, 1H), 7.41 − 7.26 (m, 3H), 7.12 − 6.96 (m, 1H), 6.21 − 6.11 (m, 1H), 4.89 − 4.62 (m, 3H), 4.46 − 4.30 (m, 1H), 4.28 − 4.19 (m, 1H), 4.13 − 3.98 (m, 2H), 1.72 − 1.53 (m, 5H), 1.52 − 1.38 (m, 2H), 1.19 − 1.01 (m, 4H), 0.93 − 0.72 (m, 2H). ^13^C NMR (101 MHz, DMSO-*d*_*6*_) δ 172.02, 170.80, 167.82, 162.34, 152.93, 134.94, 129.83, 128.37, 125.95, 125.54, 125.52, 122.88, 115.64, 105.06, 67.85, 60.22, 47.78, 42.60, 38.58, 33.80, 33.44, 32.20, 26.43, 26.10, 25.92. TLC-MS (ESI) *m*/*z* for (C_25_H_29_Cl_3_N_4_O_5_ [M—H]^−^) calcd. 569.12, found 569.5. HPLC t_*R*_ = 9.74 min. FT-IR (ATR) [cm^−1^] 3195, 3920, 2847, 1647, 1477, 1224, 1067, 762.

#### *N*-(2-(2-(2-Chloroacetyl)-2-((2-oxo-1,2-dihydropyridin-3-yl)­methyl)­hydrazine-1-carbonyl)-2,3-dihydro-1*H*-inden-2-yl)-2-(2,4-dichlorophenoxy)­acetamide (**14k**)

Following general procedure E for the synthesis of amides, compound **14k** was synthesized using **13k** (100 mg, 0.20 mmol, 1 equiv.), 2-chloroacetyl chloride (17 μL, 0.22 mmol, 1.1 equiv.), and NaHCO_3_ (50 mg, 0.60 mmol, 3 equiv.) in acetone (8 mL). After column chromatography (NP, CH_2_Cl_2_/CH_3_OH, v/v, 100:0−95.2:4.8), the pure product **14k** was isolated as a white solid (106 mg, 92%). ^1^H NMR (400 MHz, DMSO-*d*_*6*_) δ 11.77 (s, 1H), 10.46 (s, 1H), 8.77 (s, 1H), 7.53 (d, *J* = 2.6 Hz, 1H), 7.44 − 7.39 (m, 1H), 7.39 − 7.33 (m, 1H), 7.26 − 7.20 (m, 2H), 7.21 − 7.15 (m, 3H), 7.03 (d, *J* = 9.0 Hz, 1H), 6.24 − 6.18 (m, 1H), 4.80 − 4.72 (m, 1H), 4.70 − 4.61 (m, 2H), 4.30 − 4.20 (m, 1H), 4.10 − 4.03 (m, 1H), 4.00 − 3.92 (m, 1H), 3.75 − 3.66 (m, 1H), 3.47 − 3.36 (m, 1H), 3.16 − 3.07 (m, 2H). ^13^C NMR (101 MHz, DMSO-*d*_*6*_) δ 172.08, 167.88, 167.56, 162.26, 152.58, 140.18, 140.03, 139.23, 134.62, 129.24, 127.71, 126.78, 126.67, 126.07, 124.88, 124.52, 124.40, 122.26, 115.21, 104.83, 67.09, 65.11, 54.87, 47.76, 42.63, 42.06. TLC-MS (ESI) *m*/*z* for (C_26_H_23_Cl_3_N_4_O_5_ [M—H]^−^) calcd. 575.07, found 575.1. HPLC t_*R*_ = 8.42 min. FT-IR (ATR) [cm^−1^] 3252, 2954, 1669, 1641, 1607, 1477, 1226, 741.

#### (*S*)-*N’*-(2-Chloroacetyl)-1-(2-(2,4-dichlorophenoxy)­acetyl)-*N’*-((2-oxo-1,2-dihydropyridin-3-yl)­methyl)­pyrrolidine-2-carbohydrazide (**14l**)

Following the general procedure E for the synthesis of amides, compound **14l** was synthesized using **13l** (100 mg, 0.23 mmol, 1 equiv.), 2-chloroacetyl chloride (20 μL, 0.25 mmol, 1.1 equiv.), and NaHCO_3_ (58 mg, 0.69 mmol, 3 equiv.) in acetone (8 mL). After column chromatography (NP, CH_2_Cl_2_/CH_3_OH, v/v, 100:0−94:6), the pure product **14l** was isolated as a white solid (71 mg, 60%). ^1^H NMR (400 MHz, DMSO-*d*_*6*_) δ 11.71 (s, 1H), 10.68 − 10.23 (m, 1H), 7.55 (d, *J* = 2.6 Hz, 1H), 7.44 − 7.21 (m, 3H), 7.19 − 6.95 (m, 1H), 6.22 − 6.10 (m, 1H), 5.04 − 4.87 (m, 2H), 4.87 − 4.57 (m, 1H), 4.44 − 4.26 (m, 1H), 4.25 − 4.20 (m, 1H), 4.19 − 3.93 (m, 2H), 3.67 − 3.40 (m, 2H), 2.16 − 1.71 (m, 4H). ^13^C NMR (101 MHz, DMSO-*d*_*6*_) δ 168.50, 167.47, 165.74, 161.97, 152.67, 134.70, 129.11, 127.70, 127.63, 125.53, 124.60, 122.04, 115.36, 104.58, 66.64, 58.10, 47.07, 45.57, 42.28, 41.51, 28.34. TLC-MS (ESI) *m*/*z* for (C_21_H_21_Cl_3_N_4_O_5_ [M—H]^−^) calcd 513.06, found 513.5. HPLC t_*R*_ = 6.62 min. FT-IR (ATR) [cm^−1^] 3152, 2950, 1645, 1602, 1476, 1457, 1239, 764.

#### (1*R*,2*S*,5*S*)-*N’*-(2-Chloroacetyl)-3-(2-(2,4-dichlorophenoxy)­acetyl)-6,6-dimethyl-*N’*-((2-oxo-1,2-dihydropyridin-3-yl)­methyl)-3-azabicyclo*[3.1.0]*hexane-2-carbohydrazide (**14m**)

Following general procedure E for the synthesis of amides, compound **14m** was synthesized using **13m** (100 mg, 0.21 mmol, 1 equiv.), 2-chloroacetyl chloride (18 μL, 0.23 mmol, 1.1 equiv.), and NaHCO_3_ (53 mg, 0.63 mmol, 3 equiv.) in acetone (8 mL). After column chromatography (NP, CH_2_Cl_2_/CH_3_OH, v/v, 100:0−94.5:5.5), the pure product **14m** was isolated as a white solid (108 mg, 93%). ^1^H NMR (400 MHz, DMSO-*d*_*6*_) δ 11.75 (s, 1H), 10.84 − 10.24 (m, 1H), 7.56 (d, *J* = 2.6 Hz, 1H), 7.45 − 7.33 (m, 2H), 7.34 − 7.23 (m, 1H), 7.17 − 6.85 (m, 1H), 6.25 − 6.12 (m, 1H), 5.03 (d, *J* = 15.7 Hz, 1H), 4.97 − 4.82 (m, 1H), 4.75 (d, *J* = 14.9 Hz, 1H), 4.52 − 4.31 (m, 1H), 4.29 − 4.07 (m, 2H), 4.03 − 3.95 (m, 1H), 3.90 − 3.73 (m, 1H), 3.64 − 3.49 (m, 1H), 1.65 − 1.47 (m, 1H), 1.46 − 1.29 (m, 1H), 1.05 − 0.99 (m, 3H), 0.94 − 0.84 (m, 3H). ^13^C NMR (101 MHz, DMSO-*d*_*6*_) δ 170.46, 170.35, 162.08, 162.03, 152.45, 134.66, 129.20, 127.79, 127.69, 125.76, 124.69, 122.03, 115.18, 104.67, 66.50, 59.76, 58.80, 47.34, 45.50, 42.23, 29.41, 27.51, 25.76, 25.72. TLC-MS (ESI) *m*/*z* for (C_24_H_25_Cl_3_N_4_O_5_ [M—H]^−^) calcd. 553.09, found 553.0. HRMS (ESI-TOF) *m*/*z* for (C_24_H_25_Cl_3_N_4_O_5_ [M+H^+^) calcd. 555.0969, found 555.0973. HPLC t_*R*_ = 8.39 min. FT-IR (ATR) [cm^−1^] 3192, 2950, 1647, 1608, 1477, 1424, 1238, 765.

#### (*S*)-*N’*-(2-Chloroacetyl)-1-(2-(2,4-dichlorophenoxy)­acetyl)-*N’*-((2-oxo-1,2-dihydropyridin-3-yl)­methyl)­indoline-2-carbohydrazide (**14n**)

Following general procedure E for the synthesis of amides, compound **14n** was synthesized using **13n** (100 mg, 0.21 mmol, 1 equiv.), 2-chloroacetyl chloride (18 μL, 0.23 mmol, 1.1 equiv.), and NaHCO_3_ (53 mg, 0.63 mmol, 3 equiv.) in acetone (8 mL). After column chromatography (NP, CH_2_Cl_2_/CH_3_OH, v/v, 100:0−94.2:4.8), the pure product **14n** was isolated as an off-white solid (102 mg, 86%). ^1^H NMR (400 MHz, DMSO-*d*_*6*_) δ 11.74 (s, 1H), 10.95 − 10.55 (m, 1H), 8.06 − 7.92 (m, 1H), 7.63 − 7.50 (m, 1H), 7.44 − 7.31 (m, 2H), 7.30 − 7.24 (m, 2H), 7.24 − 7.16 (m, 1H), 7.14 − 7.00 (m, 2H), 6.23 − 6.07 (m, 1H), 5.50 − 5.09 (m, 2H), 5.01 − 4.65 (m, 2H), 4.53 − 4.17 (m, 2H), 4.11 − 3.90 (m, 1H), 3.69 − 3.52 (m, 1H), 3.25 − 2.92 (m, 1H). ^13^C NMR (101 MHz, DMSO-*d*_*6*_) δ 170.30, 167.08, 165.43, 162.15, 152.60, 140.72, 134.87, 129.21, 129.19, 127.73, 127.68, 127.64, 127.28, 124.66, 124.55, 123.98, 122.07, 115.55, 115.13, 104.63, 66.48, 58.47, 58.44, 42.00, 33.96. TLC-MS (ESI) *m*/*z* for (C_25_H_21_Cl_3_N_4_O_5_ [M]^−^) calcd. 562.06, found 561.9. HPLC t_*R*_ = 7.69 min. FT-IR (ATR) [cm^−1^] 3245, 3007, 3019, 1698, 1649, 1487, 1408, 1217, 783, 744.

#### (2*S*,3*aS*,7*aS*)-*N’*-(2-Chloroacetyl)-1-(2-(2,4-dichlorophenoxy)­acetyl)-*N’*-((2-oxo-1,2-dihydropyridin-3-yl)­methyl)­octahydro-1H-indole-2-carbohydrazide (**14o**)

Following general procedure E for the synthesis of amides, compound **14o** was synthesized using **13o** (100 mg, 0.20 mmol, 1 equiv.), 2-chloroacetyl chloride (18 μL, 0.22 mmol, 1.1 equiv.), and NaHCO_3_ (50 mg, 0.60 mmol, 3 equiv.) in acetone (8 mL). After column chromatography (NP, CH_2_Cl_2_/CH_3_OH, v/v, 100:0−95:5), the pure product **14o** was isolated as a white solid (64 mg, 56%). ^1^H NMR (400 MHz, DMSO-*d*_*6*_) δ 11.68 (s, 1H), 10.69 − 10.09 (m, 1H), 7.54 (d, *J* = 2.6 Hz, 1H), 7.42 − 7.31 (m, 2H), 7.31 − 7.25 (m, 1H), 7.23 − 6.94 (m, 1H), 6.22 − 6.09 (m, 1H), 5.15 − 5.00 (m, 1H), 4.94 − 4.67 (m, 2H), 4.46 − 4.25 (m, 1H), 4.24 − 4.16 (m, 1H), 4.15 − 4.01 (m, 1H), 3.98 − 3.88 (m, 1H), 2.41 − 2.24 (m, 1H), 2.09 − 1.76 (m, 3H), 1.75 − 1.38 (m, 5H), 1.35 − 1.05 (m, 3H). ^13^C NMR (101 MHz, DMSO-*d*_*6*_) δ 171.22, 167.89, 163.75, 161.90, 152.72, 139.64, 134.62, 129.12, 127.66, 125.47, 124.63, 122.12, 115.50, 104.54, 66.29, 58.05, 56.27, 47.09, 42.35, 39.22, 36.92, 29.46, 27.22, 25.24, 23.36. TLC-MS (ESI) *m*/*z* for (C_25_H_22_Cl_3_FN_4_O_5_ [M]^−^) calcd. 568.10, found 568.0. HPLC t_*R*_ = 8.29 min. FT-IR (ATR) [cm^−1^] 3153, 2926, 2853, 1643, 1476, 1457, 1226, 762.

#### (S)-*N’*-(2-Chloroacetyl)-1-(2-(2,4-dichlorophenoxy)­acetyl)-*N’*-((2-oxo-1,2-dihydropyridin-3-yl)­methyl)­piperidine-2-carbohydrazide (**14p**)

Following general procedure E for the synthesis of amides, compound **14p** was synthesized using **13p** (100 mg, 0.22 mmol, 1 equiv.), 2-chloroacetyl chloride (19 μL, 0.24 mmol, 1.1 equiv.), and NaHCO_3_ (55 mg, 0.66 mmol, 3 equiv.) in acetone (8 mL). After column chromatography (NP, CH_2_Cl_2_/CH_3_OH, v/v, 100:0−94.2:4.8), the pure product **14p** was isolated as a white solid (107 mg, 92%). ^1^H NMR (400 MHz, DMSO-*d*_*6*_) δ 11.72 (s, 1H), 10.59 − 10.31 (m, 1H), 7.55 (d, *J* = 2.6 Hz, 1H), 7.48 − 7.21 (m, 4H), 7.13 − 6.92 (m, 1H), 6.22 − 6.11 (m, 1H), 5.18 − 5.06 (m, 1H), 4.99 − 4.91 (m, 1H), 4.86 − 4.78 (m, 1H), 4.77 − 4.60 (m, 1H), 4.43 − 4.22 (m, 2H), 4.17 − 4.05 (m, 1H), 3.74 − 3.59 (m, 1H), 2.67 − 2.50 (m, 1H), 1.68 − 1.43 (m, 4H), 1.35 − 1.23 (m, 1H). ^13^C NMR (101 MHz, DMSO-*d*_*6*_) δ 170.31, 169.43, 167.30, 162.01, 152.65, 140.02, 134.75, 129.14, 127.76, 127.67, 124.50, 122.02, 115.29, 104.65, 66.55, 59.73, 47.26, 42.18, 42.16, 26.33, 24.31, 24.27. TLC-MS (ESI) *m*/*z* for (C_22_H_23_Cl_3_N_4_O_5_ [M—H]^−^) calcd. 527.07, found 527.4. HPLC t_*R*_ = 7.42 min. FT-IR (ATR) [cm^−1^] 3160, 2939, 1636, 1609, 1477, 1430, 1233, 1103, 1012, 766.

#### (*S*)-*N’*-(2-Chloroacetyl)-2-(2-(2,4-dichlorophenoxy)­acetyl)-*N’*-((2-oxo-1,2-dihydropyridin-3-yl)­methyl)-1,2,3,4-tetrahydroisoquinoline-3-carbohydrazide (**14q**)

Following general procedure E for the synthesis of amides, compound **14q** was synthesized using **13q** (100 mg, 0.20 mmol, 1 equiv.), 2-chloroacetyl chloride (17 μL, 0.22 mmol, 1.1 equiv.), and NaHCO_3_ (50 mg, 0.60 mmol, 3 equiv.) in acetone (8 mL). After column chromatography (NP, CH_2_Cl_2_/CH_3_OH, v/v, 100:0−94.2:4.8), the pure product **14q** was isolated as a white solid (107 mg, 92%). ^1^H NMR (400 MHz, DMSO-*d*_*6*_) δ 11.69 (s, 1H), 10.64 − 10.29 (m, 1H), 7.61 − 7.52 (m, 1H), 7.34 − 7.26 (m, 3H), 7.26 − 7.17 (m, 4H), 7.16 − 7.05 (m, 1H), 7.05 − 6.95 (m, 1H), 6.22 − 5.99 (m, 1H), 5.31 − 5.10 (m, 2H), 5.03 − 4.87 (m, 1H), 4.87 − 4.76 (m, 1H), 4.75 − 4.66 (m, 2H), 4.66 − 4.58 (m, 1H), 4.00 − 3.82 (m, 1H), 3.80 − 3.68 (m, 1H), 3.59 − 3.36 (m, 1H), 3.19 − 3.09 (m, 1H). ^13^C NMR (101 MHz, DMSO-*d*_*6*_) δ 170.79, 169.05, 167.79, 162.70, 153.23, 134.89, 132.82, 129.63, 128.13, 128.09, 127.87, 127.41, 127.31, 126.77, 125.95, 125.09, 125.03, 122.56, 115.98, 105.17, 67.12, 60.21, 53.47, 48.15, 41.94, 31.31. TLC-MS (ESI) *m*/*z* for (C_26_H_23_Cl_3_N_4_O_5_ [M]^−^) calcd. 576.07, found 576.0. HPLC t_*R*_ = 7.83 min. HRMS (ESI-TOF) *m*/*z* for (C_26_H_23_Cl_3_N_4_O_5_ [M+Na]^+^) calcd. 599.0632, found 599.0631. FT-IR (ATR) [cm^−1^] 3216, 2953, 1641, 1607, 1477, 1420, 1231, 1045, 764.

#### (1*S*,3*aR*,6*aS*)-*N’*-(2-Chloroacetyl)-2-(2-(2,4-dichlorophenoxy)­acetyl)-*N’*-((2-oxo-1,2-dihydropyridin-3-yl)­methyl)­octahydrocyclopenta*[c]*pyrrole-1-carbohydrazide (**14r**)

Following general procedure E for the synthesis of amides, compound **14r** was synthesized using **13r** (100 mg, 0.21 mmol, 1 equiv.), 2-chloroacetyl chloride (18 μL, 0.23 mmol, 1.1 equiv.), and NaHCO_3_ (53 mg, 0.63 mmol, 3 equiv.) in acetone (8 mL). After column chromatography (NP, CH_2_Cl_2_/CH_3_OH, v/v, 100:0−94.5:5.5), the pure product **14r** was isolated as a white solid (105 mg, 90%). ^1^H NMR (400 MHz, DMSO-*d*_*6*_) δ 11.74 (s, 1H), 10.69 − 10.28 (m, 1H), 7.55 (d, *J* = 2.5 Hz, 1H), 7.39 − 7.32 (m, 2H), 7.32 − 7.22 (m, 1H), 7.19 − 6.89 (m, 1H), 6.21 − 6.11 (m, 1H), 4.99 (d, *J* = 15.7 Hz, 1H), 4.93 − 4.82 (m, 1H), 4.83 − 4.68 (m, 1H), 4.42 − 4.27 (m, 1H), 4.24 − 4.08 (m, 1H), 4.01 − 3.93 (m, 2H), 3.83 − 3.66 (m, 1H), 3.52 − 3.34 (m, 1H), 2.79 − 2.63 (m, 1H), 2.46 − 2.35 (m, 1H), 1.85 − 1.67 (m, 3H), 1.59 − 1.42 (m, 3H). ^13^C NMR (101 MHz, DMSO-*d*_*6*_) δ 170.82, 168.93, 167.75, 162.44, 153.02, 140.37, 135.27, 129.62, 128.15, 125.80, 125.10, 122.48, 115.77, 105.08, 67.05, 64.74, 51.97, 47.59, 47.20, 43.40, 42.86, 31.76, 31.71, 25.03. TLC-MS (ESI) *m*/*z* for (C_24_H_25_Cl_3_N_4_O_5_ [M—H]^−^) calcd. 553.09, found 553.0. HPLC t_*R*_ = 7.71 min. HRMS (ESI-TOF) *m*/*z* for (C_24_H_25_Cl_3_N_4_O_5_ [M+H^+^) calcd. 555.0969, found 559.0969. FT-IR (ATR) [cm^−1^] 3157, 2948, 2871, 1645, 1608, 1477, 1238, 766.

#### (1*S*,3*aR*,6*aS*)-*N’*-((5-Chloro-2-oxo-1,2-dihydropyridin-3-yl)­methyl)-*N’*-(2,2-dichloroacetyl)-2-(2-(2,4-dichlorophenoxy)­acetyl)­octahydrocyclopenta*[c]*pyrrole-1-carbohydrazide (**14s**)

Following the general procedure E for the synthesis of amides, compound **14s** was synthesized using **13s** (130 mg, 0.25 mmol, 1 equiv.), 2,2-dichloroacetyl chloride (27 μL, 0.28 mmol, 1.1 equiv.), and NaHCO_3_ (63 mg, 0.75 mmol, 3 equiv.) in acetone (8 mL). After column chromatography (NP, CH_2_Cl_2_/CH_3_OH, v/v, 100:0−95.6:4.4), the pure product **14s** was isolated as a white solid (120 mg, 77%). ^1^H NMR (400 MHz, DMSO-*d*_*6*_) δ 12.07 (s, 1H), 11.03 − 10.58 (m, 1H), 7.63 (d, *J* = 2.8 Hz, 1H), 7.55 (d, *J* = 2.5 Hz, 1H), 7.47 − 7.36 (m, 1H), 7.34 − 7.19 (m, 1H), 7.18 − 6.88 (m, 1H), 6.80 − 6.48 (m, 1H), 5.09 − 4.95 (m, 1H), 4.96 − 4.84 (m, 1H), 4.83 − 4.65 (m, 1H), 4.15 − 3.95 (m, 2H), 3.86 − 3.69 (m, 1H), 3.58 − 3.41 (m, 1H), 2.83 − 2.63 (m, 1H), 2.61 − 2.51 (m, 1H), 1.91 − 1.65 (m, 3H), 1.65 − 1.51 (m, 2H), 1.51 − 1.39 (m, 1H). ^13^C NMR (101 MHz, DMSO-*d*_*6*_) δ 172.02, 166.17, 160.93, 159.51, 153.04, 140.42, 134.09, 130.11, 129.61, 128.22, 125.14, 125.08, 122.59, 115.94, 67.23, 64.53, 55.38, 52.02, 47.87, 47.06, 43.65, 31.27, 31.24, 24.99. TLC-MS (ESI) *m*/*z* for (C_24_H_23_Cl_5_N_4_O_5_ [M + H]^+^) calcd. 623.4, found 623.4. HPLC t_*R*_ = 8.93 min. FT-IR (ATR) [cm^−1^] 2957, 2875, 1700, 1650, 1599, 1478, 1237, 798, 657.

#### Methyl (4-methoxy-1*H*-indole-2-carbonyl)-l-leucinate (**15a**)

Obtained from the reaction of 4-methoxy-1*H*-indole-2-carboxylic acid (191 mg, 1 mmol) and *L-*leucinemethylester • HCl (182 mg, 1 mmol) following general procedure A. Flash purification with petroleum ether/EtOAc (0−70% EtOAc). Yield: 287 mg (90%) of **15a** as a light-yellow solid. ^1^H NMR (400 MHz, CDCl_3_) δ 9.62 (s, 1H), 7.22 − 7.17 (m, 1H), 7.09 − 7.07 (m, 1H), 7.04 (d, *J* = 8.3 Hz, 1H), 6.66 (d, *J* = 8.4 Hz, 1H), 6.50 (d, *J* = 7.7 Hz, 1H), 4.92 − 4.84 (m, 1H), 3.95 (s, 3H), 3.78 (s, 3H), 1.82 − 1.68 (m, 3H), 0.99 (d, *J* = 5.7 Hz, 3H), 0.98 (d, *J* = 6.0 Hz, 3H). ESI-MS [M + Na]^+^ = 341.2. HPLC t_*R*_ = 7.96 min.

#### Methyl (4,6-difluoro-1*H*-indole-2-carbonyl)-l-leucinate (**15b**)

Obtained from the reaction of 4,6-difluoro-1*H*-indole-2-carboxylic acid (197 mg, 1 mmol) and *L-*leucinemethylester • HCl (182 mg, 1 mmol) following general procedure A. Flash purification with petroleum ether/EtOAc (0−35% EtOAc). Yield: 302 mg (93%) of **15b** as a light-yellow solid. ^1^H NMR (400 MHz, CDCl_3_) δ 10.43 (s, 1H), 7.02 − 6.96 (m, 2H), 6.95 − 6.90 (m, 1H), 6.63 − 6.54 (m, 1H), 4.95 − 4.87 (m, 1H), 3.83 (s, 3H), 1.85 − 1.70 (m, 3H), 1.02 − 1.00 (m, 3H), 1.00 − 0.97 (m, 3H). ESI-MS [M—H]^−^ = 323.2. HPLC t_*R*_ = 9.25 min.

#### Methyl (4,6-dichloro-1*H*-indole-2-carbonyl)-l-leucinate (**15c**)

Obtained from the reaction of 4,6-dichloro-1*H*-indole-2-carboxylic acid (230 mg, 1 mmol) and *L-*leucinemethylester • HCl (182 mg, 1 mmol) following general procedure A. Flash purification with petroleum ether/EtOAc (0−40% EtOAc). Yield: 307 mg (86%) of **15c** as a white solid. ^1^H NMR (400 MHz, CDCl_3_) δ 10.43 (s, 1H), 7.35 − 7.33 (m, 1H), 7.13 − 7.10 (m, 1H), 7.01 − 6.96 (m, 2H), 4.95 − 4.87 (m, 1H), 3.83 (s, 3H), 1.84 − 1.73 (m, 3H), 1.02 (d, *J* = 6.5 Hz, 3H), 1.00 (d, *J* = 6.4 Hz, 3H). ESI-MS [M—H]^−^ = 355.3. HPLC t_*R*_ = 9.76 min.

#### Methyl (S)-3-cyclohexyl-2-(4-methoxy-1H-indole-2-carboxamido)­propanoate (**15d**)

Obtained from the reaction of 4-methoxy-1*H*-indole-2-carboxylic acid (191 mg, 1 mmol) and methyl (*S*)-2-amino-3-cyclohexylpropanoate • HCl (221 mg, 1 mmol) following general procedure A. Flash purification with petroleum ether/EtOAc (0−32% EtOAc). Yield: 347 mg (97%) of **15d** as a white solid. ^1^H NMR (400 MHz, CDCl_3_) δ 9.55 (s, 1H), 7.23 − 7.18 (m, 1H), 7.08 − 7.06 (m, 1H), 7.04 (d, *J* = 8.3 Hz, 1H), 6.60 (d, *J* = 8.4 Hz, 1H), 6.51 (d, *J* = 7.7 Hz, 1H), 4.94 − 4.84 (m, 1H), 3.96 (s, 3H), 3.77 (s, 3H), 1.89 − 1.76 (m, 2H), 1.73 − 1.62 (m, 5H), 1.48 − 1.36 (m, 1H), 1.28 − 1.12 (m, 3H), 1.05 − 0.90 (m, 2H). ESI-MS [M—H]^−^ = 357.1. HPLC t_*R*_ = 8.72 min.

#### Ethyl ((benzyloxy)­carbonyl)-l-phenylalaninate (**15e**)

Obtained from the reaction of benzyl carbonochloridate (0.24 mL, 1.68 mmol) and ethyl *L*-phenylalaninate • HCl (350 mg, 1.52 mmol) following general procedure A. Flash purification with petroleum ether/EtOAc (0−25% EtOAc). Yield: 462 mg (93%) of **15e** as a white solid. ^1^H NMR (400 MHz, CDCl_3_) δ 7.31 − 7.23 (m, 5H), 7.21 − 7.15 (m, 3H), 7.06 − 7.01 (m, 2H), 5.19 − 5.11 (m, 1H), 5.07 − 4.98 (m, 2H), 4.61 − 4.51 (m, 1H), 4.14 − 3.99 (m, 2H), 3.09 − 2.97 (m, 2H), 1.15 (t, *J* = 7.1 Hz, 3H). ESI-MS [M + Na]^+^ = 350.1. HPLC t_*R*_ = 8.54 min.

#### (*S*)-*N*-(1-Hydrazineyl-4-methyl-1-oxopentan-2-yl)-4-methoxy-1*H*-indole-2-carboxamide (**16a**)

Obtained from **15a** (318 mg, 1 mmol) following general procedure B. Yield: 318 mg (100%) of **16a** as a yellow solid. ^1^H NMR (400 MHz, DMSO-*d*_*6*_) δ 11.54 (s, 1H), 9.23 (s, 1H), 8.37 (d, *J* = 8.4 Hz, 1H), 7.34 (s, 1H), 7.13 − 7.07 (m, 1H), 7.01 (d, *J* = 8.2 Hz, 1H), 6.50 (d, *J* = 7.5 Hz, 1H), 4.55 − 4.46 (m, 1H), 4.31 − 4.10 (m, 2H), 3.88 (s, 3H), 1.74 − 1.59 (m, 2H), 1.53 − 1.45 (m, 1H), 0.91 (d, *J* = 6.4 Hz, 3H), 0.87 (d, *J* = 6.4 Hz, 3H). ESI-MS [M + Na]^+^ = 341.2. HPLC t_*R*_ = 6.65 min.

#### (*S*)-4,6-Difluoro-*N*-(1-hydrazineyl-4-methyl-1-oxopentan-2-yl)-1*H*-indole-2-carboxamide (**16b**)

Obtained from **15b** (324 mg, 1 mmol) following general procedure B. Yield: 324 mg (100%) of **16b** as a white solid. ^1^H NMR (400 MHz, DMSO-*d*_*6*_) δ 9.28 (s, 1H), 8.54 (d, *J* = 8.4 Hz, 1H), 7.42 − 7.37 (m, 1H), 7.06 − 6.99 (m, 1H), 6.92 − 6.83 (m, 1H), 5.75 (s, 1H), 4.56 − 4.43 (m, 1H), 4.34 − 4.11 (m, 2H), 1.73 − 1.58 (m, 2H), 1.56 − 1.45 (m, 1H), 0.91 (d, *J* = 6.4 Hz, 3H), 0.87 (d, *J* = 6.4 Hz, 3H). ESI-MS [M + Na]^+^ = 347.3. HPLC t_*R*_ = 7.55 min.

#### (*S*)-4,6-Dichloro-*N*-(1-hydrazineyl-4-methyl-1-oxopentan-2-yl)-1*H*-indole-2-carboxamide (**16c**)

Obtained from **15c** (357 mg, 1 mmol) following general procedure B. Yield: 357 mg (100%) of **16c** as a white solid. ^1^H NMR (400 MHz, DMSO-*d*_*6*_) δ 9.29 (s, 1H), 8.71 (d, *J* = 8.4 Hz, 1H), 7.48 − 7.41 (m, 2H), 7.26 − 7.20 (m, 1H), 5.65 − 5.41 (m, 1H), 4.57 − 4.48 (m, 1H), 4.38 − 4.09 (m, 2H), 1.73 − 1.59 (m, 2H), 1.55 − 1.45 (m, 1H), 0.91 (d, *J* = 6.4 Hz, 3H), 0.87 (d, *J* = 6.4 Hz, 3H). ESI-MS [M + Na]^+^ = 355.3. HPLC t_*R*_ = 9.04 min.

#### (*S*)-*N*-(3-Cyclohexyl-1-hydrazineyl-1-oxopropan-2-yl)-4-methoxy-1*H*-indole-2-carboxamide (**16d**)

Obtained from **15d** (358 mg, 1 mmol) following general procedure B. Yield: 358 mg (100%) of **16d** as a white solid. ^1^H NMR (400 MHz, DMSO-*d*_*6*_) δ 11.53 (s, 1H), 9.20 (s, 1H), 8.35 (d, *J* = 8.4 Hz, 1H), 7.34 (s, 1H), 7.15 − 7.06 (m, 1H), 7.02 (d, *J* = 8.3 Hz, 1H), 6.51 (d, *J* = 7.5 Hz, 1H), 4.59 − 4.48 (m, 1H), 4.37 − 4.09 (m, 2H), 3.89 (s, 3H), 1.78 − 1.69 (m, 2H), 1.69 − 1.62 (m, 3H), 1.61 − 1.49 (m, 2H), 1.40 − 1.28 (m, 1H), 1.25 − 1.05 (m, 3H), 1.00 − 0.81 (m, 2H). ESI-MS [M—H]^−^ = 357.1. HPLC t_*R*_ = 8.09 min.

#### Benzyl (*S*)-(1-hydrazineyl-1-oxo-3-phenylpropan-2-yl)­carbamate (**16e**)

Obtained from **15e** (461 mg, 1.41 mmol) following general procedure B. Yield: 442 mg (100%) of **16e** as a white solid. ^1^H NMR (400 MHz, DMSO-*d*_*6*_) δ 9.31 − 9.11 (m, 1H), 7.54 (d, *J* = 8.7 Hz, 1H), 7.36 − 7.28 (m, 3H), 7.28 − 7.14 (m, 7H), 4.99 − 4.87 (m, 2H), 4.23 − 4.15 (m, 1H), 3.17 (s, 2H), 2.94 − 2.87 (m, 1H), 2.76 (dd, *J* = 13.6, 10.4 Hz, 1H). ESI-MS [M + Na]^+^ = 336.1. HPLC t_*R*_ = 6.02 min.

#### (*S*)-*N*-(1-(2-(2,2-Dichloroacetyl)-2-((2-oxo-1,2-dihydropyridin-3-yl)­methyl)­hydrazineyl)-4-methyl-1-oxopentan-2-yl)-4-methoxy-1*H*-indole-2-carboxamide (**20a**)

Following general procedure E for the synthesis of amides, compound **20a** was synthesized using **19a** (100 mg, 0.24 mmol, 1 equiv.), 2,2-dichloroacetyl chloride (25 μL, 0.26 mmol, 1.1 equiv.), and NaHCO_3_ (61 mg, 0.72 mmol, 3 equiv.) in acetone (8 mL). After column chromatography (NP, CH_2_Cl_2_/CH_3_OH, v/v, 100:0−94:6), the pure product **20a** was isolated as a white solid (114 mg, 89%). ^1^H NMR (400 MHz, DMSO-*d*_*6*_) δ 11.73 (s, 1H), 11.62 − 11.52 (m, 1H), 10.98 − 10.59 (m, 1H), 8.56 (d, *J* = 6.1 Hz, 1H), 7.40 − 7.37 (m, 1H), 7.36 − 7.30 (m, 2H), 7.13 − 7.07 (m, 1H), 7.05 − 7.00 (m, 1H), 6.89 − 6.67 (m, 1H), 6.51 (d, *J* = 7.4 Hz, 1H), 6.21 − 6.10 (m, 1H), 4.87 − 4.69 (m, 1H), 4.51 − 4.26 (m, 1H), 4.15 − 3.98 (m, 1H), 3.88 (s, 3H), 1.78 − 1.62 (m, 2H), 1.60 − 1.36 (m, 1H), 0.95 − 0.88 (m, 3H), 0.88 − 0.82 (m, 3H). ^13^C NMR (101 MHz, DMSO-*d*_*6*_) δ 172.28, 165.54, 161.82, 161.73, 153.64, 139.41, 137.90, 134.73, 129.37, 124.92, 124.55, 118.04, 105.48, 104.58, 101.56, 99.23, 64.43, 55.07, 50.51, 47.78, 39.43, 24.29, 22.96, 21.33. TLC-MS (ESI) *m*/*z* for (C_24_H_27_Cl_2_N_5_O_5_ [M—H]^−^) calcd. 534.14, found 534.8. HRMS (ESI-TOF) *m*/*z* for (C_24_H_27_Cl_2_N_5_O_5_ [M+H]^+^) calcd. 536.1467, found 536.1472. HPLC t_*R*_ = 7.58 min. FT-IR (ATR) [cm^−1^] 3090, 2957, 2927, 1681, 1662, 1610, 1534, 1256, 1102, 753.

#### (*S*)-*N*-(1-(2-((5-Chloro-2-oxo-1,2-dihydropyridin-3-yl)­methyl)-2-(2,2-dichloroacetyl)­hydrazineyl)-4-methyl-1-oxopentan-2-yl)-4-methoxy-1*H*-indole-2-carboxamide (**20b**)

Following general procedure E for the synthesis of amides, compound **20b** was synthesized using **19b** (200 mg, 0.43 mmol, 1 equiv.), 2,2-dichloroacetyl chloride (46 μL, 0.48 mmol, 1.1 equiv.), and NaHCO_3_ (108 mg, 1.29 mmol, 3 equiv.) in acetone (8 mL). After column chromatography (NP, CH_2_Cl_2_/CH_3_OH, v/v, 100:0−96.2:3.8), the pure product **20b was isolated** as an off white solid (190 mg, 77%). ^1^H NMR (400 MHz, DMSO-*d*_*6*_) δ 12.04 (s, 1H), 11.67 − 11.48 (m, 1H), 11.09 − 10.64 (m, 1H), 8.70 − 8.51 (m, 1H), 7.72 − 7.54 (m, 1H), 7.45 − 7.29 (m, 2H), 7.17 − 7.07 (m, 1H), 7.07 − 7.00 (m, 1H), 6.94 − 6.69 (m, 1H), 6.51 (d, *J* = 7.6 Hz, 1H), 4.86 − 4.65 (m, 1H), 4.44 − 4.24 (m, 1H), 4.23 − 4.05 (m, 1H), 3.89 (s, 3H), 1.83 − 1.60 (m, 2H), 1.60 − 1.37 (m, 1H), 0.98 − 0.90 (m, 3H), 0.88 (d, *J* = 4.3 Hz, 3H). ^13^C NMR (101 MHz, DMSO-*d*_*6*_) δ 172.33, 161.82 (2C), 160.38, 153.65, 139.37, 137.92, 129.32, 124.58 (2C), 118.04 (2C), 105.50, 101.67, 101.65, 99.24, 64.33, 59.75, 55.08 (2C), 39.63, 24.29, 22.92, 21.37. TLC-MS (ESI) *m*/*z* for (C_24_H_26_Cl_3_N_5_O_5_ [M—H]^−^) calcd. 568.10, found 568.3. HPLC t_*R*_ = 8.05 min. HRMS (ESI-TOF) *m*/*z* for (C_24_H_26_Cl_3_N_5_O_5_ [M+Na]^+^) calcd. 592.0897, found 592.0890. FT-IR (ATR) [cm^−1^] 3268, 2957, 1654, 1607, 1537, 1249, 1090, 755, 650.

#### (*S*)-*N*-(1-(2-((5-Fluoro-2-oxo-1,2-dihydropyridin-3-yl)­methyl)-2-(2,2-dichloroacetyl)­hydrazineyl)-4-methyl-1-oxopentan-2-yl)-4-methoxy-1*H*-indole-2-carboxamide (**20c**)

Following general procedure E for the synthesis of amides, compound **20c** was synthesized using **19c** (155 mg, 0.35 mmol), 2,2-dichloroacetyl chloride (37 μL, 0.38 mmol), and NaHCO_3_ (88 mg, 1.05 mmol, 3 equiv.) in acetone (8 mL). After column chromatography (NP, CH_2_Cl_2_/CH_3_OH, v/v, 100:0−96.2:3.8), the pure product **20c** was isolated as a white solid (112 mg, 58%). ^1^H NMR (400 MHz, DMSO-*d*_*6*_) δ 11.70 (s, 1H), 11.64 − 11.54 (m, 1H), 11.10 − 10.63 (m, 1H), 8.69 − 8.51 (m, 1H), 7.66 − 7.52 (m, 1H), 7.51 − 7.43 (m, 1H), 7.43 − 7.35 (m, 1H), 7.16 − 7.07 (m, 1H), 7.03 (d, *J* = 8.2 Hz, 1H), 6.95 − 6.71 (m, 1H), 6.51 (d, *J* = 7.5 Hz, 1H), 4.76 (d, *J* = 15.0 Hz, 1H), 4.45 − 4.26 (m, 1H), 4.16 (d, *J* = 15.2 Hz, 1H), 3.89 (s, 3H), 1.79 − 1.63 (m, 2H), 1.57 − 1.40 (m, 1H), 0.90 (d, *J* = 5.8 Hz, 3H), 0.88 (d, *J* = 5.8 Hz, 3H). ^13^C NMR (101 MHz, DMSO-*d*_*6*_) δ 172.74, 162.27, 160.28, 160.26, 154.15, 154.12, 138.40, 129.82, 125.05 (2C), 118.52 (2C), 105.97, 102.10, 99.75, 99.72, 64.79, 55.55 (2C), 49.07, 39.90, 24.76, 23.36, 21.85. TLC-MS (ESI) *m*/*z* for (C_24_H_26_Cl_2_FN_5_O_5_ [M—H]^−^) calcd. 552.13, found 552.4. HRMS (ESI-TOF) *m*/*z* for (C_24_H_26_Cl_2_FN_5_O_5_ [M+Na]^+^) calcd. 576.1193, found 576.1185. HPLC t_*R*_ = 7.21 min. FT-IR (ATR) [cm^−1^] 3253, 2961, 1619, 1545, 1253, 1101, 809, 759.

#### (*S*)-*N*-(1-(2-((5-Methyl-2-oxo-1,2-dihydropyridin-3-yl)­methyl)-2-(2,2-dichloroacetyl)­hydrazineyl)-4-methyl-1-oxopentan-2-yl)-4-methoxy-1*H*-indole-2-carboxamide (**20d**)

Following general procedure E for the synthesis of amides, compound **20d** was synthesized using **19d** (138 mg, 0.31 mmol), 2,2-dichloroacetyl chloride (33 μL, 0.35 mmol), and NaHCO_3_ (78 mg, 0.93 mmol, 3 equiv.) in acetone (8 mL). After column chromatography (NP, CH_2_Cl_2_/CH_3_OH, v/v, 100:0−96.2:3.8), the pure white product **20d** was isolated as a white solid (156 mg, 91%). ^1^H NMR (400 MHz, DMSO-*d*_*6*_) δ 11.57 (s, 1H), 11.54 (s, 1H), 10.99 − 10.56 (m, 1H), 8.62 − 8.50 (m, 1H), 7.44 − 7.34 (m, 1H), 7.21 (d, *J* = 2.3 Hz, 1H), 7.17 − 7.13 (m, 1H), 7.13 − 7.09 (m, 1H), 7.03 (d, *J* = 8.2 Hz, 1H), 6.89 − 6.67 (m, 1H), 6.51 (d, *J* = 7.4 Hz, 1H), 4.78 (d, *J* = 14.5 Hz, 1H), 4.46 − 4.28 (m, 1H), 4.12 − 4.03 (m, 1H), 3.89 (s, 3H), 1.99 (s, 3H), 1.76 − 1.63 (m, 2H), 1.46 − 1.33 (m, 1H), 0.90 (d, *J* = 6.3 Hz, 3H), 0.86 (d, *J* = 5.1 Hz, 3H). ^13^C NMR (101 MHz, DMSO-*d*_*6*_) δ 172.80, 165.99, 162.22, 161.49, 154.12, 142.51, 138.38, 132.43, 129.86, 125.03, 124.69, 118.51, 113.41, 105.96, 102.05, 99.71, 64.90, 60.22, 55.54 (2C), 39.90, 24.74, 23.43, 21.80, 16.86. TLC-MS (ESI) *m*/*z* for (C_25_H_29_Cl_2_N_5_O_5_ [M—H]^−^) calcd. 548.15, found 548.5. HRMS (ESI-TOF) *m*/*z* for (C_25_H_29_Cl_2_N_5_O_5_ [M+Na]^+^) calcd. 572.1443, found 572.1439. HPLC t_*R*_ = 7.26 min. FT-IR (ATR) [cm^−1^] 3261, 2953, 1654, 1623, 1541, 1358, 1253, 1097, 809, 763

#### (*S*)-*N*-(1-(2-(2,2-Dichloroacetyl)-2-((2-oxo-5-(trifluoromethyl)-1,2-dihydropyridin-3-yl) methyl)­hydrazineyl)-4-methyl-1-oxopentan-2-yl)-4-methoxy-1*H*-indole-2-carboxamide (**20e**)

Following general procedure E for the synthesis of amides, compound **20e** was synthesized using **19e** (168 mg, 0.34 mmol, 1 equiv.), 2,2-dichloroacetyl chloride (36 μL, 0.37 mmol, 1.1 equiv.), and NaHCO_3_ (86 mg, 1.02 mmol, 3 equiv.) in acetone (8 mL). After column chromatography (NP, CH_2_Cl_2_/CH_3_OH, v/v, 100:0−96.2:3.8), the pure product **20e** was isolated as a white solid (188 mg, 91%). ^1^H NMR (400 MHz, DMSO-*d*_*6*_) δ 12.37 (s, 1H), 11.68 − 11.49 (m, 1H), 11.13 − 10.64 (m, 1H), 8.66 − 8.50 (m, 1H), 8.03 − 7.89 (m, 1H), 7.64 − 7.49 (m, 1H), 7.47 − 7.32 (m, 1H), 7.15 − 7.07 (m, 1H), 7.03 (d, *J* = 8.2 Hz, 1H), 6.96 − 6.71 (m, 1H), 6.51 (d, *J* = 7.2 Hz, 1H), 4.80 (d, *J* = 14.9 Hz, 1H), 4.46 − 4.27 (m, 1H), 4.19 (d, *J* = 14.8 Hz, 1H), 3.89 (s, 3H), 1.78 − 1.63 (m, 2H), 1.43 − 1.29 (m, 1H), 0.90 (d, *J* = 5.9 Hz, 3H), 0.86 (d, *J* = 5.6 Hz, 3H). ^13^C NMR (101 MHz, DMSO-*d*_*6*_) δ 172.87, 170.80, 162.32, 161.91, 154.12, 138.40, 135.77, 134.71, 129.78, 126.66, 125.06, 124.32 (d, *J* = 269.3 Hz), 118.51, 107.41, 105.97, 102.13, 99.71, 64.80, 60.22, 55.54 (2C), 39.68, 24.72, 23.32, 21.69. TLC-MS (ESI) *m*/*z* for (C_25_H_26_Cl_2_F_3_N_5_O_5_ [M—H]^−^) calcd. 602.13, found 602.6. HPLC t_*R*_ = 7.96 min. FT-IR (ATR) [cm^−1^] 3253, 2957, 1662, 1619, 1545, 1331, 1242, 1121, 809, 759.

#### (*S*)-*N*-(1-(2-(2,2-Dichloroacetyl)-2-((2-oxo-1,2-dihydropyridin-3-yl)­methyl)­hydrazineyl)-4-methyl-1-oxopentan-2-yl)-4,6-difluoro-1*H*-indole-2-carboxamide (**20f**)

Following general procedure E for the synthesis of amides, compound **20f** was synthesized using **19f** (137 mg, 0.32 mmol, 1 equiv.), 2,2-dichloroacetyl chloride (34 μL, 0.35 mmol, 1.1 equiv.), and NaHCO_3_ (81 mg, 0.96 mmol, 3 equiv.) in acetone (8 mL). After column chromatography (NP, CH_2_Cl_2_/CH_3_OH, v/v, 100:0−94:6), the pure product **20f** was isolated as a white solid (161 mg, 93%). ^1^H NMR (400 MHz, DMSO-*d*_*6*_) δ 12.09 − 11.96 (m, 1H), 11.73 (s, 1H), 11.02 − 10.58 (m, 1H), 8.73 (d, *J* = 6.1 Hz, 1H), 7.44 − 7.38 (m, 1H), 7.38 − 7.28 (m, 2H), 7.08 − 7.01 (m, 1H), 6.93 − 6.85 (m, 1H), 6.85 − 6.65 (m, 1H), 6.21 − 6.09 (m, 1H), 4.87 − 4.69 (m, 1H), 4.53 − 4.27 (m, 1H), 4.16 − 3.98 (m, 1H), 1.77 − 1.61 (m, 2H), 1.59 − 1.34 (m, 1H), 0.95 − 0.88 (m, 3H), 0.89 − 0.82 (m, 3H). ^13^C NMR (101 MHz, DMSO-*d*_*6*_) δ 172.58, 162.31, 161.51, 161.04, 156.33 (d, *J* = 249.1 Hz), 156.18 (d, *J* = 249.1 Hz), 139.99, 138.22, 135.32, 132.33, 125.36, 113.47, 105.07, 100.06, 95.71, 95.03, 64.84, 55.38, 51.05, 42.78, 24.73, 23.41, 21.79. TLC-MS (ESI) *m*/*z* for (C_23_H_23_Cl_2_F_2_N_5_O_4_ [M—H]^−^) calcd. 540.11, found 540.7. HPLC t_*R*_ = 8.51 min. FT-IR (ATR) [cm^−1^] 3267, 2956, 2871, 1636, 1540, 1276, 1215, 1120, 807, 766.

#### (*S*)-4,6-Dichloro-*N*-(1-(2-(2,2-dichloroacetyl)-2-((2-oxo-1,2-dihydropyridin-3-yl)­methyl) hydrazineyl)-4-methyl-1-oxopentan-2-yl)-1*H*-indole-2-carboxamide (**20g**)

Following general procedure E for the synthesis of amides, compound **20g** was synthesized using **19g** (220 mg, 0.47 mmol, 1 equiv.), 2,2-dichloroacetyl chloride (50 μL, 0.52 mmol, 1.1 equiv.), and NaHCO_3_ (118 mg, 1.41 mmol, 3 equiv.) in acetone (8 mL). After column chromatography (NP, CH_2_Cl_2_/CH_3_OH, v/v, 100:0−94.5:5.5), the pure product **20g** was isolated as a white solid (150 mg, 56%). ^1^H NMR (400 MHz, DMSO-*d*_*6*_) δ 12.10 (s, 1H), 11.85 − 11.63 (m, 1H), 11.03 − 10.60 (m, 1H), 8.88 (d, *J* = 6.4 Hz, 1H), 7.50 − 7.41 (m, 2H), 7.35 (d, *J* = 6.5 Hz, 2H), 7.29 − 7.21 (m, 1H), 6.88 − 6.62 (m, 1H), 6.24 − 6.07 (m, 1H), 4.92 − 4.71 (m, H), 4.55 − 4.30 (m, 1H), 4.19 − 3.99 (m, 1H), 1.82 − 1.62 (m, 2H), 1.60 − 1.35 (m, 1H), 0.97 − 0.90 (m, 3H), 0.88 (d, *J* = 3.8 Hz, 3H). ^13^C NMR (101 MHz, DMSO-*d*_*6*_) δ 172.50, 165.90, 162.30, 161.47, 140.00, 139.80, 137.38, 135.27, 128.40, 126.91, 125.36, 125.20, 120.00, 111.69, 105.05, 102.45, 64.84, 55.38, 51.13, 40.13, 24.73, 23.43, 21.75. TLC-MS (ESI) *m*/*z* for (C_23_H_23_Cl_4_N_5_O_4_ [M+H]^+^) calcd. 574.05, found 573.8. HRMS (ESI-TOF) *m*/*z* for (C_23_H_23_Cl_4_N_5_O_4_ [M+H]^+^) calcd. 574.0582, found 574.0582. HPLC t_*R*_ = 9.24 min. FT-IR (ATR) [cm^−1^] 3167, 3093, 2961, 1693, 1654, 1619, 1537, 1245, 841, 755.

#### (*S*)-*N*-(3-cyclohexyl-1-(2-(2,2-dichloroacetyl)-2-((2-oxo-1,2-dihydropyridin-3-yl)­methyl) hydrazineyl)-1-oxopropan-2-yl)-4-methoxy-1*H*-indole-2-carboxamide (**20h**)

Following general procedure E for the synthesis of amides, compound **20h** was synthesized using **19h** (153 mg, 0.33 mmol, 1 equiv.), 2,2-dichloroacetyl chloride (35 μL, 0.36 mmol, 1.1 equiv.), and NaHCO_3_ (83 mg, 0.99 mmol, 3 equiv.) in acetone (8 mL). After column chromatography (NP, CH_2_Cl_2_/CH_3_OH, v/v, 100:0−94.5:5.5), the pure product **20h** was isolated as a white solid (165 mg, 87%). ^1^H NMR (400 MHz, DMSO-*d*_*6*_) δ 11.73 (s, 1H), 11.63 − 11.53 (m, 1H), 11.02 − 10.59 (m, 1H), 8.62 − 8.50 (m, 1H), 7.44 − 7.37 (m, 1H), 7.34 (d, *J* = 6.6 Hz, 2H), 7.14 − 7.06 (m, 1H), 7.03 (d, *J* = 8.2 Hz, 1H), 6.93 − 6.64 (m, 1H), 6.51 (d, *J* = 7.4 Hz, 1H), 6.21 − 6.10 (m, 1H), 4.88 − 4.70 (m, 1H), 4.54 − 4.27 (m, 1H), 4.18 − 4.06 (m, 1H), 3.89 (s, 3H), 1.77 − 1.67 (m, 2H), 1.67 − 1.54 (m, 4H), 1.53 − 1.41 (m, 1H), 1.40 − 1.28 (m, 1H), 1.23 − 1.04 (m, 3H), 1.00 − 0.78 (m, 2H). ^13^C NMR (101 MHz, DMSO-*d*_*6*_) δ 172.35, 165.65, 161.79, 161.71, 153.63, 139.03, 137.89, 134.64, 129.35, 124.93, 124.55, 118.04, 105.48, 104.57, 101.57, 99.22, 64.39, 55.06, 49.84, 48.59, 37.46, 33.50, 33.03, 31.71, 26.02, 25.61, 25.49. TLC-MS (ESI) *m*/*z* for (C_27_H_31_Cl_2_N_5_O_5_ [M—H]^−^) calcd. 574.17, found 574.3. HRMS (ESI-TOF) *m*/*z* for (C_27_H_31_Cl_2_N_5_O_5_ [M+H]^+^) calcd. 576.1780, found 581.1127. HPLC t_*R*_ = 8.25 min. FT-IR (ATR) [cm^−1^] 3167, 2922, 2844, 1642, 1607, 1537, 1253, 1101, 755.

#### Benzyl (*S*)-(1-(2-(2,2-dichloroacetyl)-2-((2-oxo-1,2-dihydropyridin-3-yl)­methyl)­hydrazineyl)-1-oxo-3-phenylpropan-2-yl)­carbamate (**20i**)

Following general procedure E for the synthesis of amides, compound **20i** was synthesized using **19i** (102 mg, 0.24 mmol, 1 equiv.), 2,2-dichloroacetyl chloride (26 μL, 0.27 mmol, 1.1 equiv.), and NaHCO_3_ (61 mg, 0.72 mmol, 3 equiv.) in acetone (8 mL). After column chromatography (NP, CH_2_Cl_2_/CH_3_OH, v/v, 100:0−94.5:5.5), the pure product **20i was isolated** as a white solid (107 mg, 84%). ^1^H NMR (400 MHz, DMSO-*d*_*6*_) δ 11.73 (s, 1H), 11.03 − 10.66 (m, 1H), 7.96 − 7.71 (m, 1H), 7.47 − 7.31 (m, 4H), 7.31 − 7.21 (m, 7H), 7.21 − 7.11 (m, 1H), 6.87 − 6.54 (m, 1H), 6.16 (t, *J* = 6.6 Hz, 1H), 5.12 − 4.91 (m, 2H), 4.88 − 4.66 (m, 1H), 4.36 − 4.13 (m, 1H), 4.06 − 3.86 (m, 1H), 3.10 − 2.72 (m, 2H). ^13^C NMR (101 MHz, DMSO-*d*_*6*_) δ 172.17, 165.88, 162.30, 162.26, 139.38, 137.82, 137.19, 135.09, 129.61 (2C), 128.80 (2C), 128.75, 128.62, 128.30, 128.28, 128.19, 126.93, 125.49, 105.08, 66.08, 64.71, 55.42, 55.38, 39.91. TLC-MS (ESI) *m*/*z* for (C_25_H_24_Cl_2_N_4_O_5_ [M + Na]^+^) calcd. 553.11, found 552.9. HPLC t_*R*_ = 7.54 min. FT-IR (ATR) [cm^−1^] 3268, 3031, 2938, 1685, 1646, 1607, 1502, 1245, 1054, 393.

## Biological Assays and Biophysical Studies

### Protein Purification

The g-block of the SARS-CoV-2 M^pro^ gene was codon-optimized and synthesized from IDT (Integrated DNA Technologies). The M^pro^ g-block was cloned into the pE-SUMO expression vector using BsaI restriction sites. The *N*-terminal 6XHis-Sumo fusion tag M^pro^ was overexpressed in the *E. coli* BL21­(DE3) strain (New England Biolabs catalog no. C2527H). For protein production, a single colony was grown overnight to saturation in 25 mL Luria Broth medium supplemented with 100 mg/mL carbenicillin (Thermo Fisher Scientific catalog no. J6194903). 1% of this primary culture was used to inoculate 1 L of Luria Broth (LB) supplemented with 100 mg/mL of carbenicillin and incubated at 37 °C and 180 rpm Once the optical density (OD) reached 0.6. The culture was induced with 0.5 mM IPTG (Thermo Fisher Scientific catalog no. 15529019), and the temperature was lowered to 18 °C for an additional 20 h. The cells were collected by centrifugation at 6,000 g, resuspended in 50 mM Tris, pH 8.0, 250 mM NaCl, 5 mM β-mercaptoethanol (Thermo Fisher Scientific catalog no. O33461−100), 5 mM imidazole (Thermo Fisher Scientific catalog no. A1022122), and 5% glycerol (Thermo Fisher Scientific catalog no. A16205AP), and lysed by sonication. M^pro^ was captured from cleared lysate using a nickel-nitrilotriacetic acid gravity flow affinity column (Fisher Scientific catalog no R90115), washed with a gradient of imidazole, and eluted with 300 mM imidazole. The eluted fraction with M^pro^ was pooled together and treated with Ulp1 Sumo-protease to remove the 6XHis-Sumo tag and further purified by gel filtration using HiLoad 26/600 Superdex 200 pg (Cytvia Life Sciences catalog no. 28989336) in 20 mM Tris-HCl, pH 8.0, 150 mM NaCl, 0.5 mM TCEP. The peak fractions showing M^pro^ in SDS-PAGE were pooled and concentrated to 15 mg/mL as determined by UV absorbance (NanoDrop 8000 spectrophotometer).

### Cells and Viruses

VeroE6 cells were obtained from the American Type Culture Collection (ATCC) (CRL-1586) (Manassas, VA). They were maintained in Dulbecco’s modified Eagle’s medium (d-MEM) supplemented with 10% fetal bovine serum (FCS), 100 μg/mL of penicillin, and 100 μg/mL of streptomycin. HeLahACE2-TMPRSS2 cells were obtained from the Japanese Collection of Research Bioresources (JCRB) Cell Bank (JCRB1835, Osaka, Japan). They were maintained in the same conditioned medium as the VeroE6 cell line except for G418 (0.5 mg/mL). Those cells were regularly tested and confirmed to be negative for mycoplasma contamination by using PCR. A SARS-CoV-2 strain JPN/TY/WK-521/2020 (SARS-CoV-2^WK521^) was obtained from the National Institute of Infectious Diseases (Tokyo, Japan), and an Omicron BQ.1.1 strain (GISAID Accession ID; EPI_ISL_15579783) (SARS-CoV-2BQ. 1.1) was provided by the Tokyo Metropolitan Institute of Public Health, Tokyo, Japan. Two recombinant SARS-CoV-2 variants, a wild-type SARS-CoV-2 (rgSARS-CoV-2WT) and a SARS-CoV-2 carrying an E166V mutation in the Nsp5 (rgSARS-CoV-2E166V), were generated as previously described.^46,69,70^

### Antiviral Activity and Cytotoxicity Assays

Two clinical isolates, SARS-CoV-2^WK521^ and SARS-CoV-2^BQ.1.1,^ were examined for their anti-SARS-CoV-2 activity using VeroE6 and HeLaACE2-TMPRSS2 cells, respectively. At the same time, two recombinant SARS-CoV-2 variants, rgSARS-CoV-2WT and rgSARS-CoV-2E166V, were analyzed using VeroE6 cells in the presence of P-glycoprotein inhibitor, CP-100356 (efflux inhibitor, EI) (Sigma-Aldrich, Co. LLC). Cells were seeded in a 96-well plate (2 × 10^4^ cells/well) and incubated for 1 day, then the virus was inoculated onto the culture at multiplicities of infection (MOI) of 80 for SARS-CoV-2^WK-521^ and SARS-CoV-2^BQ.1.1^ and 100 for rgSARS-CoV-2WT and rgSARS-CoV-2E166V. One hour post-viral exposure, the virus was removed, and the cells were washed once with a culture medium and incubated for 3−4 days with each drug solution. After incubation, culture supernatants were harvested, and viral RNA was extracted using a QIAamp viral RNA mini kit (Qiagen, Hilden, Germany). RT-qPCR was performed using One Step PrimeScript III RT-qPCR mix (TaKaRa Bio, Shiga, Japan) and a 7500 Fast Real-Time PCR Instrument (Applied Biosystems, Waltham, MA, USA) according to the manufacturer’s instructions. The primers and probe used for detecting SARS-CoV-2 nucleocapsid were 5′-AAATTTTGGGGAC-CAGGAAC-3′ (forward), 5′-TGGCAGCTGTGTAGGTCAAC-3′ (reverse), and 5′-FAM-ATGTCGCGCATTGGCATGGA-black hole quencher (BHQ1)-3′ (probe). Each 50% effective concentration (EC_50_) value was calculated as previously described.^42^ To determine the cytotoxicity of each compound, cells were seeded in a 96-well plate (2 × 10^4^ cells/well). One day later, various concentrations of each compound were added, and the cells were incubated for 3 days. The values of 50% cytotoxic concentrations (CC_50_) were determined using the WST-8 assay and Cell Counting Kit-8 (Dojindo, Kumamoto, Japan).

### Cell Line and Virus

The Vero AT cells (BEI, #NR-54970) were used to assess the CPE. This study used an attenuated strain of SARS-CoV-2 containing a deletion of ORF3a and ORF7b (rSARS-CoV-2 Δ3a/7b)^48^

### Measurement of Antiviral Activity

The antiviral activity was measured by inhibiting virus-induced CPE by adding compounds to confluent Vero AT cells in 96-well microplates. After 3 h, the viral suspensions of the live attenuated SARS-CoV-2 strain (120 PFU/well) were added, resulting in a final concentration of 20 μM, achieved through a serial dilution (5-fold) over 10 experimental points. At 4 days postinfection, Cell-Titer Glo-based readout (according to the manufacturer’s protocol) was used to measure the number of viable cells. The readouts were normalized and plotted using GraphPad Prism software. In parallel, sets of cells were fixed and stained with 0.5% violet crystal solution for 5 min and imaged using a LiCor imager. For CPE-based experiments, the mean values ± SD are derived from 2 to 6 experimental replicates.

### MERS-CoV M^pro^ Enzymatic Inhibition Assays

The potential inhibitory activity of the compounds against MERS-CoV M^pro^ (R&D Systems, E719) was assessed as described.^67^ The assay buffer consisted of 50 mM HEPES (pH 7.5), 150 mM NaCl, 1 mM EDTA, and 0.01% Tween 20. The initial screening was performed after a 15-min preincubation of the enzyme (200 nM) with 20 μM of each compound in 384-well black microplates. Then, the fluorogenic substrate Ac-Abu-Tle-Leu-Gln-AMC (Biosynth, FA178674) was added (40 μM), starting the reaction. The final compounds, enzyme, and substrate concentrations were 10 μM, 50 nM, and 10 μM, respectively, and the final DMSO concentration was up to 0.1% at a total volume of 20 μL. The fluorescence was monitored using a Synergy HTX (Biotek, US) microplate reader at 37 °C for 2 h, with excitation and emission wavelengths of 360 and 460 nm, respectively. NMV (100 nM) was used as a positive control inhibitor. Enzymatic activity was normalized to the wells that lacked compounds or the inhibitor (DMSO controls), to calculate the inhibition percentages. A concentration−response inhibition assay was performed for compounds that inhibited enzyme activity by≥ 80%, which was then followed by a six-point concentration−response assay (0.001−5 μM). The half-maximal inhibitory concentration (IC_50_) values were calculated by nonlinear regression, and the deviation of each data point from the calculated nonlinear regression was less than 10%. Data were analyzed using GraphPad Prism 9.0 (GraphPad Software, San Diego, California, US). All conditions were tested in triplicate in two or more independent assays (*n* ≥ 6 data points).

### Human Cathepsin L (catl) Inhibition Assays

To understand whether another essential cysteine protease for the SARS-CoV-2 cycle, the human CatL, could be inhibited, the hits obtained for MERS-CoV M^pro^ were preincubated at 20 μM for 15 min with 160 pM of recombinant enzyme (R&D Systems, 952-CY-010) in an assay buffer consisting of 40 mM sodium acetate at pH 5.5, 100 mM NaCl, 5 mM DTT, 1 mM EDTA, and 0.001% bovine serum albumin (BSA). Assays were performed in 384-well black microplates using 40 μM of the fluorogenic substrate Z-FR-AMC (Bachem, I-1160) as described.^71–73^ Enzyme and substrate were diluted with an equal volume of the same buffer (total volume of 10 μL) with 10 μL of compounds. Catalysis was measured up to 1 h at 25 °C using the Synergy HTX (360/460 nm). The final enzyme, substrate, and compound concentrations were 40 pM, 10 μM, and 10 μM, respectively. The cysteine protease inhibitor E64 (Research Products International, IL, USA) was used as a positive control (100 nM). Two independent assays in triplicate were performed (*n* = 6 data points). Data were analyzed using GraphPad Prism 9.0 (GraphPad Software, San Diego, California, USA).

### M^pro^ Cocrystallization

SARS-CoV-2 M^pro^ cocrystallization with **20a** compound was carried out using the sitting drop vapor diffusion method using an Art Robbins Phoenix Robot in the Structural Biology Core at the University of Texas Health Science Center at San Antonio. M^pro^ was prepared as mentioned in the protein purification section. One mM of **20a** compound was mixed with 15 mg/mL M^pro^ and incubated on ice for 1 h. Before setting up the crystallization tray, the complex was centrifuged at 12000 rpm for 10 min to remove any precipitate. The M^pro^-**20a** complex was mixed with the crystal screen in a 1:1 ratio, resulting in a total drop volume of 0.8 μL. The M^pro^ crystal was grown at 20 °C in Molecular Dimension Morpheus E5 conditions (10% w/v PEG 20000, 20% v/v PEG MME 550, 0.12 M ethylene glycol, 0.1 M MOPS/HEPES-Na pH 7.5). Crystals were flash-cooled in liquid nitrogen and mounted for data collection. Diffraction data was processed using AUTOPROC.^74^ The structure was determined by the molecular replacement method implemented in PHASER^75^ using coordinates from PDB entry 8HOL^76^ as the search model. COOT^77^ and PHENIX^78^ were used for iterative model building and refinement, respectively. The model was verified using composite omit map analysis.^79^ Data collection and refinement statistics are shown in Table S1. COOT and PyMOL were used to generate images for the crystal structure (The PyMOL Molecular Graphics System, Version 2.2, Schrödinger, LLC.).

### ITC Measurements

The experiments were performed on a MicroCal PEAQ-ITC (Malvern Panalytical, UK) at 25 °C. The M^pro^ without any tag was diluted in ITC buffer (20 mM Tris-HCl, 8.0, 100 mM NaCl, 0.5 mM TCEP, and 1.5% DMSO) to a final concentration of 10 μM. 300 μL of 10 μM M^pro^ was placed in a calorimetric cell, and the syringe was loaded with 70 μL of 150 μM of **20a** compound. The reference cell was filled with 300 μL of Milli-Q water. Twenty-five injections of 1.5 μL of **20a** were made into the cell at intervals of 150 s with a stirring speed of 750 rpm. GC-376 (covalent) and ensitrelvir (noncovalent) were used as positive control. A similar experiment was performed with 300 μM GC-376 and 300 μM ensitrelvir. To measure the binding affinity (*K*_D_), enthalpy (ΔH), entropy (ΔS), and stoichiometry (n), the raw ITC data were analyzed using MicroCal PEAQ-ITC analysis software.

## Supplementary Material







## Data Availability

The crystal structure has been deposited in the Protein Data Bank (www.rcsb.org) with the accession number 9MDQ (SARS-CoV-2 M^pro^/**20a** complex). All other data are available in the manuscript or the Supporting Information.

## Notes

2*mF*_o_-*DF*_c_ electron density map contoured at 1.0 r.m.s.d (Figure S1), antiviral activity of selected M^pro^ inhibitors (Figure S2), X-ray data collection and refinement statistics (Table S1), and ^1^H NMR, ^13^NMR, HRMS, and HPLC traces (PDF)
Molecular formula strings and biological data of final compounds (CSV)
CCDC file for **20a** (PDB)

## Abbreviations

ACE2: angiotensin converting enzyme 2
ATCC: American Type Culture Collection
CatL: human cathepsin L
COVID-19: coronavirus disease 2019
CoV: coronavirus
CC_50_: 50% cytotoxic concentrations;
DIPEA: diisopropylethylamine;
CL^pro^: chymotrypsin-like protease
CYP3A4: human cytochrome P450 3A4
Cys145: cysteine 145
CPE: cytopathic effect
EDTA: 2,2′,2″,2‴-(ethane-1,2-diyldinitrilo)­tetraacetic acid
EC_50_: 50% effective concentration
ESI-MS: electrospray ionization mass spectrometry
FRET: fluorescence resonance transfer
FCS: fetal bovine serum
GSH: glutathione
HPLC: high-pressure liquid chromatography
HCoV-NL-63: Human coronavirus NL63
HIV: human immunodeficiency virus
HATU: *O*-(7-azabenzotriazol-1-yl)-*N*,*N*,*N*′,*N*′-tetramethyluronium hexafluorophosphate
HOBt: hydroxybenzotriazole
ITC: isothermal titration calorimetry
JCRB: Japanese Collection of Research Bioresources
MOPS: 3-(morpholin-4-yl)­propane-1-sulfonic acid
M^pro^: main protease
MERS: Middle East respiratory syndrome
NMR: nuclear magnetic resonance
NSP: nonstructural protein
NMV: nirmatrelvir
ESV: ensitrelvir
PBC: periodic boundary condition
PDB: Protein Data Bank
PFU: plaque forming units
PL^pro^: papain-like protease
PME: Particle Mesh Ewald
PPB: plasma protein binding
RdRp: RNA-dependent RNA polymerase
RMSD: root-mean-square deviation
S: spike glycoprotein
SD: standard deviation
SAR: structure−activity relationship
SARS: severe acute respiratory syndrome
SARS-CoV: severe acute respiratory syndrome-related coronavirus
TEA: triethylamine
THF: tetrahydrofuran
TLC: thin-layer chromatography
T3P: 2,4,6-tripropyl-1,3,5,2,4,6-trioxatriphosphinane 2,4,6-trioxide
TMPRSS2: transmembrane protease serine subtype 2
TüKIC: Tübingen Kinase Inhibitor Collection
WT: wild type
