# Supplementary material for: Azapeptide-Based SARS-CoV-2 Main Protease Inhibitors: Design, Synthesis, Enzyme Inhibition, Structural Determination, and Antiviral Activity
Source: J Med Chem. 2025 Sep 11;68(18):19339–76. doi: 10.1021/acs.jmedchem.5c01520 (PMC12481482; doi:10.1021/acs.jmedchem.5c01520)
Supplement: Supplementary file 1 [file jm5c01520_si_001.pdf]

SUPPORTING INFORMATION

Azapeptide-based SARS-CoV-2 Main Protease Inhibitors: Design, Synthesis, Enzyme Inhibition, Structural Determination, and Antiviral Activity

Philipp Flury[a,b], Jyoti Vishwakarma[c], Katharina Sylvester[d], Nobuyo Higashi-Kuwata[e], Agnieszka K. Dabrowska[c], Renee Delgado[c], Ashley Cuell[c], Rahul Basu[c], Alexander B. Taylor[c,f], Ellen Gonçalves de Oliveira[g,h], Mateus Sá Magalhães Serafim[g,h] , Jingxin Qiao[i], Yan Chen[i], Shengyong Yang[i], Anthony J. O'Donoghue[h], Hiroaki Mitsuya[e], Michael Gütschow[d], Stefan A. Laufer[a,b], Christa E. Müller [d], Reuben S. Harris[c,j] and Thanigaimalai Pillaiyar[a,b,*]

[a]Institute of Pharmaceutical Sciences, Department of Pharmaceutical and Medicinal Chemistry, Eberhard Karls University Tübingen, Auf der Morgenstelle 8, 72076 Tübingen, Germany

[b]Tübingen Center for Academic Drug Discovery & Development (TüCAD$_2$), Eberhard Karls University Tübingen, Auf der Morgenstelle 8, 72076 Tübingen, Germany

[c]Department of Biochemistry and Structural Biology, University of Texas Health Science Center at San Antonio, San Antonio, Texas, USA, 78229

[d]PharmaCenter Bonn, Pharmaceutical Institute, Pharmaceutical & Medicinal Chemistry, University of Bonn, An der Immenburg 4, 53121 Bonn, Germany

[e]Department of Refractory Viral Diseases, National Institute of Global Health and Medicine, Japan Institute for Health Security,1-21-1 Toyama, Shinjuku-ku, Tokyo 162-8655, Japan

[f]Greehey Children's Cancer Research Institute, University of Texas Health Science Center at San Antonio, San Antonio, TX, USA, 78229

[g]Department of Microbiology, Institute of Biological Sciences, Federal University of Minas Gerais, Belo Horizonte, 31270-901, Minas Gerais, Brazil

[h]Center for Discovery and Innovation in Parasitic Diseases, Skaggs School of Pharmacy and Pharmaceutical Sciences, University of California, San Diego, 9500 Gilman Drive, La Jolla, CA 92093-0657, USA

[i]Department of Biotherapy, Cancer Center and State Key Laboratory of Biotherapy, West China Hospital, Sichuan University, Chengdu, Sichuan, 610041, China

[j]Howard Hughes Medical Institute, University of Texas Health San Antonio, San Antonio, Texas, USA, 78229

***Corresponding author**

Thanigaimalai Pillaiyar − Institute of Pharmacy, Pharmaceutical/Medicinal Chemistry and Tübingen Center for Academic Drug Discovery, Eberhard Karls University Tübingen, Auf der Morgenstelle 8, 72076 Tübingen, Germany. E-mail: thanigaimalai.pillaiyar@uni-tuebingen.de

**Contents**	**Page**
**Figure S1: A**. A $2mF_o$-DF_c electron density map contoured at 1.0 r.m.s.d.	S3
**Figure S2.** Antiviral activity of selected M^{pro} inhibitors.	S4
**Table S1**. Data Collection and refinement statistics for **20a** (FP-637)	S5
NMR chart for selected compounds	S6
HPLC chart for selected compounds	S34
HRMS Spectra for selected compounds	S75

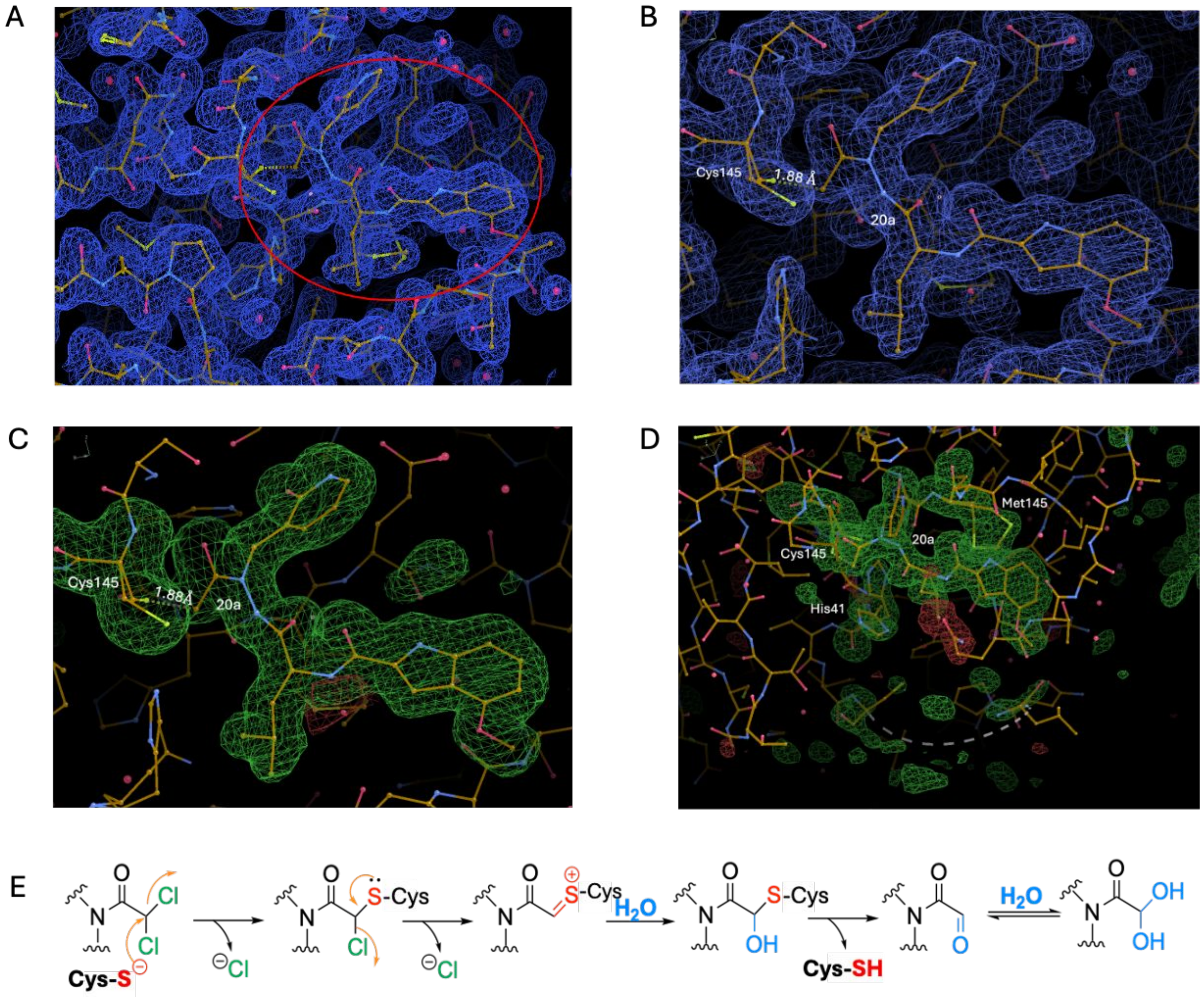

Figure S1: A. A $2mF_o$-DF_c electron density map contoured at 1.0 r.m.s.d. Electron density within the red circle is **20a**. **B.** Zoomed in given the covalent interaction between **20a** and Cys145, with a bond length of 1.88 Å. **C.** Polder map (contoured at 3.0 r.m.s.d) showing apparent positive electron density around compound **20a** covalently linked to Cys145, confirming covalent adduct formation. **D.** Polder map (contoured at 3.0 r.m.s.d) revealing alternative conformations of His41 and Met165, scattered and fragmented density suggests significant flexibility or partial disorder in the missing loop region (residues 45-49) shown with the dashed line. **E.** Proposed reaction mechanism illustrating the catalytic Cys145 initiates nucleophilic attack on the electrophilic carbon atom of the dihaloacetamide warhead of compound **20a**, followed by stepwise displacement of two chlorine atoms to form a thioether adduct. Under aqueous conditions, the thioether adduct undergoes hydrolysis, followed by thiol exchange to regenerate free Cys145 and release a carboxylic acid derivative. This reaction mechanism demonstrates both covalent inhibition and the potential for reversibility.

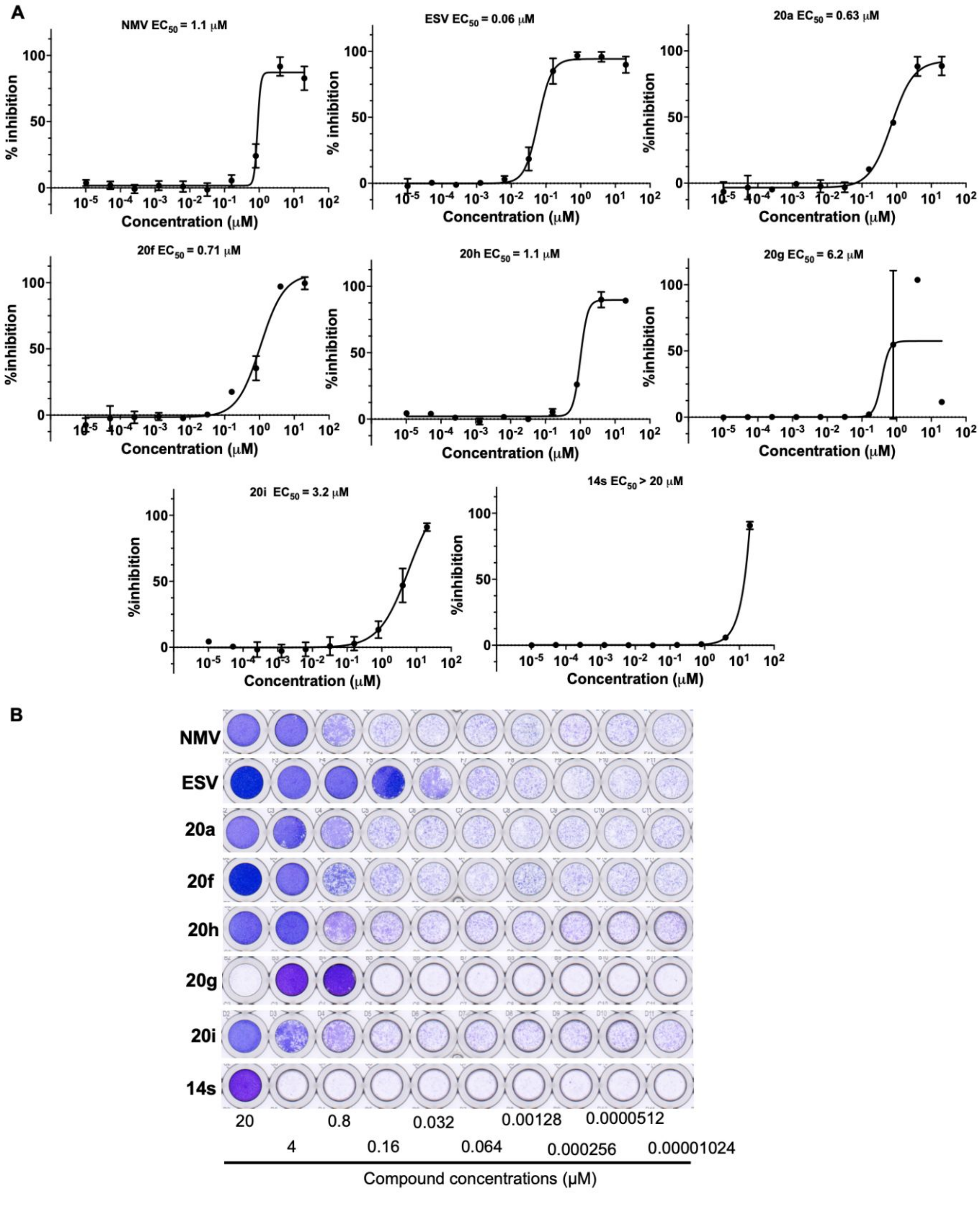

Figure S2. Antiviral activity of selected M^{pro} inhibitors. Panel A compares the CPE at different concentrations (20 µM to 5-fold dilutions over 10 points) for two controls (NMV and ESV) and the indicated M^{pro} inhibitor candidates (a). EC_{50} values are provided in the inset of each graph. Individual data points represent the mean values ± SD of 2-6 experimental replicates. Panel B demonstrates the visual representation of the CPE where the compounds are diluted similarly, left (highest) to right (lowest), and a violet crystal staining shows the cell death (no violet stain) or survival (violet stain) in Vero E6 SARS-CoV-2 infected condition (b).

Table S1. Data Collection and refinement statistics for **20a (FP-637)**.

Parameter	Value
**PDB Entry**	9MDQ
**Space group**	C2
**Unit cell parameters (Å)**	97.88, 83.07, 52.01; 90.0, 115.17, 90.0
**Wavelength (Å)**	1.54184
**Resolution range (Å)**	44.29 – 1.60 (1.63 – 1.60)
**Number of observations**	299424 (5831)
**Number of unique reflections**	48638 (1934)
**Completeness (%)**	98.5 (100.0)
**Mean I/σ(I)**	14.4 (0.8)
**Multiplicity**	6.2 (7.4)
**Rmerge (all)**	0.060 (0.937)
**Rpim (all)**	0.024 (0.594)
**CC1/2**	0.999 (0.621)
**Wilson B-factor (Å^2)**	21.04
**R-work**	0.1657 (0.3140)
**R-free**	0.2032 (0.3564)
**RMS (bonds)**	0.007
**RMSD (angles)**	0.9
**Number of protein atoms**	2321
**Number of ligand atoms**	34
**Number of water molecules**	184
**Average B-factor – protein (Å^2)**	33
**Average B-factor – ligand (Å^2)**	28
**Average B-factor – water (Å^2)**	39
**Ramachandran favored (%)**	98.29
**Ramachandran allowed (%)**	1.71
**Ramachandran outliers (%)**	0
**Clashscore**	2.81

NMR data for selected compounds

5e

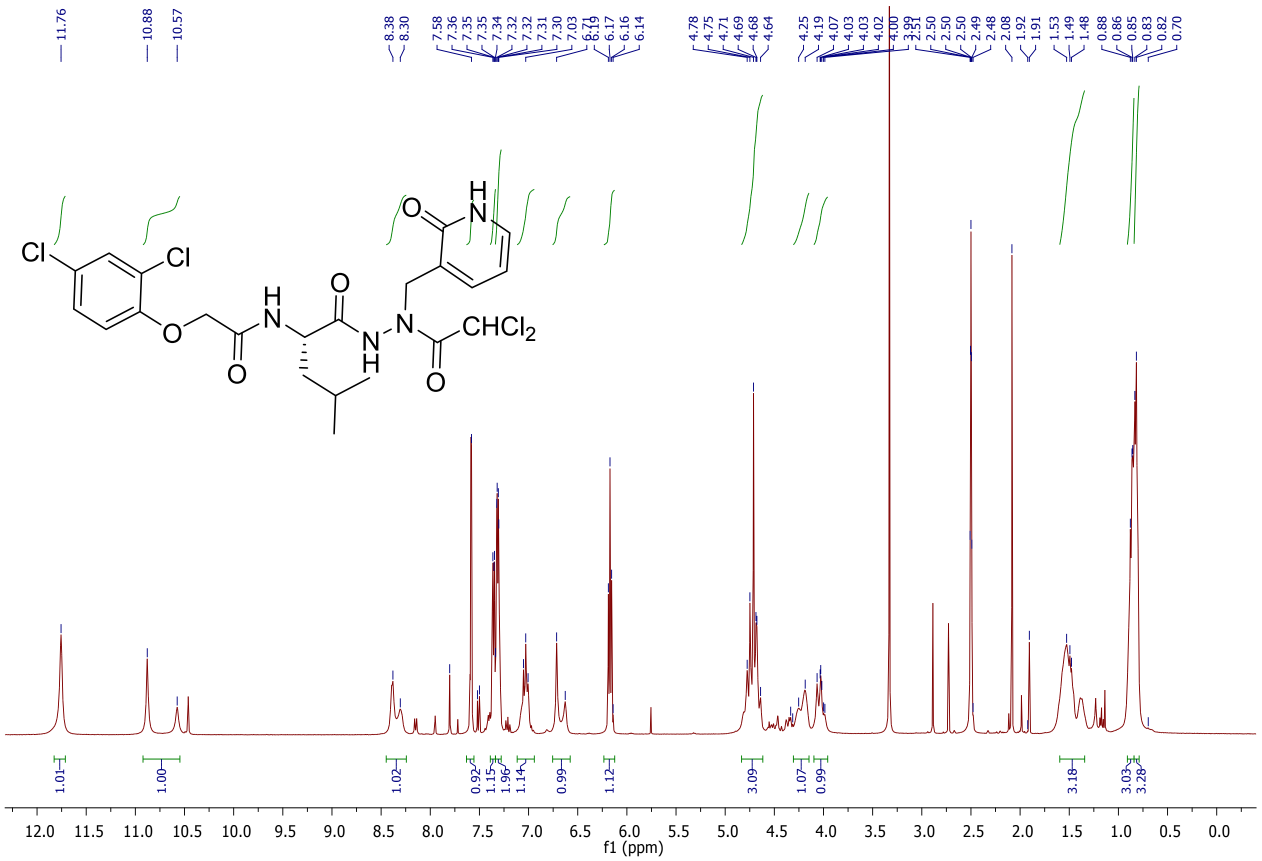

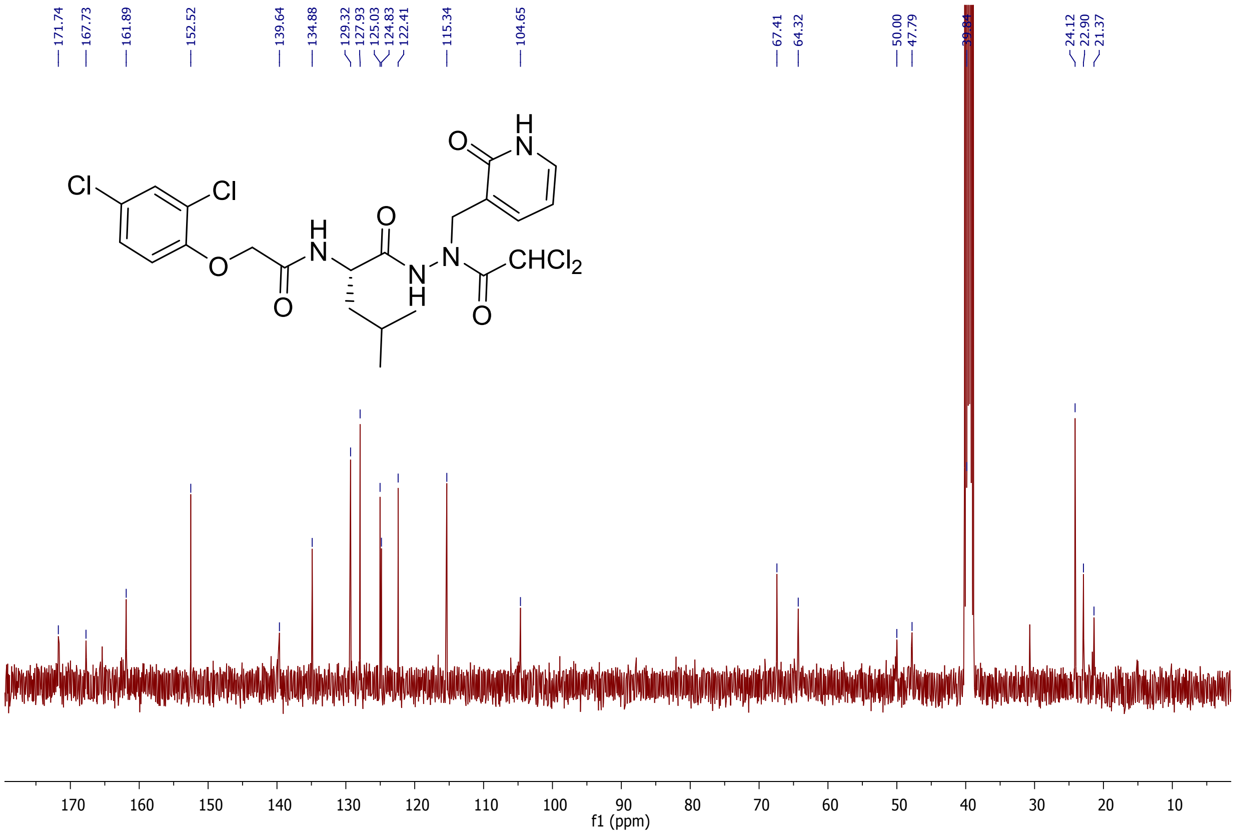

5f

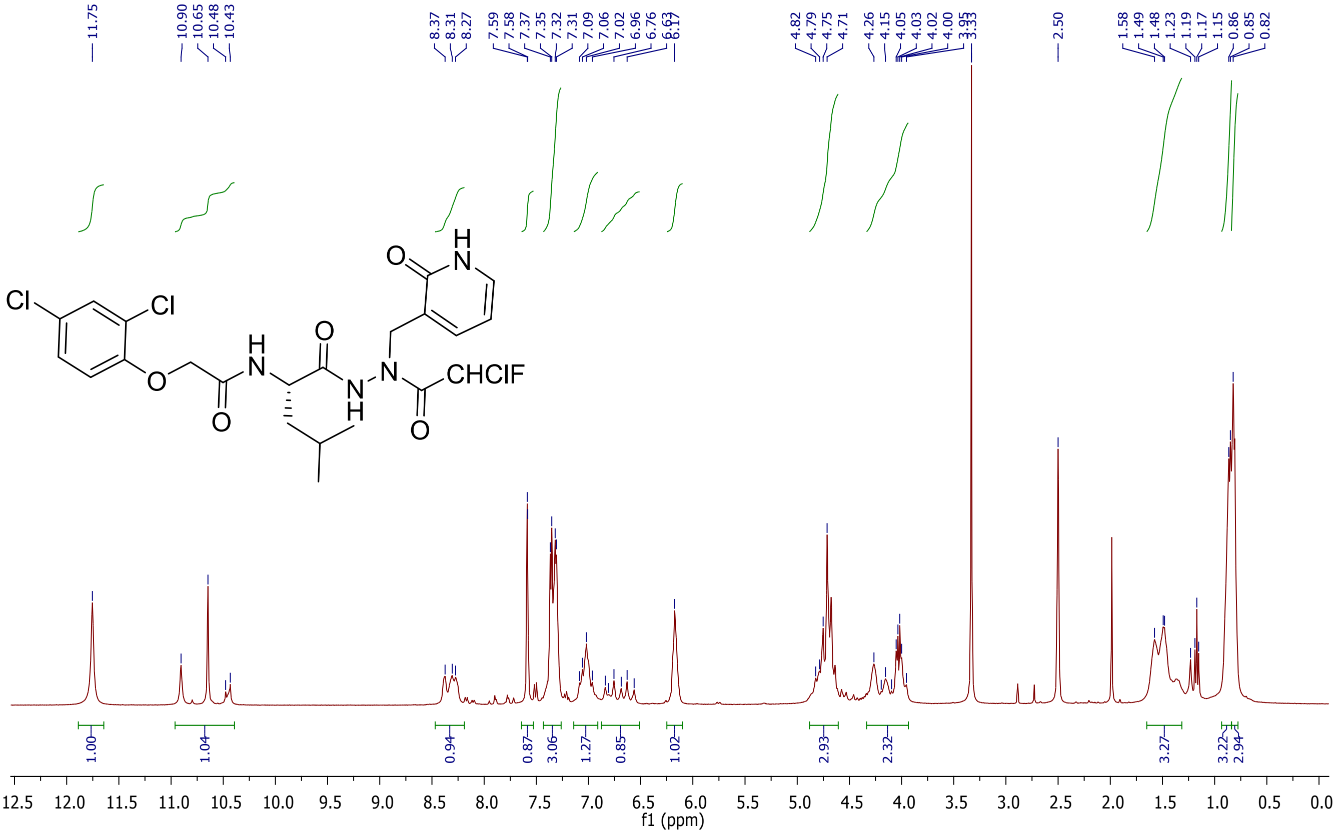

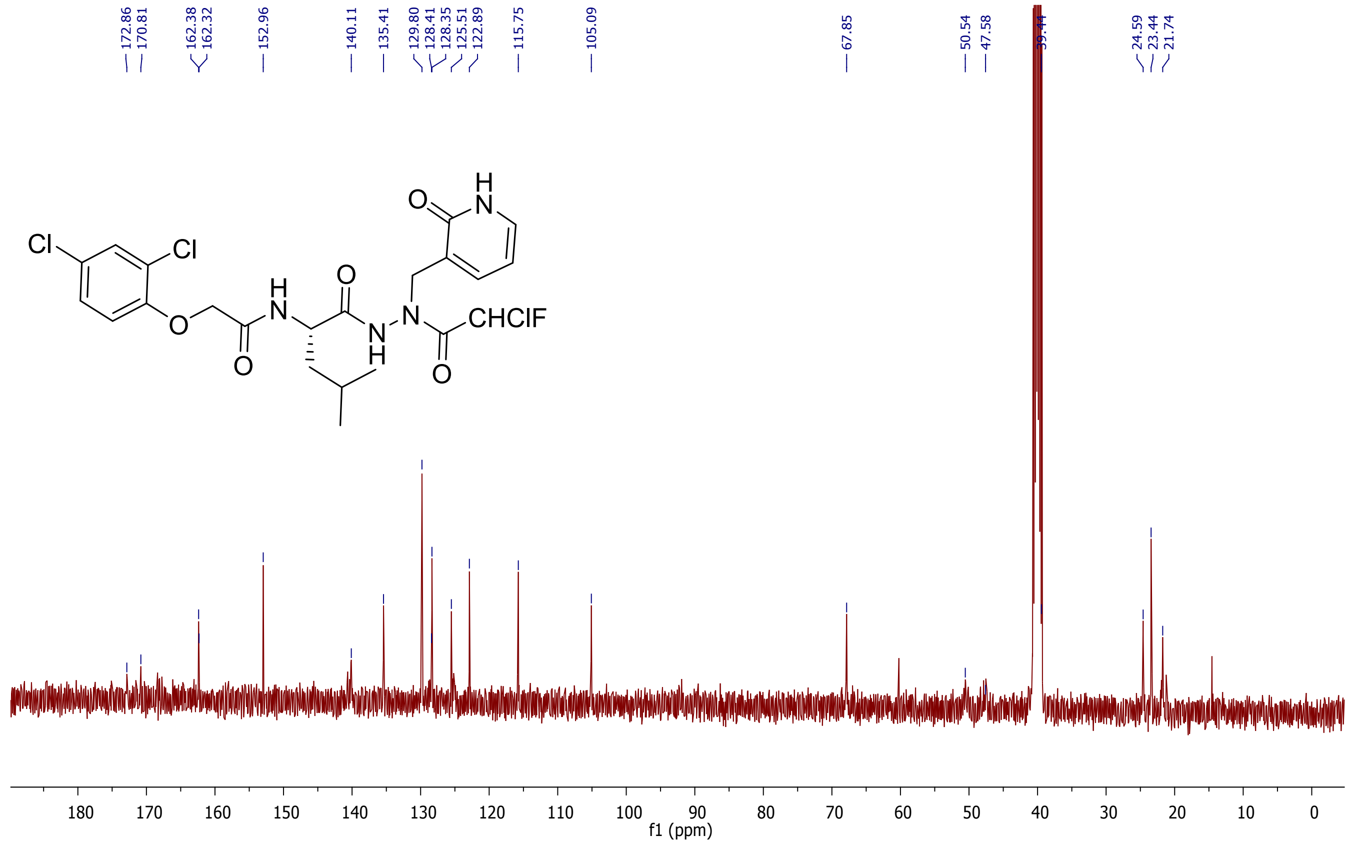

5h

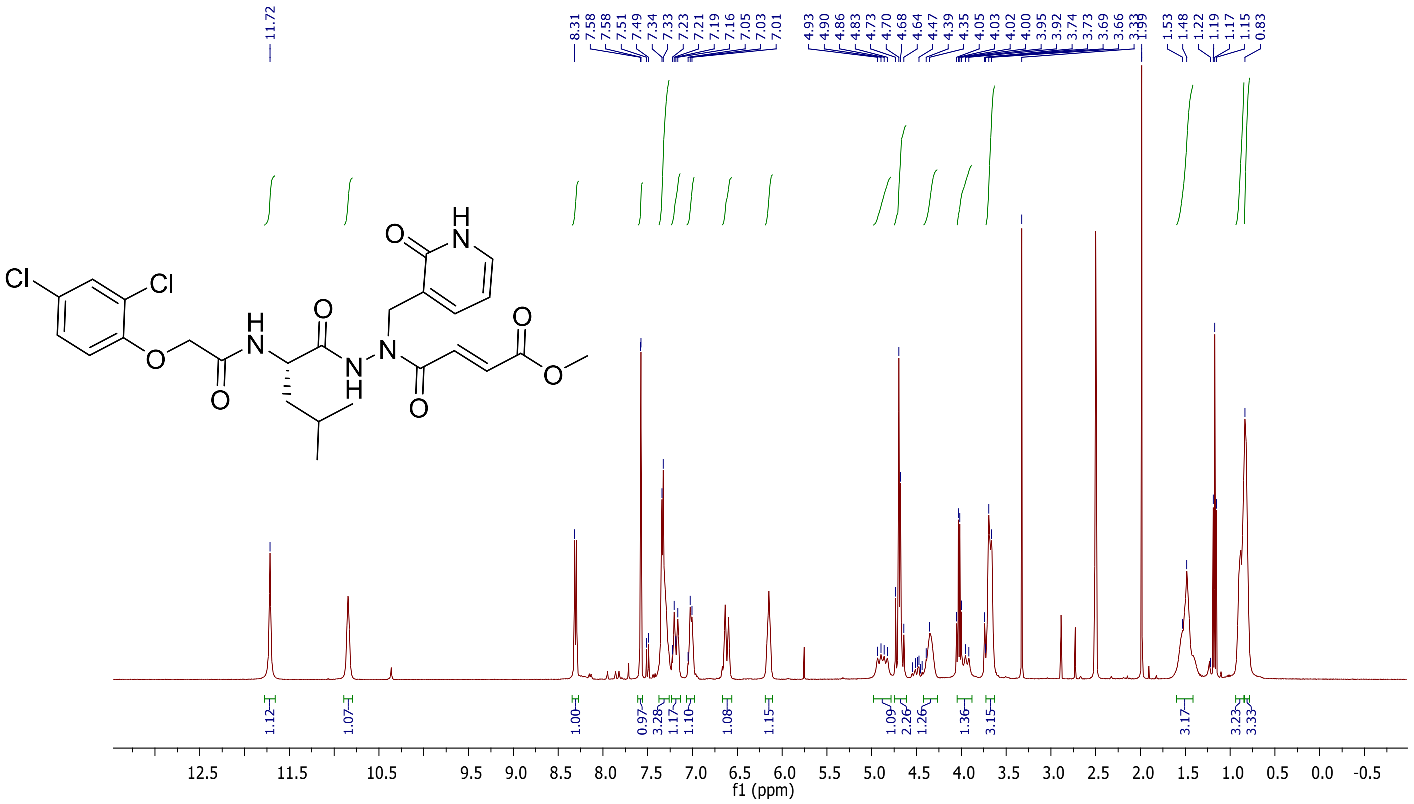
11.72
8.31 7.58 7.58 7.51 7.49 7.34 7.33 7.23 7.21 7.19 7.16 7.05 7.03 7.01
4.93 4.90 4.86 4.83 4.73 4.70 4.68 4.64 4.47 4.39 4.35 4.05 4.03 4.02 4.00 3.95 3.92 3.74 3.73 3.69 3.66 3.33 1.99
1.53 1.48 1.22 1.19 1.17 1.15 0.83
Cl
Cl
O
H
N
O
O
N
H
N
O
O
O
O
1.12 1.07 1.00 0.97 3.28 1.17 1.10 1.08 1.15 1.09 2.26 1.26 1.36 3.15 3.17 3.23 3.33
12.5 11.5 10.5 9.5 9.0 8.5 8.0 7.5 7.0 6.5 6.0 5.5 5.0 4.5 4.0 3.5 3.0 2.5 2.0 1.5 1.0 0.5 0.0 -0.5
f1 (ppm)

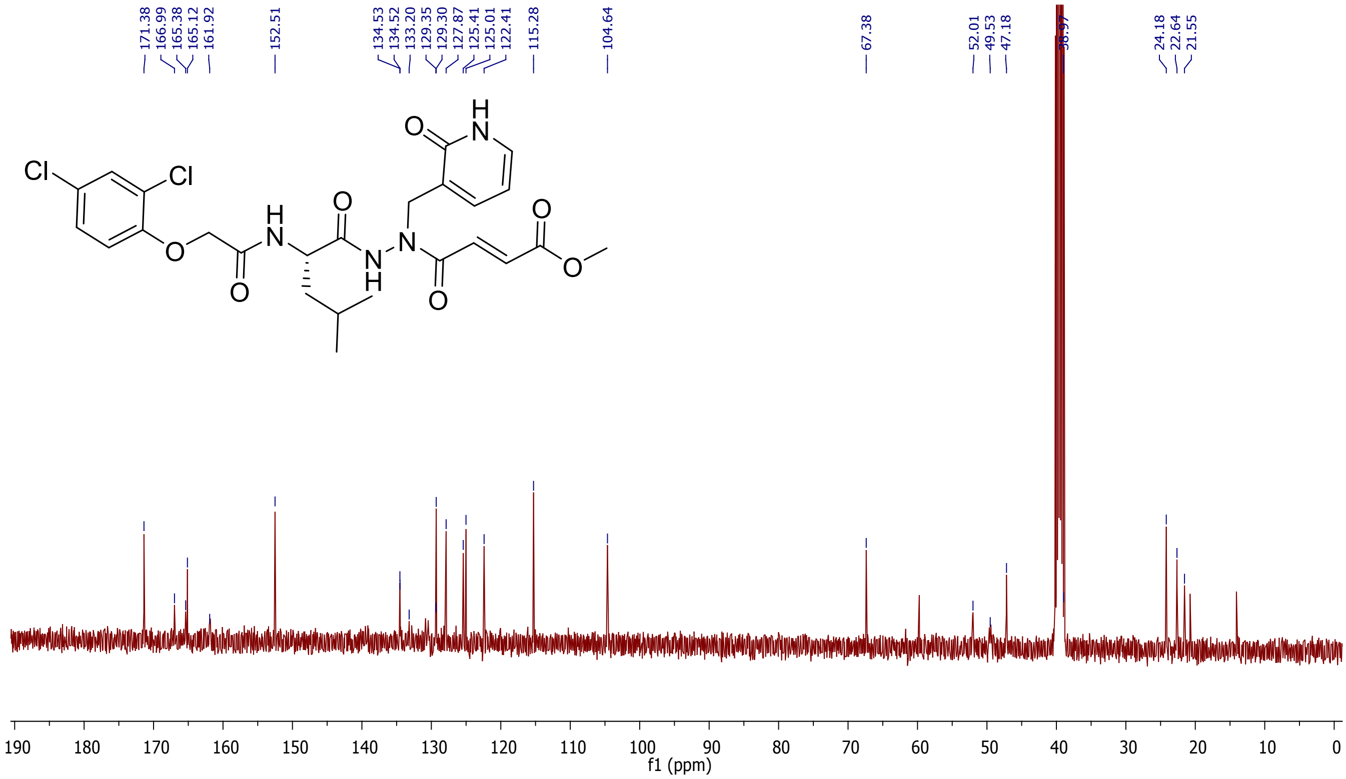
171.38 166.99 165.38 165.12 161.92
152.51
134.53 134.52 133.20 129.35 129.30 127.87 125.41 125.01 122.41
115.28
104.64
67.38
52.01 49.53 47.18
24.18 22.64 21.55
Cl
Cl
O
H
N
O
O
N
H
N
O
O
O
O
190 180 170 160 150 140 130 120 110 100 90 80 70 60 50 40 30 20 10 0
f1 (ppm)

6a

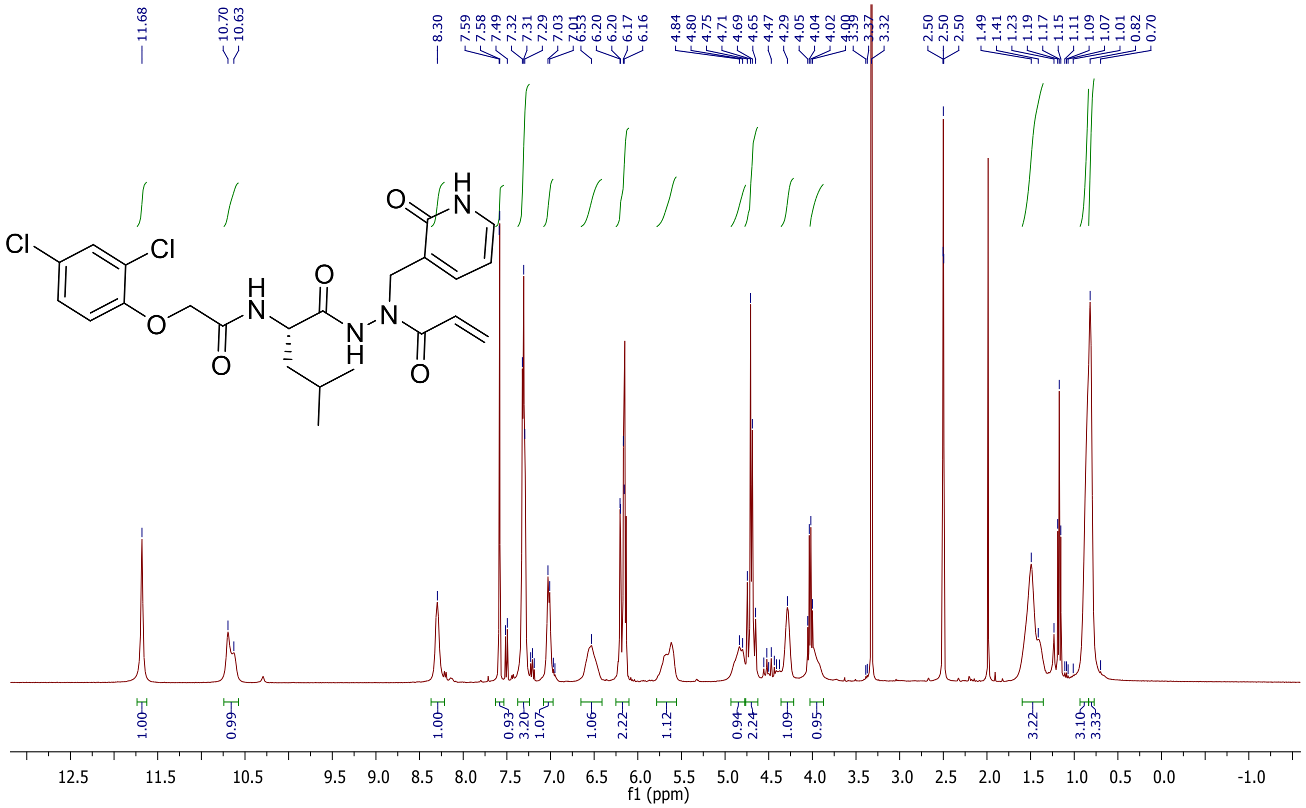

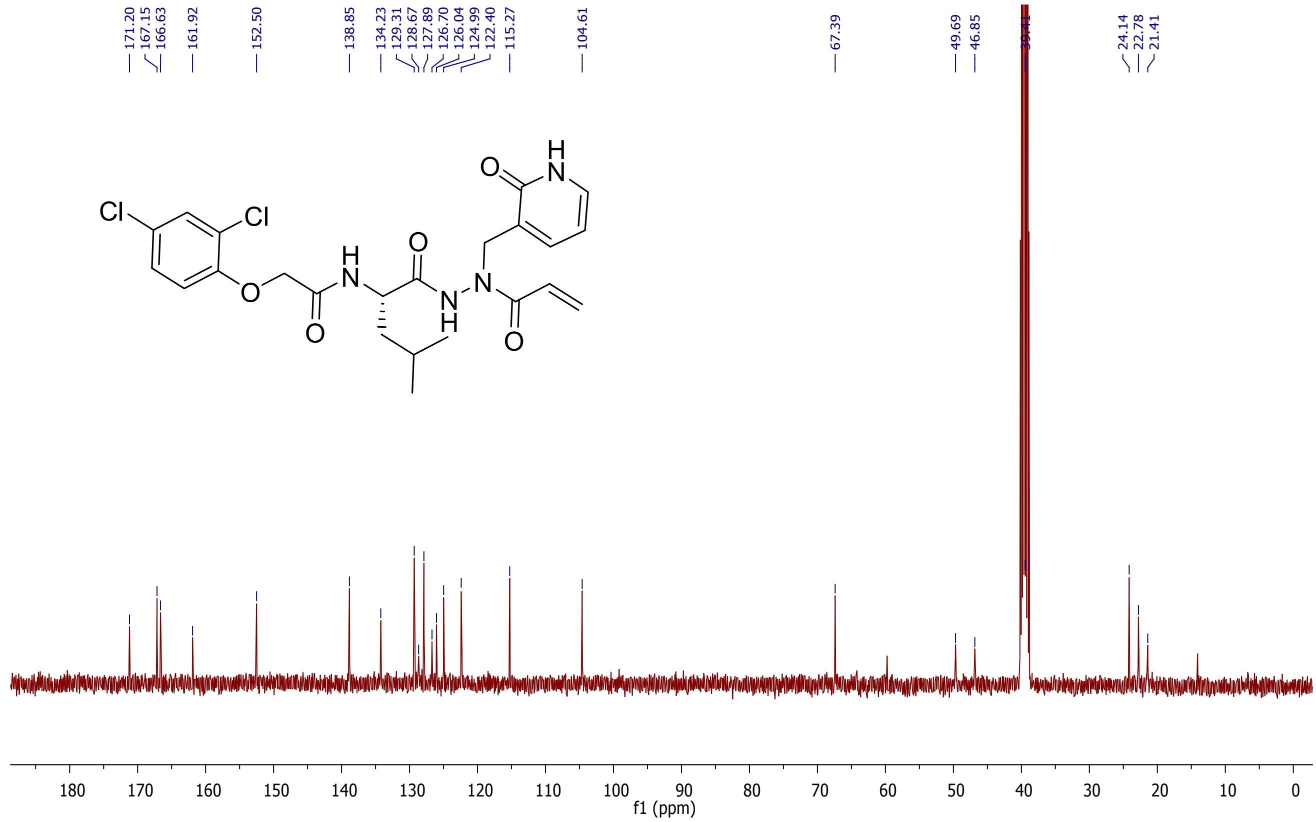

6c

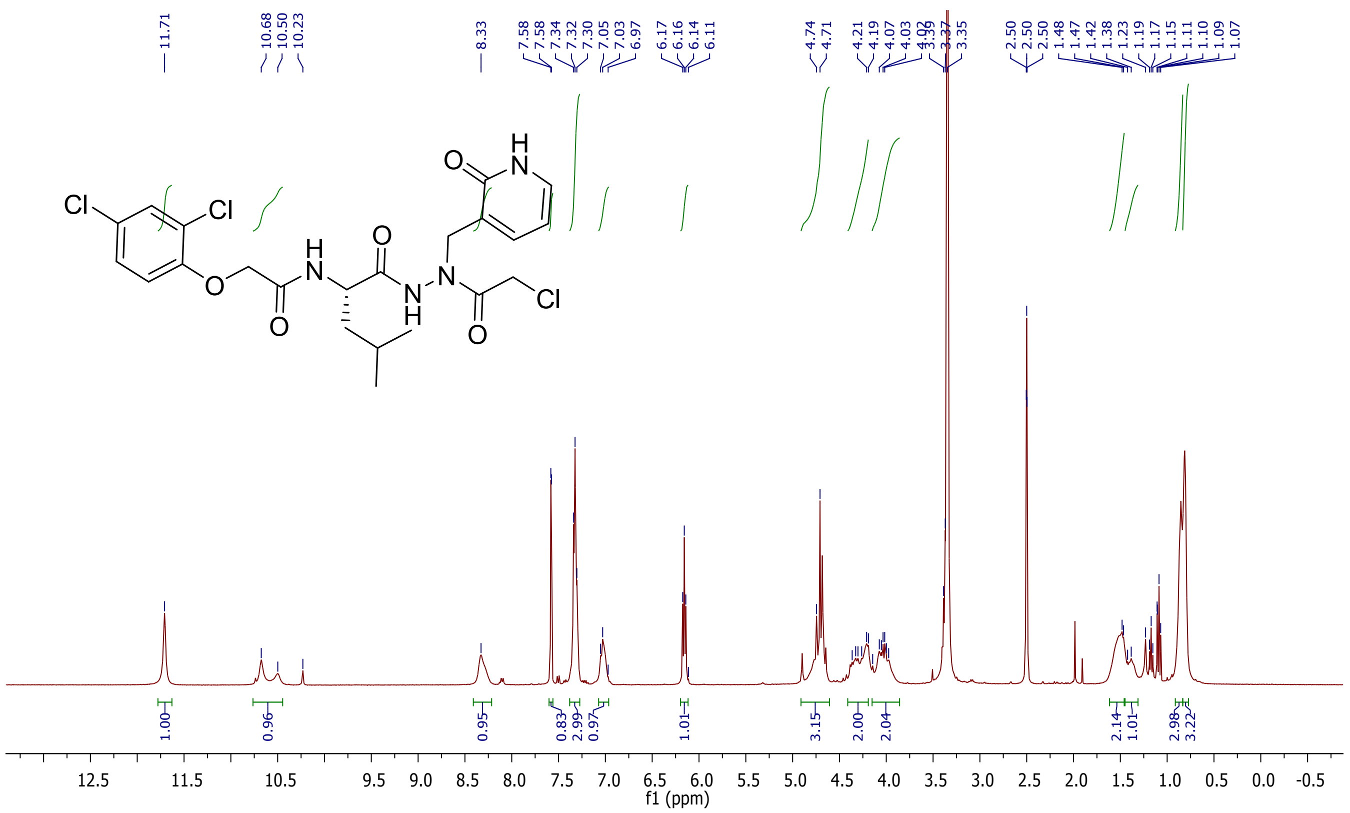

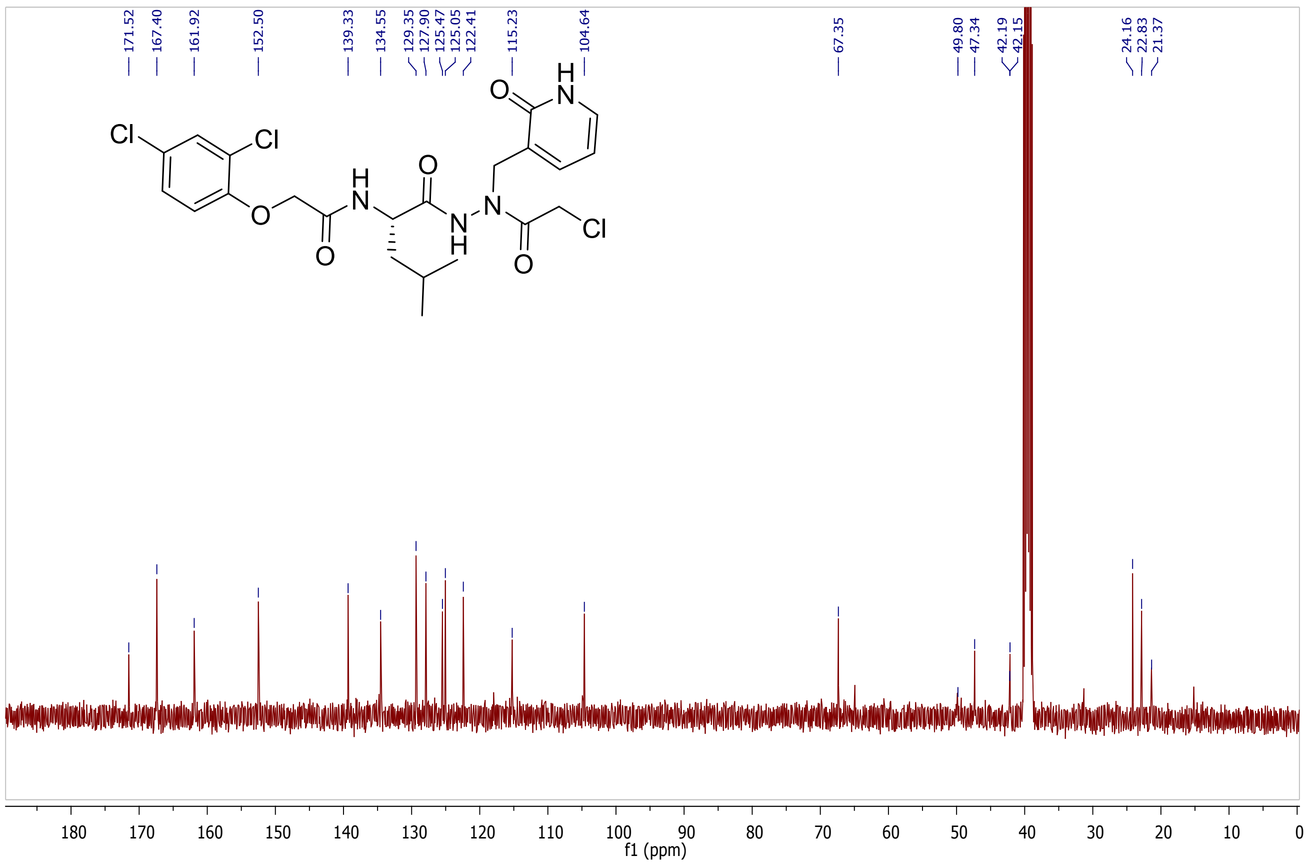

8a

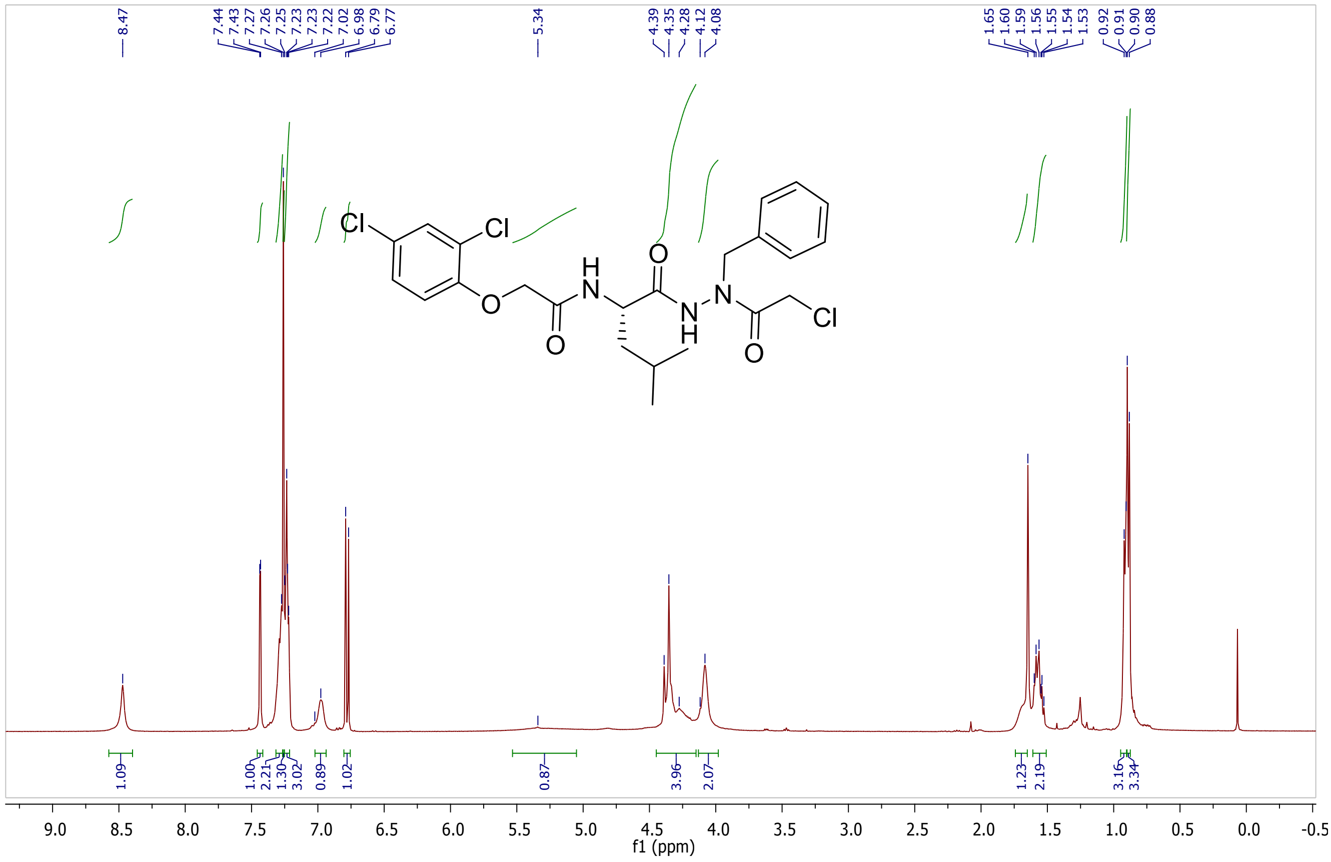

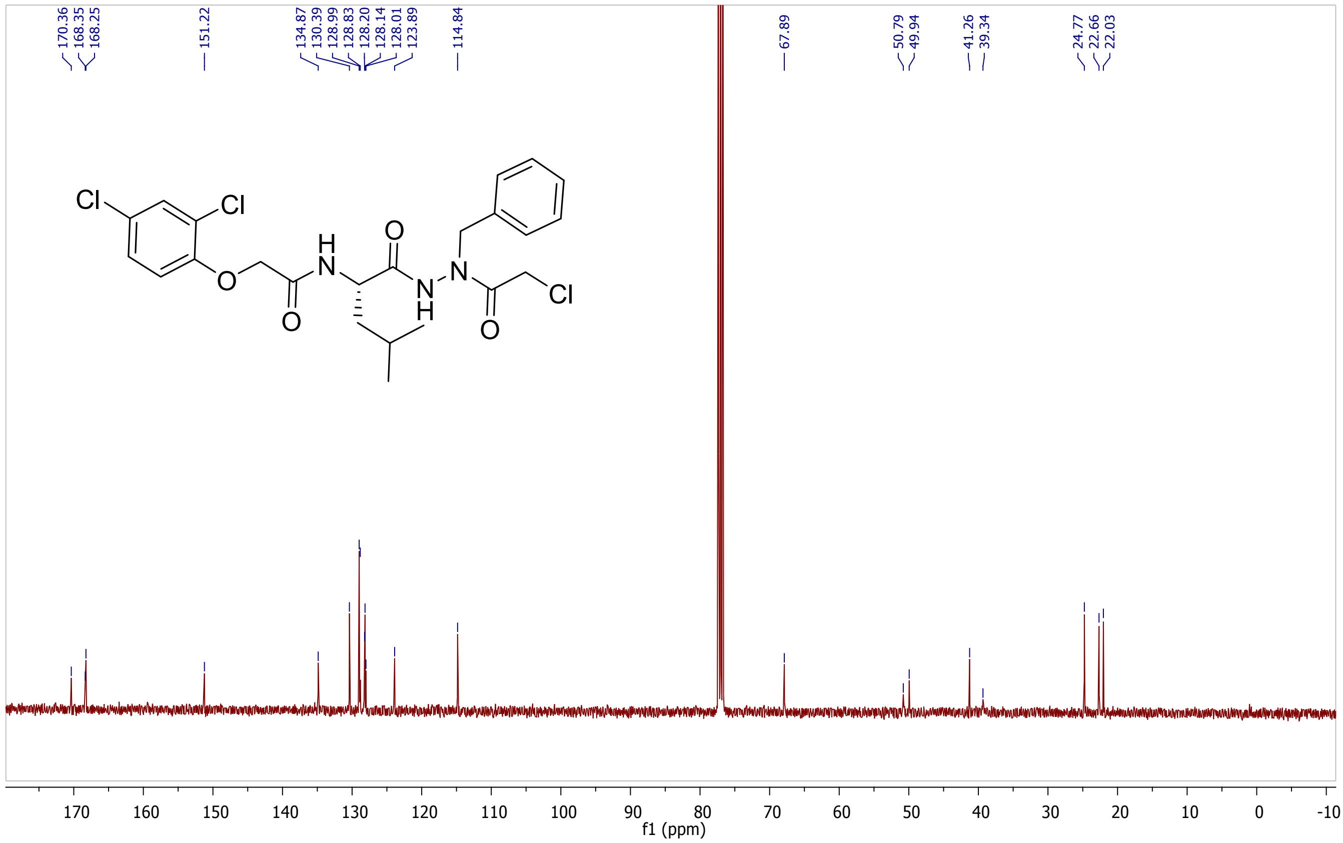

8b

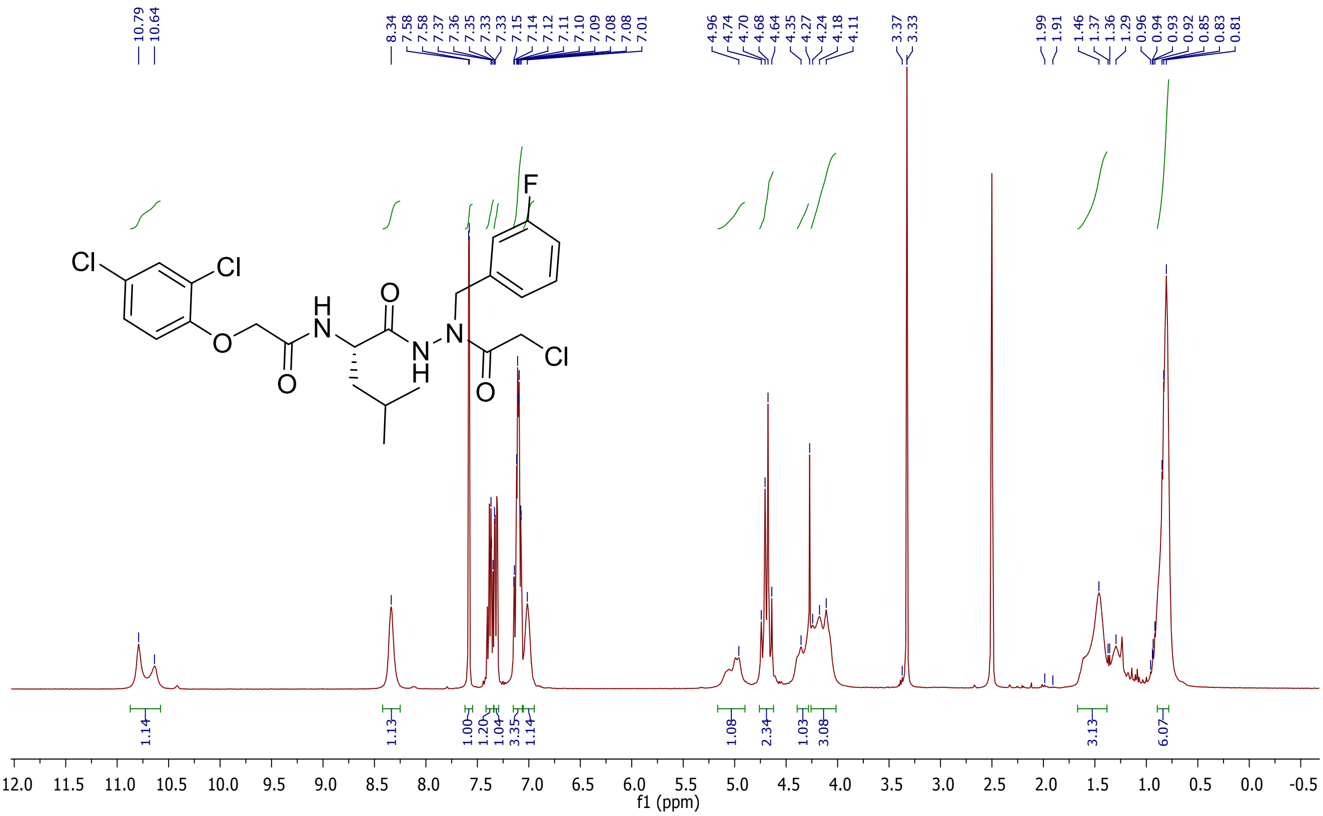
10.79
10.64
8.34
7.58
7.58
7.37
7.36
7.35
7.33
7.33
7.15
7.14
7.12
7.11
7.10
7.09
7.08
7.08
7.01
4.96
4.74
4.70
4.68
4.64
4.35
4.27
4.24
4.18
4.11
3.37
3.33
1.99
1.91
1.46
1.37
1.36
1.29
0.96
0.94
0.93
0.92
0.85
0.83
0.81
F
Cl
Cl
O
O
H
N
O
N
N
H
O
Cl
1.14
1.13
1.00
1.20
1.04
3.35
1.14
1.08
2.34
1.03
3.08
3.13
6.07
f1 (ppm)

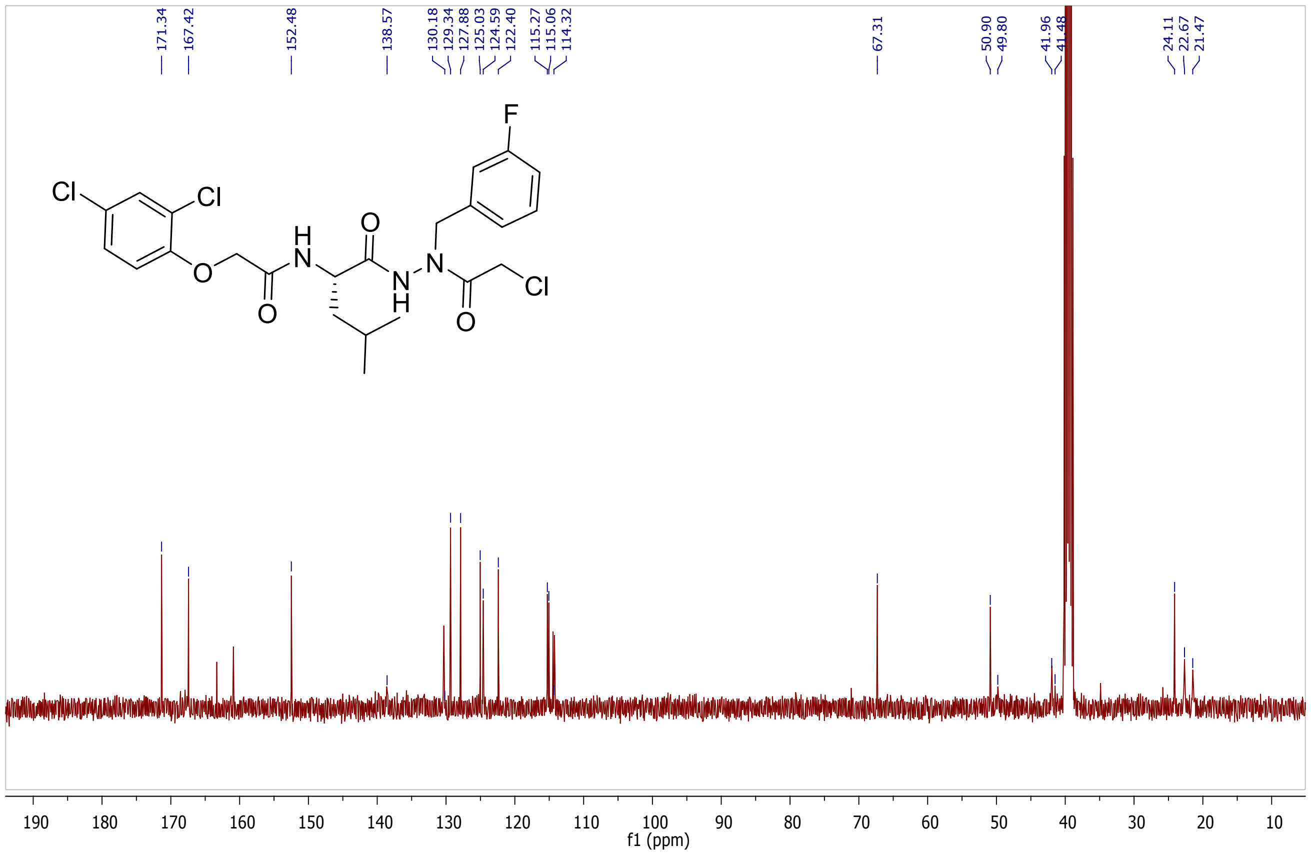
171.34
167.42
152.48
138.57
130.18
129.34
127.88
125.03
124.59
122.40
115.27
115.06
114.32
67.31
50.90
49.80
41.96
41.48
24.11
22.67
21.47
F
Cl
Cl
O
O
H
N
O
N
N
H
O
Cl
f1 (ppm)

8c

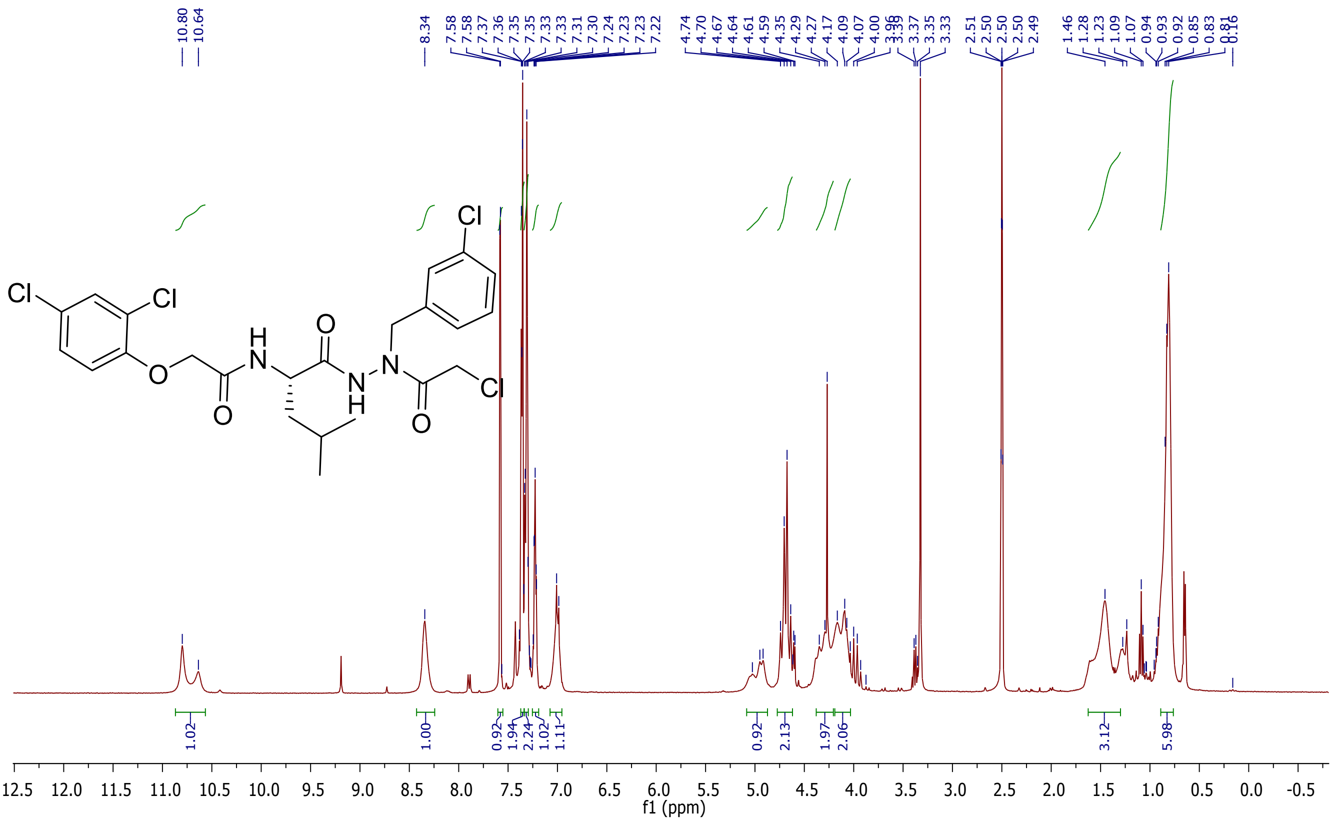
10.80
10.64
8.34
7.58
7.58
7.37
7.36
7.35
7.35
7.33
7.33
7.31
7.30
7.24
7.23
7.23
7.22
4.74
4.70
4.67
4.64
4.61
4.59
4.35
4.29
4.27
4.17
4.09
4.07
4.00
3.37
3.35
3.33
2.51
2.50
2.50
2.50
2.49
1.46
1.28
1.23
1.09
1.07
0.94
0.93
0.92
0.85
0.83
0.16
Cl
Cl
Cl
Cl
O
O
H
N
O
N
H
N
O
1.02
1.00
0.92
1.94
2.24
1.02
1.11
0.92
2.13
1.97
2.06
3.12
5.98
12.5 12.0 11.5 11.0 10.5 10.0 9.5 9.0 8.5 8.0 7.5 7.0 6.5 6.0 5.5 5.0 4.5 4.0 3.5 3.0 2.5 2.0 1.5 1.0 0.5 0.0 -0.5
f1 (ppm)

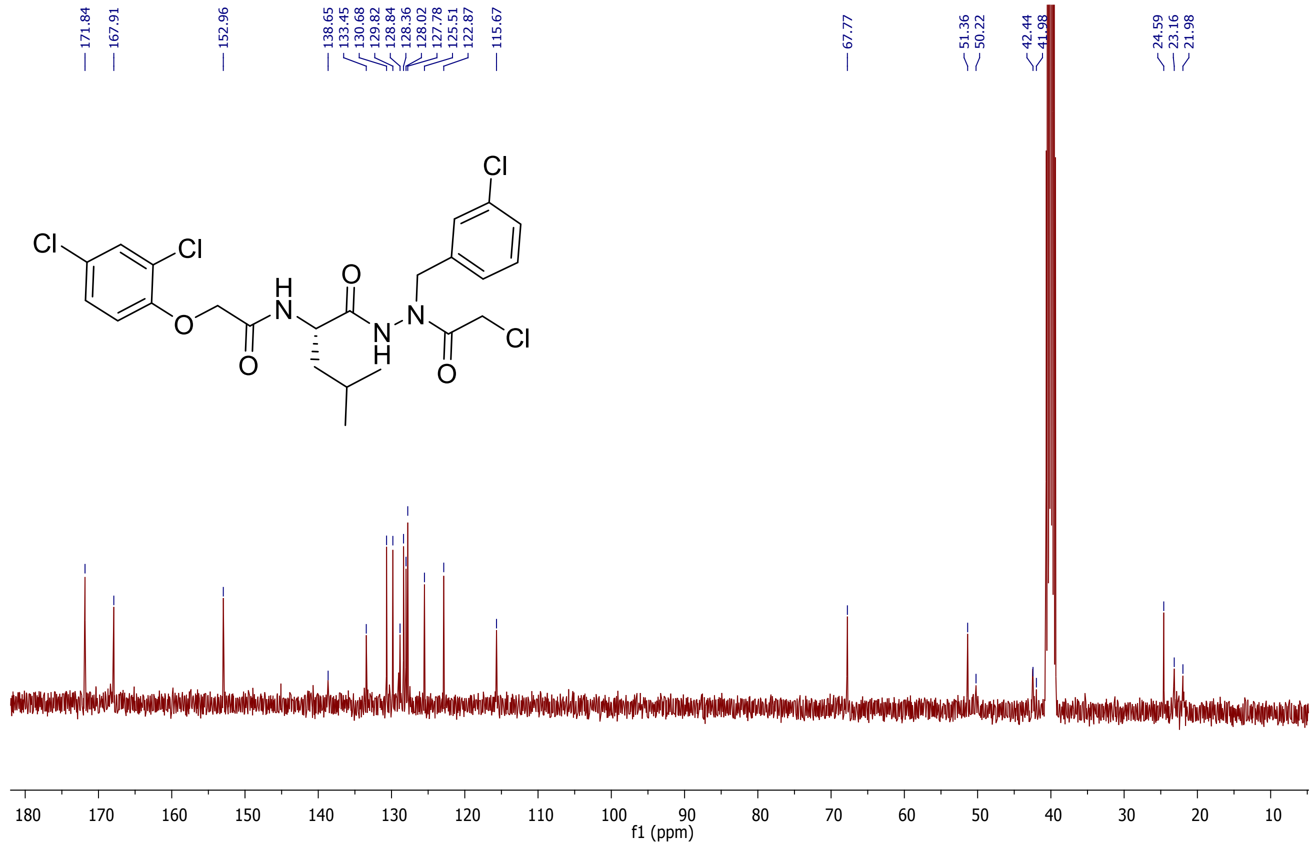
171.84
167.91
152.96
138.65
133.45
130.68
129.82
128.84
128.36
128.02
127.78
125.51
122.87
115.67
67.77
51.36
50.22
42.44
41.98
24.59
23.16
21.98
Cl
Cl
Cl
Cl
O
O
H
N
O
N
H
N
O
180 170 160 150 140 130 120 110 100 90 80 70 60 50 40 30 20 10
f1 (ppm)

8f

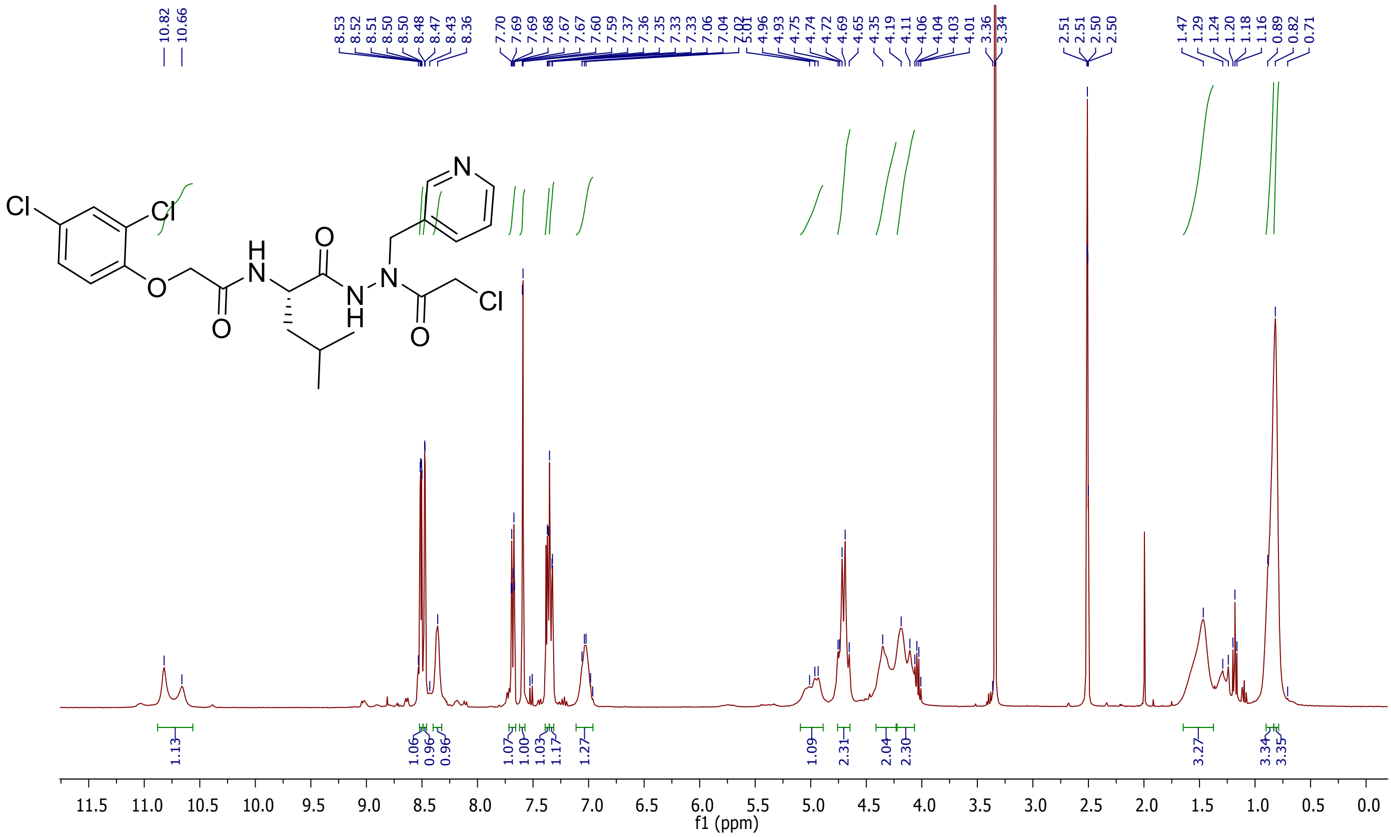
10.82
10.66
8.53
8.52
8.51
8.50
8.50
8.48
8.47
8.43
8.36
7.70
7.69
7.69
7.68
7.67
7.60
7.59
7.37
7.36
7.35
7.33
7.33
7.06
7.04
7.02
5.01
4.96
4.93
4.75
4.74
4.72
4.69
4.65
4.35
4.19
4.11
4.06
4.04
4.03
4.01
3.36
3.34
2.51
2.51
2.50
2.50
1.47
1.29
1.24
1.20
1.18
1.16
0.89
0.82
0.71
Cl
Cl
O
H
N
O
N
H
N
N
Cl
O
O
1.13
1.06
0.96
0.96
1.07
1.00
1.03
1.17
1.27
1.09
2.31
2.04
2.30
3.27
3.34
3.35
f1 (ppm)

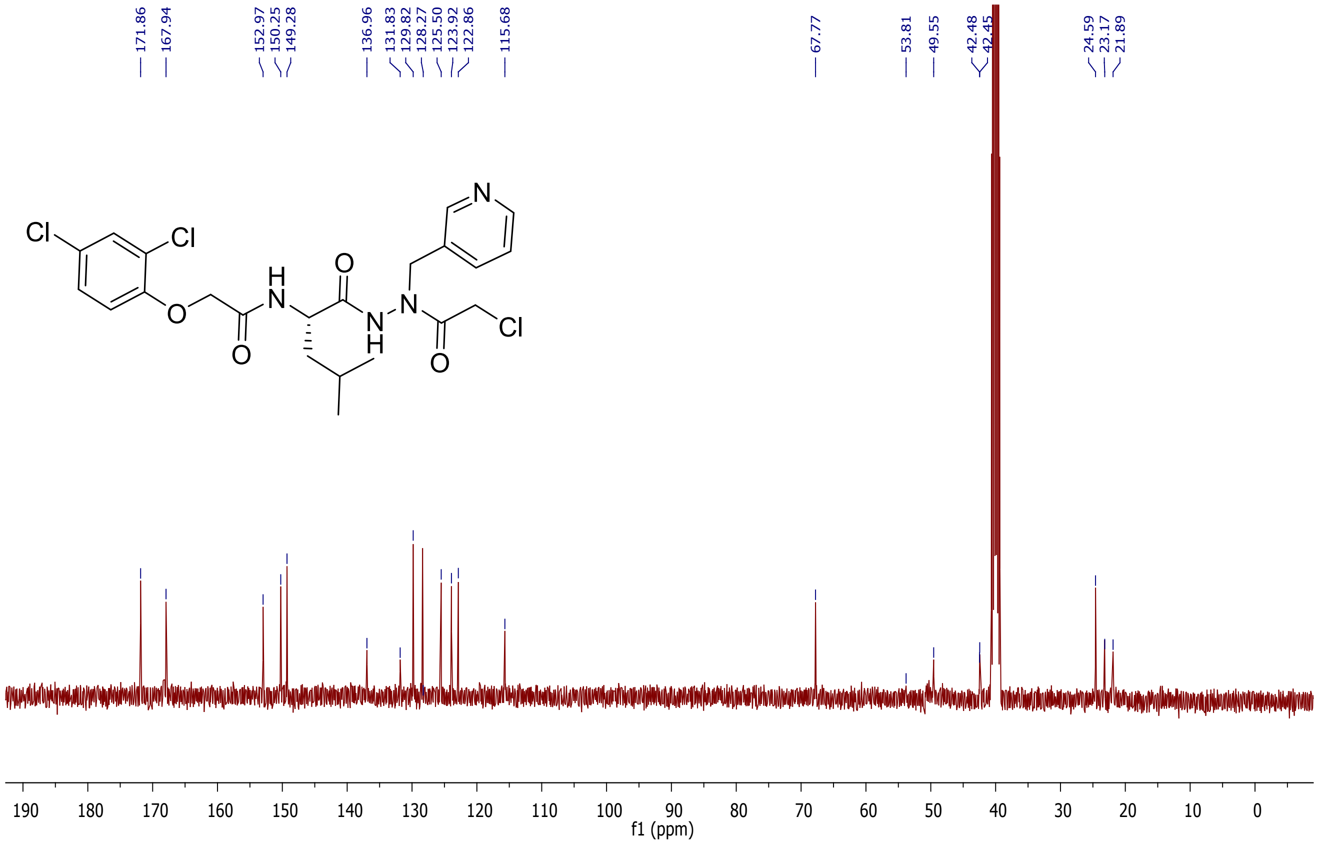
171.86
167.94
152.97
150.25
149.28
136.96
131.83
129.82
128.27
125.50
123.92
122.86
115.68
67.77
53.81
49.55
42.48
42.45
24.59
23.17
21.89
Cl
Cl
O
H
N
O
N
H
N
N
Cl
O
O
f1 (ppm)

8g

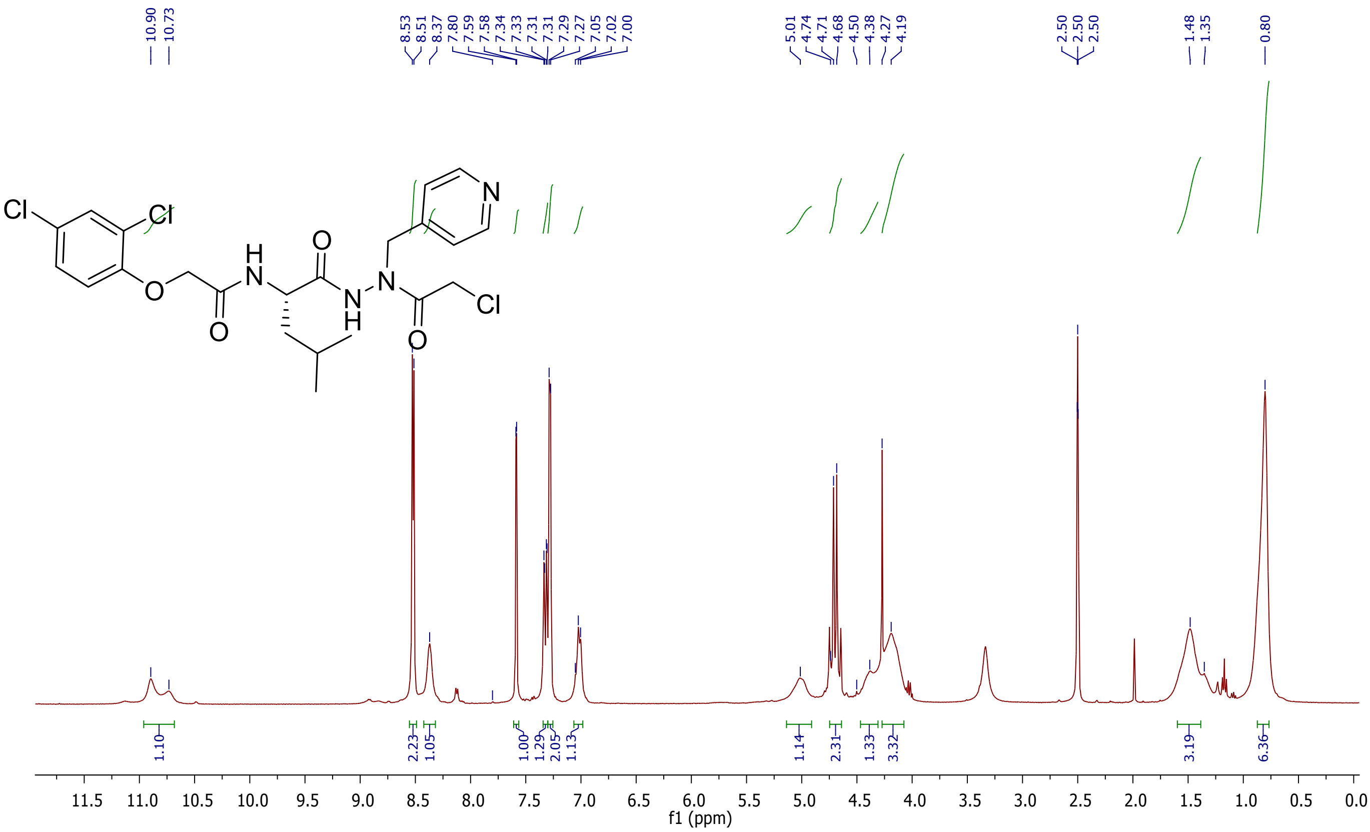
10.90
10.73
8.53
8.51
8.37
7.80
7.59
7.58
7.34
7.33
7.31
7.31
7.29
7.27
7.05
7.02
7.00
5.01
4.74
4.71
4.68
4.50
4.38
4.27
4.19
2.50
2.50
2.50
1.48
1.35
0.80
Cl
Cl
O
N
H
O
N
H
N
N
O
Cl
O
1.10
2.23
1.05
1.00
1.29
2.05
1.13
1.14
2.31
1.33
3.32
3.19
6.36
f1 (ppm)

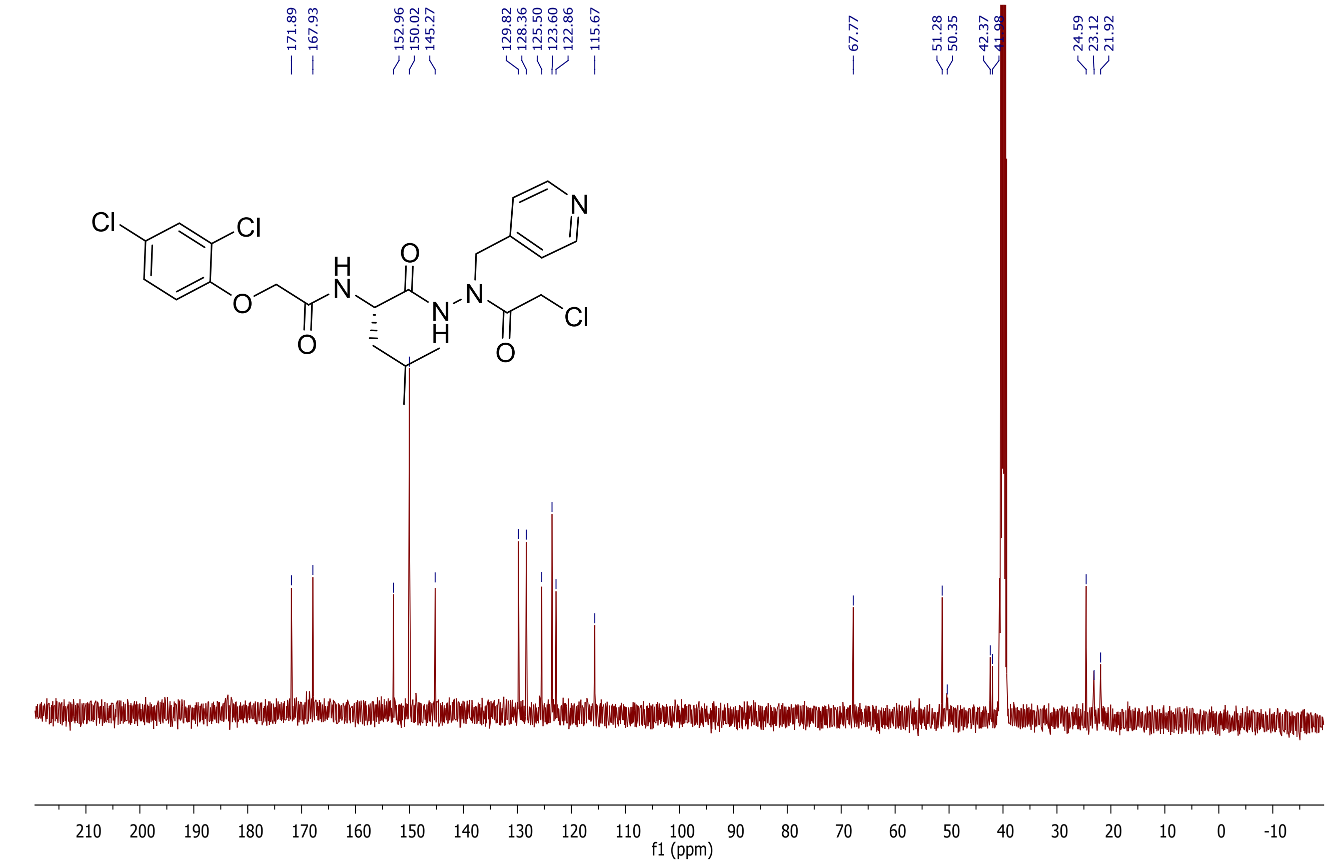
171.89
167.93
152.96
150.02
145.27
129.82
128.36
125.50
123.60
122.86
115.67
67.77
51.28
50.35
42.37
41.98
24.59
23.12
21.92
Cl
Cl
O
N
H
O
N
H
N
N
O
Cl
O
f1 (ppm)

8i

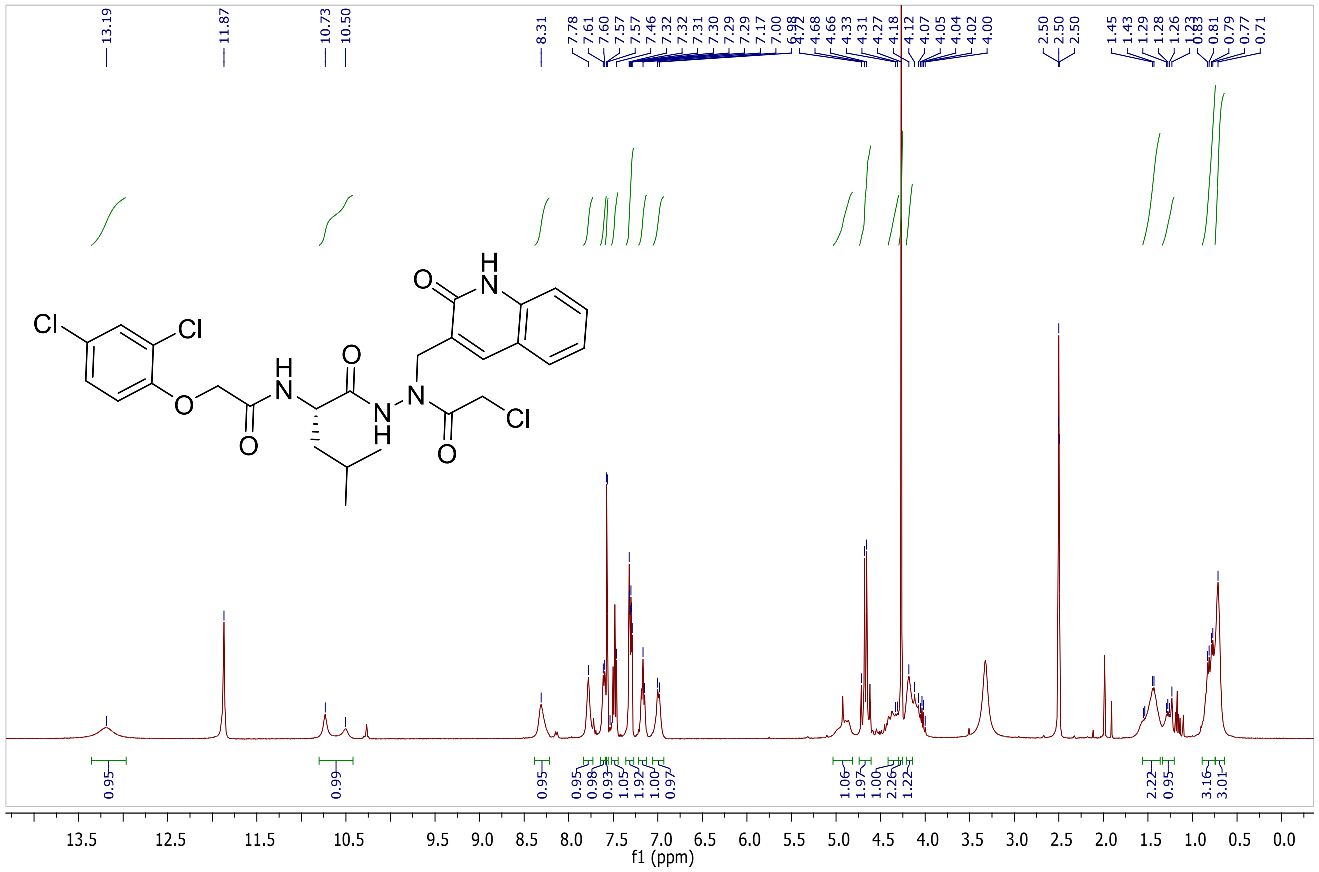

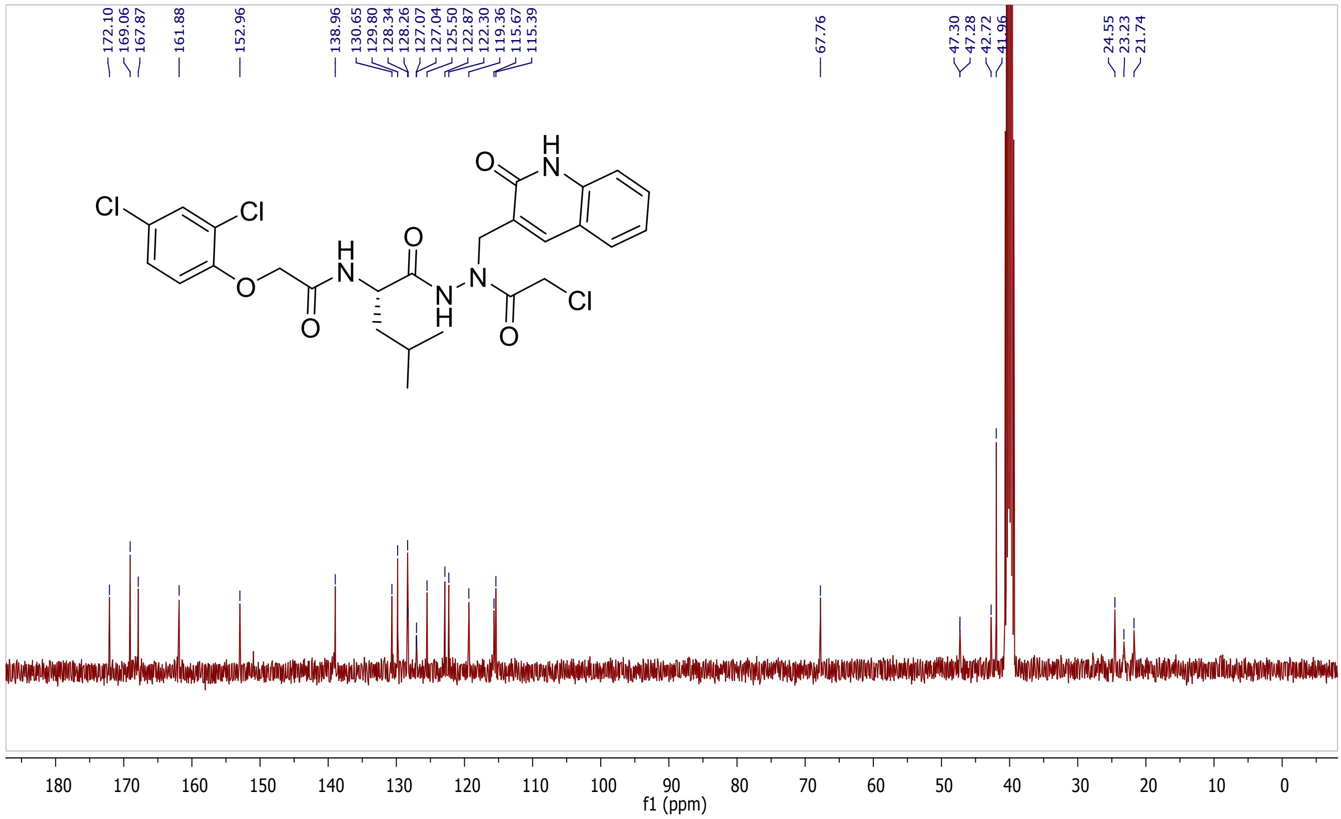

8j

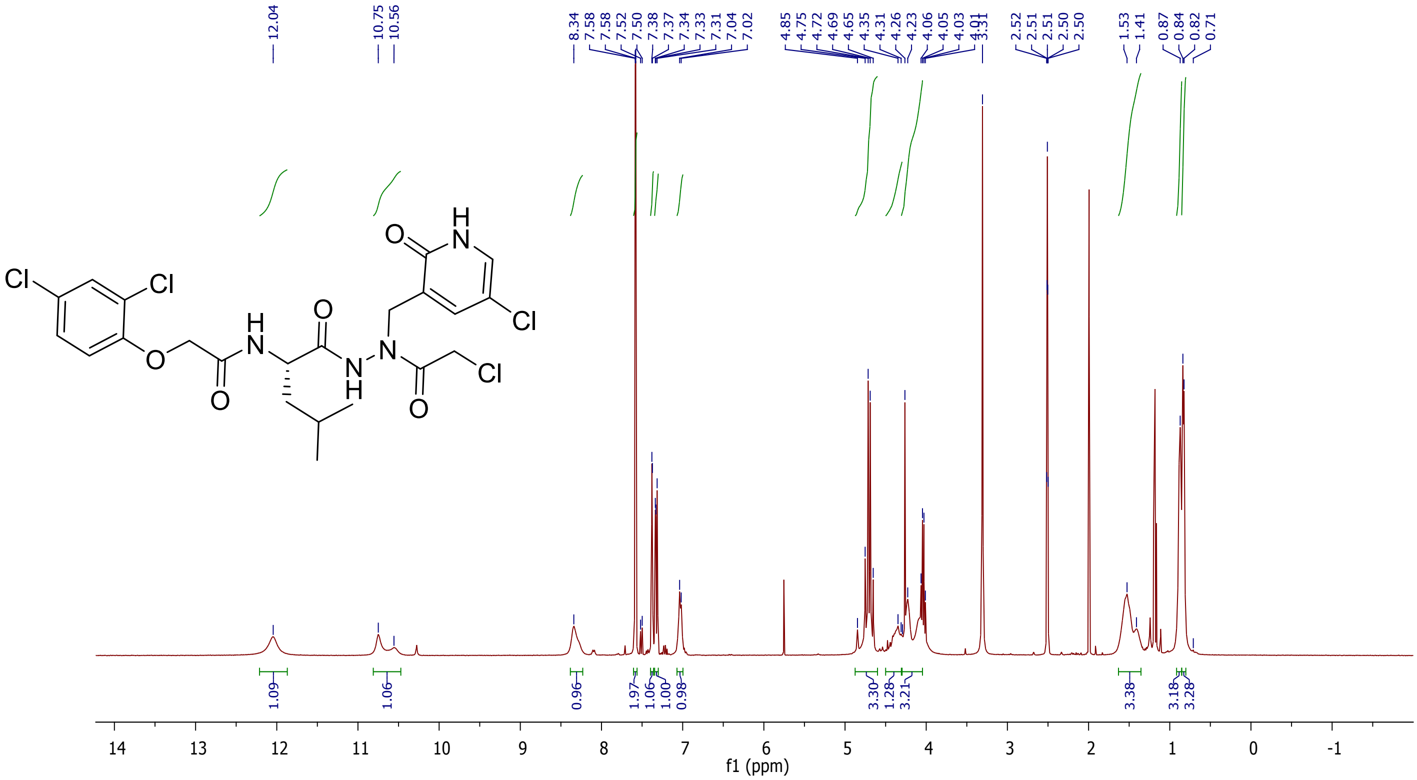

12.04
10.75
10.56
8.34
7.58
7.58
7.52
7.50
7.38
7.37
7.34
7.33
7.31
7.04
7.02
4.85
4.75
4.72
4.69
4.65
4.35
4.31
4.26
4.23
4.06
4.05
4.03
4.01
3.31
2.52
2.51
2.51
2.50
2.50
1.53
1.41
0.87
0.84
0.82
0.71
1.09
1.06
0.96
1.97
1.06
1.00
0.98
3.30
1.28
3.21
3.38
3.18
3.28
f1 (ppm)

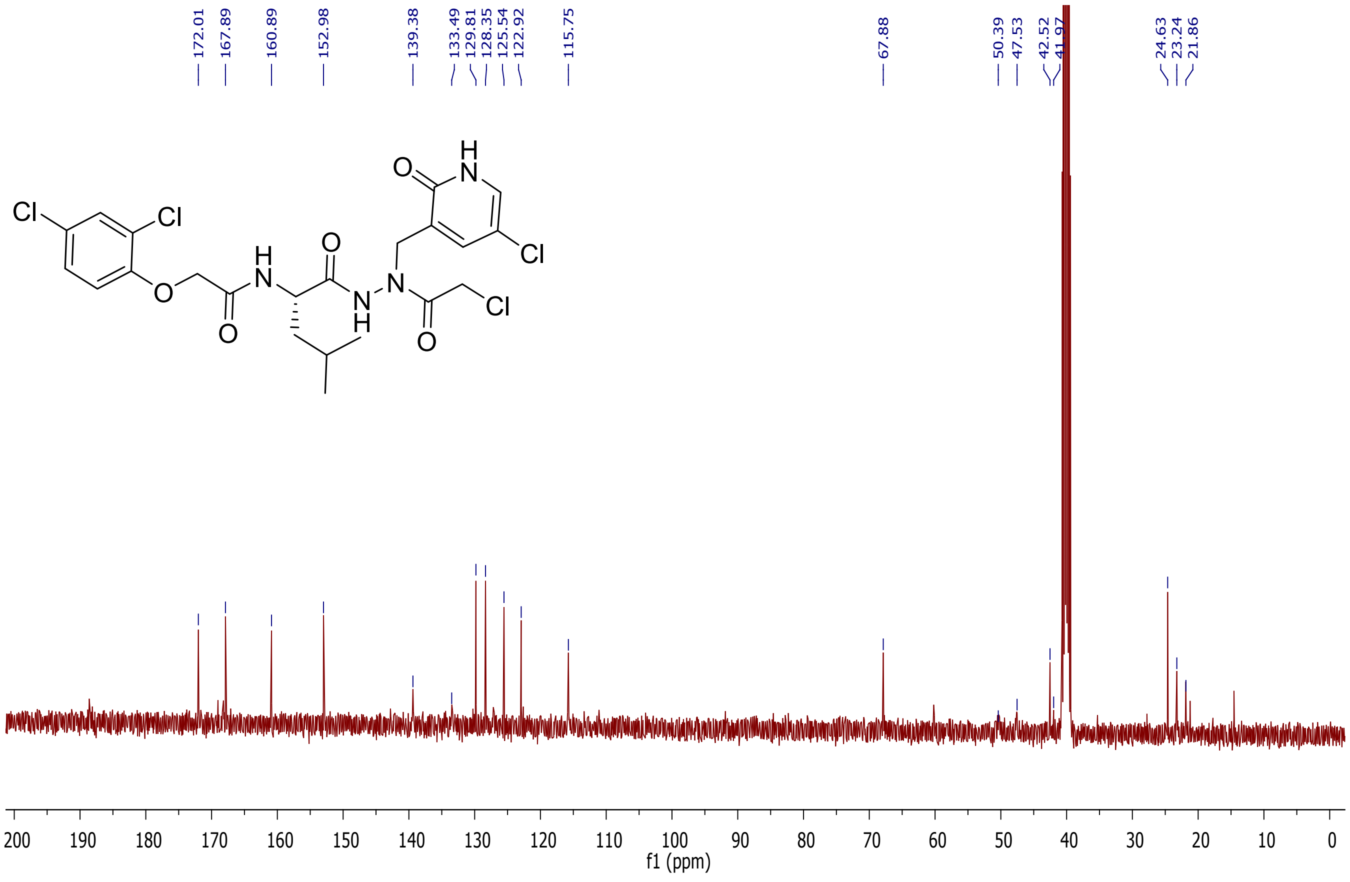

172.01
167.89
160.89
152.98
139.38
133.49
129.81
128.35
125.54
122.92
115.75
67.88
50.39
47.53
42.52
41.97
24.63
23.24
21.86
f1 (ppm)

8k

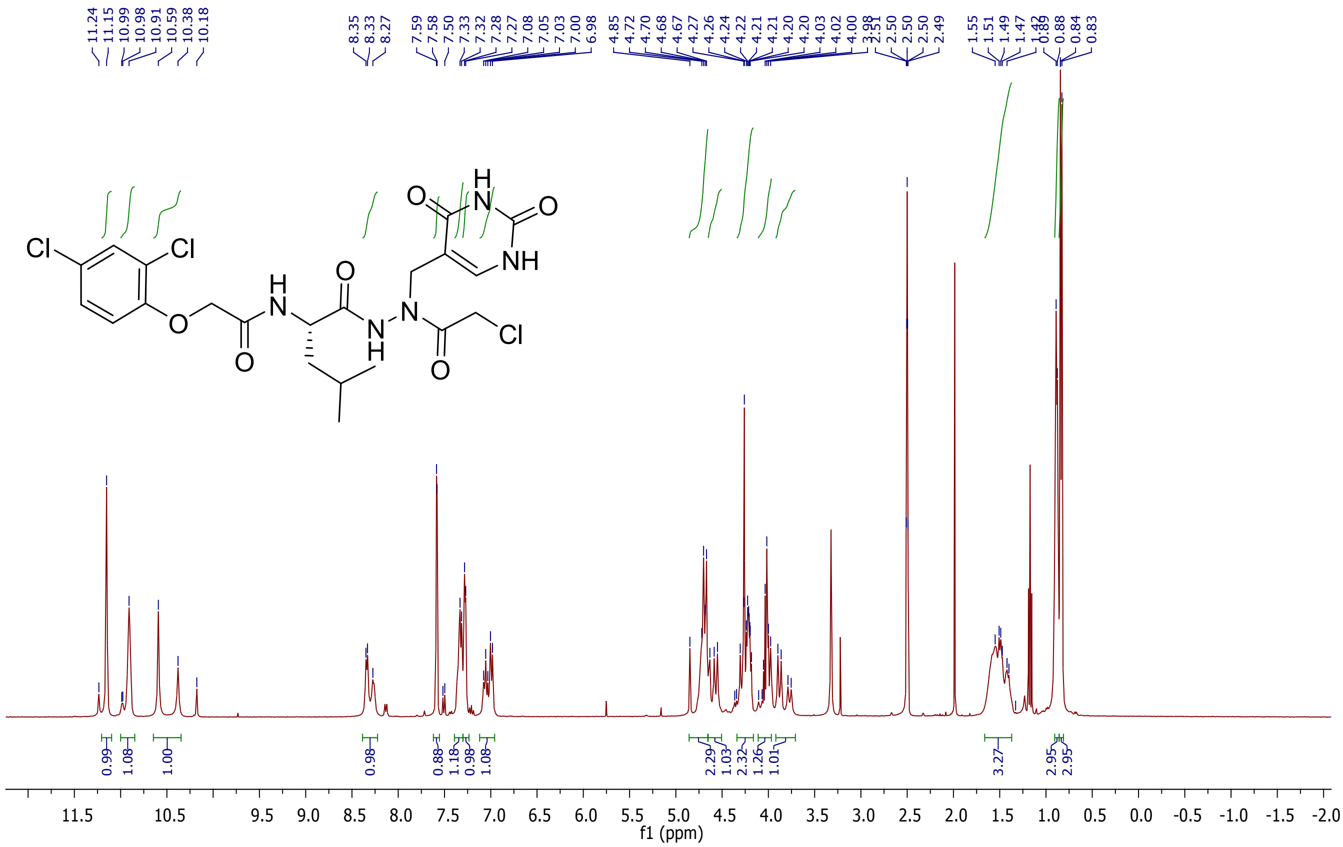
11.24
11.15
10.99
10.98
10.91
10.59
10.38
10.18
8.35
8.33
8.27
7.59
7.58
7.50
7.33
7.32
7.28
7.27
7.08
7.05
7.03
7.00
6.98
4.85
4.72
4.70
4.68
4.67
4.27
4.26
4.24
4.22
4.21
4.21
4.20
4.20
4.03
4.02
4.00
3.98
2.51
2.50
2.50
2.50
2.49
1.55
1.51
1.49
1.47
1.42
0.89
0.88
0.84
0.83
Cl
Cl
O
O
H
N
O
N
H
N
O
Cl
O
H
N
O
NH
0.99
1.08
1.00
0.98
0.88
1.18
0.98
1.08
2.29
1.03
2.32
1.26
1.01
3.27
2.95
2.95
f1 (ppm)

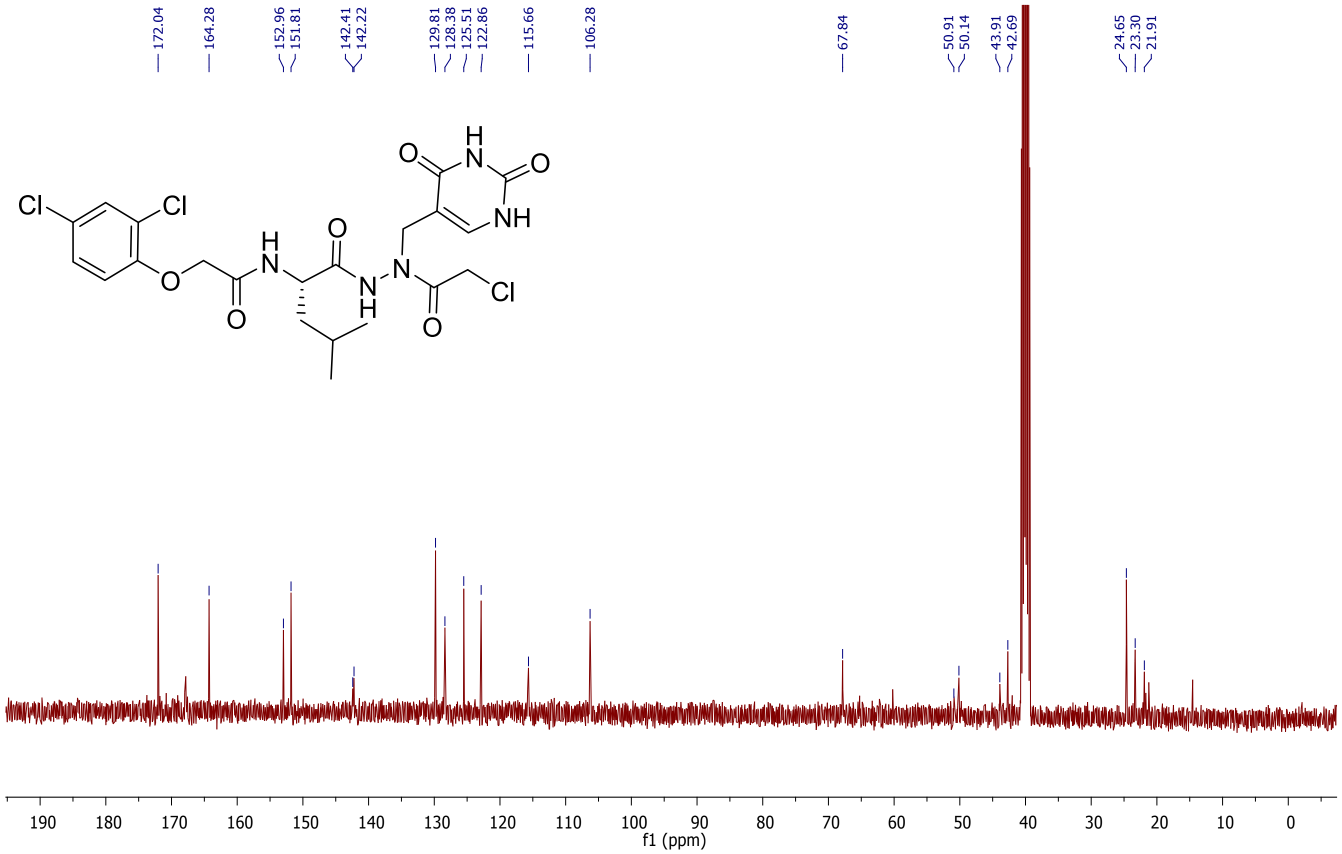
172.04
164.28
152.96
151.81
142.41
142.22
129.81
128.38
125.51
122.86
115.66
106.28
67.84
50.91
50.14
43.91
42.69
24.65
23.30
21.91
Cl
Cl
O
O
H
N
O
N
H
N
O
Cl
O
H
N
O
NH
f1 (ppm)

14c

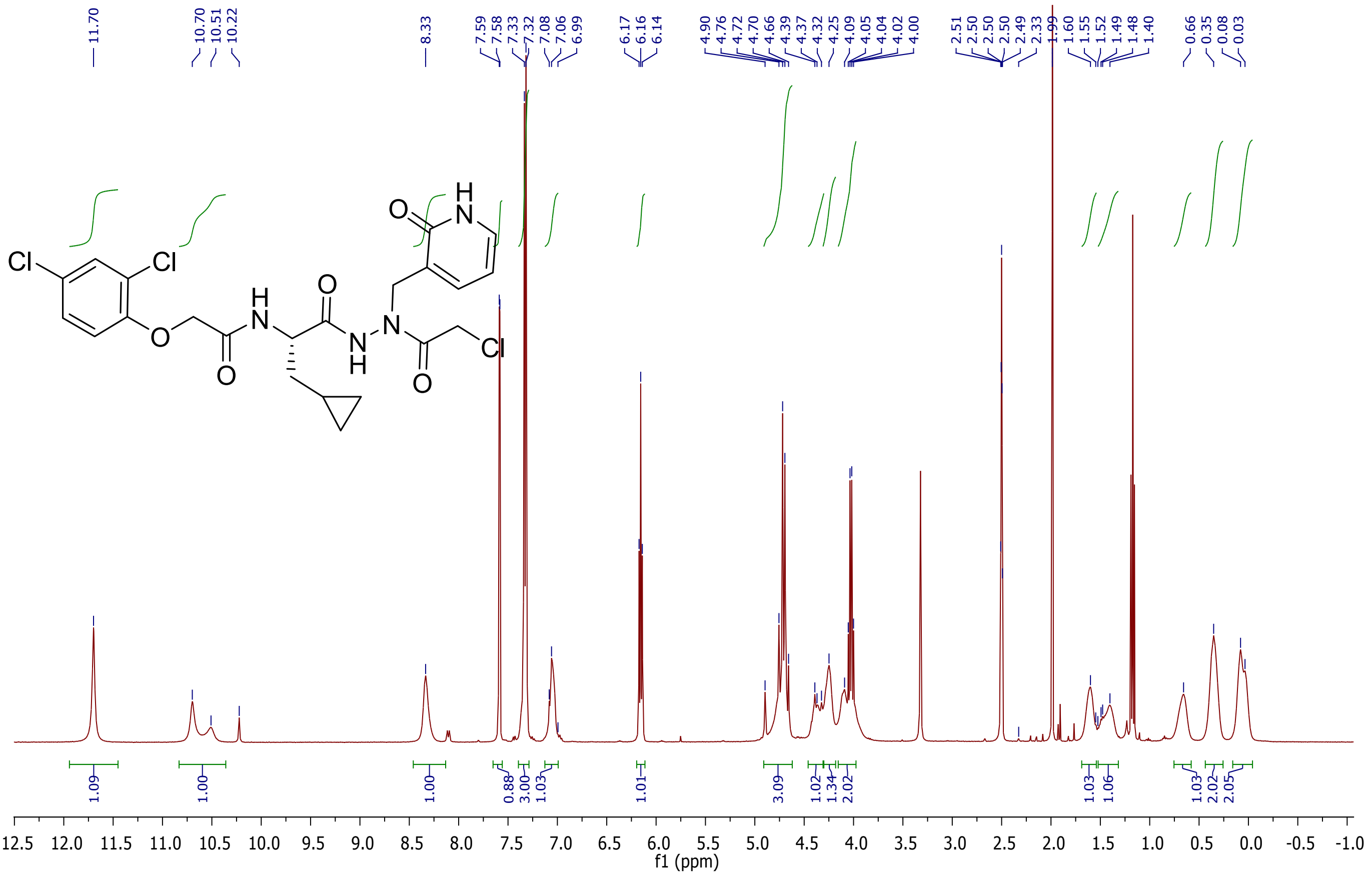

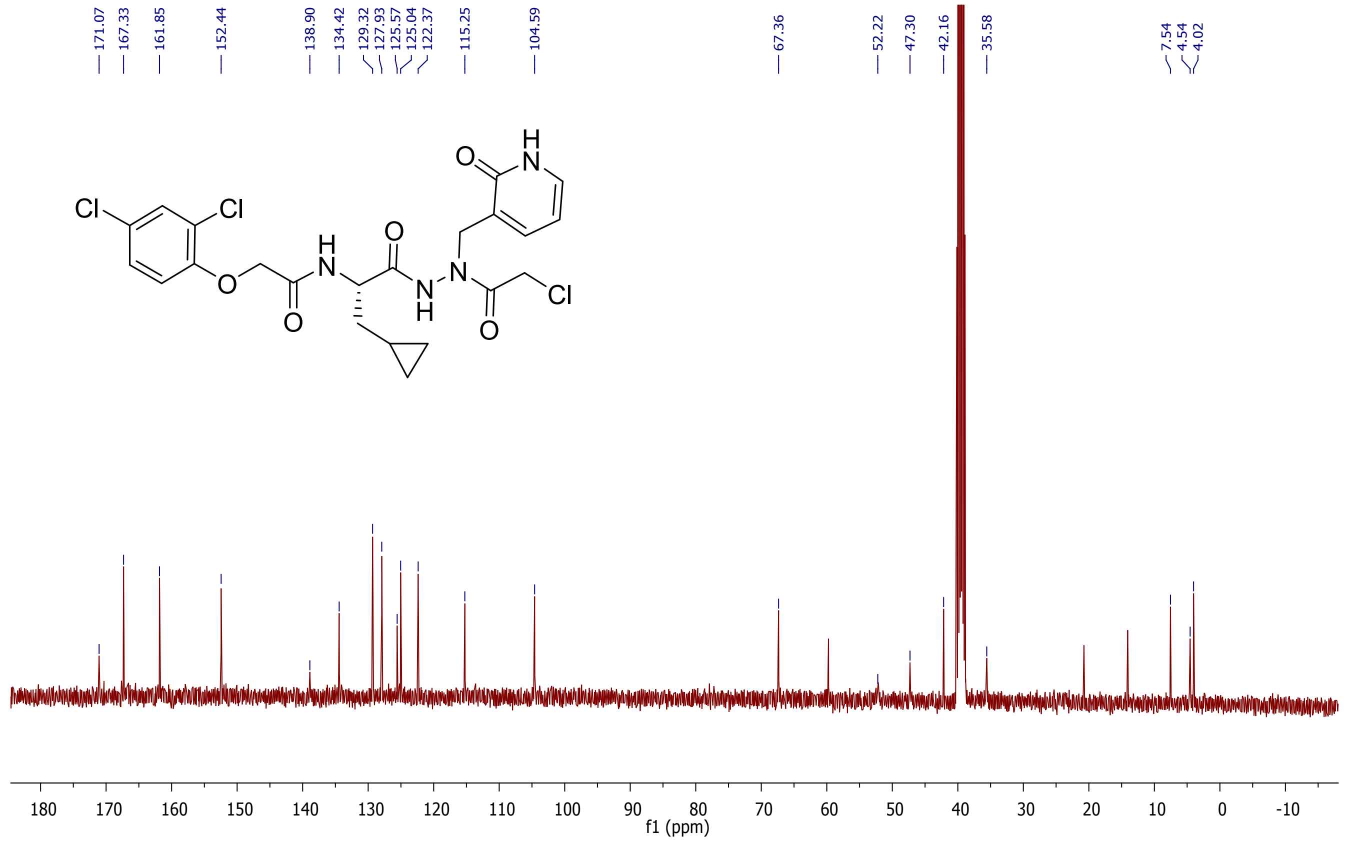

14d

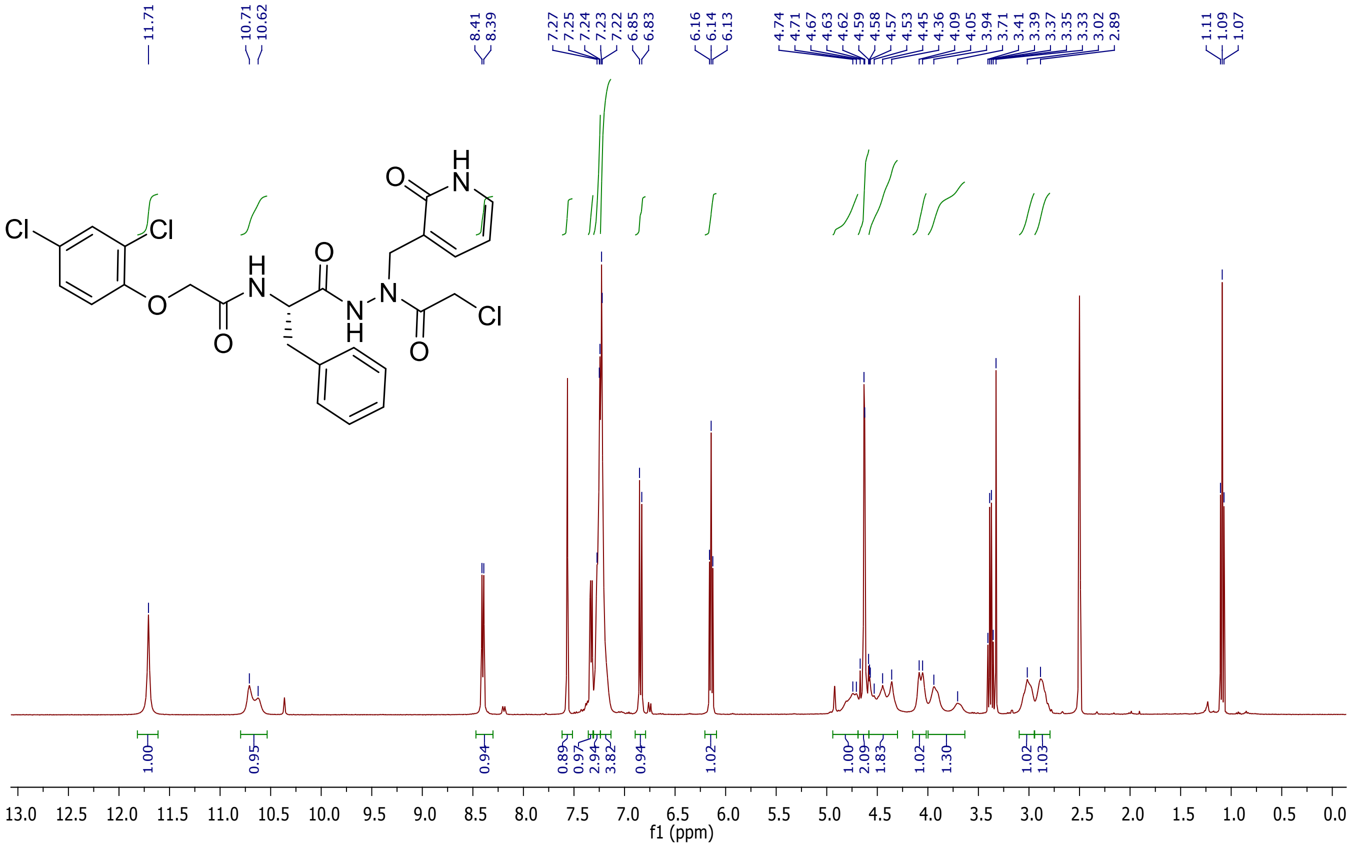

11.71
10.71
10.62
8.41
8.39
7.27
7.25
7.24
7.23
7.22
6.85
6.83
6.16
6.14
6.13
4.74
4.71
4.67
4.63
4.62
4.59
4.58
4.57
4.53
4.45
4.36
4.09
4.05
3.94
3.71
3.41
3.39
3.37
3.35
3.33
3.02
2.89
1.11
1.09
1.07
1.00
0.95
0.94
0.89
0.97
2.94
3.82
0.94
1.02
1.00
2.09
1.83
1.02
1.30
1.02
1.03
f1 (ppm)

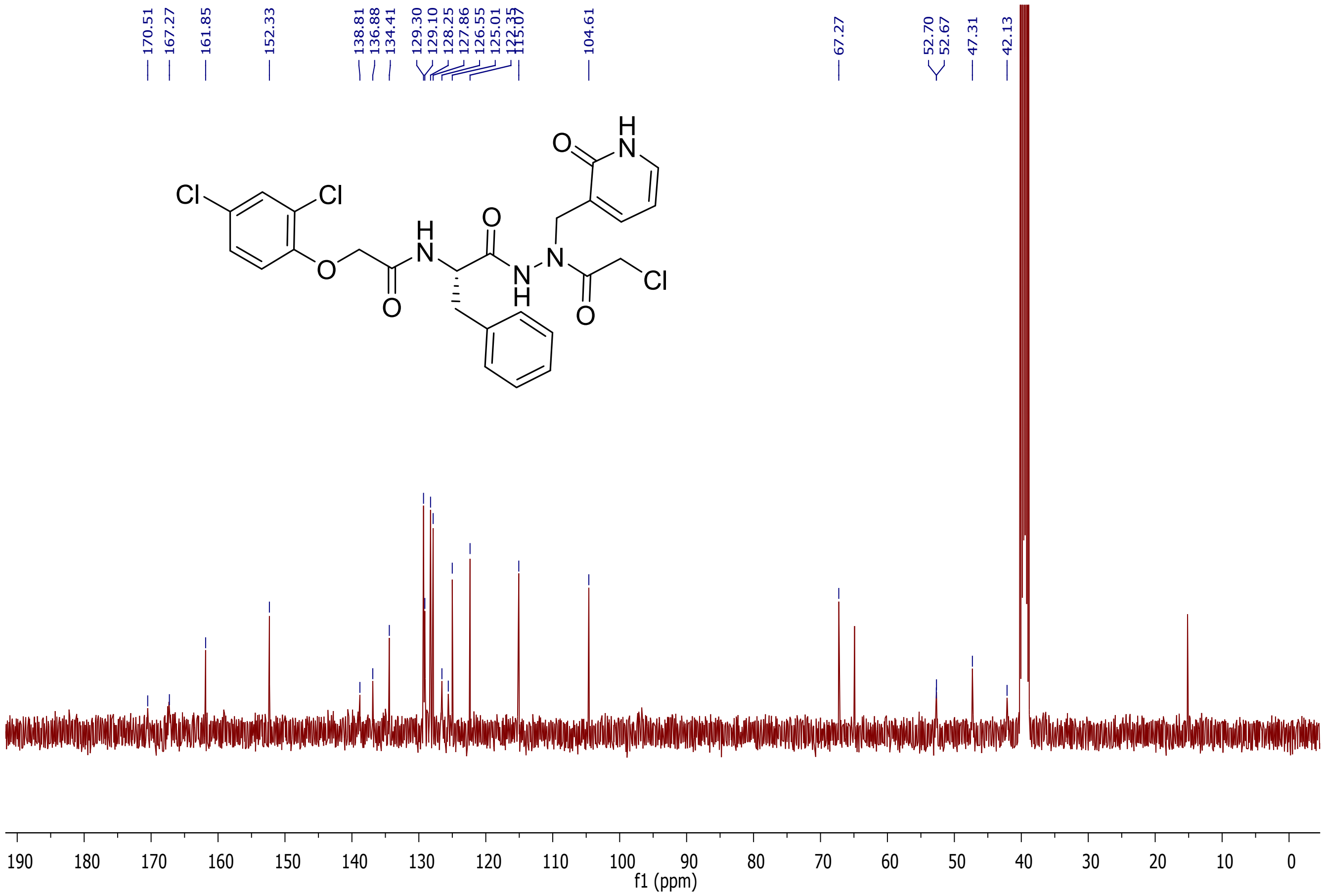

170.51
167.27
161.85
152.33
138.81
136.88
134.41
129.30
129.10
128.25
127.86
126.55
125.01
104.61
67.27
52.70
52.67
47.31
42.13
f1 (ppm)

14f

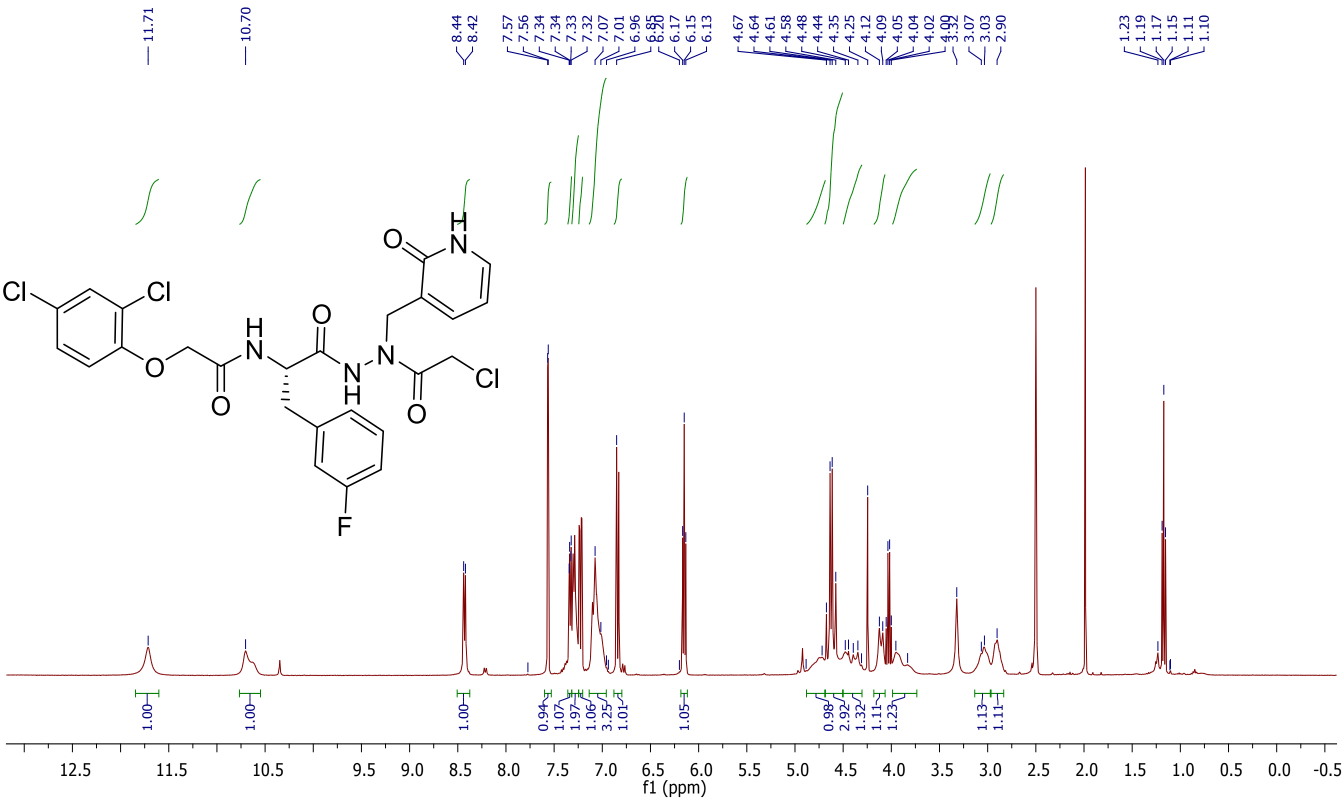

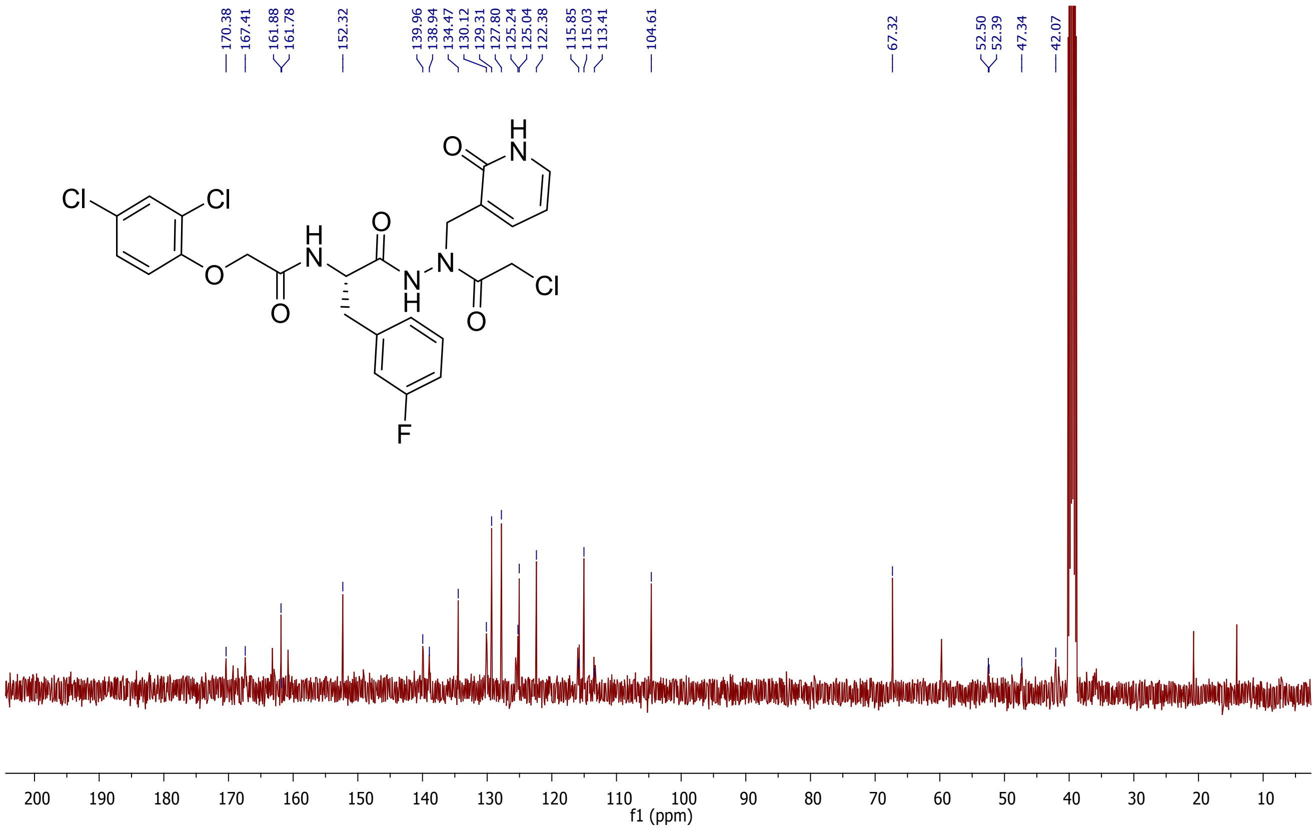

14g

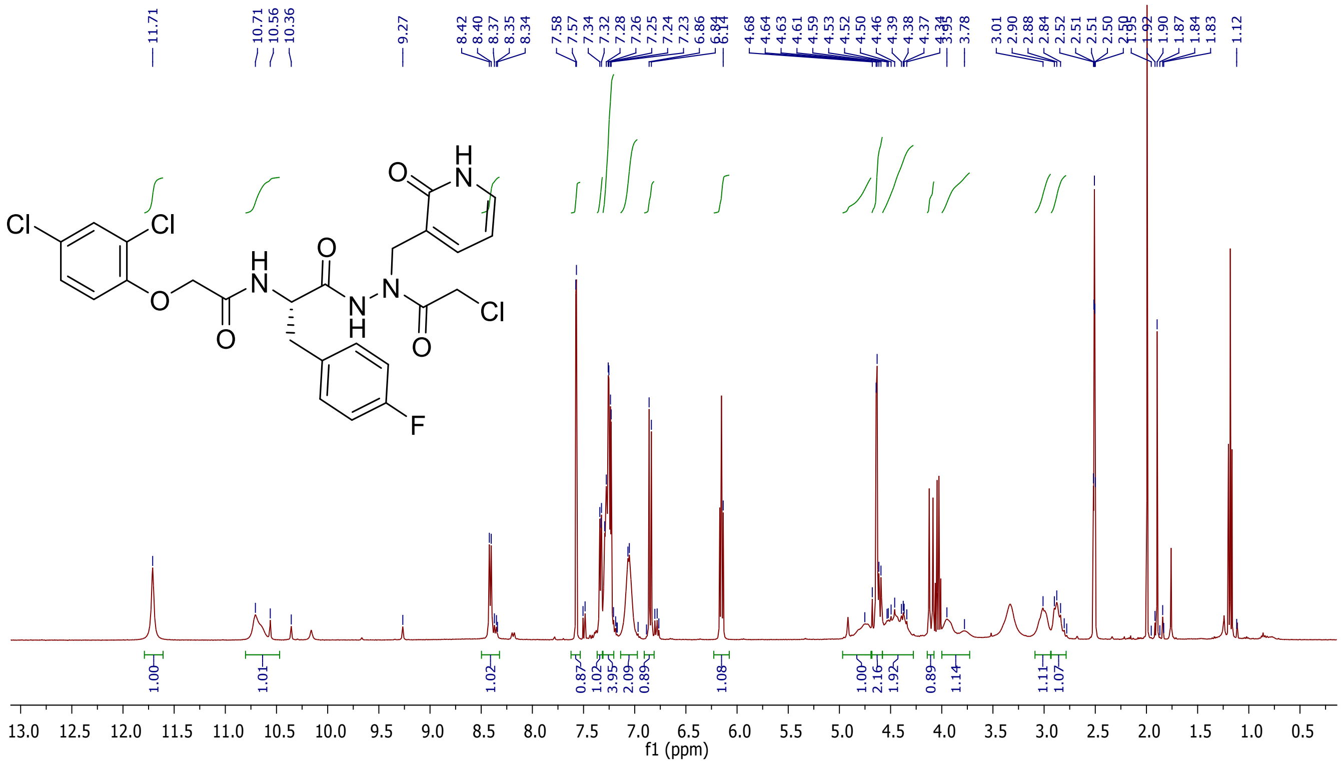

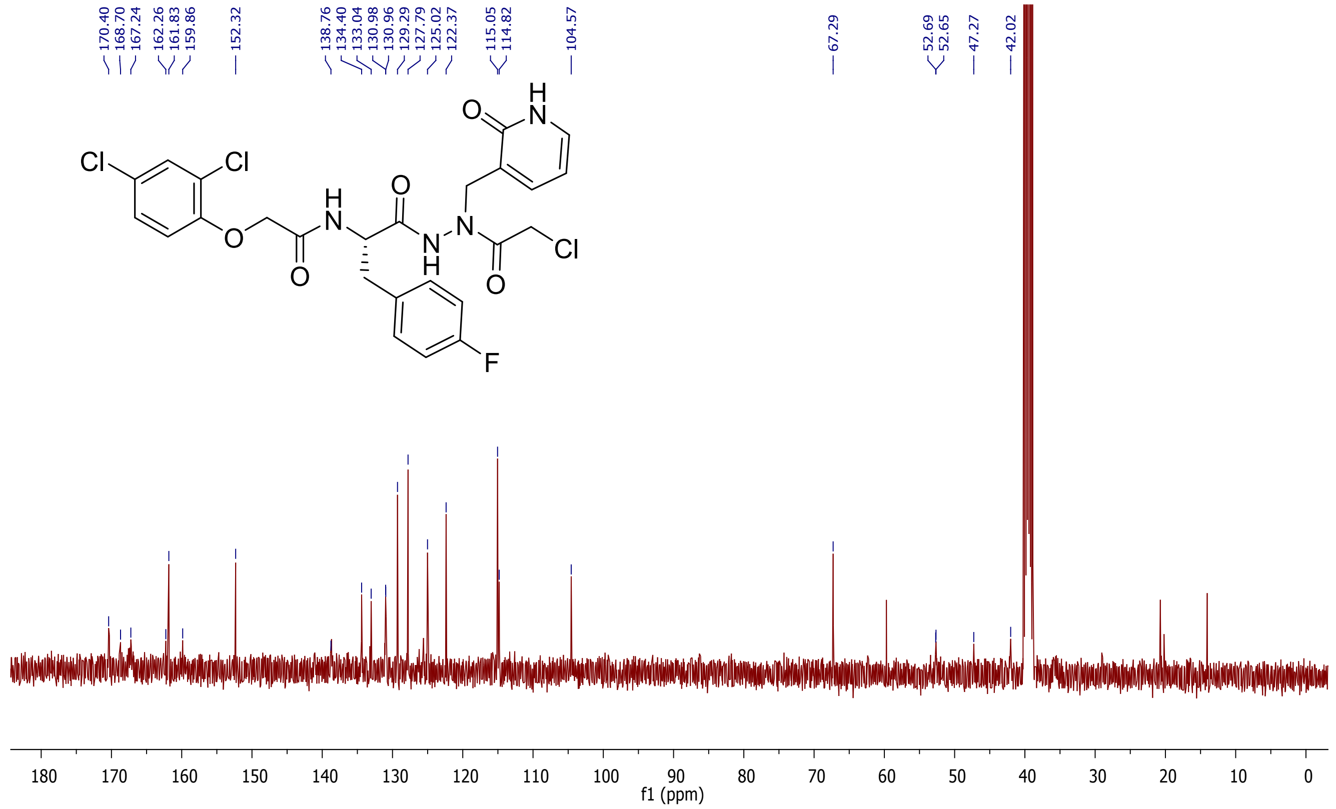

14h

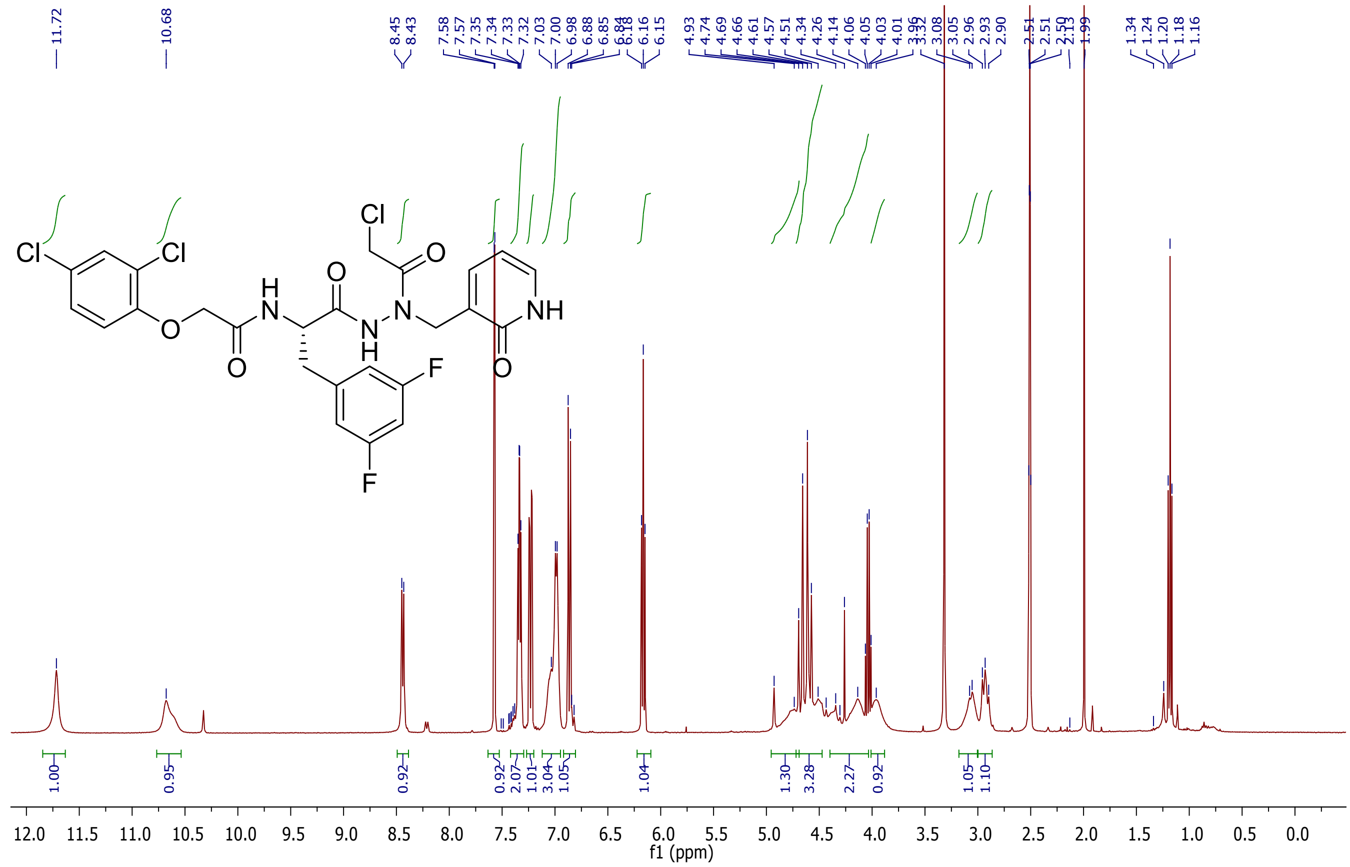

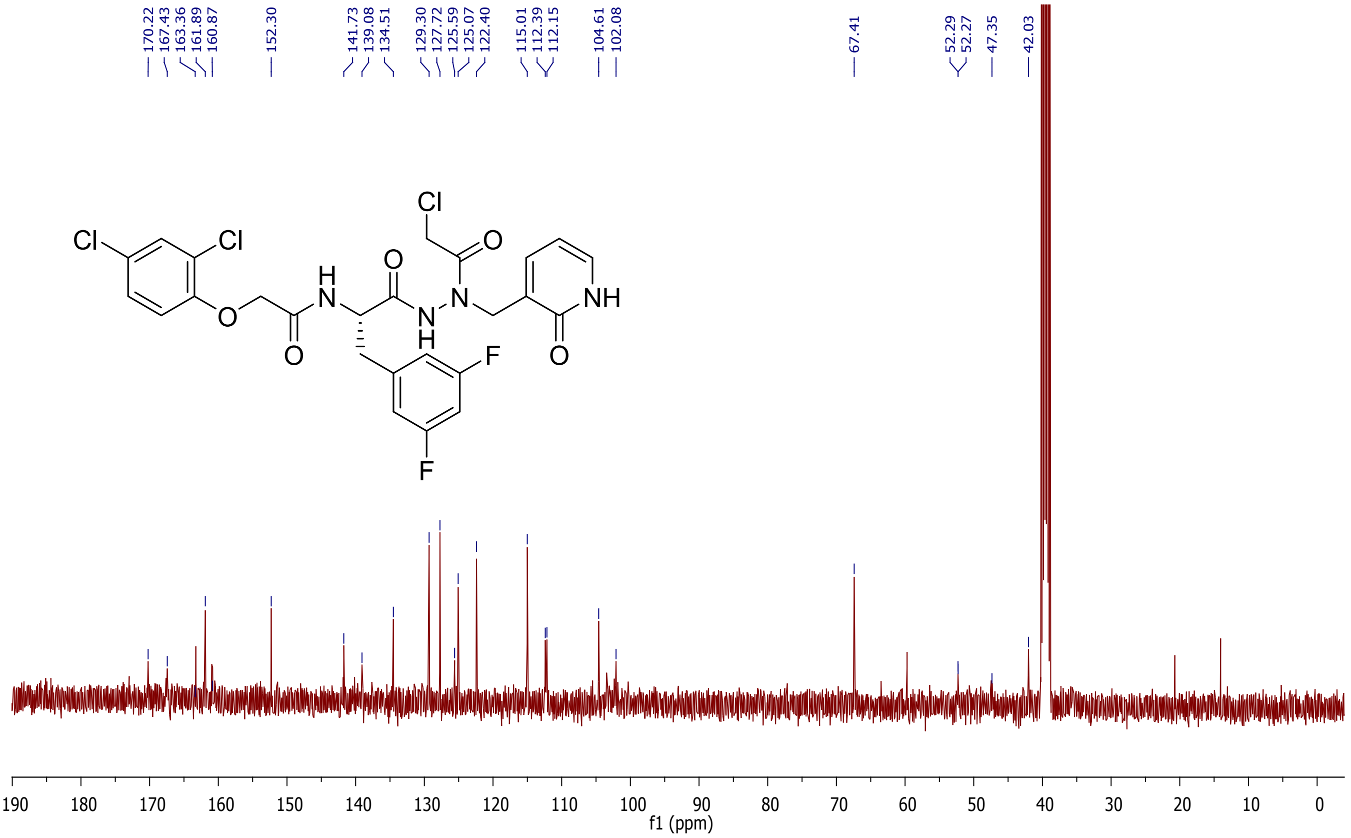

14j

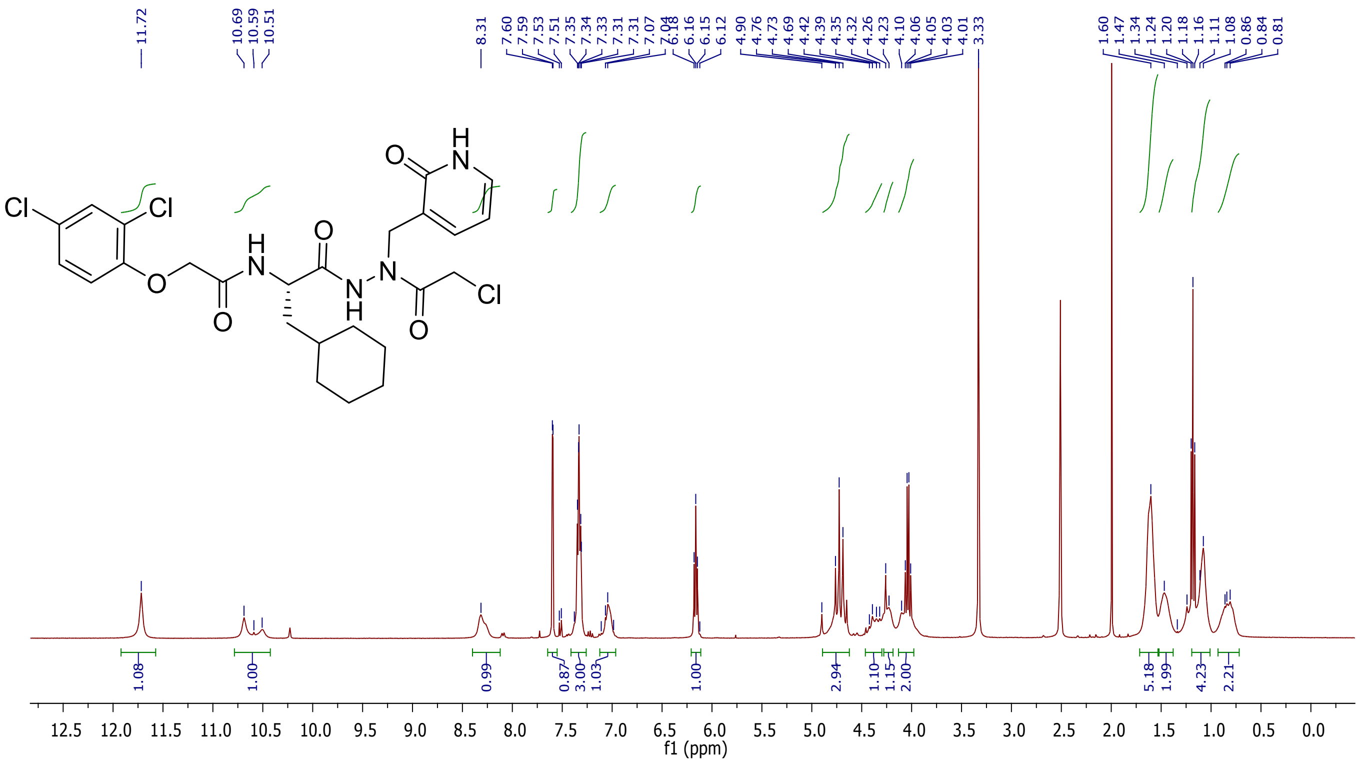

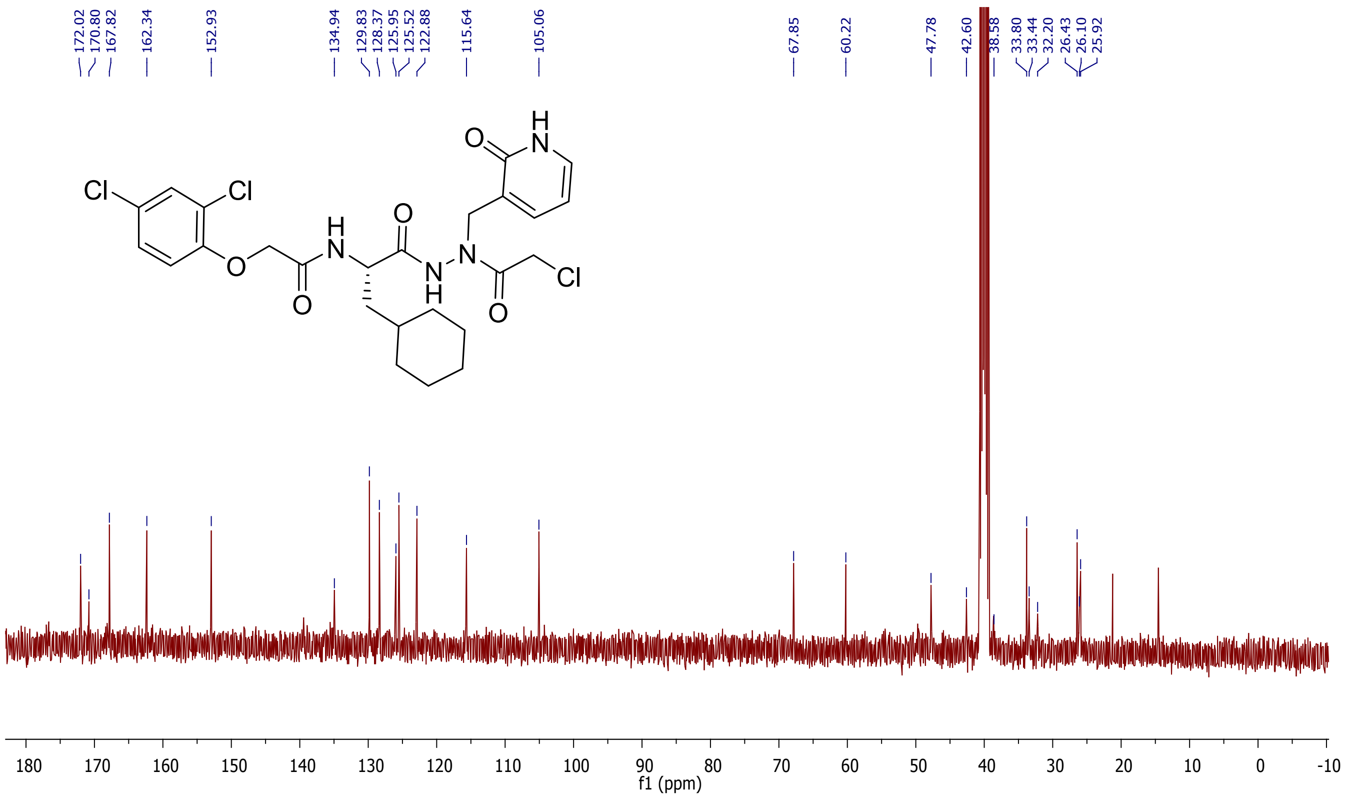

14k

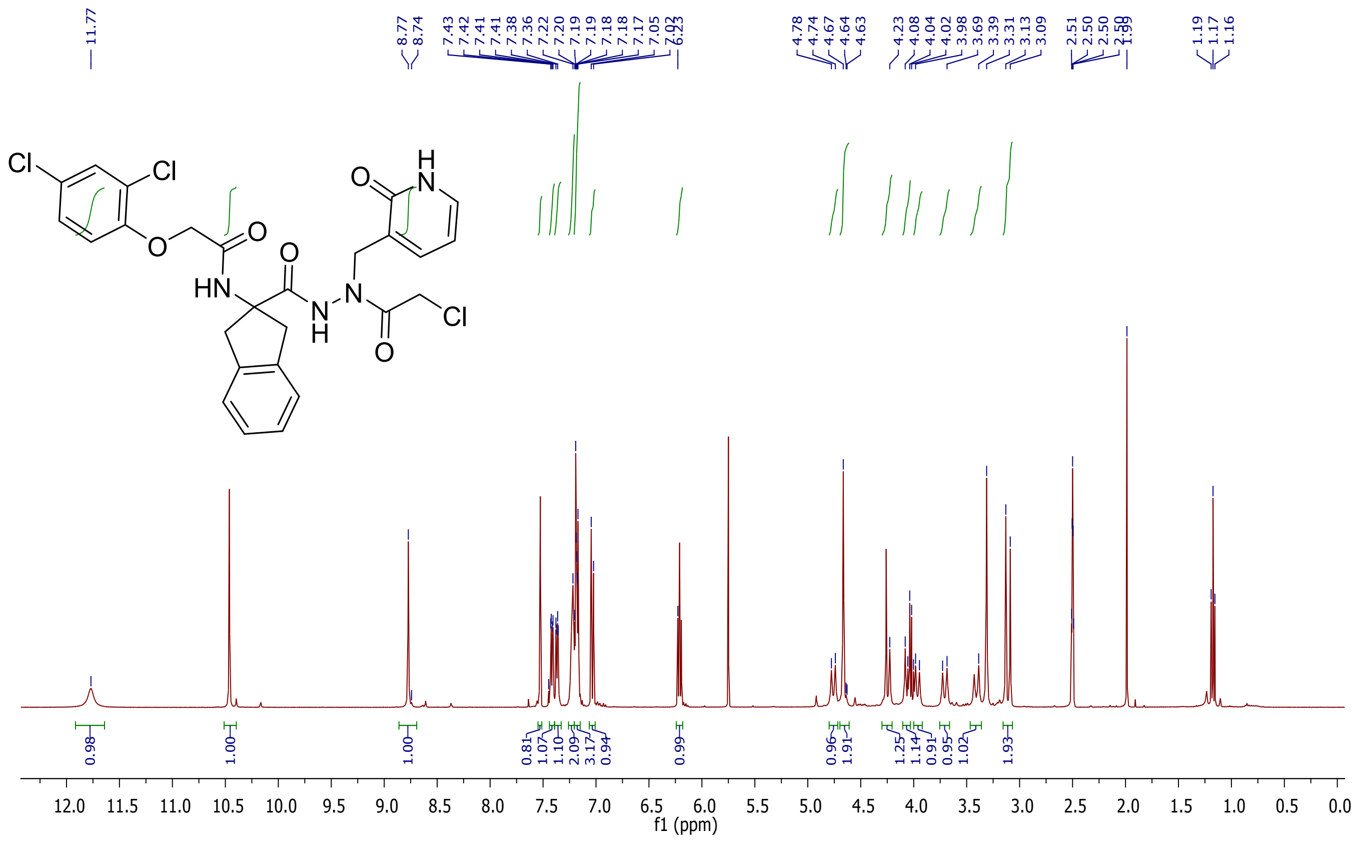

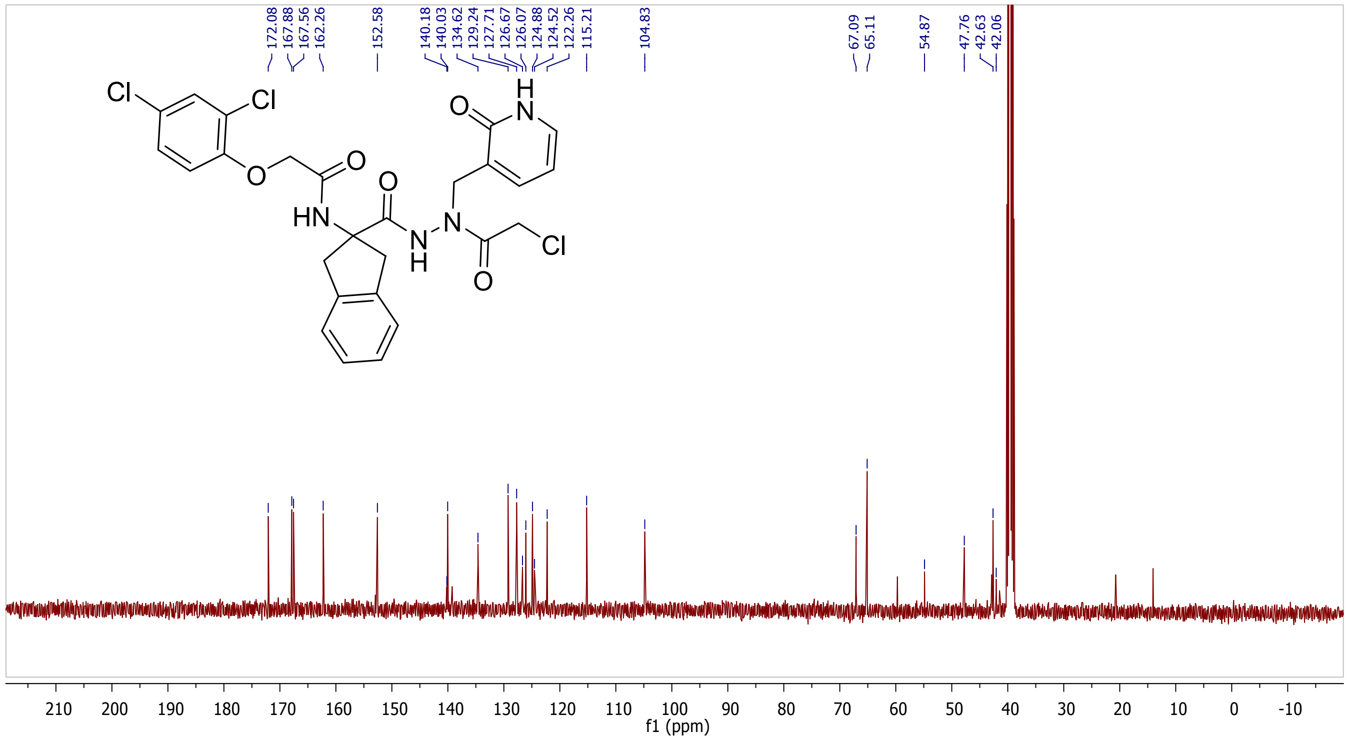

14m

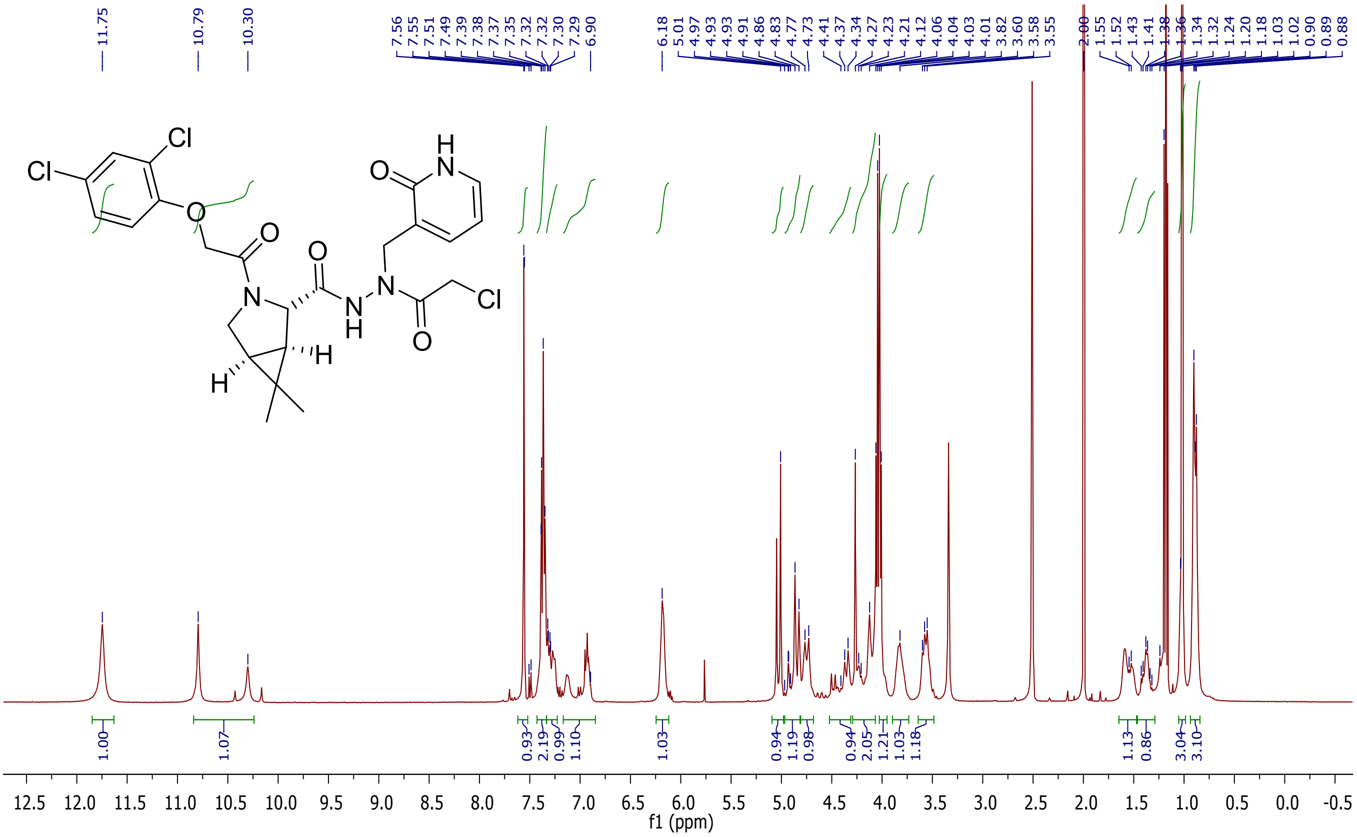
11.75
10.79
10.30
7.56 7.55 7.51 7.49 7.39 7.38 7.37 7.35 7.32 7.32 7.30 7.29 6.90
6.18 5.01 4.97 4.93 4.93 4.91 4.86 4.83 4.77 4.73 4.41 4.37 4.34 4.27 4.23 4.21 4.12 4.06 4.04 4.03 4.01 3.82 3.60 3.58 3.55
2.00 1.55 1.52 1.43 1.41 1.38 1.36 1.34 1.32 1.24 1.20 1.18 1.03 1.02 0.90 0.89 0.88
Cl
Cl
O
O
O
N
N
H
N
H
O
Cl
O
H
H
1.00
1.07
0.93 2.19 0.99 1.10
1.03
0.94 1.19 0.98
0.94 2.05 1.21 1.03 1.18
1.13 0.86
3.04 3.10
f1 (ppm)

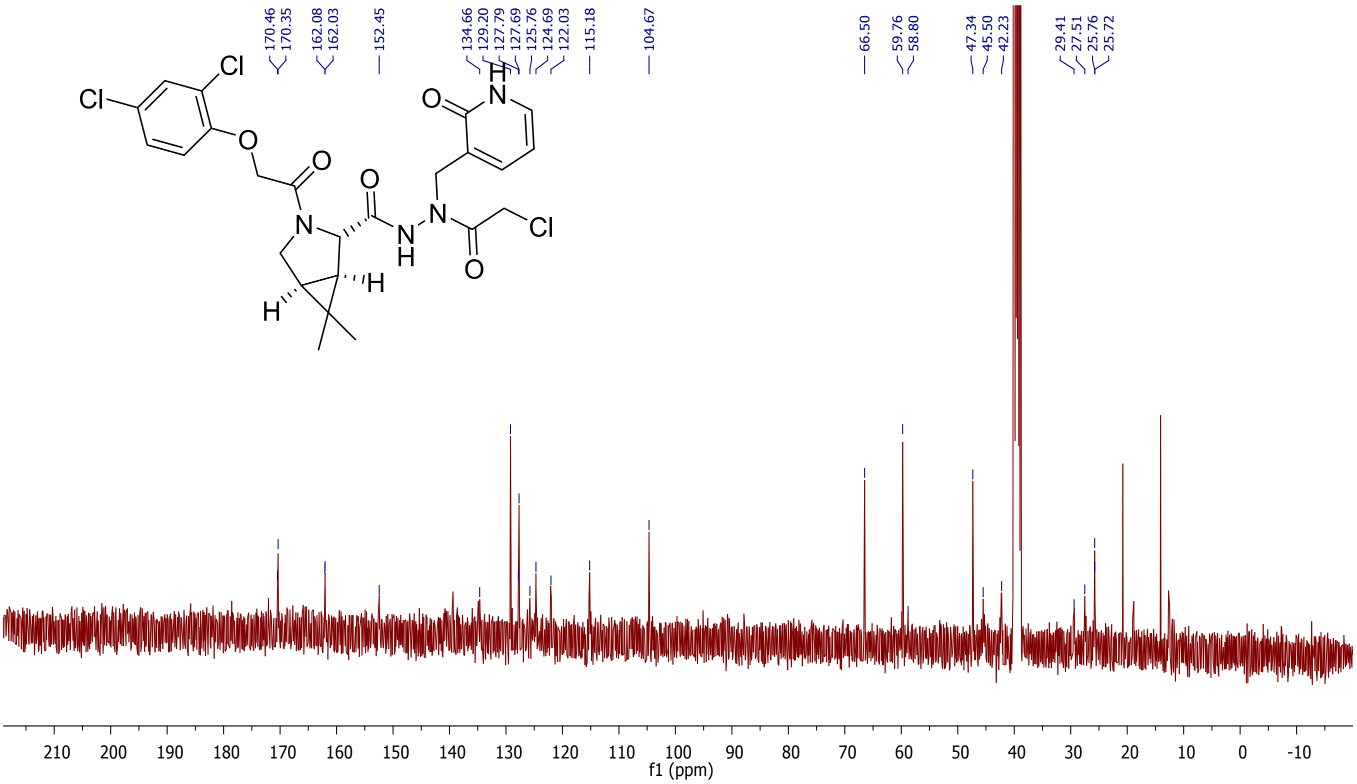
170.46 170.35
162.08 162.03
152.45
134.66 129.20 127.79 127.69 125.76 124.69 122.03
115.18
104.67
66.50
59.76 58.80
47.34 45.50 42.23
29.41 27.51 25.76 25.72
f1 (ppm)

14r

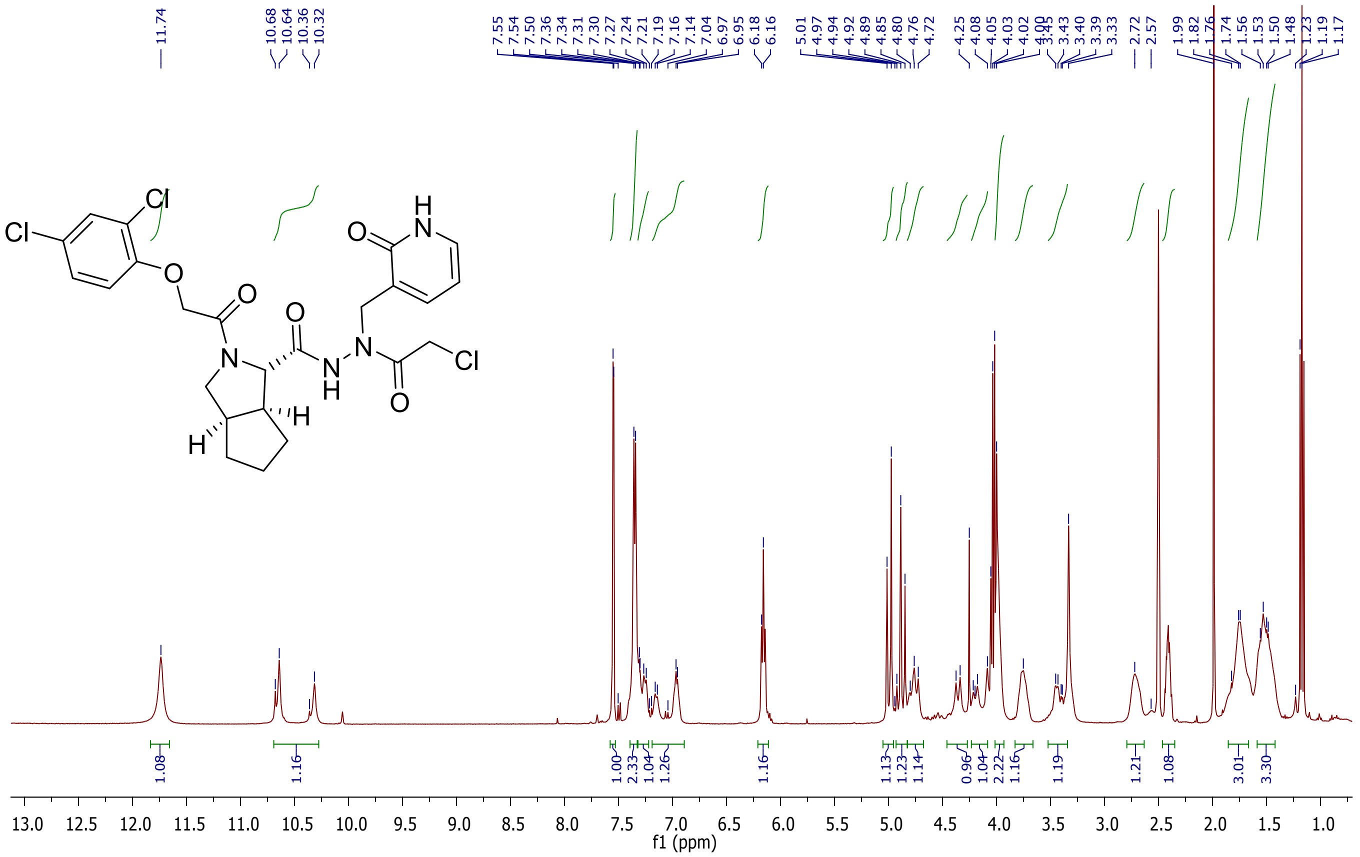
11.74
10.68 10.64 10.36 10.32
7.55 7.54 7.50 7.36 7.34 7.31 7.30 7.27 7.24 7.21 7.19 7.16 7.14 7.04 6.97 6.95 6.18 6.16
5.01 4.97 4.94 4.92 4.89 4.85 4.80 4.76 4.72
4.25 4.08 4.05 4.03 4.02 4.00 3.45 3.43 3.40 3.39 3.33
2.72 2.57
1.99 1.82 1.76 1.74 1.56 1.53 1.50 1.48 1.23 1.19 1.17
Cl
Cl
O
O
O
N
N
H
N
H
N
O
Cl
O
H
H
1.08 1.16 1.00 2.33 1.04 1.26 1.16 1.13 1.23 1.14 0.96 1.04 2.22 1.16 1.19 1.21 1.08 3.01 3.30
13.0 12.5 12.0 11.5 11.0 10.5 10.0 9.5 9.0 8.5 8.0 7.5 7.0 6.5 6.0 5.5 5.0 4.5 4.0 3.5 3.0 2.5 2.0 1.5 1.0
f1 (ppm)

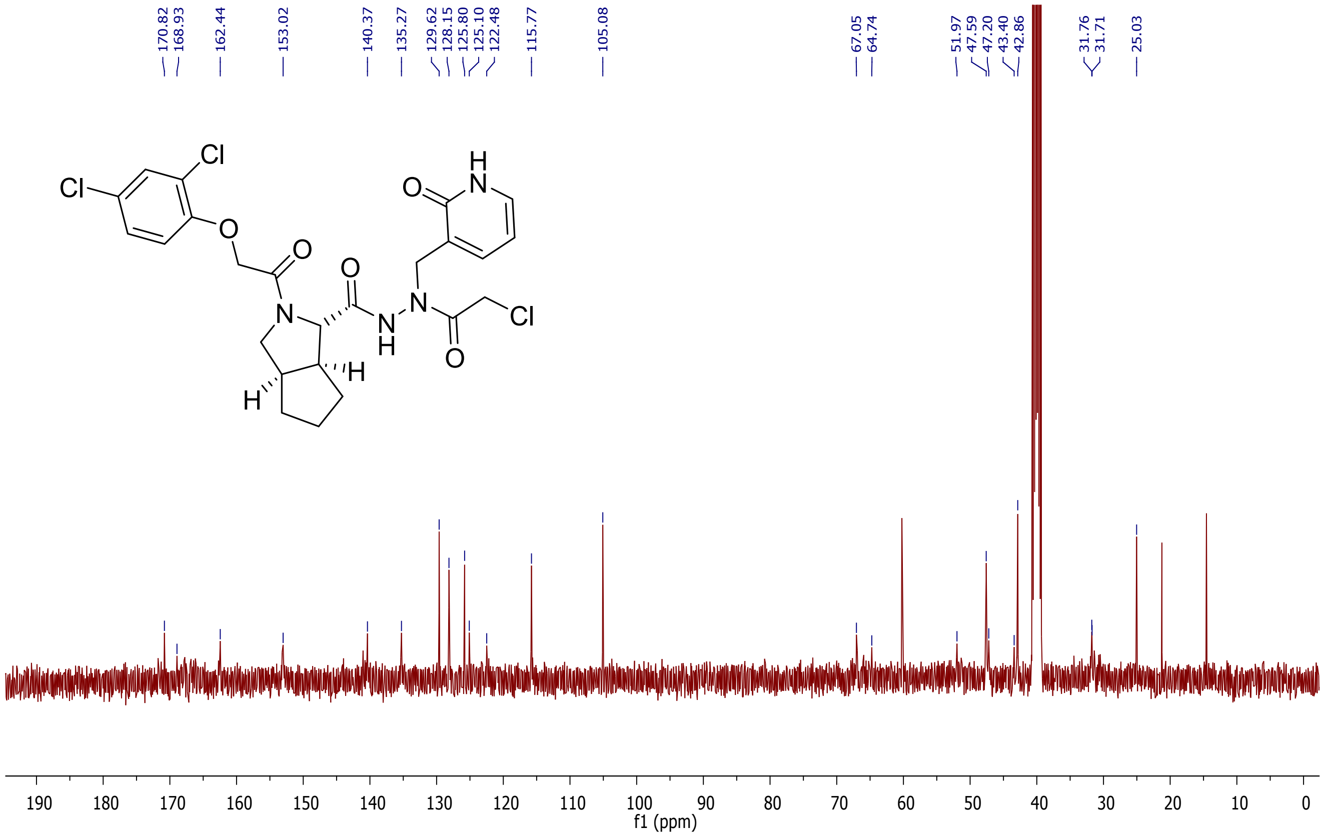
170.82 168.93 162.44 153.02 140.37 135.27 129.62 128.15 125.80 125.10 122.48 115.77 105.08
67.05 64.74
51.97 47.59 47.20 43.40 42.86
31.76 31.71 25.03
Cl
Cl
O
O
O
N
N
H
N
H
N
O
Cl
O
H
H
190 180 170 160 150 140 130 120 110 100 90 80 70 60 50 40 30 20 10 0
f1 (ppm)

20a

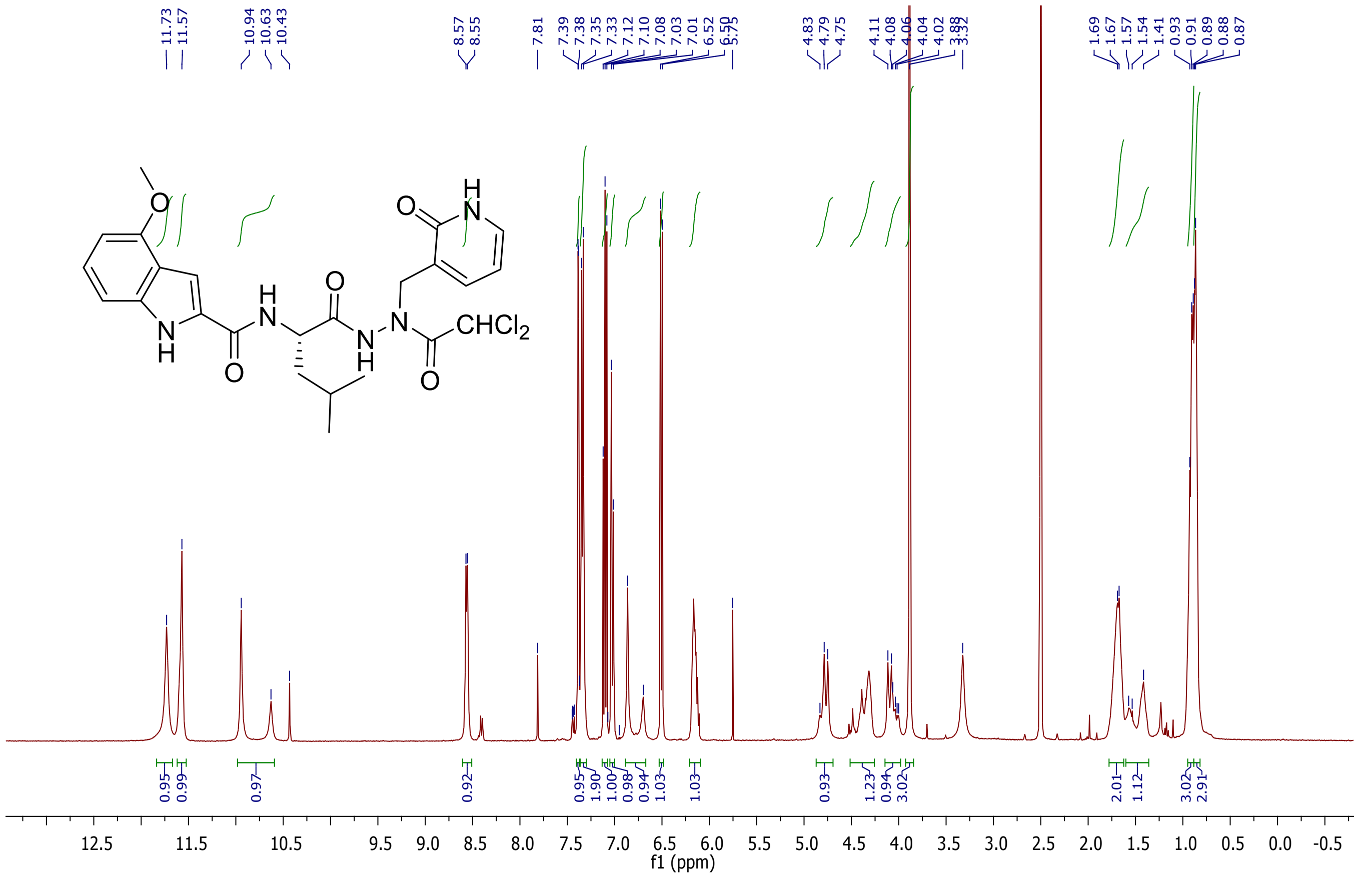

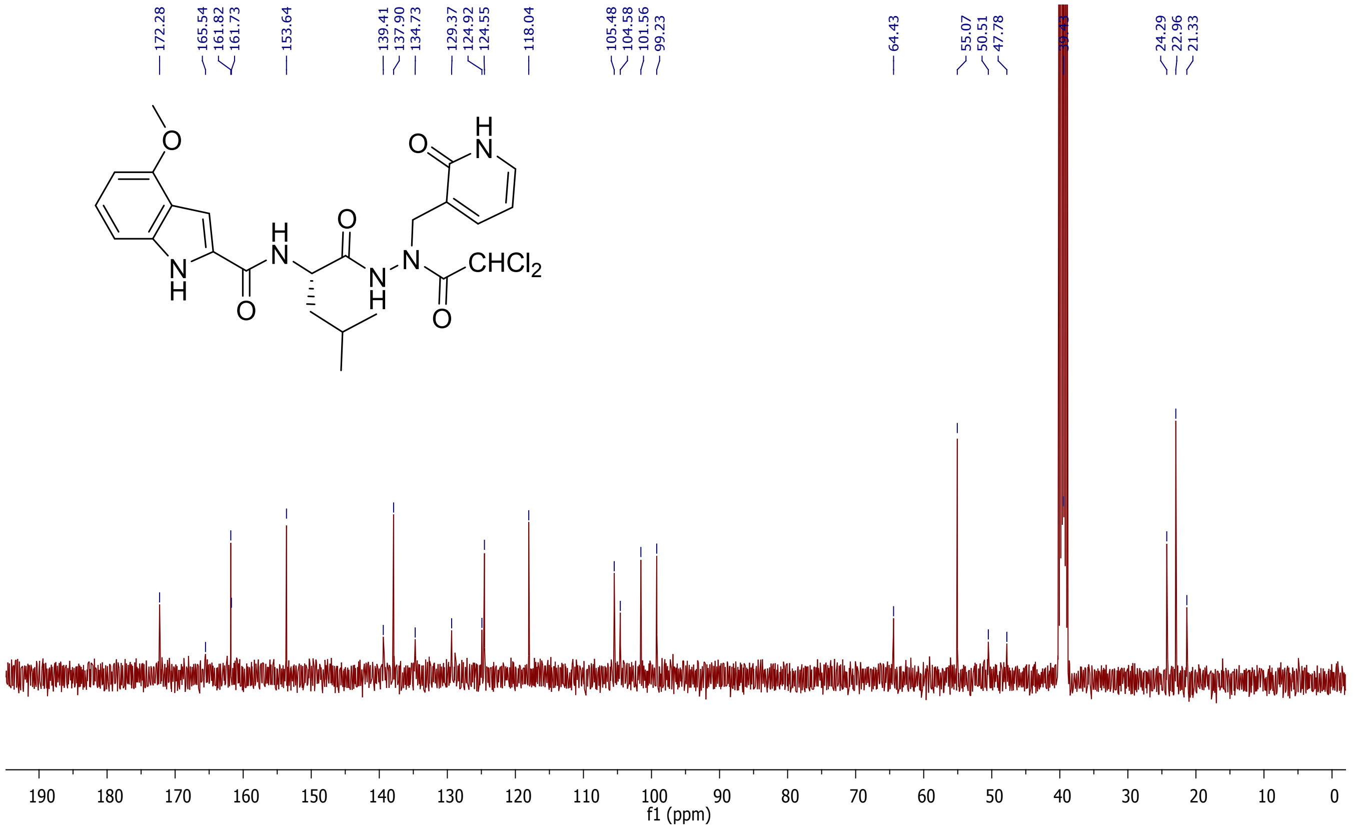

20b

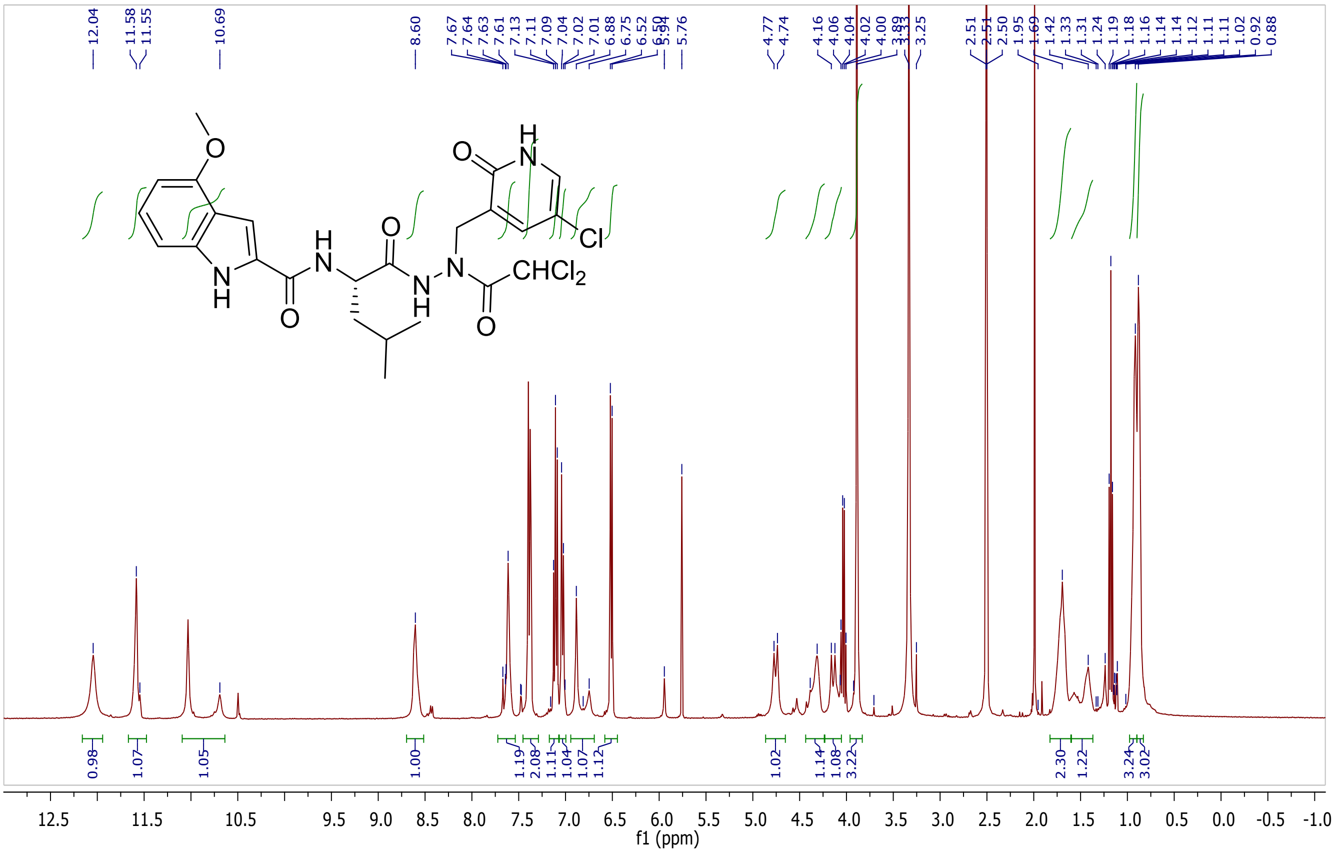
12.04
11.58
11.55
10.69
8.60
7.67
7.64
7.63
7.61
7.13
7.11
7.09
7.04
7.02
7.01
6.88
6.75
6.52
6.50
5.94
5.76
4.77
4.74
4.16
4.06
4.04
4.02
4.00
3.89
3.33
3.25
2.51
2.51
2.50
1.95
1.69
1.42
1.33
1.31
1.24
1.19
1.18
1.16
1.14
1.14
1.12
1.11
1.11
1.02
0.92
0.88
O
H
N
O
Cl
N
H
H
N
O
N
N
H
CHCl2
O
O
0.98
1.07
1.05
1.00
1.19
2.08
1.11
1.04
1.07
1.12
1.02
1.14
1.08
3.22
2.30
1.22
3.24
3.02
12.5 11.5 10.5 9.5 9.0 8.5 8.0 7.5 7.0 6.5 6.0 5.5 5.0 4.5 4.0 3.5 3.0 2.5 2.0 1.5 1.0 0.5 0.0 -0.5 -1.0
f1 (ppm)

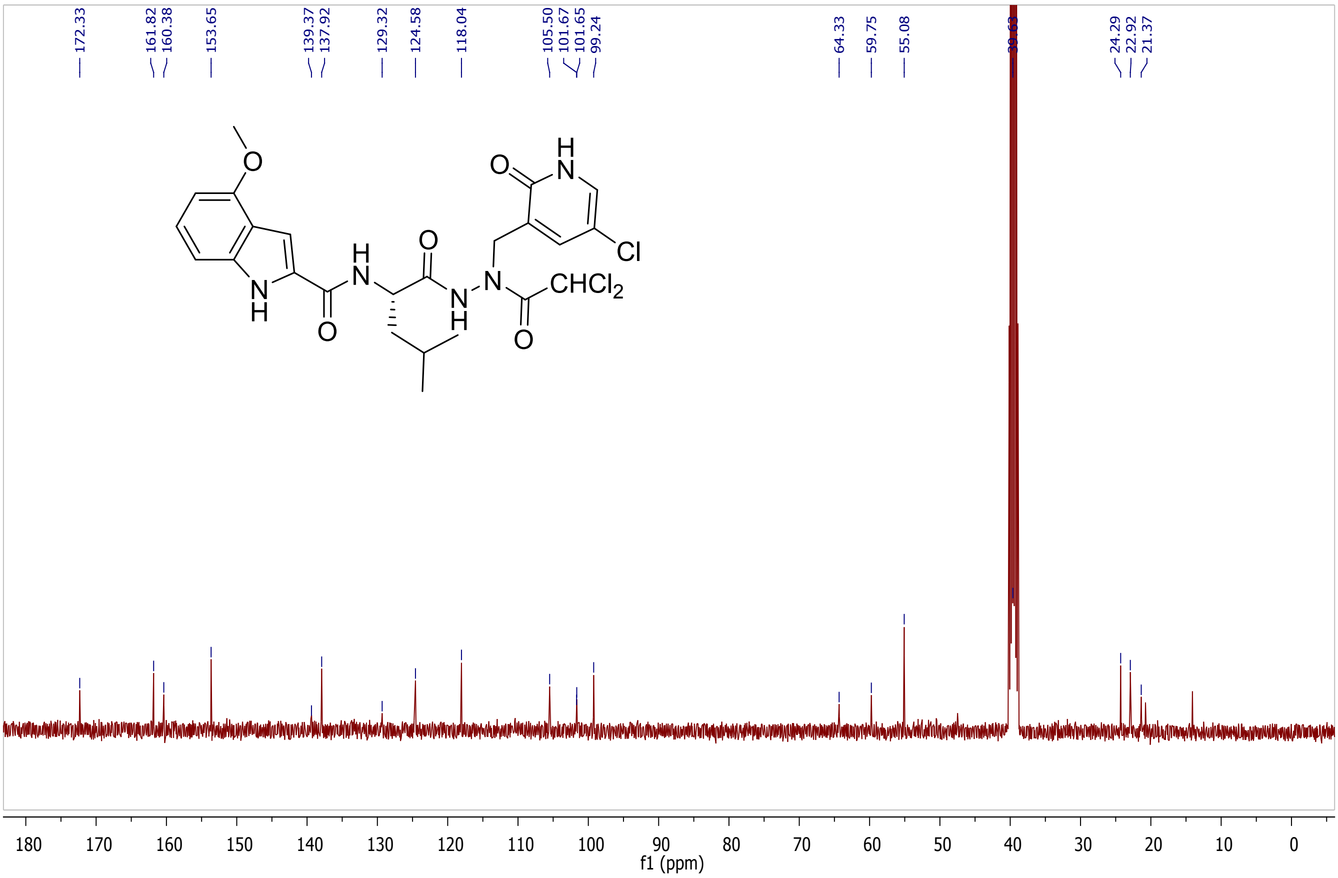
172.33
161.82
160.38
153.65
139.37
137.92
129.32
124.58
118.04
105.50
101.67
101.65
99.24
64.33
59.75
55.08
39.63
24.29
22.92
21.37
O
H
N
O
Cl
N
H
H
N
O
N
N
H
CHCl2
O
O
180 170 160 150 140 130 120 110 100 90 80 70 60 50 40 30 20 10 0
f1 (ppm)

20f

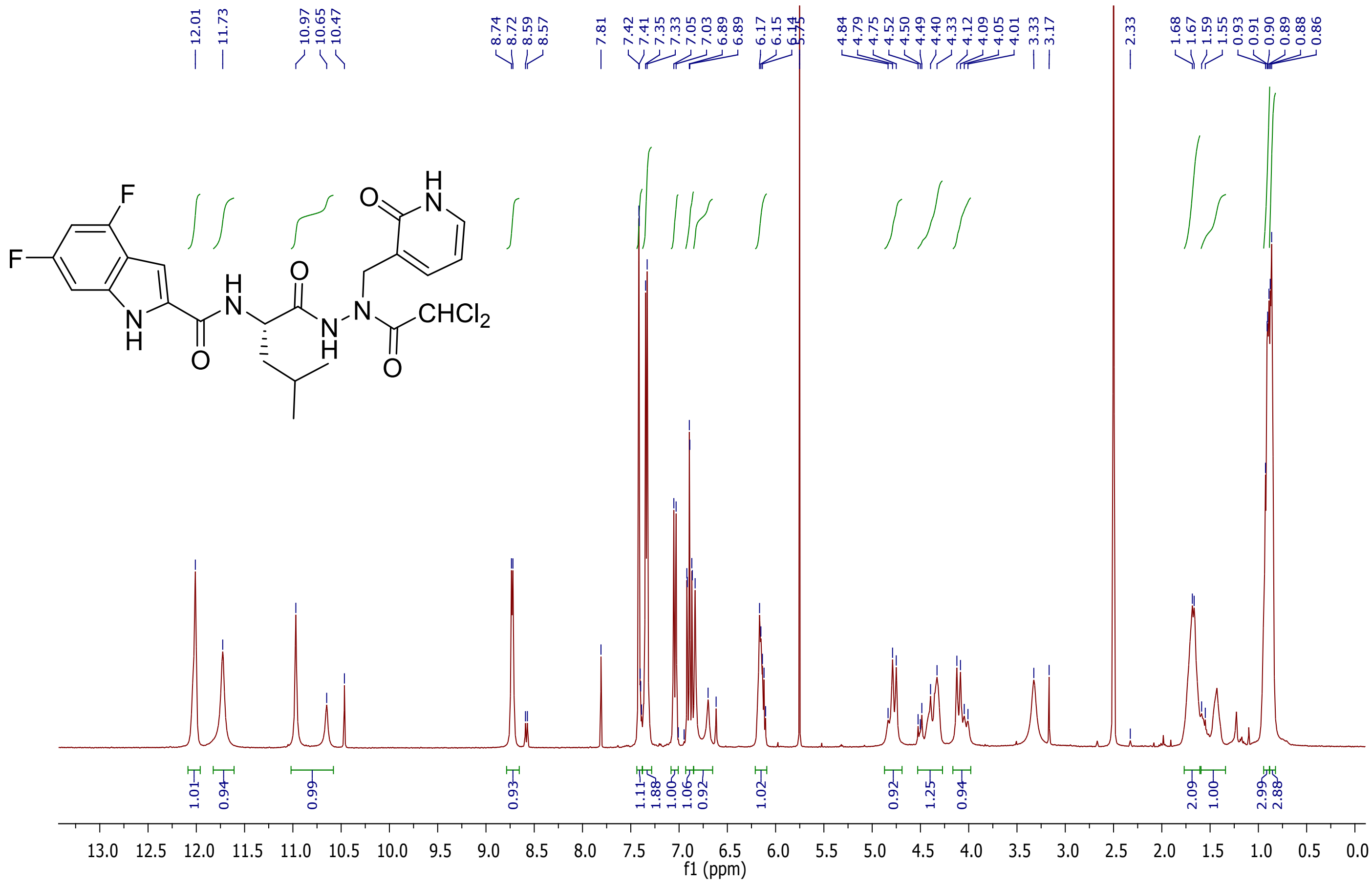
12.01
11.73
10.97
10.65
10.47
8.74
8.72
8.59
8.57
7.81
7.42
7.41
7.35
7.33
7.05
7.03
6.89
6.89
6.17
6.15
6.14
5.75
4.84
4.79
4.75
4.52
4.50
4.49
4.40
4.33
4.12
4.09
4.05
4.01
3.33
3.17
2.33
1.68
1.67
1.59
1.55
0.93
0.91
0.90
0.89
0.88
0.86
F
F
N
H
H
N
O
O
O
N
H
N
H
N
CHCl2
O
1.01
0.94
0.99
0.93
1.11
1.88
1.00
1.06
0.92
1.02
0.92
1.25
0.94
2.09
1.00
2.99
2.88
f1 (ppm)

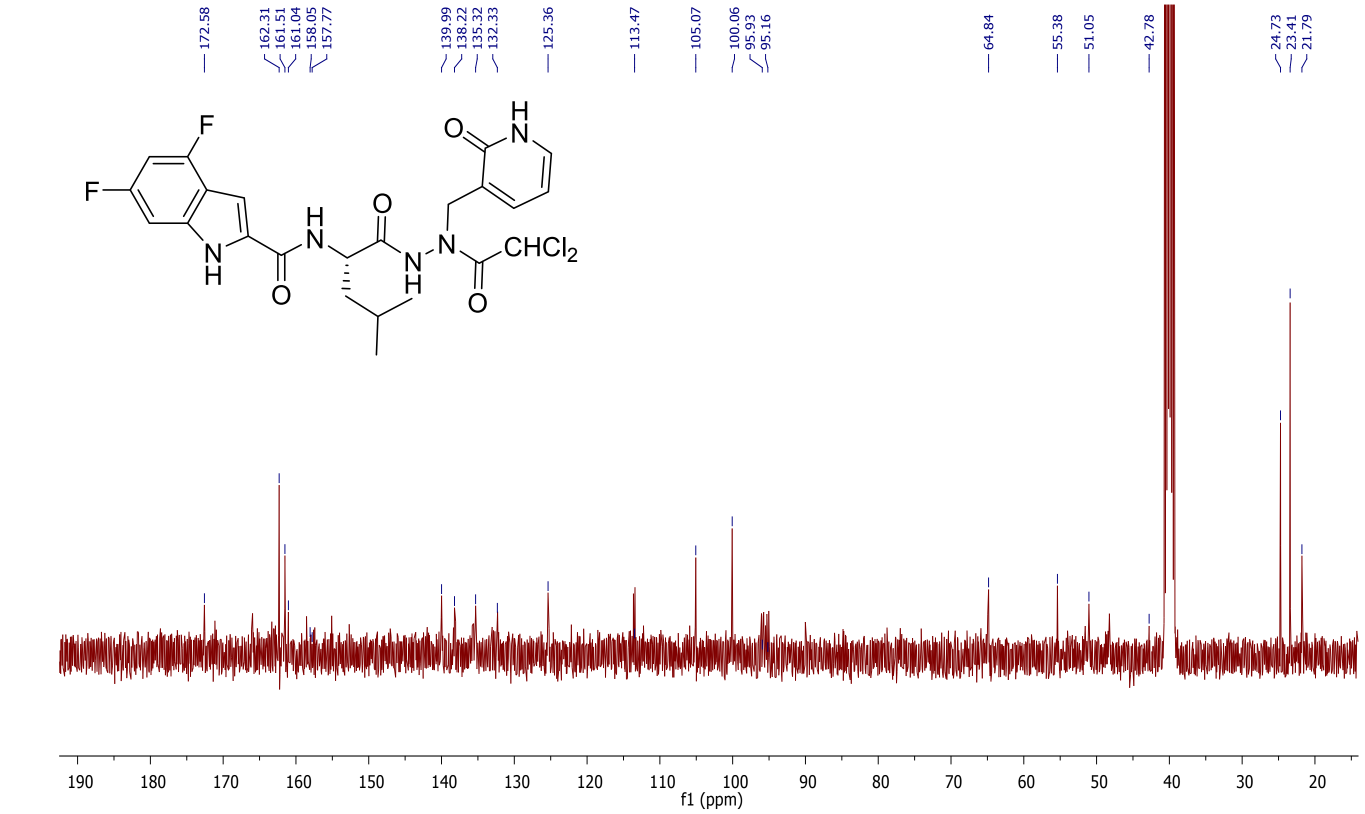
172.58
162.31
161.51
161.04
158.05
157.77
139.99
138.22
135.32
132.33
125.36
113.47
105.07
100.06
95.93
95.16
64.84
55.38
51.05
42.78
24.73
23.41
21.79
F
F
N
H
H
N
O
O
O
N
H
N
H
N
CHCl2
O
f1 (ppm)

20g

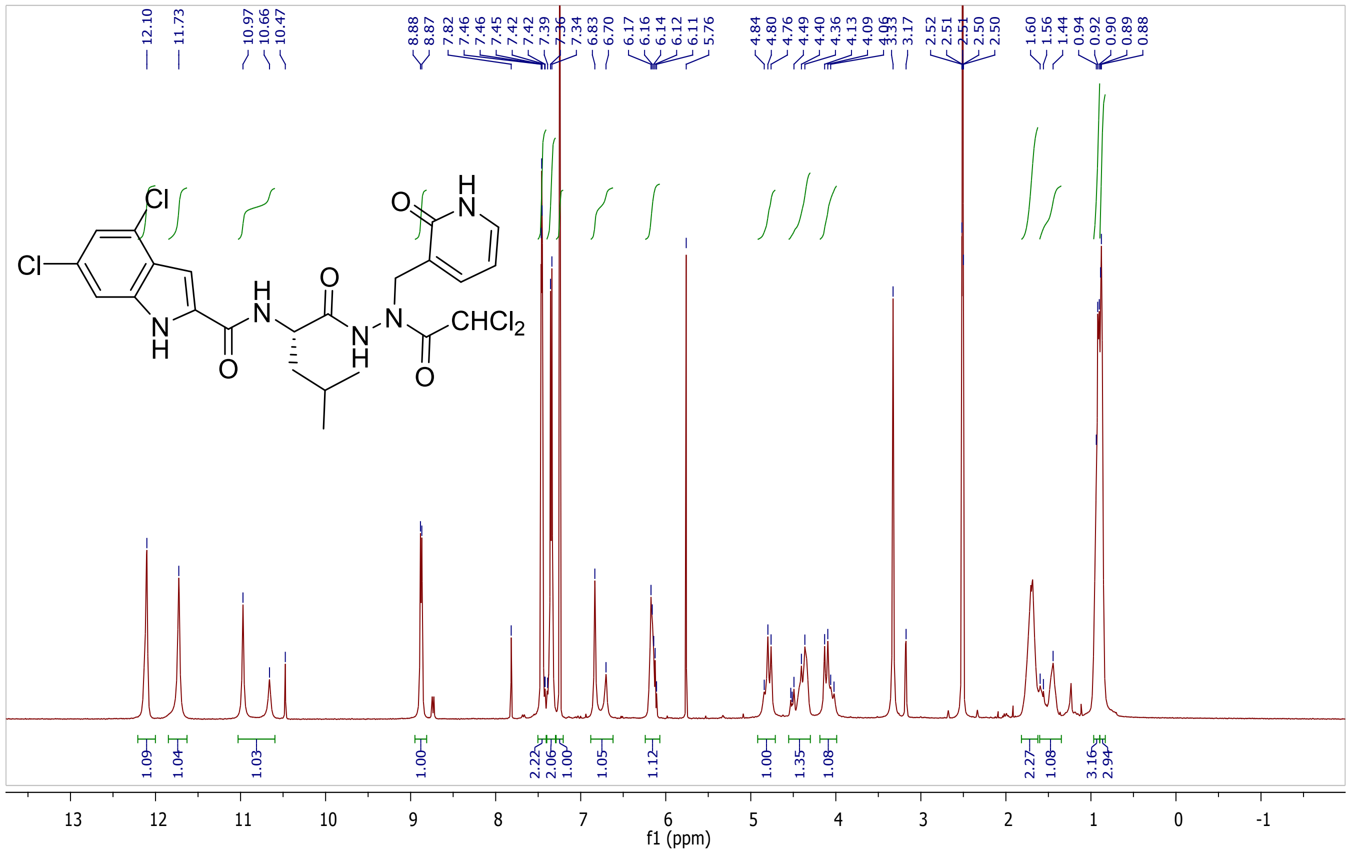

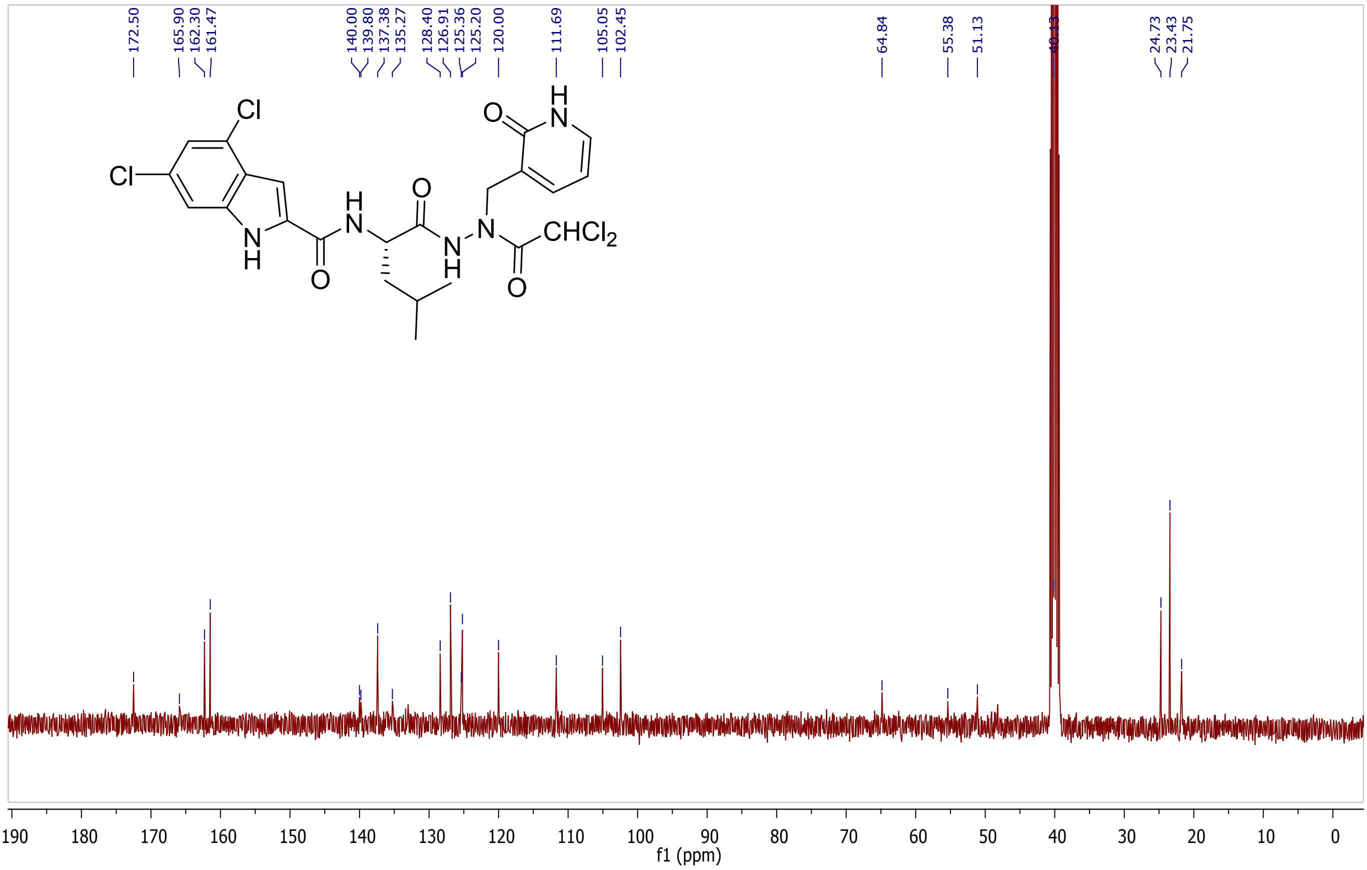

20h

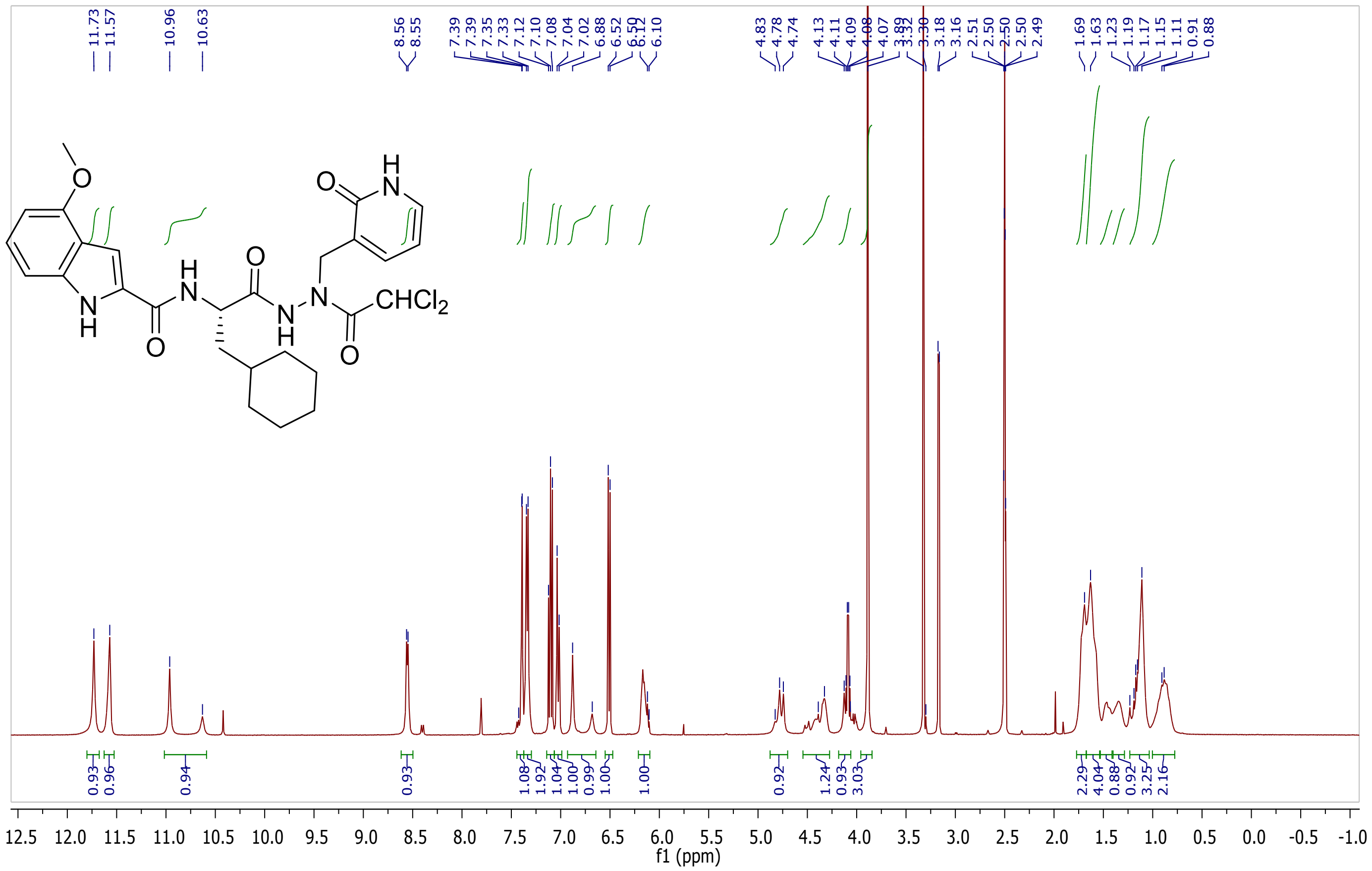

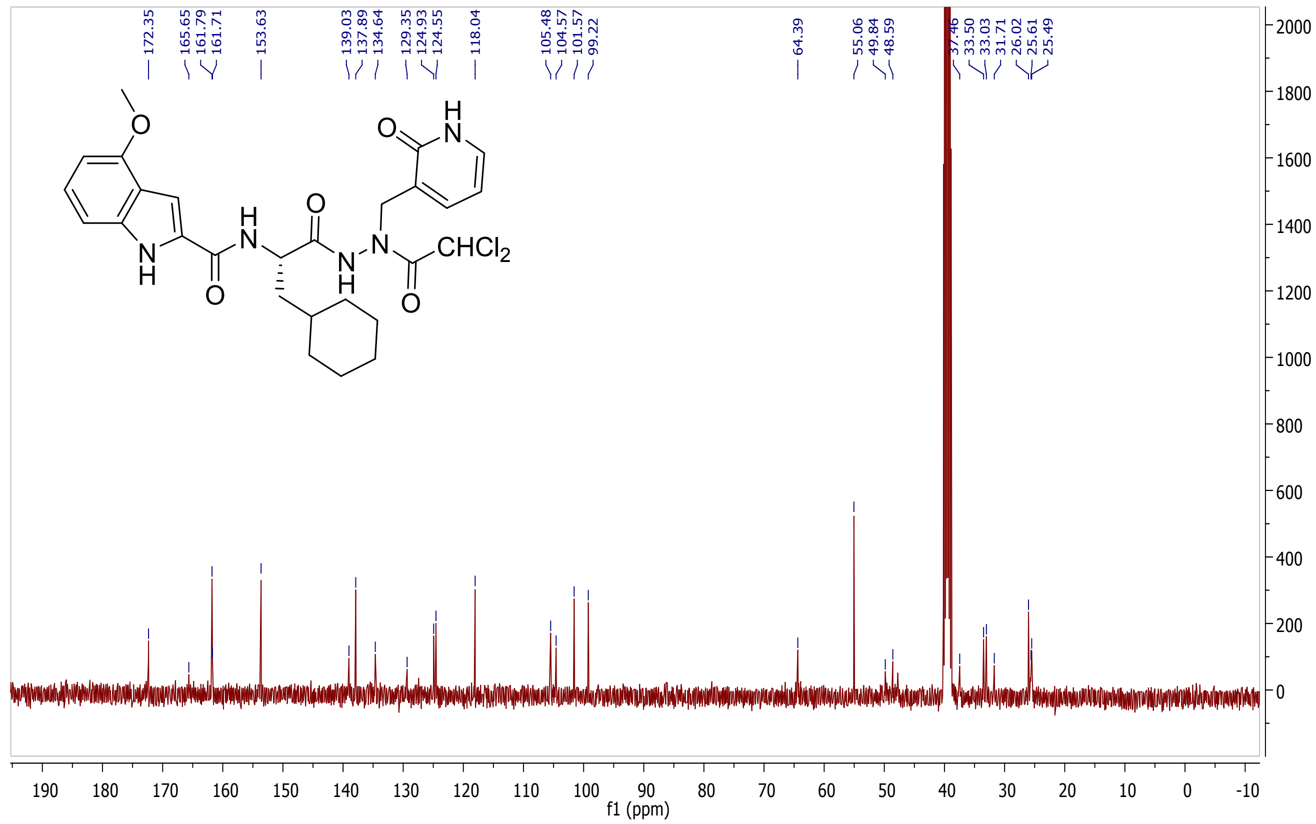

20i

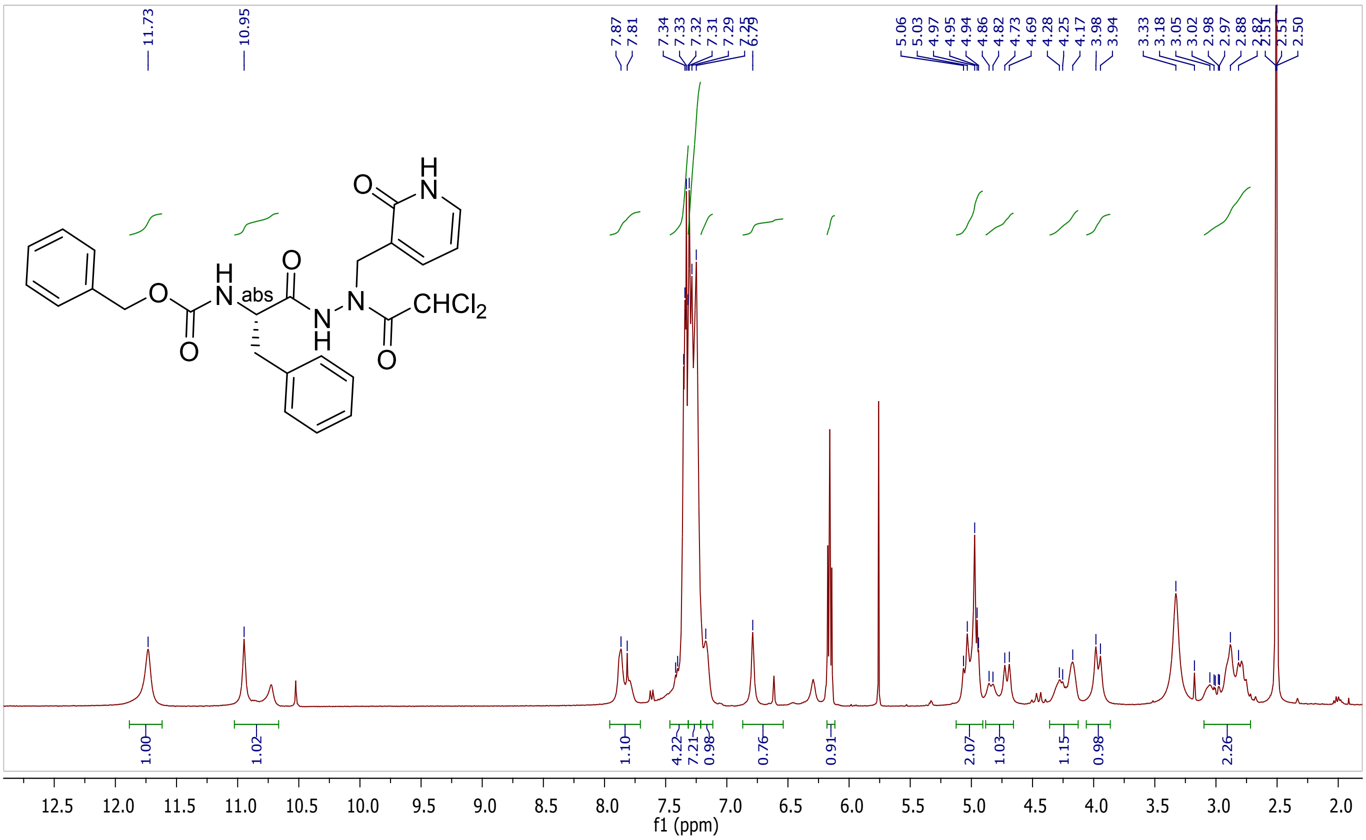
11.73
10.95
7.87
7.81
7.34
7.33
7.32
7.31
7.29
7.25
6.79
5.06
5.03
4.97
4.95
4.94
4.86
4.82
4.73
4.69
4.28
4.25
4.17
3.98
3.94
3.33
3.18
3.05
3.02
2.98
2.97
2.88
2.82
2.51
2.50
1.00
1.02
1.10
4.22
7.21
0.98
0.76
0.91
2.07
1.03
1.15
0.98
2.26
abs
CHCl2
f1 (ppm)

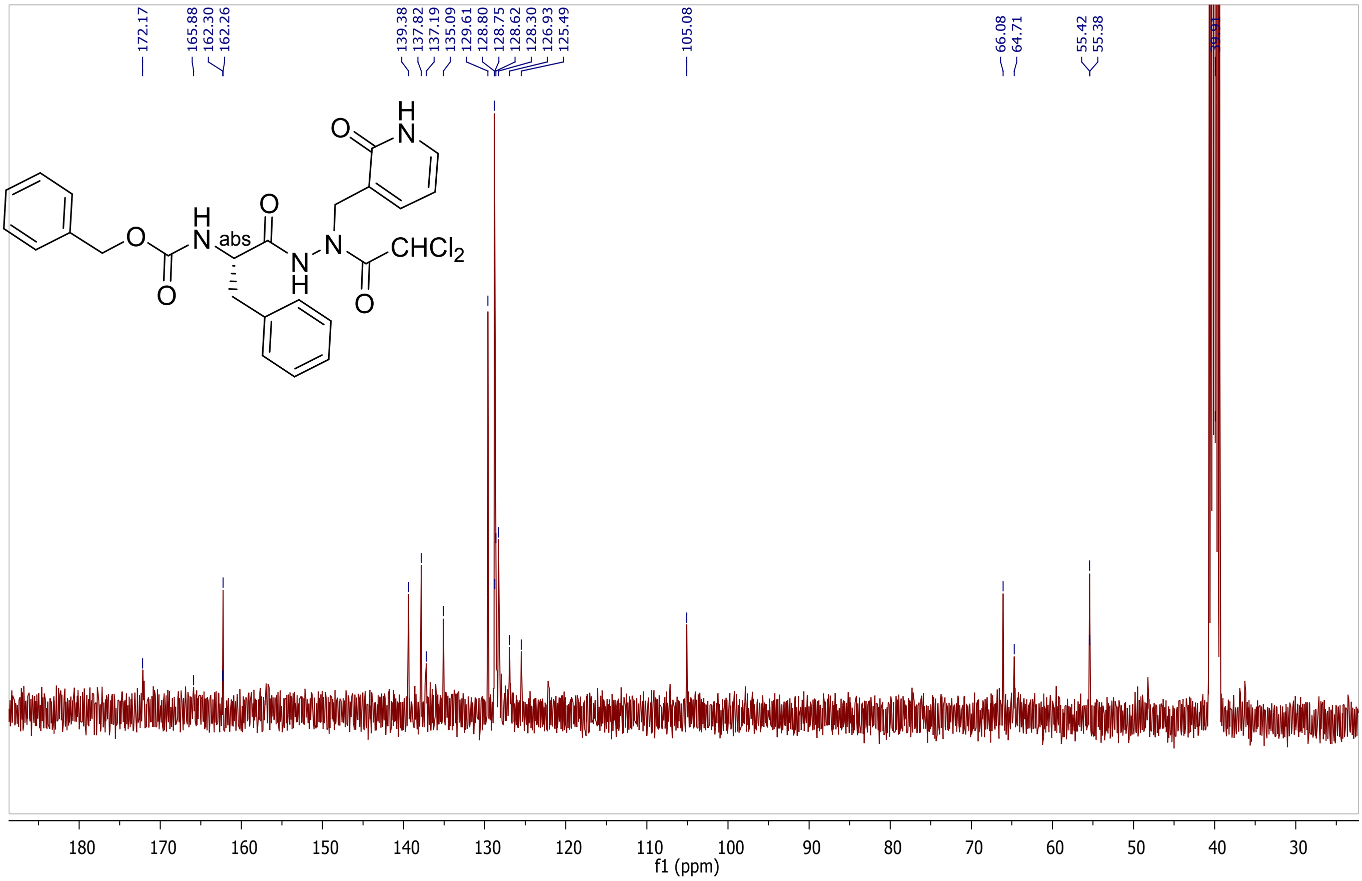
172.17
165.88
162.30
162.26
139.38
137.82
137.19
135.09
129.61
128.80
128.75
128.62
128.30
126.93
125.49
105.08
66.08
64.71
55.42
55.38
39.91
abs
CHCl2
f1 (ppm)

HPLC chart for selected compounds

5e

```
=====================================================================
Acq. Operator   : Michi                          Seq. Line :   3
Acq. Instrument : Instrument 1                    Location : Vial 3
Injection Date  : 04.11.2022 12:14:35                  Inj :   1
                                                Inj Volume : 5.000 µl
Acq. Method     : C:\CHEM32\1\DATA\22110401\22110201 2022-11-04 09-12-59\ZORBAX1.M
Last changed    : 28.09.2021 15:34:30 by Flo
Analysis Method : C:\PROGRAM FILES (X86)\CHEMSTATION\1\METHODS\DEF_LC.M
Additional Info : Peak(s) manually integrated
```

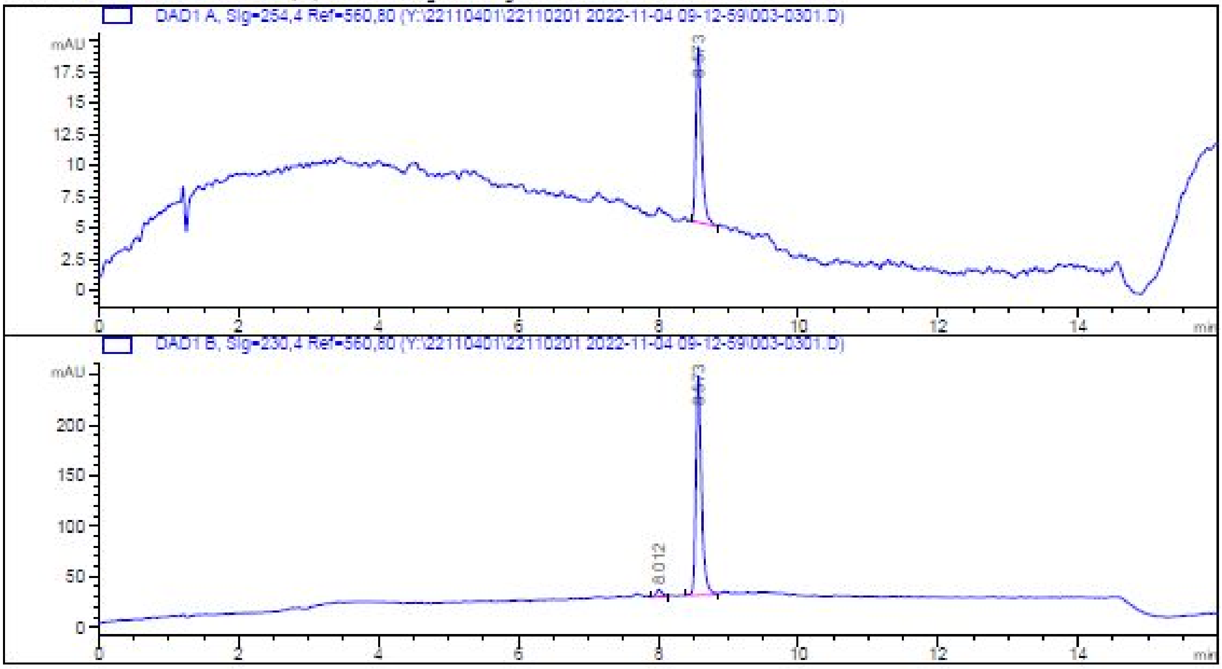


```
=====================================================================
                       Area Percent Report
=====================================================================
```

Sorted By : Signal
Multiplier: : 1.0000
Dilution: : 1.0000
Use Multiplier & Dilution Factor with ISTDs

Signal 1: DAD1 A, Sig=254,4 Ref=560,80

Peak #	RetTime [min]	Type	Width [min]	Area [mAU*s]	Height [mAU]	Area %
1	8.573	BB	0.0831	78.34969	14.04089	100.0000

Totals : 78.34969 14.04089

Signal 2: DAD1 B, Sig=230,4 Ref=560,80

Peak #	RetTime [min]	Type	Width [min]	Area [mAU*s]	Height [mAU]	Area %
1	8.012	BV	0.0847	42.23396	7.17429	3.3476
2	8.573	VV	0.0836	1219.38843	216.90369	96.6524

5f

```
=====================================================================
Acq. Operator   : Philipp                         Seq. Line :   2
Acq. Instrument : Instrument 1                     Location : Vial 2
Injection Date  : 16.11.2022 08:04:03                   Inj :   1
                                                 Inj Volume : 5.000 µl
Acq. Method     : C:\CHEM32\1\DATA\22111601\22111601 2022-11-16 07-45-53\ZORBAX1.M
Last changed    : 28.09.2021 15:34:30 by Flo
Analysis Method : C:\PROGRAM FILES (X86)\CHEMSTATION\1\METHODS\DEF_LC.M
Additional Info : Peak(s) manually integrated
```

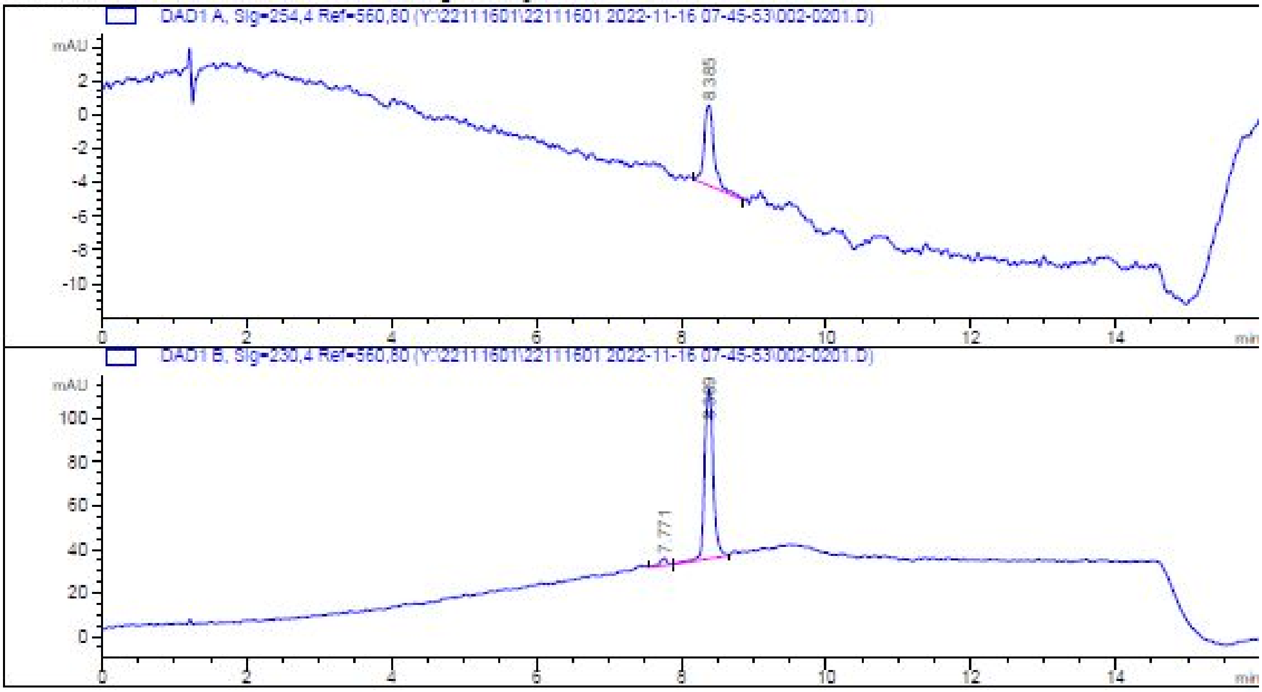


```
=====================================================================
                         Area Percent Report
=====================================================================

Sorted By             :      Signal
Multiplier:                   :      1.0000
Dilution:                     :      1.0000
Use Multiplier & Dilution Factor with ISTDs


Signal 1: DAD1 A, Sig=254,4 Ref=560,80

Peak RetTime Type  Width     Area      Height     Area
  #   [min]        [min]   [mAU*s]     [mAU]        %
----|-------|----|-------|----------|----------|--------|
   1   8.385 BB    0.1298   45.30830    4.72888 100.0000

Totals :                    45.30830    4.72888


Signal 2: DAD1 B, Sig=230,4 Ref=560,80

Peak RetTime Type  Width     Area      Height     Area
  #   [min]        [min]   [mAU*s]     [mAU]        %
----|-------|----|-------|----------|----------|--------|
   1   7.771 BB    0.1018   20.32908    3.21165   3.0897
   2   8.389 BV    0.1295  637.62390   77.89893  96.9103
```

5h

```
=====================================================================
Acq. Operator   : Rapha                           Seq. Line :   3
Acq. Instrument : Instrument 1                     Location : Vial 3
Injection Date  : 06.04.2023 09:50:34                   Inj :   1
                                                 Inj Volume : 5.000 µl
Acq. Method     : C:\CHEM32\1\DATA\23040601\23040601 2023-04-06 07-28-50\ZORBAX1.M
Last changed    : 28.09.2021 15:34:30 by Flo
Analysis Method : C:\PROGRAM FILES (X86)\CHEMSTATION\1\METHODS\DEF_LC.M
Additional Info : Peak(s) manually integrated
```

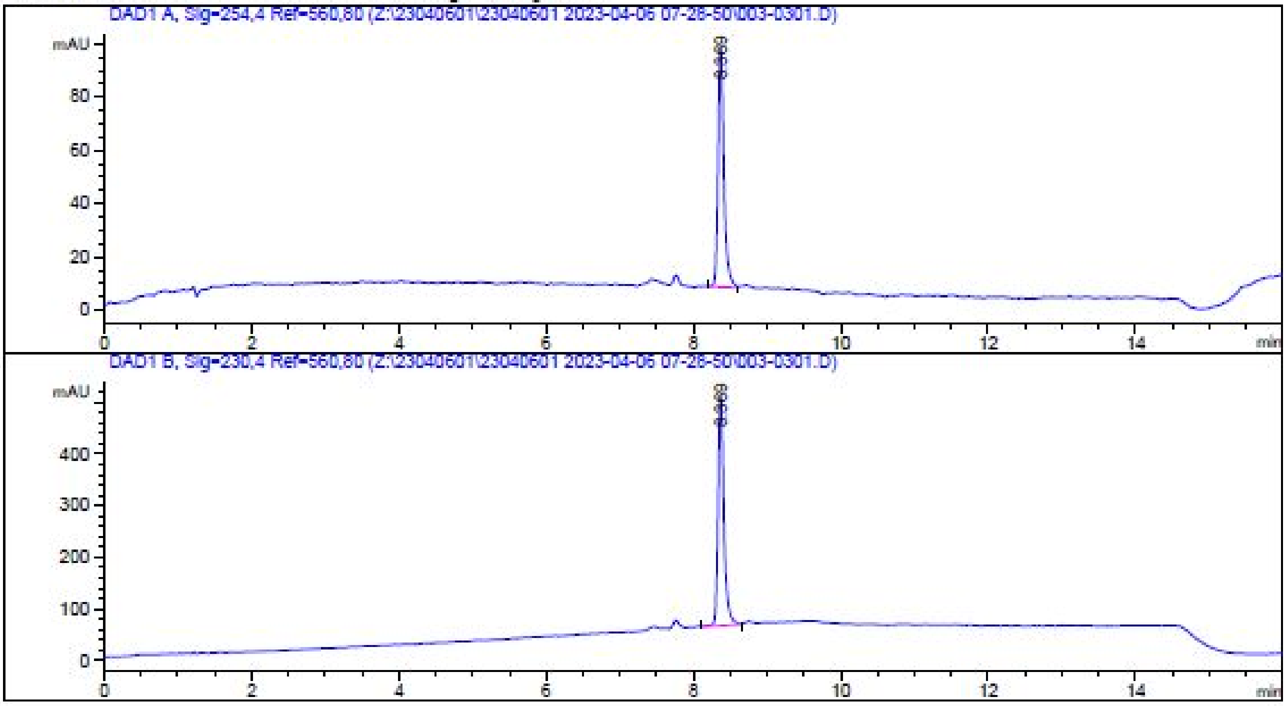


```
=====================================================================
                         Area Percent Report
=====================================================================

Sorted By             :      Signal
Multiplier:                   :      1.0000
Dilution:                     :      1.0000
Use Multiplier & Dilution Factor with ISTDs


Signal 1: DAD1 A, Sig=254,4 Ref=560,80

Peak RetTime Type  Width     Area      Height     Area
  #   [min]        [min]   [mAU*s]     [mAU]        %
----|-------|----|-------|----------|----------|--------|
   1   8.369 BV    0.0821  494.75598   89.98809 100.0000

Totals :                   494.75598   89.98809


Signal 2: DAD1 B, Sig=230,4 Ref=560,80

Peak RetTime Type  Width     Area      Height     Area
  #   [min]        [min]   [mAU*s]     [mAU]        %
----|-------|----|-------|----------|----------|--------|
   1   8.369 VV    0.0823 2462.55396  447.05502 100.0000
```

6a

```
==========================================================================
Acq. Operator   : Bene                              Seq. Line :  13
Acq. Instrument : Instrument 1                       Location : Vial 13
Injection Date  : 26.10.2022 14:07:27                     Inj :   1
                                                   Inj Volume : 5.000 µl
Acq. Method     : C:\CHEM32\1\DATA\22102601\22102601 2022-10-26 08-24-50\ZORBAX1.M
Last changed    : 28.09.2021 15:34:30 by Flo
Analysis Method : C:\PROGRAM FILES (X86)\CHEMSTATION\1\METHODS\DEF_LC.M
Additional Info : Peak(s) manually integrated
```

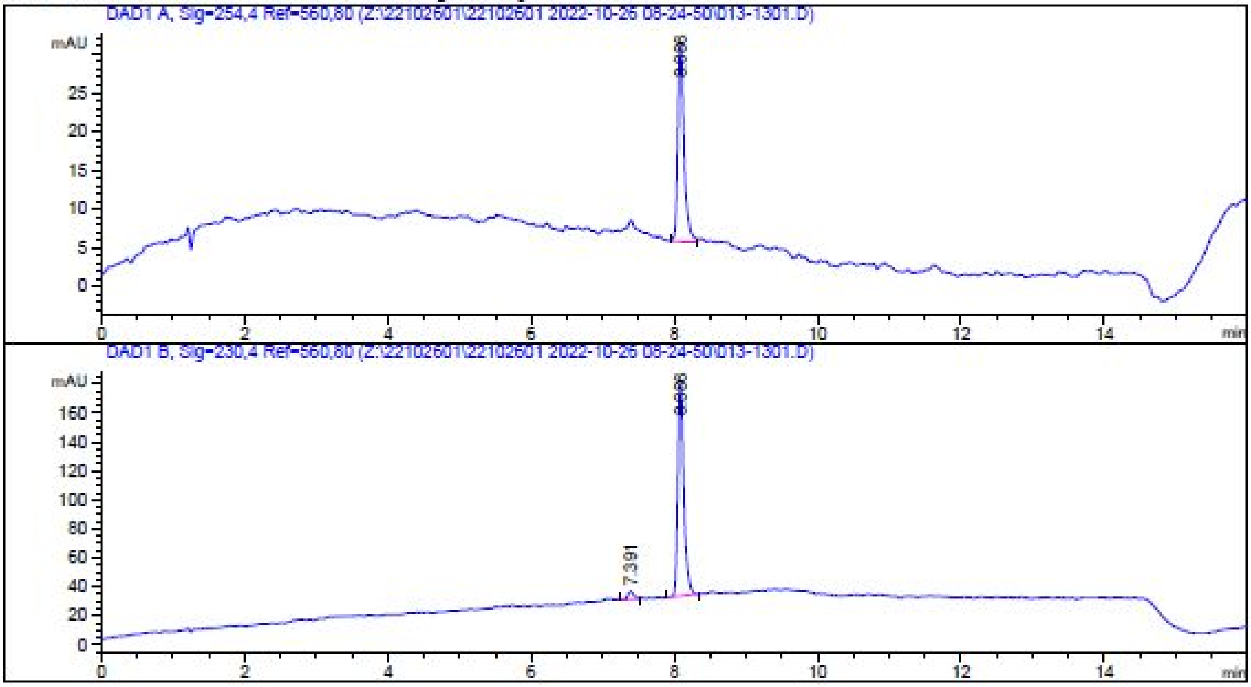


```
==========================================================================
                         Area Percent Report
==========================================================================

Sorted By             :      Signal
Multiplier:                  :      1.0000
Dilution:                    :      1.0000
Use Multiplier & Dilution Factor with ISTDs


Signal 1: DAD1 A, Sig=254,4 Ref=560,80

Peak RetTime Type  Width     Area      Height     Area
  #   [min]        [min]   [mAU*s]     [mAU]        %
----|-------|----|-------|----------|----------|--------|
   1   8.086 BV    0.0894  149.52585   25.13779 100.0000

Totals :                   149.52585   25.13779


Signal 2: DAD1 B, Sig=230,4 Ref=560,80

Peak RetTime Type  Width     Area      Height     Area
  #   [min]        [min]   [mAU*s]     [mAU]        %
----|-------|----|-------|----------|----------|--------|
   1   7.391 BV    0.0849   33.30994    5.80511   3.8594
   2   8.086 BB    0.0844  829.77991  145.86308  96.1406
```

6c

```
==================================================================
Acq. Operator   : Alex                          Seq. Line :   6
Acq. Instrument : Instrument 1                   Location : Vial 6
Injection Date  : 25.10.2022 15:32:13                 Inj :   1
                                               Inj Volume : 5.000 µl
Acq. Method     : C:\CHEM32\1\DATA\22102501\22102501 2022-10-25 09-39-53\ZORBAX1.M
Last changed    : 28.09.2021 15:34:30 by Flo
Analysis Method : C:\PROGRAM FILES (X86)\CHEMSTATION\1\METHODS\DEF_LC.M
Additional Info : Peak(s) manually integrated
```

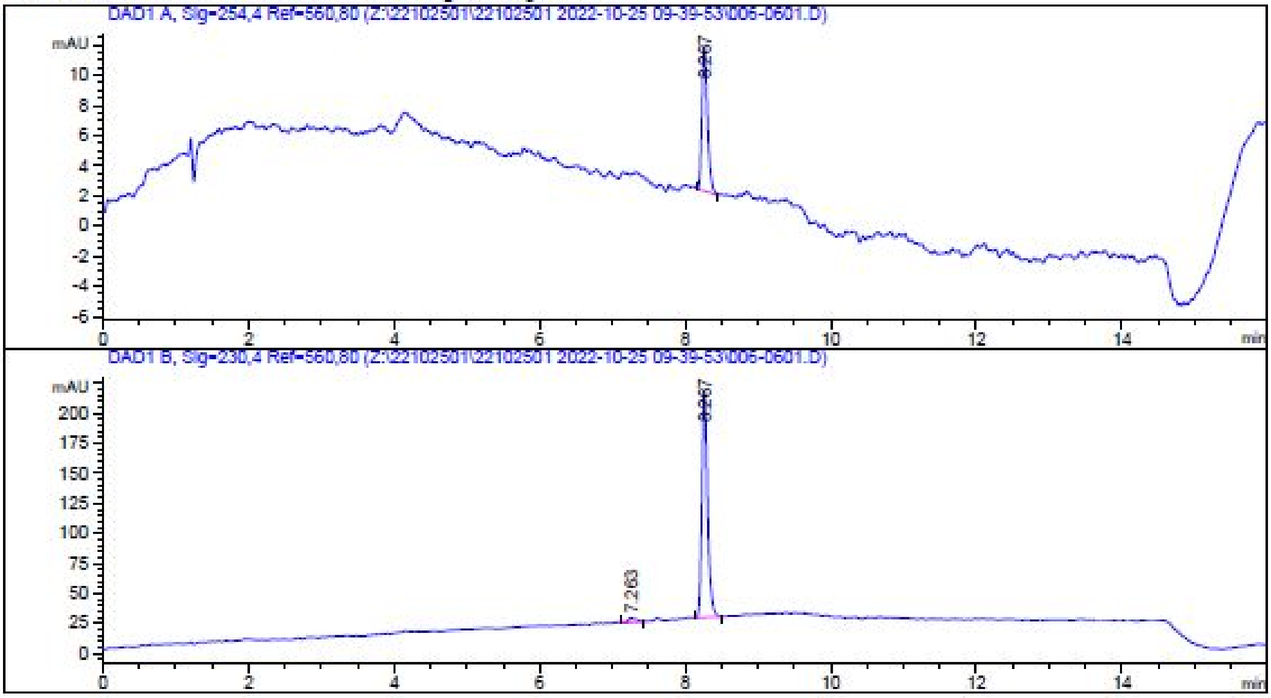


```
==================================================================
                        Area Percent Report
==================================================================

Sorted By               :      Signal
Multiplier:                     :       1.0000
Dilution:                       :       1.0000
Use Multiplier & Dilution Factor with ISTDs


Signal 1: DAD1 A, Sig=254,4 Ref=560,80

Peak RetTime Type  Width     Area      Height     Area
  #   [min]        [min]   [mAU*s]     [mAU]        %
----|-------|----|-------|----------|----------|--------|
   1   8.267 BB    0.0795   50.34161    9.54801 100.0000

Totals :                    50.34161    9.54801


Signal 2: DAD1 B, Sig=230,4 Ref=560,80

Peak RetTime Type  Width     Area      Height     Area
  #   [min]        [min]   [mAU*s]     [mAU]        %
----|-------|----|-------|----------|----------|--------|
   1   7.263 VV    0.1097   28.94133    3.60473   2.7536
   2   8.267 BV    0.0812 1022.08490  188.76826  97.2464
```

8a

```
=====================================================================
Acq. Operator   : Michi                          Seq. Line :   9
Acq. Instrument : Instrument 1                    Location : Vial 9
Injection Date  : 09.11.2022 12:14:34                  Inj :   1
                                                Inj Volume : 5.000 µl
Method          : C:\CHEM32\1\DATA\22110901\22110901 2022-11-09 08-19-37\ZORBAX1.M (Sequence
                  Method)
Last changed    : 27.06.2019 11:09:07 by Gregor
Additional Info : Peak(s) manually integrated
```

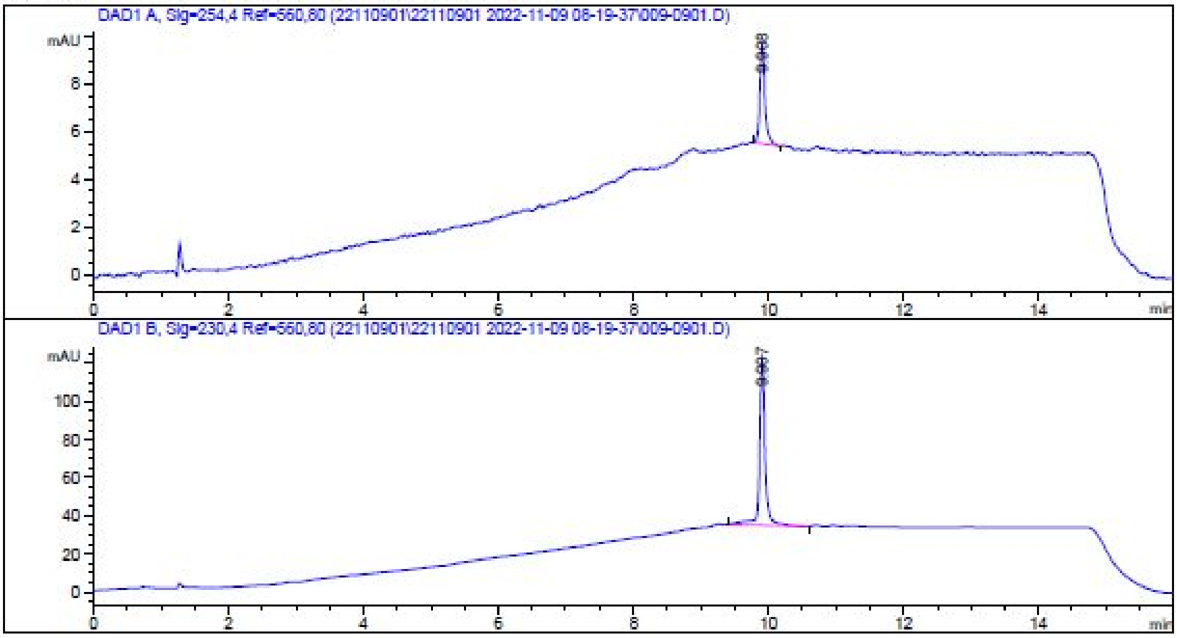

```
=====================================================================
                         Area Percent Report
=====================================================================

Sorted By             :      Signal
Multiplier:                   :      1.0000
Dilution:                     :      1.0000
Use Multiplier & Dilution Factor with ISTDs


Signal 1: DAD1 A, Sig=254,4 Ref=560,80

Peak RetTime Type  Width     Area      Height     Area
  #   [min]        [min]   [mAU*s]     [mAU]        %
----|-------|----|-------|----------|----------|--------|
   1   9.908 BB    0.0806   22.37797    4.17142 100.0000

Totals :                    22.37797    4.17142



Signal 2: DAD1 B, Sig=230,4 Ref=560,80

Peak RetTime Type  Width     Area      Height     Area
  #   [min]        [min]   [mAU*s]     [mAU]        %
----|-------|----|-------|----------|----------|--------|
   1   9.907 BB    0.0866  514.31213   87.40937 100.0000
```

8b

```
=====================================================================
Acq. Operator   : Eric                          Seq. Line :  11
Acq. Instrument : Instrument 1                   Location : Vial 11
Injection Date  : 10.01.2023 16:06:29                 Inj :   1
                                               Inj Volume : 5.000 µl
Acq. Method     : C:\CHEM32\1\DATA\23011001\23011001 2023-01-10 08-44-18\ZORBAX1.M
Last changed    : 28.09.2021 15:34:30 by Flo
Analysis Method : C:\PROGRAM FILES (X86)\CHEMSTATION\1\METHODS\DEF_LC.M
Additional Info : Peak(s) manually integrated
```

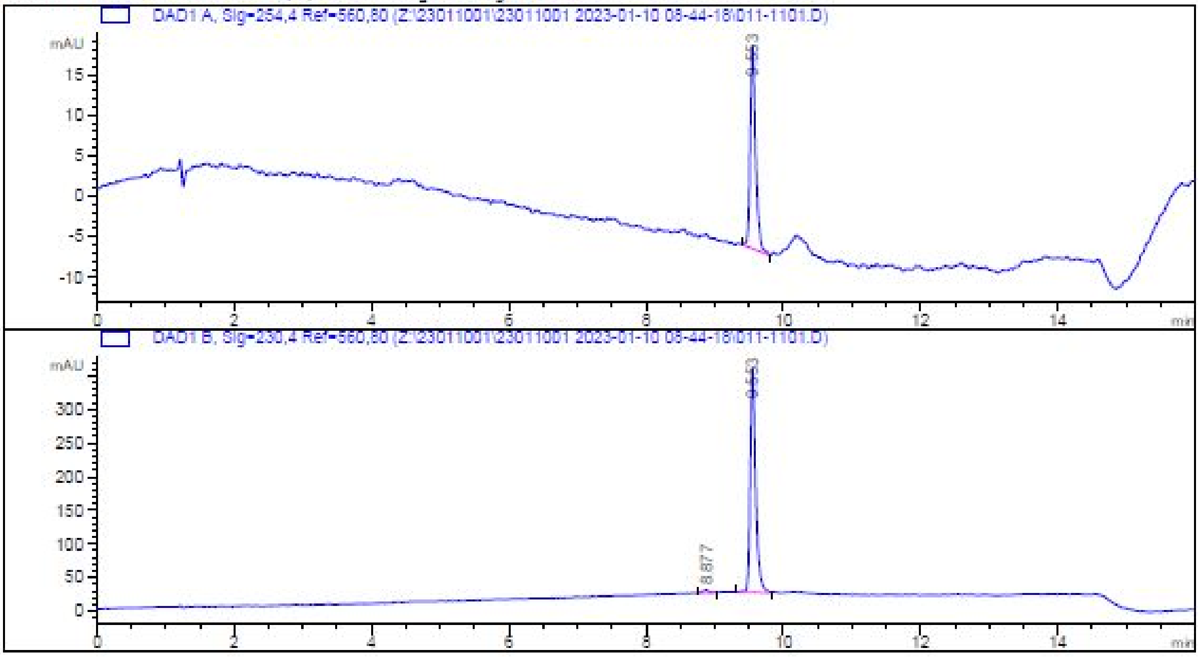


```
=====================================================================
                         Area Percent Report
=====================================================================

Sorted By             :      Signal
Multiplier:                    :      1.0000
Dilution:                      :      1.0000
Use Multiplier & Dilution Factor with ISTDs


Signal 1: DAD1 A, Sig=254,4 Ref=560,80

Peak RetTime Type  Width     Area       Height      Area
  #    [min]        [min]   [mAU*s]     [mAU]        %
----|-------|----|-------|----------|----------|--------|
   1   9.553 BB    0.0822  137.04338   24.91868 100.0000

Totals :                   137.04338   24.91868


Signal 2: DAD1 B, Sig=230,4 Ref=560,80

Peak RetTime Type  Width     Area       Height      Area
  #    [min]        [min]   [mAU*s]     [mAU]        %
----|-------|----|-------|----------|----------|--------|
   1   8.877 BB    0.0904   30.76879    4.58028   1.6296
   2   9.553 VV    0.0831 1857.38953  332.77261  98.3704
```

8c

```
=====================================================================
Acq. Operator   : Eric                           Seq. Line :   8
Acq. Instrument : Instrument 1                    Location : Vial 8
Injection Date  : 10.01.2023 15:00:07                  Inj :   1
                                               Inj Volume : 5.000 µl
Acq. Method     : C:\CHEM32\1\DATA\23011001\23011001 2023-01-10 08-44-18\ZORBAX1.M
Last changed    : 28.09.2021 15:34:30 by Flo
Analysis Method : C:\PROGRAM FILES (X86)\CHEMSTATION\1\METHODS\DEF_LC.M
Additional Info : Peak(s) manually integrated
```

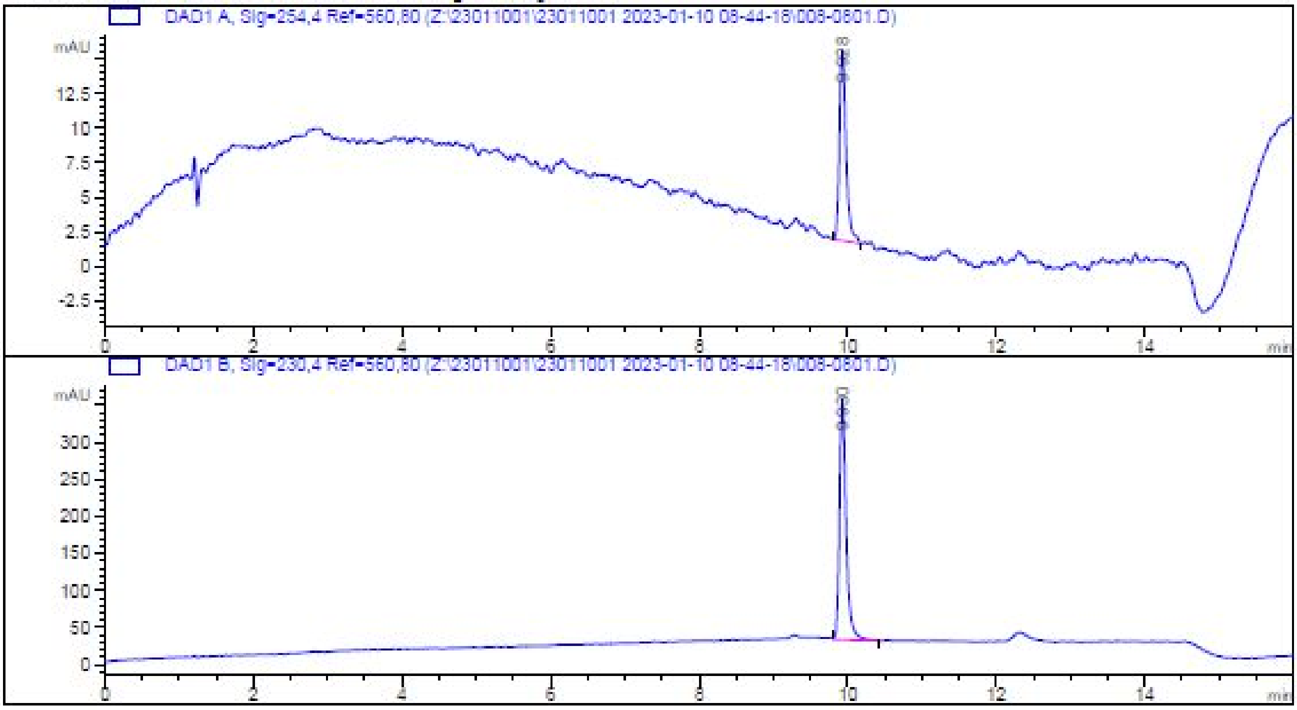


```
=====================================================================
                         Area Percent Report
=====================================================================

Sorted By             :      Signal
Multiplier:                  :      1.0000
Dilution:                    :      1.0000
Use Multiplier & Dilution Factor with ISTDs


Signal 1: DAD1 A, Sig=254,4 Ref=560,80

Peak RetTime Type  Width     Area      Height     Area
  #   [min]        [min]   [mAU*s]     [mAU]       %
----|-------|----|-------|----------|----------|--------|
   1   9.928 BB    0.0911   84.42631   13.85966 100.0000

Totals :                    84.42631   13.85966


Signal 2: DAD1 B, Sig=230,4 Ref=560,80

Peak RetTime Type  Width     Area      Height     Area
  #   [min]        [min]   [mAU*s]     [mAU]       %
----|-------|----|-------|----------|----------|--------|
   1   9.930 VB    0.0917 2002.55164  325.79822 100.0000
```

8f

```
=====================================================================
Acq. Operator   : Bene                            Seq. Line :   4
Acq. Instrument : Instrument 1                     Location : Vial 4
Injection Date  : 25.07.2023 15:31:42                   Inj :   1
                                                 Inj Volume : 5.000 µl
Acq. Method     : C:\CHEM32\1\DATA\23072501\23072501 2023-07-25 15-30-35\ZORBAX1.M
Last changed    : 28.09.2021 15:34:30 by Flo
Analysis Method : C:\PROGRAM FILES (X86)\CHEMSTATION\1\METHODS\DEF_LC.M
Additional Info : Peak(s) manually integrated
```

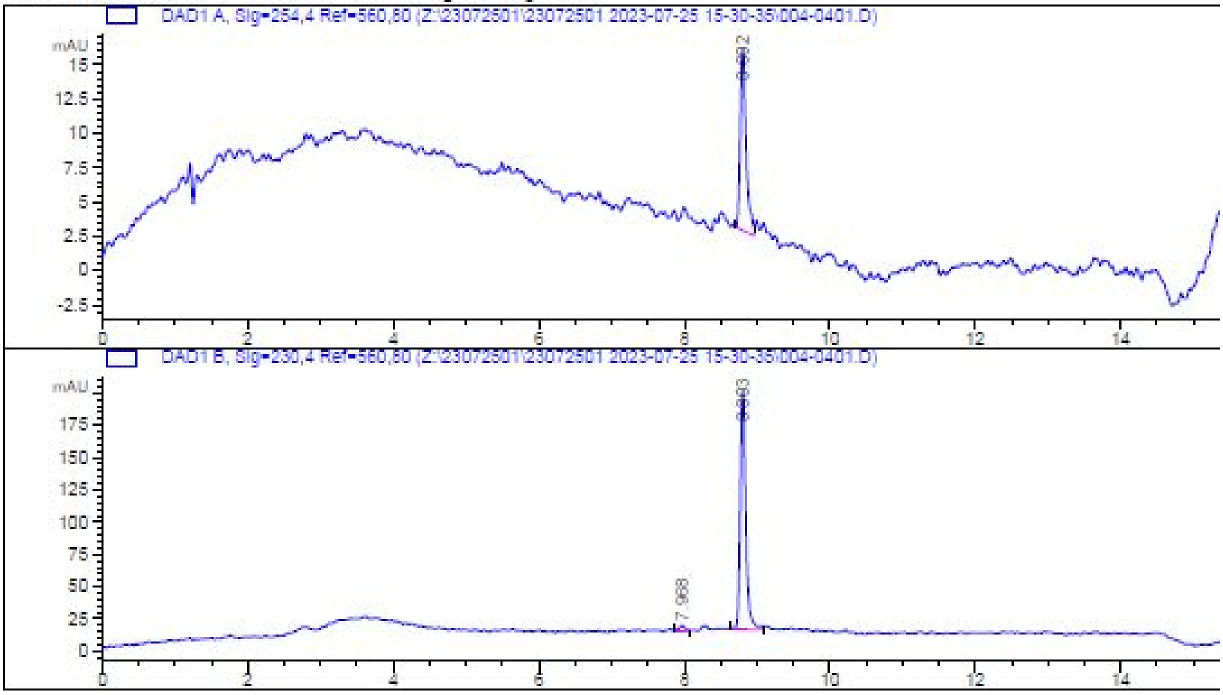


```
=====================================================================
                        Area Percent Report
=====================================================================

Sorted By             :      Signal
Multiplier:                  :      1.0000
Dilution:                    :      1.0000
Use Multiplier & Dilution Factor with ISTDs


Signal 1: DAD1 A, Sig=254,4 Ref=560,80

Peak RetTime Type  Width     Area      Height     Area
  #   [min]        [min]   [mAU*s]     [mAU]        %
----|-------|----|-------|----------|----------|--------|
   1   8.802 BV    0.0822   73.03151   13.26594 100.0000

Totals :                    73.03151   13.26594


Signal 2: DAD1 B, Sig=230,4 Ref=560,80

Peak RetTime Type  Width     Area      Height     Area
  #   [min]        [min]   [mAU*s]     [mAU]        %
----|-------|----|-------|----------|----------|--------|
   1   7.968 VV    0.0925   28.56111    4.35622   2.8449
   2   8.803 BV    0.0793  975.37323  185.54117  97.1551
```

8g

```
=======================================================================
Acq. Operator   : Bene                              Seq. Line :  11
Acq. Instrument : Instrument 1                       Location : Vial 11
Injection Date  : 13.01.2023 13:58:56                     Inj :   1
                                                  Inj Volume : 5.000 µl
Acq. Method     : C:\CHEM32\1\DATA\23011301\23011301 2023-01-13 07-52-54\ZORBAX1.M
Last changed    : 27.06.2019 11:09:07 by Gregor
Analysis Method : C:\PROGRAM FILES (X86)\CHEMSTATION\1\METHODS\DEF_LC.M
Last changed    : 13.01.2023 14:16:37
                  (modified after loading)
Additional Info : Peak(s) manually integrated
```

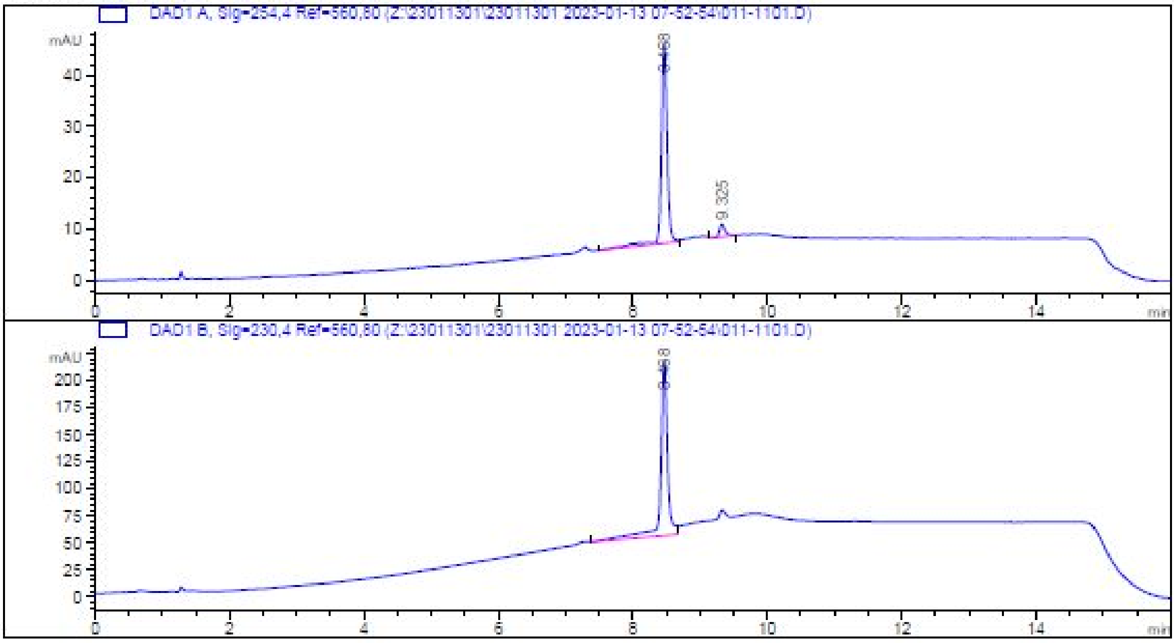


```
=======================================================================
                          Area Percent Report
=======================================================================

Sorted By             :      Signal
Multiplier:                  :      1.0000
Dilution:                    :      1.0000
Use Multiplier & Dilution Factor with ISTDs


Signal 1: DAD1 A, Sig=254,4 Ref=560,80

Peak RetTime Type  Width     Area      Height     Area
  #   [min]        [min]   [mAU*s]     [mAU]       %
----|-------|----|-------|----------|----------|--------|
   1   8.468 BV    0.0931  236.08357   38.71935  94.0713
   2   9.325 VB    0.0913   14.87883    2.43527   5.9287


Signal 2: DAD1 B, Sig=230,4 Ref=560,80

Peak RetTime Type  Width     Area      Height     Area
  #   [min]        [min]   [mAU*s]     [mAU]       %
----|-------|----|-------|----------|----------|--------|
   1   8.468 BV    0.1050 1157.63708  162.69322 100.0000

Totals :                  1157.63708  162.69322
```

8h

```
===================================================================
Acq. Operator   : Bene                          Seq. Line :   6
Acq. Instrument : Instrument 1                   Location : Vial 6
Injection Date  : 13.01.2023 10:45:34                 Inj :   1
                                               Inj Volume : 5.000 µl
Acq. Method     : C:\CHEM32\1\DATA\23011301\23011301 2023-01-13 07-52-54\ZORBAX1.M
Last changed    : 27.06.2019 11:09:07 by Gregor
Analysis Method : C:\PROGRAM FILES (X86)\CHEMSTATION\1\METHODS\DEF_LC.M
Additional Info : Peak(s) manually integrated
```

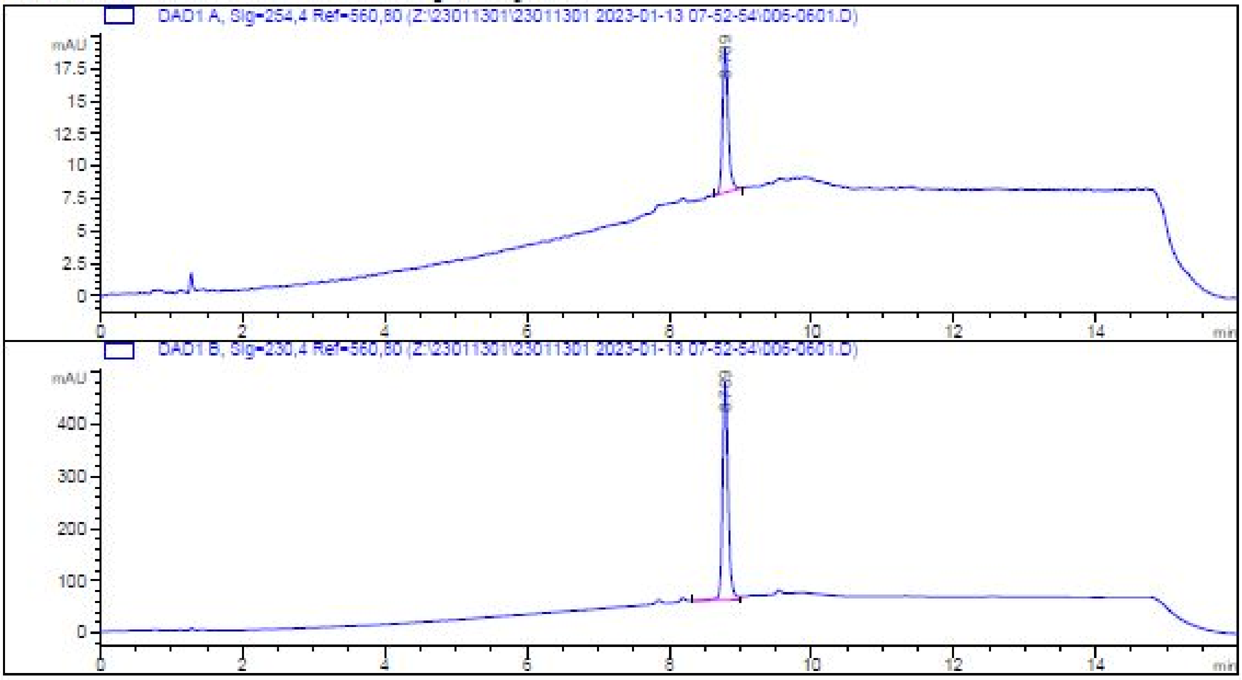


```
===================================================================
                       Area Percent Report
===================================================================

Sorted By             :      Signal
Multiplier:                   :      1.0000
Dilution:                     :      1.0000
Use Multiplier & Dilution Factor with ISTDs


Signal 1: DAD1 A, Sig=254,4 Ref=560,80

Peak RetTime Type  Width     Area      Height     Area
  #   [min]        [min]   [mAU*s]     [mAU]       %
----|-------|----|-------|----------|----------|--------|
   1   8.789 BB    0.0808   60.23560   11.18937 100.0000

Totals :                    60.23560   11.18937


Signal 2: DAD1 B, Sig=230,4 Ref=560,80

Peak RetTime Type  Width     Area      Height     Area
  #   [min]        [min]   [mAU*s]     [mAU]       %
----|-------|----|-------|----------|----------|--------|
   1   8.789 BV    0.0810 2268.62646  419.80127 100.0000
```

8i

```
=====================================================================
Acq. Operator   : Bene                              Seq. Line :    4
Acq. Instrument : Instrument 1                       Location : Vial 4
Injection Date  : 28.07.2023 15:30:57                     Inj :    1
                                                   Inj Volume : 5.000 µl
Acq. Method     : C:\CHEM32\1\DATA\23072801\23072801 2023-07-28 15-29-53\ZORBAX1.M
Last changed    : 28.09.2021 15:34:30 by Flo
Analysis Method : C:\PROGRAM FILES (X86)\CHEMSTATION\1\METHODS\DEF_LC.M
Additional Info : Peak(s) manually integrated
```

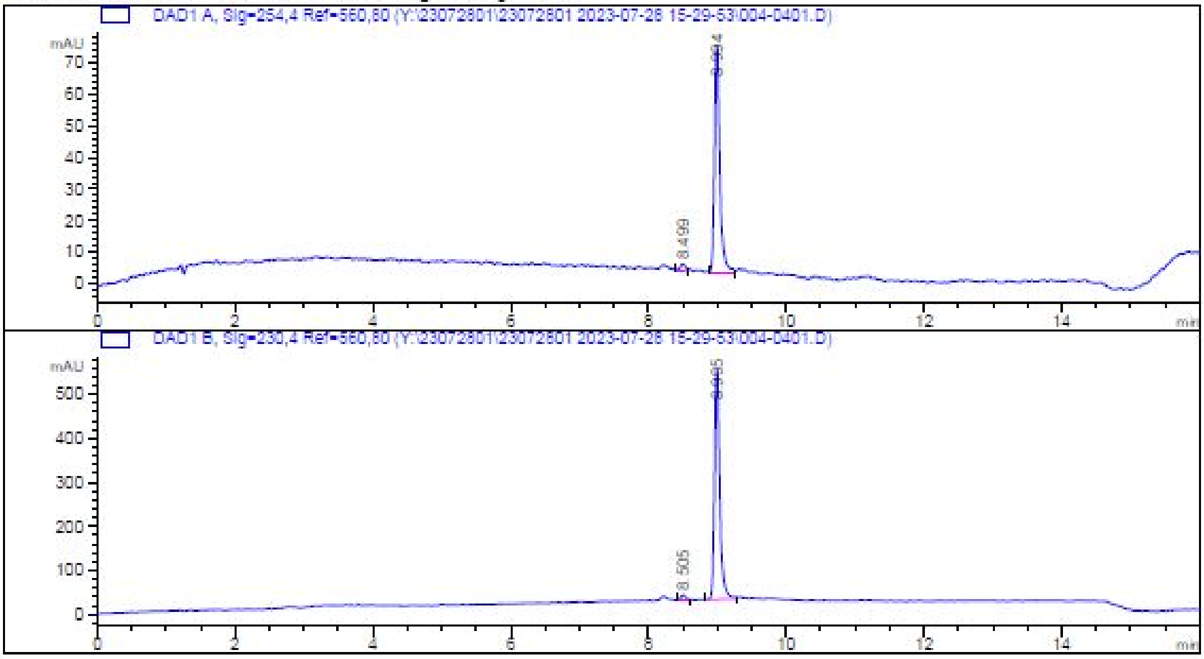


```
=====================================================================
                         Area Percent Report
=====================================================================

Sorted By             :      Signal
Multiplier:                  :      1.0000
Dilution:                    :      1.0000
Use Multiplier & Dilution Factor with ISTDs


Signal 1: DAD1 A, Sig=254,4 Ref=560,80
```

Peak #	RetTime [min]	Type	Width [min]	Area [mAU*s]	Height [mAU]	Area %
1	8.499	VV	0.0861	12.52975	2.21079	3.1993
2	8.994	BV	0.0796	379.10846	71.84521	96.8007

Peak #	RetTime [min]	Type	Width [min]	Area [mAU*s]	Height [mAU]	Area %
1	8.505	BV	0.0754	57.96172	11.77777	2.0645
2	8.995	BV	0.0795	2749.63330	521.59949	97.9355

8j

```
==================================================================
Acq. Operator   : Moritz                        Seq. Line :   5
Acq. Instrument : Instrument 1                   Location : Vial 5
Injection Date  : 10.07.2023 13:10:23                 Inj :   1
                                               Inj Volume : 5.000 µl
Acq. Method     : C:\CHEM32\1\DATA\23071001\23071001 2023-07-10 12-34-39\ZORBAX1.M
Last changed    : 28.09.2021 15:34:30 by Flo
Analysis Method : C:\PROGRAM FILES (X86)\CHEMSTATION\1\METHODS\DEF_LC.M
Additional Info : Peak(s) manually integrated
```

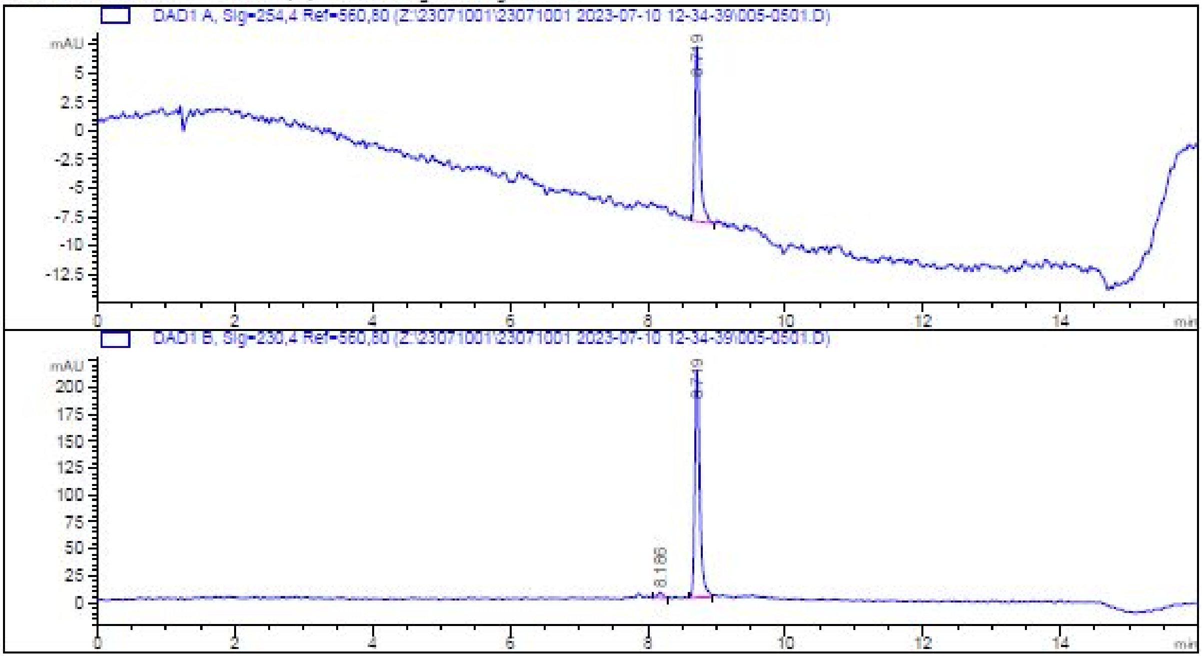


```
==================================================================
                        Area Percent Report
==================================================================

Sorted By             :      Signal
Multiplier:                   :      1.0000
Dilution:                     :      1.0000
Use Multiplier & Dilution Factor with ISTDs


Signal 1: DAD1 A, Sig=254,4 Ref=560,80

Peak RetTime Type  Width     Area      Height     Area
  #   [min]        [min]   [mAU*s]     [mAU]        %
----|-------|----|-------|----------|----------|--------|
   1   8.719 BB    0.0757   78.14903   15.27063 100.0000

Totals :                    78.14903   15.27063


Signal 2: DAD1 B, Sig=230,4 Ref=560,80

Peak RetTime Type  Width     Area      Height     Area
  #   [min]        [min]   [mAU*s]     [mAU]        %
----|-------|----|-------|----------|----------|--------|
   1   8.186 BV    0.0816   29.98278    5.33170   2.7285
   2   8.719 BV    0.0769 1068.88904  211.57648  97.2715
```

8k

===

Acq. Operator : Rapha   Seq. Line : 7
Acq. Instrument : Instrument 1   Location : Vial 7
Injection Date : 30.03.2023 13:05:45   Inj : 1
                                                          Inj Volume : 5.000 µl
Acq. Method : C:\CHEM32\1\DATA\23033001\23033001 2023-03-30 07-07-17\ZORBAX1.M
Last changed : 28.09.2021 15:34:30 by Flo
Analysis Method : C:\PROGRAM FILES (X86)\CHEMSTATION\1\METHODS\DEF_LC.M
Additional Info : Peak(s) manually integrated

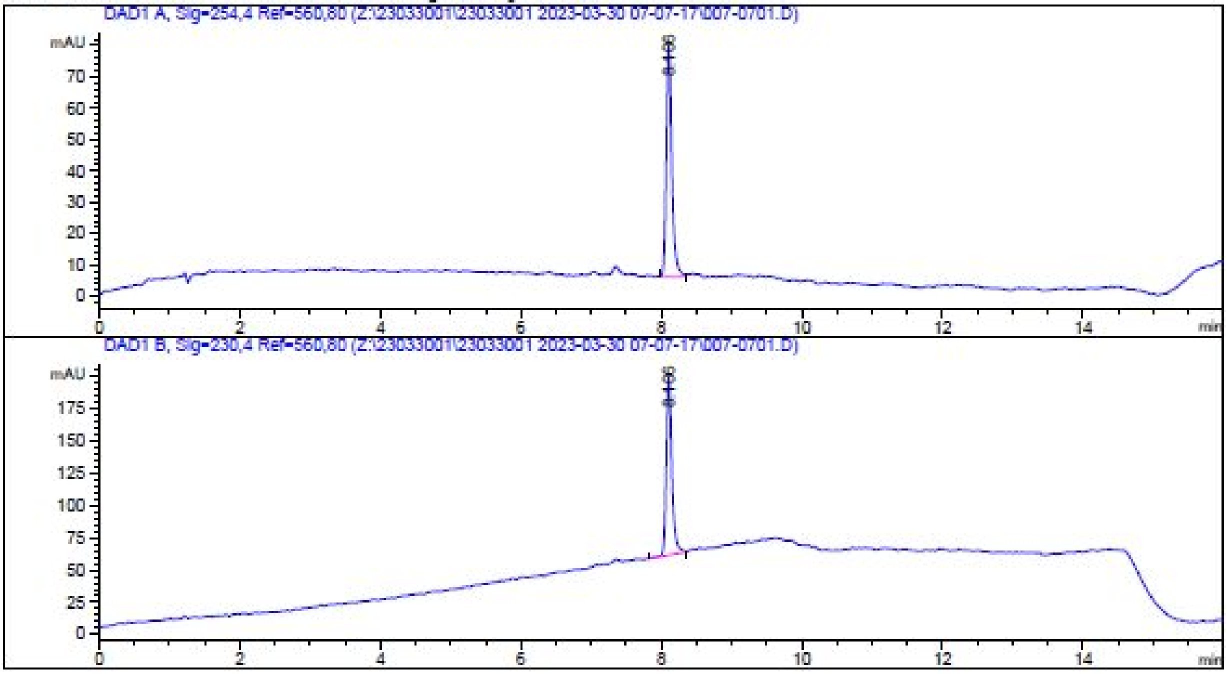

===

Area Percent Report

===

Sorted By : Signal
Multiplier: : 1.0000
Dilution: : 1.0000
Use Multiplier & Dilution Factor with ISTDs

Signal 1: DAD1 A, Sig=254,4 Ref=560,80

Peak #	RetTime [min]	Type	Width [min]	Area [mAU*s]	Height [mAU]	Area %
1	8.106	VV	0.0846	408.93933	73.81310	100.0000

Totals : 408.93933 73.81310

Signal 2: DAD1 B, Sig=230,4 Ref=560,80

Peak #	RetTime [min]	Type	Width [min]	Area [mAU*s]	Height [mAU]	Area %
1	8.106	BB	0.0848	765.74042	137.80475	100.0000

14a

```
======================================================================
Acq. Operator   : Pascal                              Seq. Line :   9
Acq. Instrument : Instrument 1                         Location : Vial 9
Injection Date  : 07.12.2023 12:04:12                       Inj :   1
                                                     Inj Volume : 5.000 µl
Acq. Method     : C:\CHEM32\1\DATA\23120701\23120701 2023-12-07 08-41-00\ZORBAX1.M
Last changed    : 27.06.2019 11:09:07 by Gregor
Analysis Method : C:\PROGRAM FILES (X86)\CHEMSTATION\1\METHODS\DEF_LC.M
Sample Info     : undried material

Additional Info : Peak(s) manually integrated
```

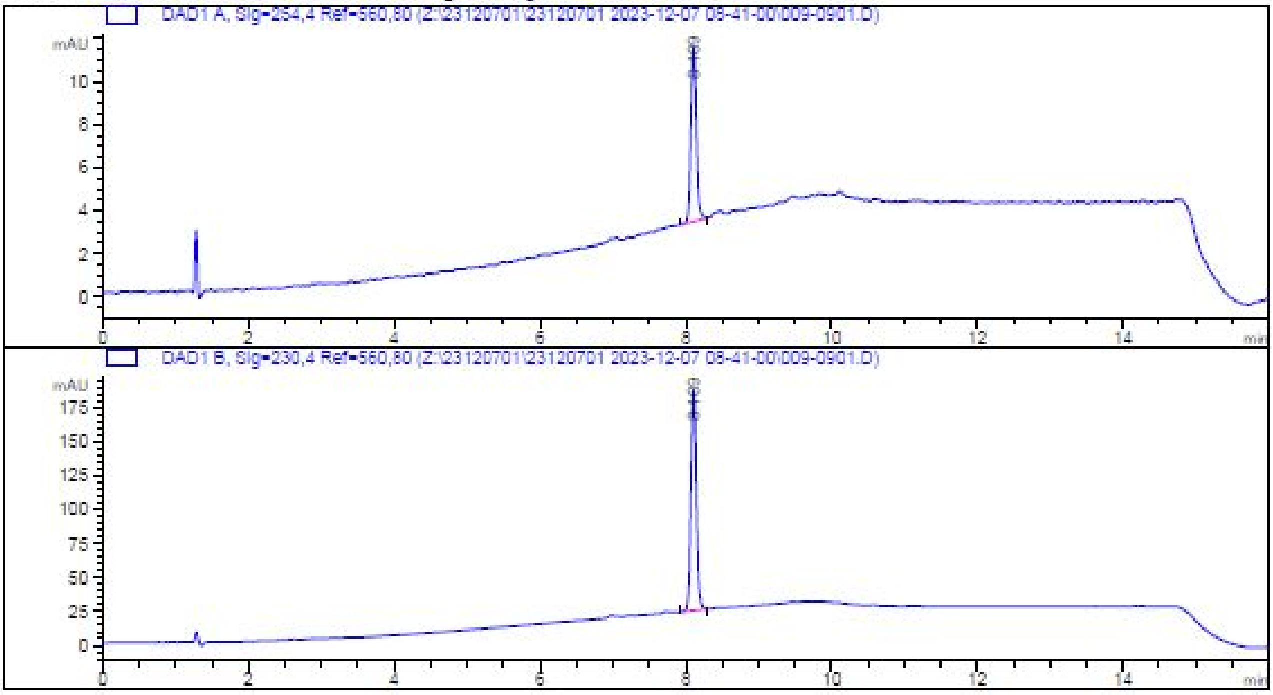


```
======================================================================
                         Area Percent Report
======================================================================

Sorted By             :      Signal
Multiplier:                   :      1.0000
Dilution:                     :      1.0000
Use Multiplier & Dilution Factor with ISTDs


Signal 1: DAD1 A, Sig=254,4 Ref=560,80

Peak RetTime Type  Width     Area      Height     Area
  #   [min]        [min]   [mAU*s]     [mAU]        %
----|-------|----|-------|----------|----------|--------|
   1   8.109 BB    0.0798   41.53514    8.10843 100.0000


Signal 2: DAD1 B, Sig=230,4 Ref=560,80

Peak RetTime Type  Width     Area      Height     Area
  #   [min]        [min]   [mAU*s]     [mAU]        %
----|-------|----|-------|----------|----------|--------|
   1   8.109 BB    0.0769  823.26501  162.94606 100.0000
```

14b

```
=====================================================================
Acq. Operator   : Pascal                          Seq. Line :   4
Acq. Instrument : Instrument 1                     Location : Vial 4
Injection Date  : 07.12.2023 11:40:18                   Inj :   1
                                                Inj Volume : 5.000 µl
Acq. Method     : C:\CHEM32\1\DATA\23120701\23120701 2023-12-07 11-39-14\ZORBAX1.M
Last changed    : 28.09.2021 15:34:30 by Flo
Analysis Method : C:\PROGRAM FILES (X86)\CHEMSTATION\1\METHODS\DEF_LC.M
Additional Info : Peak(s) manually integrated
```

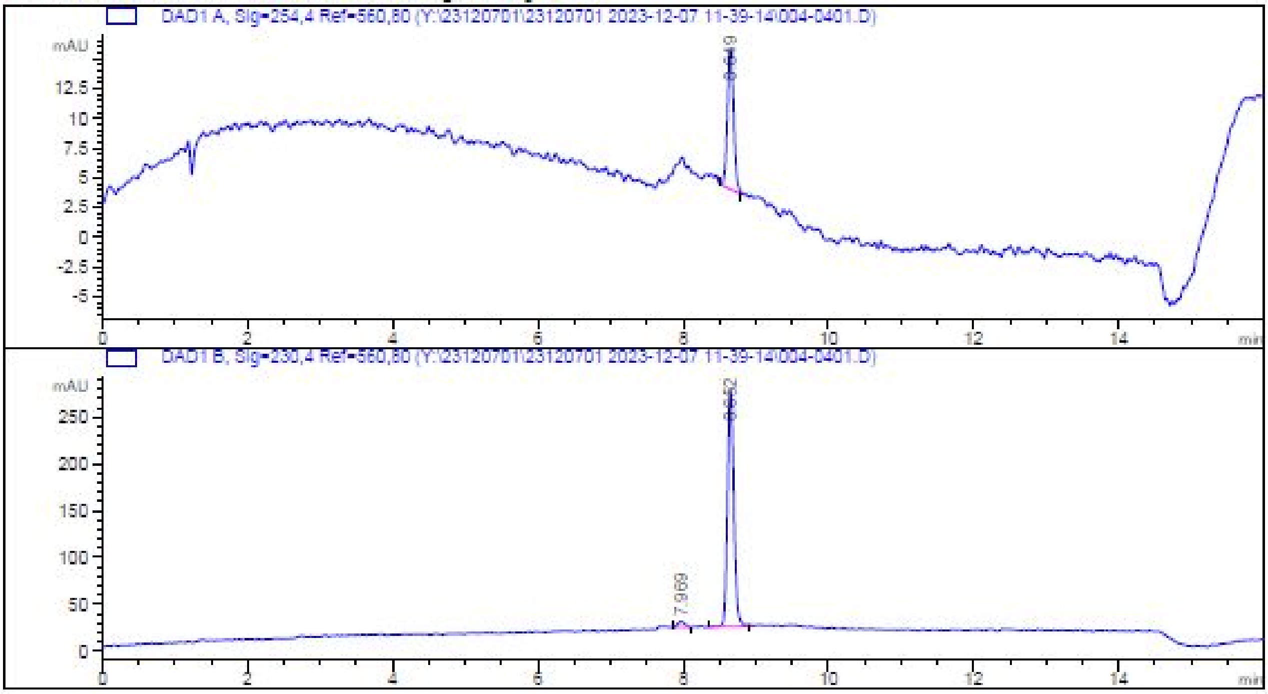


```
=====================================================================
                         Area Percent Report
=====================================================================

Sorted By             :      Signal
Multiplier:                   :      1.0000
Dilution:                     :      1.0000
Use Multiplier & Dilution Factor with ISTDs


Signal 1: DAD1 A, Sig=254,4 Ref=560,80

Peak RetTime Type  Width     Area      Height     Area
  #   [min]        [min]   [mAU*s]     [mAU]        %
----|-------|----|-------|----------|----------|--------|
   1   8.649 BB    0.0961   71.12656   11.83409 100.0000

Totals :                    71.12656   11.83409


Signal 2: DAD1 B, Sig=230,4 Ref=560,80

Peak RetTime Type  Width     Area      Height     Area
  #   [min]        [min]   [mAU*s]     [mAU]        %
----|-------|----|-------|----------|----------|--------|
   1   7.969 BB    0.1023   42.66342    6.04954   2.6758
   2   8.652 BV    0.0957 1551.75769  252.38835  97.3242
```

14c

```
=====================================================================
Acq. Operator   : Reiner                          Seq. Line :   5
Acq. Instrument : Instrument 1                     Location : Vial 5
Injection Date  : 20.04.2023 14:17:22                   Inj :   1
                                                 Inj Volume : 5.000 µl
Acq. Method     : C:\CHEM32\1\DATA\23042001\23042001 2023-04-20 10-53-14\ZORBAX1.M
Last changed    : 28.09.2021 15:34:30 by Flo
Analysis Method : C:\PROGRAM FILES (X86)\CHEMSTATION\1\METHODS\DEF_LC.M
Additional Info : Peak(s) manually integrated
```

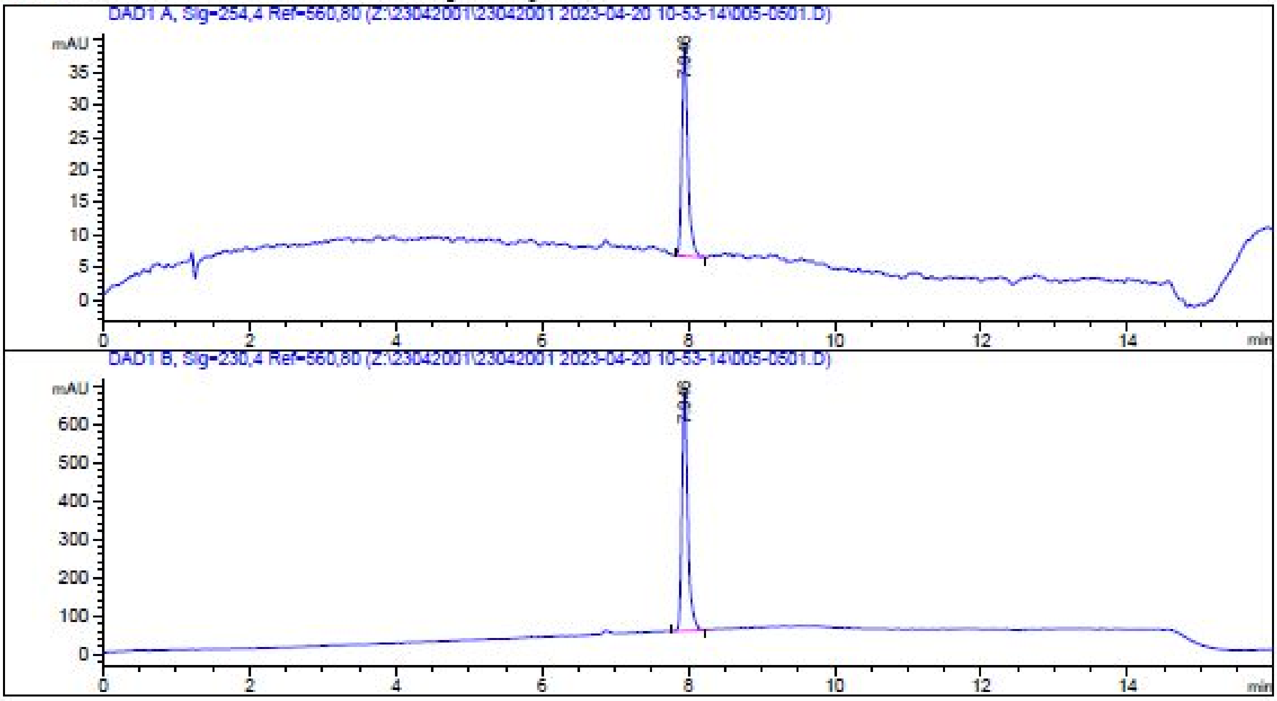


```
=====================================================================
                         Area Percent Report
=====================================================================

Sorted By             :      Signal
Multiplier:                   :       1.0000
Dilution:                     :       1.0000
Use Multiplier & Dilution Factor with ISTDs


Signal 1: DAD1 A, Sig=254,4 Ref=560,80

Peak RetTime Type  Width     Area      Height     Area
  #   [min]        [min]   [mAU*s]     [mAU]       %
----|-------|----|-------|----------|----------|--------|
   1   7.946 BB    0.0837  180.86108   32.13832 100.0000

Totals :                   180.86108   32.13832


Signal 2: DAD1 B, Sig=230,4 Ref=560,80

Peak RetTime Type  Width     Area      Height     Area
  #   [min]        [min]   [mAU*s]     [mAU]       %
----|-------|----|-------|----------|----------|--------|
   1   7.946 BB    0.0827 3438.62500  620.31268 100.0000
```

14d

```
=====================================================================
Acq. Operator   : Alex                              Seq. Line :   9
Acq. Instrument : Instrument 1                       Location : Vial 9
Injection Date  : 17.03.2023 14:38:06                     Inj :   1
                                                   Inj Volume : 5.000 µl
Different Inj Volume from Sequence !     Actual Inj Volume : 20.000 µl
Acq. Method     : C:\CHEM32\1\DATA\23031701\23031701 2023-03-17 09-18-32\ZORBAX1.M
Last changed    : 27.06.2019 11:09:07 by Gregor
Analysis Method : C:\CHEM32\1\METHODS\ELLEN1.M
Last changed    : 14.03.2023 16:34:29 by Moritz
                  (modified after loading)
Additional Info : Peak(s) manually integrated
```

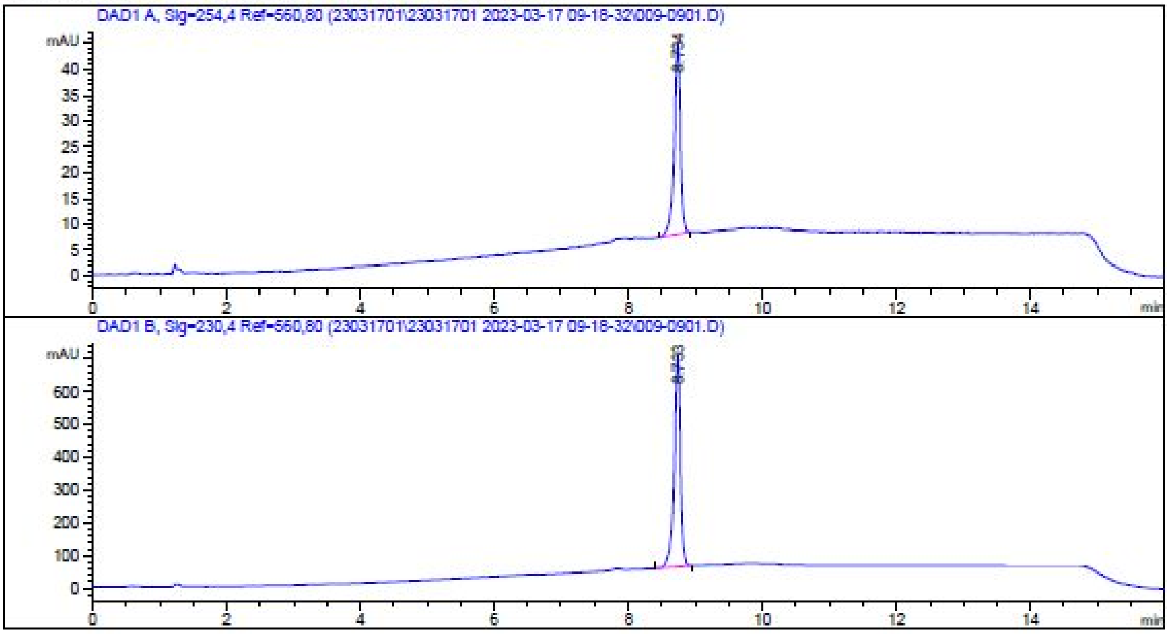


```
=====================================================================
                         Area Percent Report
=====================================================================

Sorted By             :      Signal
Multiplier:                  :      1.0000
Dilution:                    :      1.0000
Use Multiplier & Dilution Factor with ISTDs


Signal 1: DAD1 A, Sig=254,4 Ref=560,80

Peak RetTime Type  Width     Area      Height     Area
  #   [min]        [min]   [mAU*s]     [mAU]       %
----|-------|----|-------|----------|----------|--------|
   1   8.734 BB    0.0933  230.98932   36.78432 100.0000


Signal 2: DAD1 B, Sig=230,4 Ref=560,80

Peak RetTime Type  Width     Area      Height     Area
  #   [min]        [min]   [mAU*s]     [mAU]       %
----|-------|----|-------|----------|----------|--------|
   1   8.733 BB    0.0954 4044.46436  642.82727 100.0000
```

14e

Acq. Operator : Bene
Seq. Line : 14
Acq. Instrument : Instrument 1
Location : Vial 14
Injection Date : 06.12.2023 16:10:09
Inj : 1
Inj Volume : 5.000 µl
Acq. Method : C:\CHEM32\1\DATA\23120601\23120601 2023-12-06 08-44-09\ZORBAX1.M
Last changed : 27.06.2019 11:09:07 by Gregor
Analysis Method : C:\PROGRAM FILES (X86)\CHEMSTATION\1\METHODS\DEF_LC.M
Additional Info : Peak(s) manually integrated

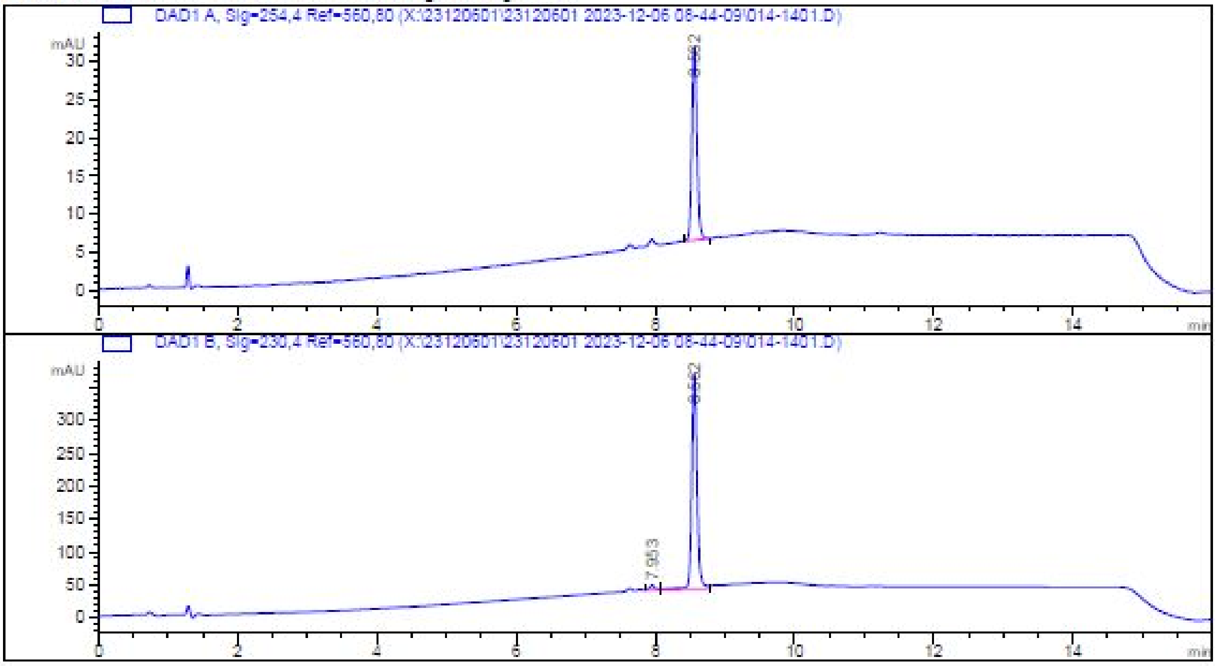

Area Percent Report

Sorted By : Signal
Multiplier: : 1.0000
Dilution: : 1.0000
Use Multiplier & Dilution Factor with ISTDs

Signal 1: DAD1 A, Sig=254,4 Ref=560,80

Peak #	RetTime [min]	Type	Width [min]	Area [mAU*s]	Height [mAU]	Area %
1	8.562	BB	0.0760	126.37993	25.43976	100.0000

Totals : 126.37993 25.43976

Signal 2: DAD1 B, Sig=230,4 Ref=560,80

Peak #	RetTime [min]	Type	Width [min]	Area [mAU*s]	Height [mAU]	Area %
1	7.953	BV	0.0796	37.37649	7.07844	2.1444
2	8.562	VV	0.0788	1705.63940	327.34149	97.8556

14f

```
=====================================================================
Acq. Operator   : Alex                          Seq. Line :   7
Acq. Instrument : Instrument 1                   Location : Vial 7
Injection Date  : 21.03.2023 15:14:13                 Inj :   1
                                               Inj Volume : 5.000 µl
Acq. Method     : C:\CHEM32\1\DATA\23032101\23032101 2023-03-21 13-06-31\ZORBAX1.M
Last changed    : 28.09.2021 15:34:30 by Flo
Analysis Method : C:\PROGRAM FILES (X86)\CHEMSTATION\1\METHODS\DEF_LC.M
Additional Info : Peak(s) manually integrated
```

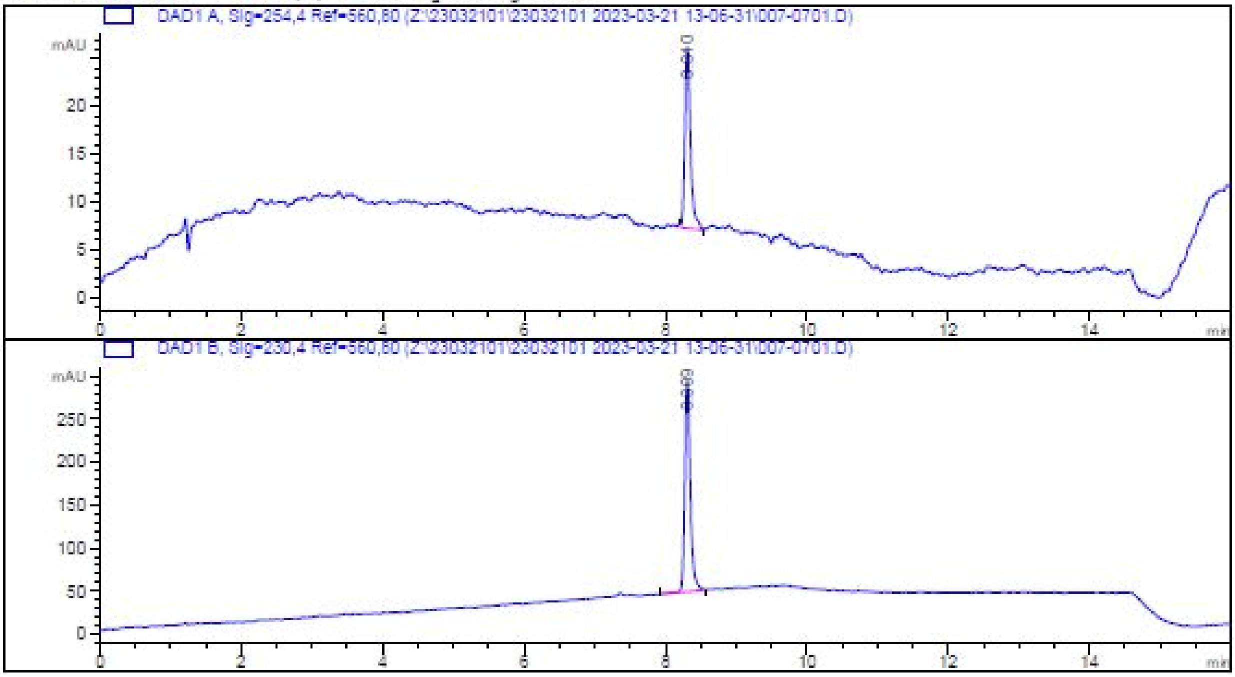


```
=====================================================================
                         Area Percent Report
=====================================================================

Sorted By             :      Signal
Multiplier:                   :      1.0000
Dilution:                     :      1.0000
Use Multiplier & Dilution Factor with ISTDs


Signal 1: DAD1 A, Sig=254,4 Ref=560,80

Peak RetTime Type  Width     Area      Height     Area
  #   [min]        [min]   [mAU*s]     [mAU]        %
----|-------|----|-------|----------|----------|--------|
   1   8.310 VB    0.0849  106.10252   19.06941 100.0000

Totals :                   106.10252   19.06941


Signal 2: DAD1 B, Sig=230,4 Ref=560,80

Peak RetTime Type  Width     Area      Height     Area
  #   [min]        [min]   [mAU*s]     [mAU]        %
----|-------|----|-------|----------|----------|--------|
   1   8.309 BB    0.0816 1341.14697  246.06294 100.0000
```

14g

```
=====================================================================
Acq. Operator   : Bene                              Seq. Line :  12
Acq. Instrument : Instrument 1                       Location : Vial 12
Injection Date  : 16.06.2023 14:54:46                     Inj :   1
                                                   Inj Volume : 5.000 µl
Acq. Method     : C:\CHEM32\1\DATA\23061601\23061602 2023-06-16 07-40-28\ZORBAX1.M
Last changed    : 28.09.2021 15:34:30 by Flo
Analysis Method : C:\CHEM32\1\METHODS\ZORBAX1.M
Last changed    : 28.09.2021 15:34:30 by Flo
Additional Info : Peak(s) manually integrated
```

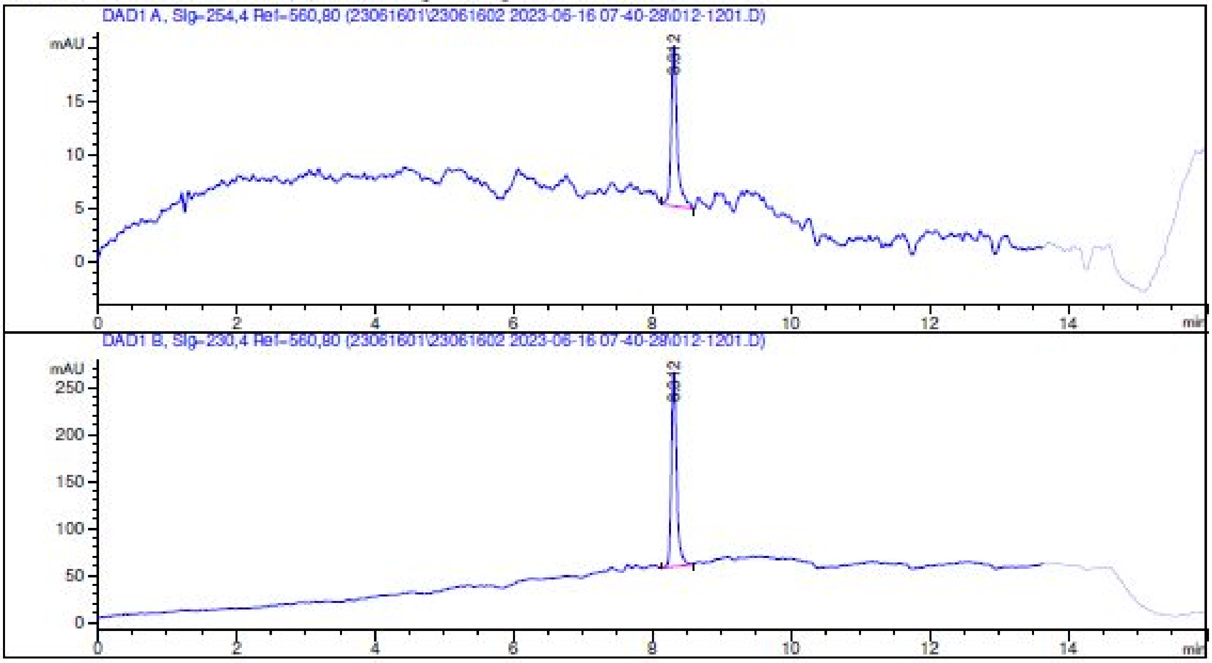


```
=====================================================================
                         Area Percent Report
=====================================================================

Sorted By             :      Signal
Multiplier:                  :       1.0000
Dilution:                    :       1.0000
Use Multiplier & Dilution Factor with ISTDs


Signal 1: DAD1 A, Sig=254,4 Ref=560,80

Peak RetTime Type  Width     Area      Height     Area
  #   [min]        [min]   [mAU*s]     [mAU]        %
----|-------|----|-------|----------|----------|--------|
   1   8.312 BB    0.0874   88.91631   14.94604 100.0000

Totals :                    88.91631   14.94604


Peak RetTime Type  Width     Area      Height     Area
  #   [min]        [min]   [mAU*s]     [mAU]        %
----|-------|----|-------|----------|----------|--------|
   1   8.312 BV    0.0792 1084.71875  206.77315 100.0000
```

14h

```
======================================================================
Acq. Operator   : Freddy                          Seq. Line :  11
Acq. Instrument : Instrument 1                     Location : Vial 11
Injection Date  : 16.06.2023 15:06:33                   Inj :   1
                                               Inj Volume : 5.000 µl
Acq. Method     : C:\CHEM32\1\DATA\23061601\23061601 2023-06-16 08-53-04\ZORBAX1.M
Last changed    : 27.06.2019 11:09:07 by Gregor
Analysis Method : C:\CHEM32\1\METHODS\ZORBAX1.M
Last changed    : 27.06.2019 11:09:07 by Gregor
Additional Info : Peak(s) manually integrated
```

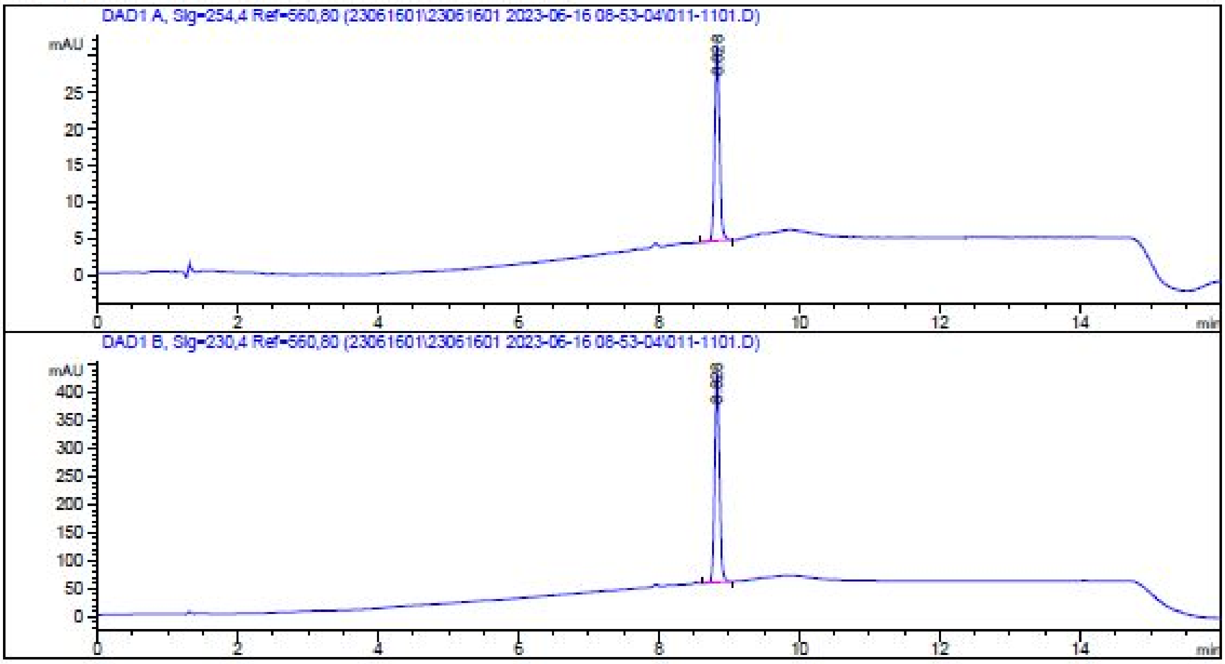


```
======================================================================
                         Area Percent Report
======================================================================

Sorted By             :      Signal
Multiplier:                  :      1.0000
Dilution:                    :      1.0000
Use Multiplier & Dilution Factor with ISTDs


Signal 1: DAD1 A, Sig=254,4 Ref=560,80

Peak RetTime Type  Width     Area      Height     Area
  #   [min]        [min]   [mAU*s]     [mAU]        %
----|-------|----|-------|----------|----------|--------|
   1   8.828 BB    0.0743  128.22592   26.59147 100.0000


Signal 2: DAD1 B, Sig=230,4 Ref=560,80

Peak RetTime Type  Width     Area      Height     Area
  #   [min]        [min]   [mAU*s]     [mAU]        %
----|-------|----|-------|----------|----------|--------|
   1   8.828 BB    0.0736 1775.89294  372.91135 100.0000
```

14i

```
=====================================================================
Acq. Operator   : Pascal                          Seq. Line :   3
Acq. Instrument : Instrument 1                     Location : Vial 3
Injection Date  : 04.12.2023 13:50:06                   Inj :   1
                                                Inj Volume : 5.000 µl
Acq. Method     : C:\CHEM32\1\DATA\23120401\23120401 2023-12-04 13-49-01\ZORBAX1.M
Last changed    : 28.09.2021 15:34:30 by Flo
Analysis Method : C:\PROGRAM FILES (X86)\CHEMSTATION\1\METHODS\DEF_LC.M
Additional Info : Peak(s) manually integrated
```

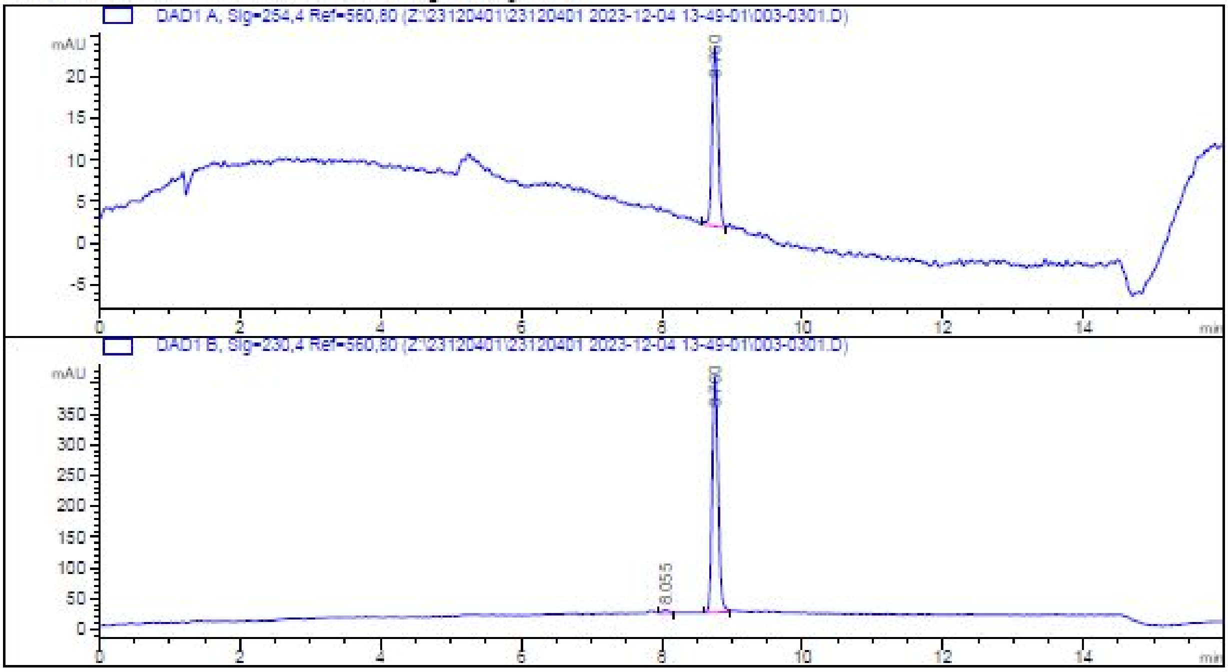


```
=====================================================================
                     Area Percent Report
=====================================================================

Sorted By             :      Signal
Multiplier:                  :      1.0000
Dilution:                    :      1.0000
Use Multiplier & Dilution Factor with ISTDs


Signal 1: DAD1 A, Sig=254,4 Ref=560,80

Peak RetTime Type  Width     Area      Height     Area
  #   [min]        [min]   [mAU*s]     [mAU]       %
----|-------|----|-------|----------|----------|--------|
   1   8.760 BV    0.0908  127.23988   21.59209 100.0000

Totals :                   127.23988   21.59209


Signal 2: DAD1 B, Sig=230,4 Ref=560,80

Peak RetTime Type  Width     Area      Height     Area
  #   [min]        [min]   [mAU*s]     [mAU]       %
----|-------|----|-------|----------|----------|--------|
   1   8.055 VB    0.1091   35.23910    5.19815   1.5288
   2   8.760 BV    0.0913 2269.73291  382.31110  98.4712
```

14j

```
Acq. Operator   : Bene                              Seq. Line :  10
Acq. Instrument : Instrument 1                       Location : Vial 10
Injection Date  : 20.04.2023 15:05:43                     Inj :   1
                                                   Inj Volume : 5.000 µl
Acq. Method     : C:\CHEM32\1\DATA\23042001\23042001 2023-04-20 08-02-09\ZORBAX1.M
Last changed    : 27.06.2019 11:09:07 by Gregor
Analysis Method : C:\CHEM32\1\METHODS\ZORBAX1.M
Last changed    : 27.06.2019 11:09:07 by Gregor
Additional Info : Peak(s) manually integrated
```

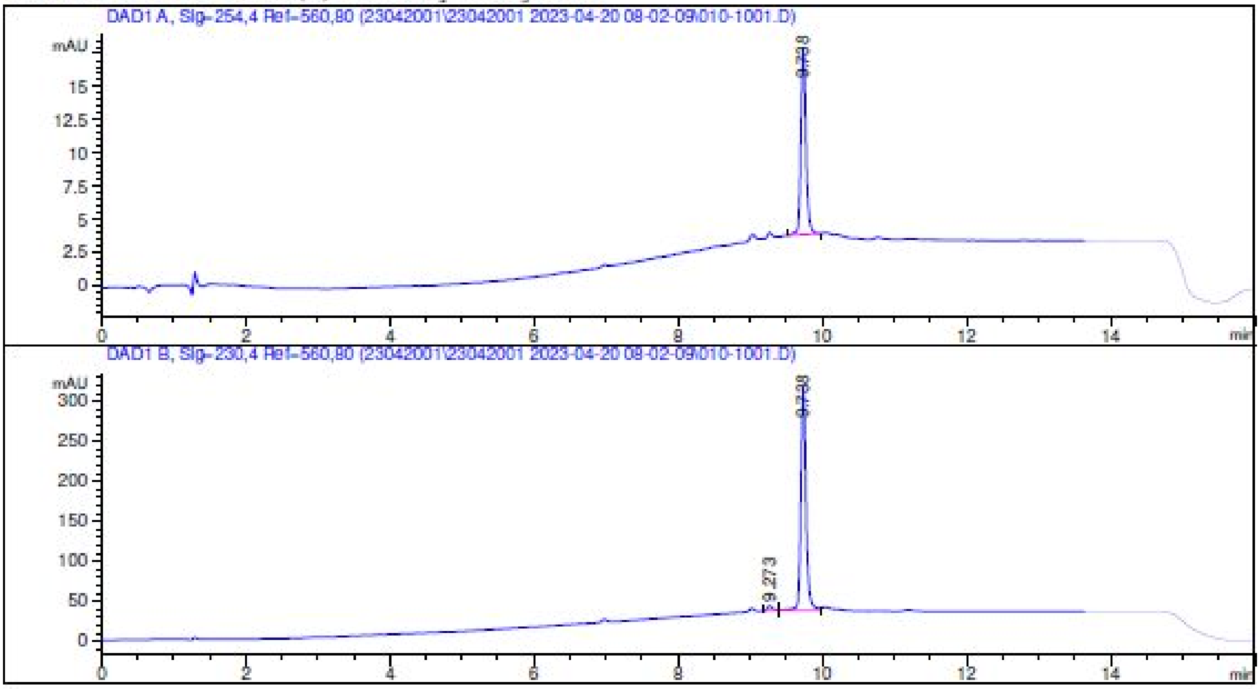

Area Percent Report

```
Sorted By             :      Signal
Multiplier:                  :      1.0000
Dilution:                    :      1.0000
Use Multiplier & Dilution Factor with ISTDs
```

Signal 1: DAD1 A, Sig=254,4 Ref=560,80

Peak #	RetTime [min]	Type	Width [min]	Area [mAU*s]	Height [mAU]	Area %
1	9.738	BB	0.0759	70.00239	14.09526	100.0000

Totals : 70.00239 14.09526

Peak #	RetTime [min]	Type	Width [min]	Area [mAU*s]	Height [mAU]	Area %
1	9.273	BB	0.0738	28.55097	5.96770	1.9815
2	9.738	BV	0.0768	1412.34033	280.16821	98.0185

14k

```
=====================================================================
Acq. Operator   : Bene                           Seq. Line :    7
Acq. Instrument : Instrument 1                    Location : Vial 7
Injection Date  : 21.06.2023 12:48:01                  Inj :    1
                                                Inj Volume : 5.000 µl
Acq. Method     : C:\CHEM32\1\DATA\23062101\23062101 2023-06-21 12-29-51\ZORBAX1.M
Last changed    : 28.09.2021 15:34:30 by Flo
Analysis Method : C:\CHEM32\1\METHODS\ZORBAX1.M
Last changed    : 28.09.2021 15:34:30 by Flo
Additional Info : Peak(s) manually integrated
```

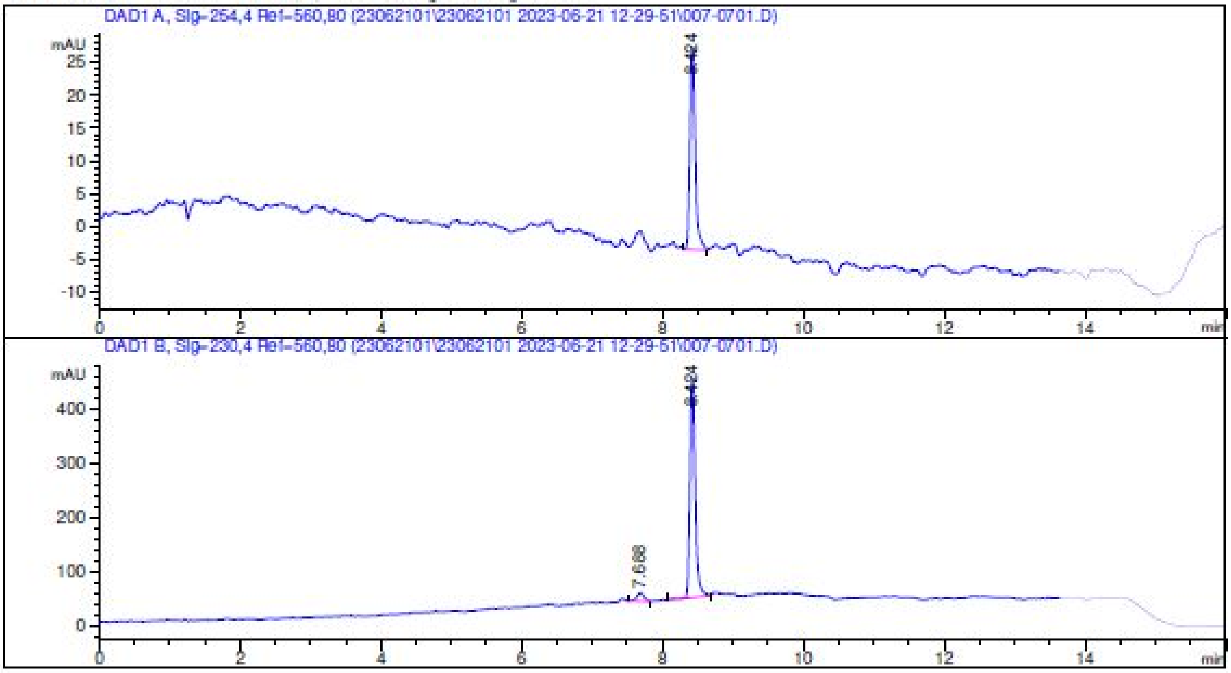


```
=====================================================================
                         Area Percent Report
=====================================================================

Sorted By             :      Signal
Multiplier:                  :      1.0000
Dilution:                    :      1.0000
Use Multiplier & Dilution Factor with ISTDs


Signal 1: DAD1 A, Sig=254,4 Ref=560,80

Peak RetTime Type  Width     Area      Height     Area
  #    [min]        [min]   [mAU*s]     [mAU]       %
----|-------|----|-------|----------|----------|--------|
   1   8.424 BB    0.0754  158.22021   31.07209 100.0000
```

```
Peak RetTime Type  Width     Area       Height      Area
  #    [min]        [min]   [mAU*s]      [mAU]        %
----|-------|----|-------|----------|----------|--------|
   1   7.688 BB    0.1074  103.09982   14.76963   4.7235
   2   8.424 BV    0.0775 2079.59351  407.44928  95.2765
```

14m

```
=====================================================================
Acq. Operator   : Philipp                        Seq. Line :    7
Acq. Instrument : Instrument 1                    Location : Vial 7
Injection Date  : 24.01.2023 13:49:55                  Inj :    1
                                               Inj Volume : 5.000 µl
Acq. Method     : C:\CHEM32\1\DATA\23012401\23012401 2023-01-24 08-07-33\ZORBAX1.M
Last changed    : 27.06.2019 11:09:07 by Gregor
Analysis Method : C:\PROGRAM FILES (X86)\CHEMSTATION\1\METHODS\DEF_LC.M
Additional Info : Peak(s) manually integrated
```

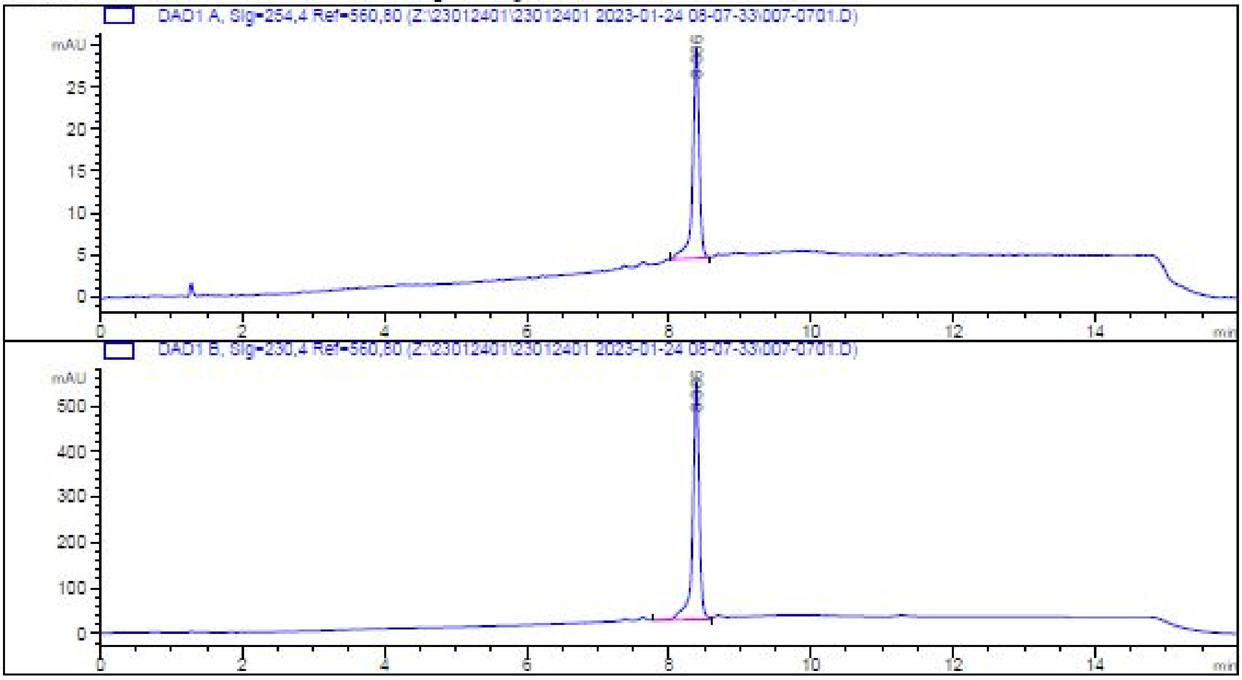


```
=====================================================================
                         Area Percent Report
=====================================================================

Sorted By             :      Signal
Multiplier:                    :      1.0000
Dilution:                      :      1.0000
Use Multiplier & Dilution Factor with ISTDs


Signal 1: DAD1 A, Sig=254,4 Ref=560,80

Peak RetTime Type  Width     Area      Height     Area
  #   [min]        [min]   [mAU*s]     [mAU]        %
----|-------|----|-------|----------|----------|--------|
   1   8.386 BB    0.0930  157.47594   25.15134 100.0000

Totals :                   157.47594   25.15134


Signal 2: DAD1 B, Sig=230,4 Ref=560,80

Peak RetTime Type  Width     Area      Height     Area
  #   [min]        [min]   [mAU*s]     [mAU]        %
----|-------|----|-------|----------|----------|--------|
   1   8.386 BV    0.0920 3312.05420  521.86890 100.0000
```

14n

```
=====================================================================
Acq. Operator   : Rapha                          Seq. Line :    7
Acq. Instrument : Instrument 1                    Location : Vial 7
Injection Date  : 19.06.2023 13:45:35                   Inj :    1
                                                Inj Volume : 5.000 µl
Acq. Method     : C:\CHEM32\1\DATA\23061901\23061901 2023-06-19 13-10-19\ZORBAX1.M
Last changed    : 28.09.2021 15:34:30 by Flo
Analysis Method : C:\PROGRAM FILES (X86)\CHEMSTATION\1\METHODS\DEF_LC.M
Additional Info : Peak(s) manually integrated
```

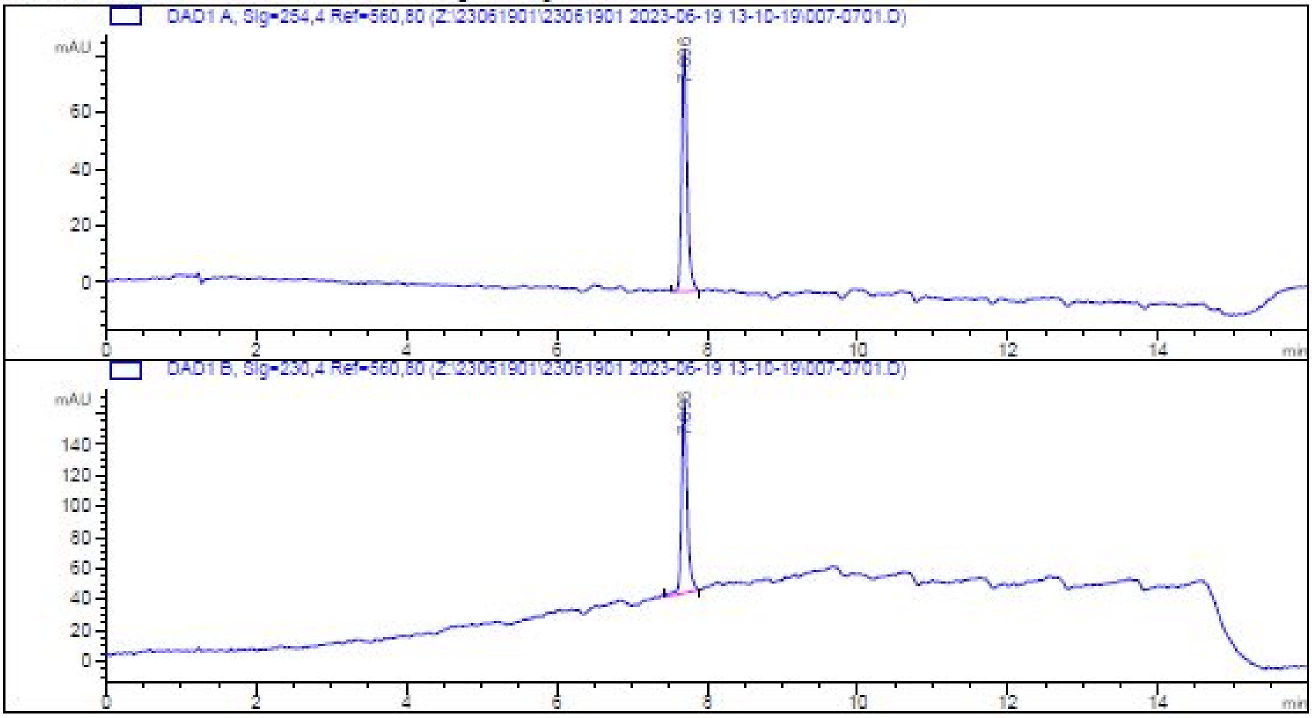


```
=====================================================================
                         Area Percent Report
=====================================================================

Sorted By             :      Signal
Multiplier:                  :      1.0000
Dilution:                    :      1.0000
Use Multiplier & Dilution Factor with ISTDs


Signal 1: DAD1 A, Sig=254,4 Ref=560,80

Peak RetTime Type  Width     Area      Height     Area
  #   [min]        [min]   [mAU*s]     [mAU]        %
----|-------|----|-------|----------|----------|--------|
   1   7.696 BB    0.0781  441.25458   85.67175 100.0000

Totals :                   441.25458   85.67175


Signal 2: DAD1 B, Sig=230,4 Ref=560,80

Peak RetTime Type  Width     Area      Height     Area
  #   [min]        [min]   [mAU*s]     [mAU]        %
----|-------|----|-------|----------|----------|--------|
   1   7.696 VB    0.0809  662.69318  122.94835 100.0000
```

14o

```
=====================================================================
Acq. Operator   : Rapha                          Seq. Line :    9
Acq. Instrument : Instrument 1                    Location : Vial 9
Injection Date  : 19.06.2023 15:05:44                  Inj :    1
                                                Inj Volume : 5.000 µl
Acq. Method     : C:\CHEM32\1\DATA\23061901\23061901 2023-06-19 13-10-19\ZORBAX1.M
Last changed    : 28.09.2021 15:34:30 by Flo
Analysis Method : C:\PROGRAM FILES (X86)\CHEMSTATION\1\METHODS\DEF_LC.M
Additional Info : Peak(s) manually integrated
```

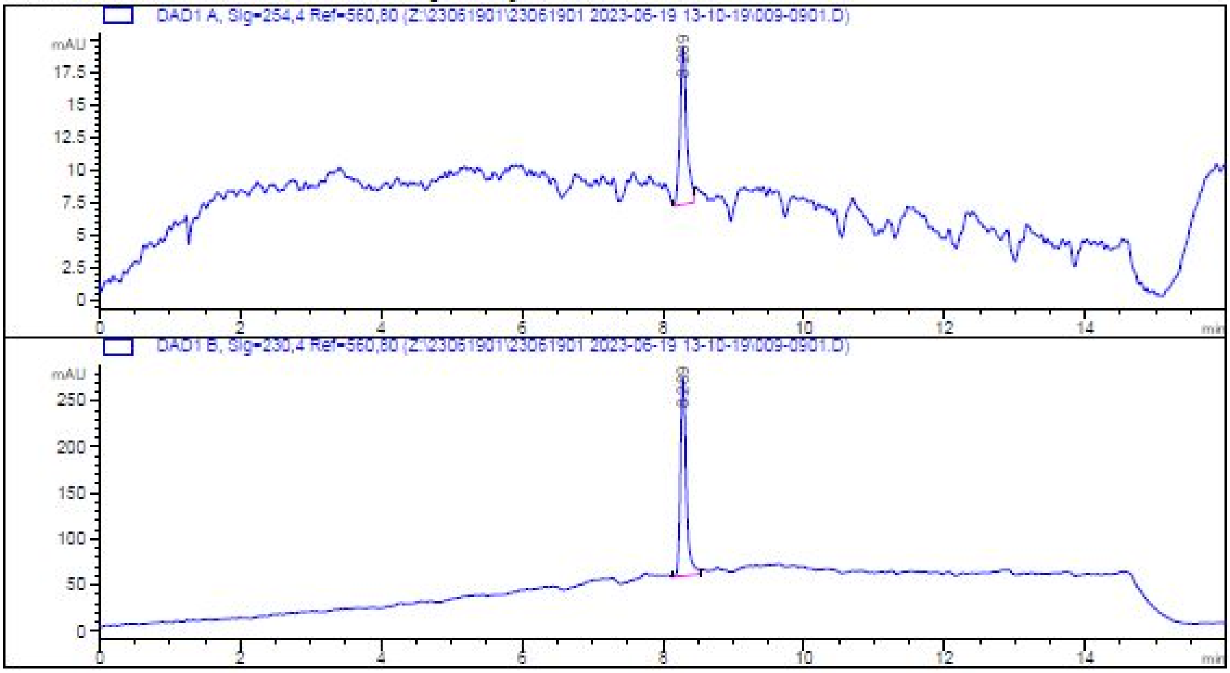


```
=====================================================================
                         Area Percent Report
=====================================================================

Sorted By             :      Signal
Multiplier:                    :       1.0000
Dilution:                      :       1.0000
Use Multiplier & Dilution Factor with ISTDs


Signal 1: DAD1 A, Sig=254,4 Ref=560,80

Peak RetTime Type  Width     Area      Height     Area
  #   [min]        [min]   [mAU*s]     [mAU]        %
----|-------|----|-------|----------|----------|--------|
   1   8.289 BV    0.0953   78.08306   12.09621 100.0000

Totals :                    78.08306   12.09621


Signal 2: DAD1 B, Sig=230,4 Ref=560,80

Peak RetTime Type  Width     Area      Height     Area
  #   [min]        [min]   [mAU*s]     [mAU]        %
----|-------|----|-------|----------|----------|--------|
   1   8.289 BV    0.0875 1246.66003  215.34679 100.0000
```

14p

```
Acq. Operator   : Philipp                          Seq. Line :   2
Acq. Instrument : Instrument 1                      Location : Vial 2
Injection Date  : 24.03.2023 08:09:04                    Inj :   1
                                                  Inj Volume : 5.000 µl
Acq. Method     : C:\CHEM32\1\DATA\23032401\23032401 2023-03-24 07-50-53\ZORBAX1.M
Last changed    : 28.09.2021 15:34:30 by Flo
Analysis Method : C:\CHEM32\1\METHODS\ZORBAX1.M
Last changed    : 28.09.2021 15:34:30 by Flo
Additional Info : Peak(s) manually integrated
```

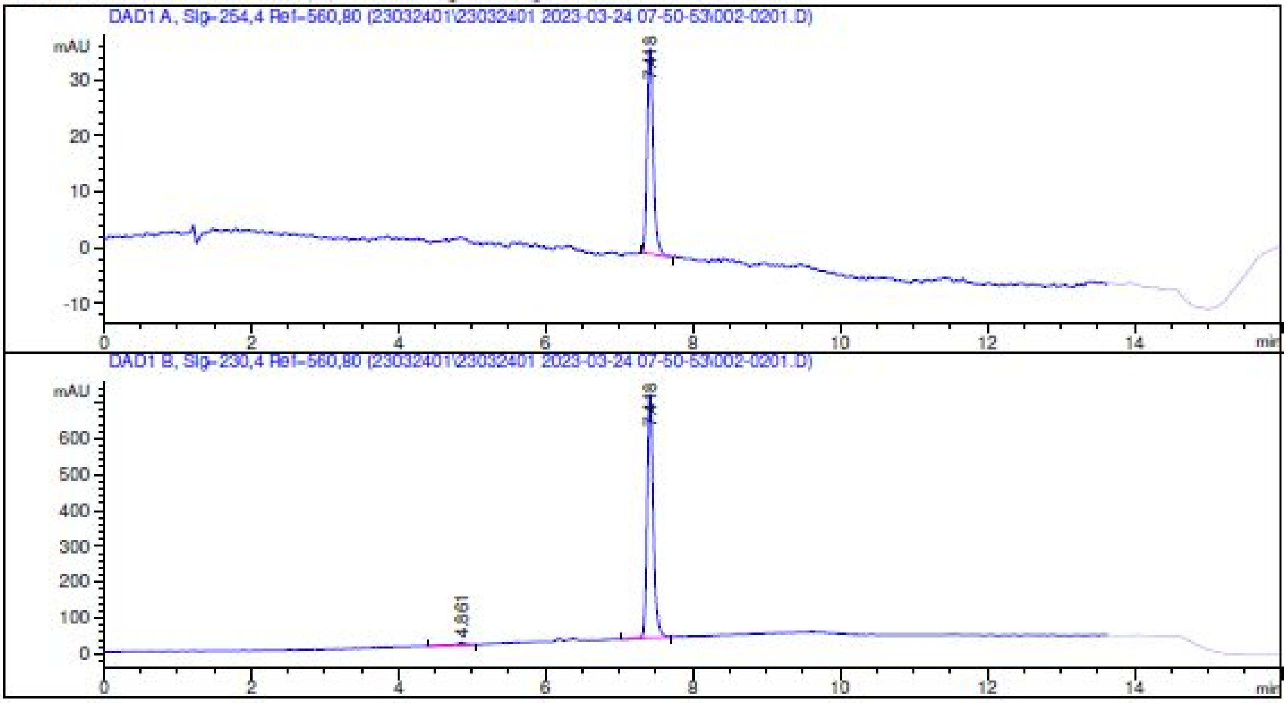

Area Percent Report

```
Sorted By             :      Signal
Multiplier:                  :      1.0000
Dilution:                    :      1.0000
Use Multiplier & Dilution Factor with ISTDs
```

Signal 1: DAD1 A, Sig=254,4 Ref=560,80

Peak #	RetTime [min]	Type	Width [min]	Area [mAU*s]	Height [mAU]	Area %
1	7.418	BB	0.0861	207.75070	36.69154	100.0000

Totals : 207.75070 36.69154

Peak #	RetTime [min]	Type	Width [min]	Area [mAU*s]	Height [mAU]	Area %
1	4.861	BB	0.1386	68.80330	7.00442	1.7523
2	7.418	BV	0.0864	3857.57422	677.34973	98.2477

14q

```
=====================================================================
Acq. Operator   : Rapha                              Seq. Line :   6
Acq. Instrument : Instrument 1                        Location : Vial 6
Injection Date  : 19.06.2023 13:28:27                      Inj :   1
                                                    Inj Volume : 5.000 µl
Acq. Method     : C:\CHEM32\1\DATA\23061901\23061901 2023-06-19 13-10-19\ZORBAX1.M
Last changed    : 28.09.2021 15:34:30 by Flo
Analysis Method : C:\PROGRAM FILES (X86)\CHEMSTATION\1\METHODS\DEF_LC.M
Additional Info : Peak(s) manually integrated
```

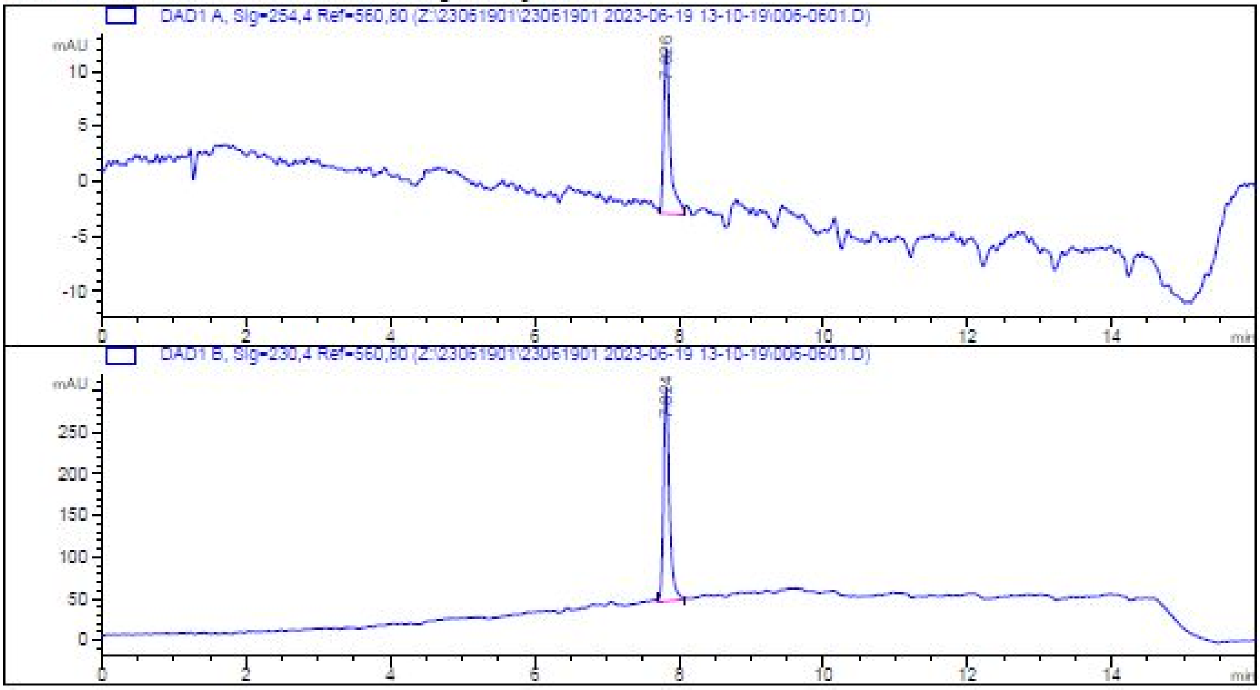


```
=====================================================================
                        Area Percent Report
=====================================================================

Sorted By             :      Signal
Multiplier:                  :      1.0000
Dilution:                    :      1.0000
Use Multiplier & Dilution Factor with ISTDs


Signal 1: DAD1 A, Sig=254,4 Ref=560,80

Peak RetTime Type  Width     Area      Height     Area
  #   [min]        [min]   [mAU*s]     [mAU]        %
----|-------|----|-------|----------|----------|--------|
   1   7.826 BV    0.0940   95.46284   15.04751 100.0000

Totals :                    95.46284   15.04751


Signal 2: DAD1 B, Sig=230,4 Ref=560,80

Peak RetTime Type  Width     Area      Height     Area
  #   [min]        [min]   [mAU*s]     [mAU]        %
----|-------|----|-------|----------|----------|--------|
   1   7.824 VV    0.0887 1516.89124  257.58679 100.0000
```

14r

```
=====================================================================
Acq. Operator   : Philipp                        Seq. Line :   7
Acq. Instrument : Instrument 1                    Location : Vial 7
Injection Date  : 24.01.2023 13:50:32                  Inj :   1
                                               Inj Volume : 5.000 µl
Acq. Method     : C:\CHEM32\1\DATA\23012401\23012401 2023-01-24 08-06-07\ZORBAX1.M
Last changed    : 28.09.2021 15:34:30 by Flo
Analysis Method : C:\PROGRAM FILES (X86)\CHEMSTATION\1\METHODS\DEF_LC.M
Additional Info : Peak(s) manually integrated
```

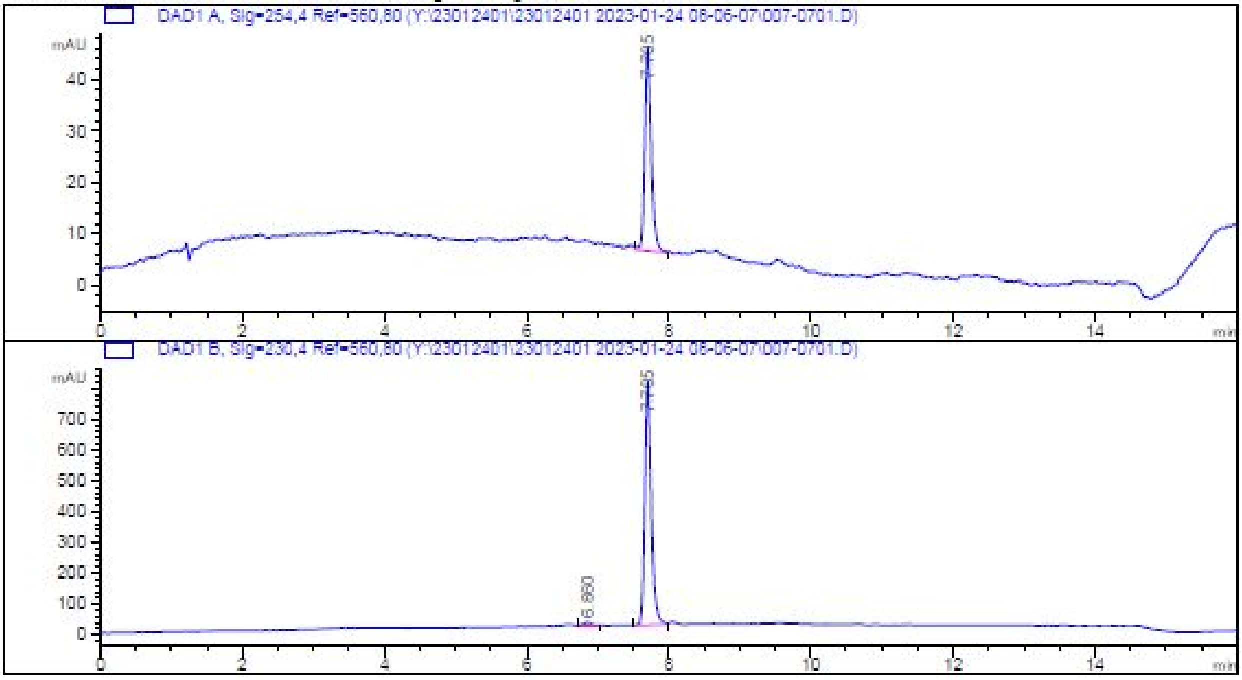


```
=====================================================================
                         Area Percent Report
=====================================================================

Sorted By             :      Signal
Multiplier:                   :      1.0000
Dilution:                     :      1.0000
Use Multiplier & Dilution Factor with ISTDs


Signal 1: DAD1 A, Sig=254,4 Ref=560,80

Peak RetTime Type  Width     Area      Height     Area
  #   [min]        [min]   [mAU*s]     [mAU]        %
----|-------|----|-------|----------|----------|--------|
   1   7.705 VV    0.0968  253.93518   39.63263 100.0000

Totals :                   253.93518   39.63263


Signal 2: DAD1 B, Sig=230,4 Ref=560,80

Peak RetTime Type  Width     Area      Height     Area
  #   [min]        [min]   [mAU*s]     [mAU]        %
----|-------|----|-------|----------|----------|--------|
   1   6.860 BV    0.1237   86.29530   10.51871   1.6642
   2   7.705 BV    0.0992 5099.01416  790.66211  98.3358
```

14s

===

Acq. Operator : Bene Seq. Line : 16
Acq. Instrument : Instrument 1 Location : Vial 16
Injection Date : 11.12.2023 15:40:03 Inj : 1
Inj Volume : 5.000 µl
Acq. Method : C:\CHEM32\1\DATA\23121101\23121101 2023-12-11 09-32-23\ZORBAX1.M
Last changed : 27.06.2019 11:09:07 by Gregor
Analysis Method : C:\PROGRAM FILES (X86)\CHEMSTATION\1\METHODS\DEF_LC.M
Additional Info : Peak(s) manually integrated

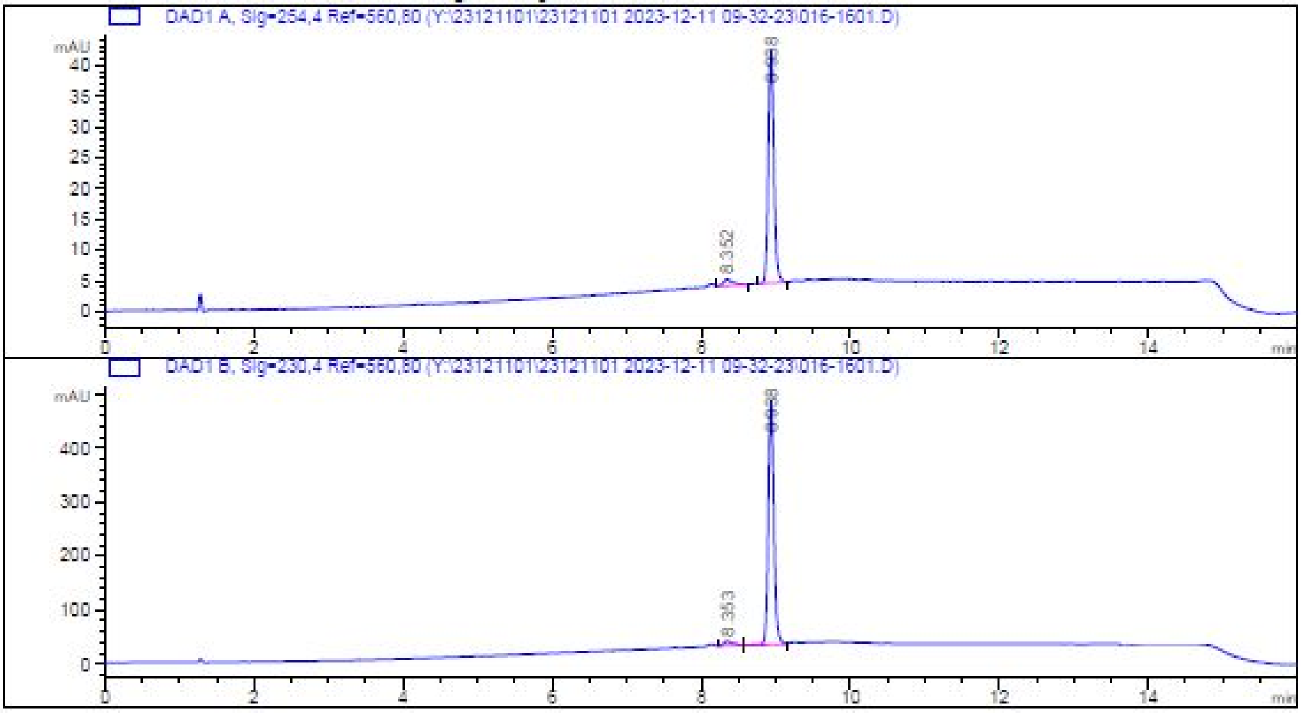

===
Area Percent Report
===

Sorted By : Signal
Multiplier: : 1.0000
Dilution: : 1.0000
Use Multiplier & Dilution Factor with ISTDs

Signal 1: DAD1 A, Sig=254,4 Ref=560,80

Peak #	RetTime [min]	Type	Width [min]	Area [mAU*s]	Height [mAU]	Area %
1	8.352	BB	0.1214	9.03835	1.03992	4.4602
2	8.938	BB	0.0796	193.60712	37.88543	95.5398

Peak #	RetTime [min]	Type	Width [min]	Area [mAU*s]	Height [mAU]	Area %
1	8.353	VB	0.1242	75.34052	8.60245	3.1223
2	8.938	BV	0.0802	2337.63062	453.21088	96.8777

20a

```
Acq. Operator   : Pascal                        Seq. Line :   5
Acq. Instrument : Instrument 1                   Location : Vial 5
Injection Date  : 28.04.2023 13:09:40                 Inj :   1
                                               Inj Volume : 5.000 µl
Acq. Method     : C:\CHEM32\1\DATA\23042801\23042801 2023-04-28 10-38-28\ZORBAX1.M
Last changed    : 27.06.2019 11:09:07 by Gregor
Analysis Method : C:\CHEM32\1\METHODS\ZORBAX1.M
Last changed    : 27.06.2019 11:09:07 by Gregor
Additional Info : Peak(s) manually integrated
```

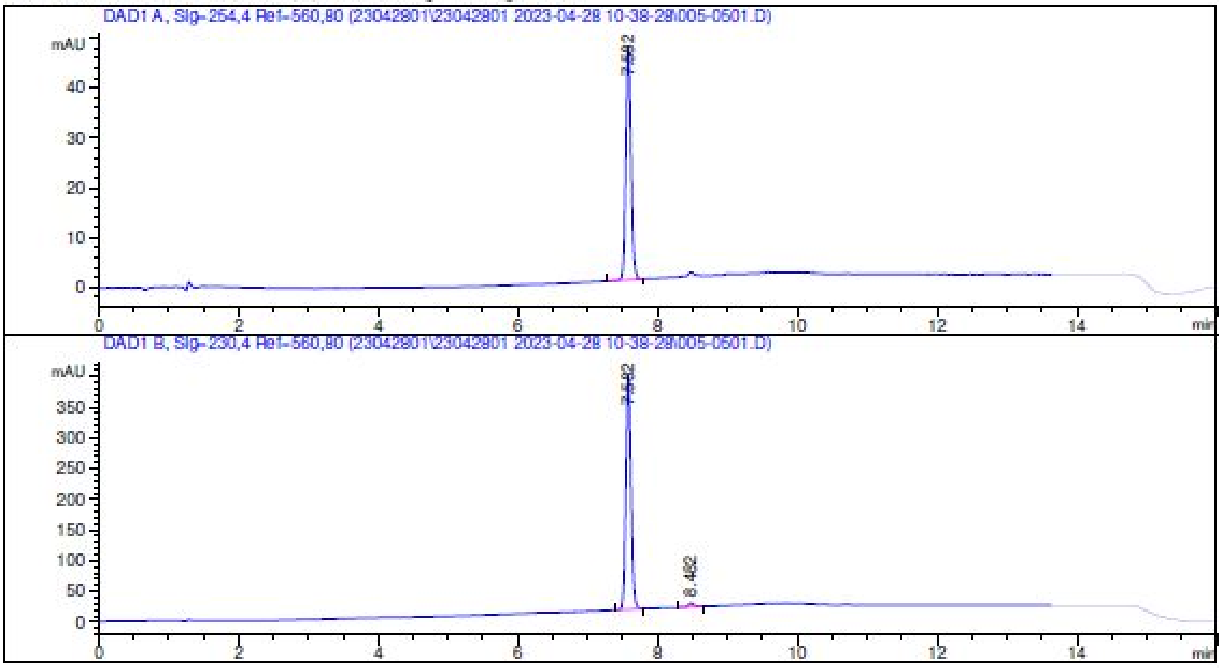

Area Percent Report

```
Sorted By              :      Signal
Multiplier:                   :      1.0000
Dilution:                     :      1.0000
Use Multiplier & Dilution Factor with ISTDs
```

Signal 1: DAD1 A, Sig=254,4 Ref=560,80

Peak #	RetTime [min]	Type	Width [min]	Area [mAU*s]	Height [mAU]	Area %
1	7.582	BB	0.0810	244.96249	46.84616	100.0000

Peak #	RetTime [min]	Type	Width [min]	Area [mAU*s]	Height [mAU]	Area %
1	7.582	BB	0.0805	1983.52368	382.18988	98.3481
2	8.482	BB	0.0805	33.31655	6.21465	1.6519

20b

==

Acq. Operator : Moritz Seq. Line : 15
Acq. Instrument : Instrument 1 Location : Vial 15
Injection Date : 03.04.2024 13:00:35 Inj : 1
Inj Volume : 5.000 µl
Acq. Method : C:\CHEM32\1\DATA\24040301\24040301 2024-04-03 08-08-27\ZORBAX1.M
Last changed : 27.06.2019 11:09:07 by Gregor
Analysis Method : C:\CHEM32\1\METHODS\ZORBAX1.M
Last changed : 27.06.2019 11:09:07 by Gregor
Additional Info : Peak(s) manually integrated

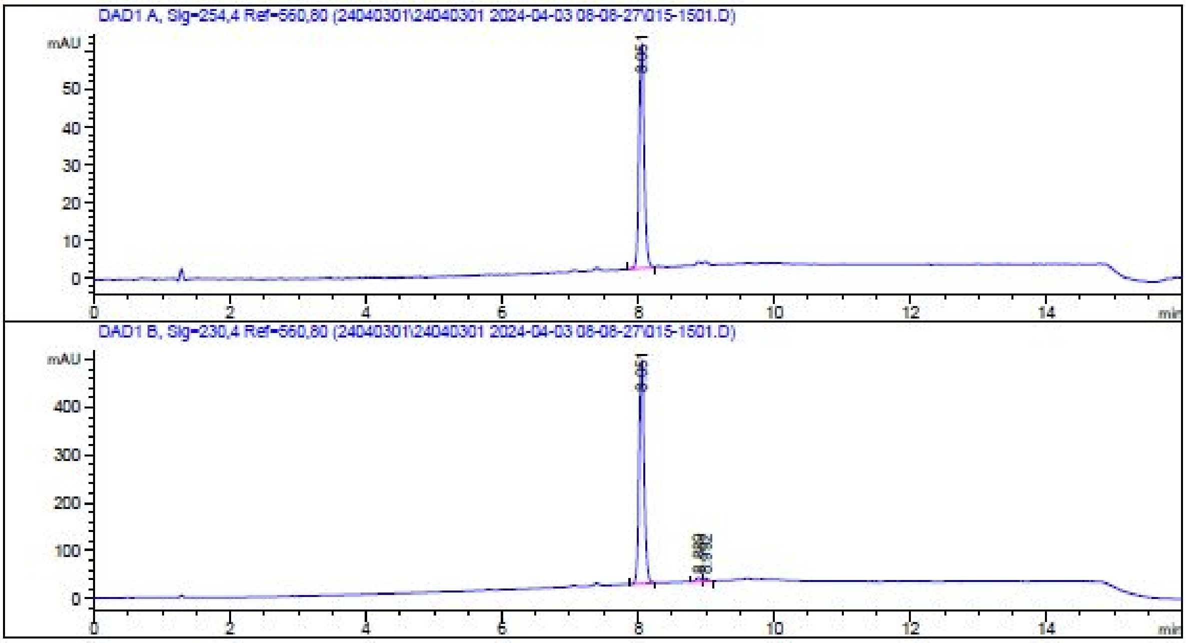

==

Area Percent Report

==

Sorted By : Signal
Multiplier: : 1.0000
Dilution: : 1.0000
Use Multiplier & Dilution Factor with ISTDs

Signal 1: DAD1 A, Sig=254,4 Ref=560,80

Peak #	RetTime [min]	Type	Width [min]	Area [mAU*s]	Height [mAU]	Area %
1	8.051	BB	0.0806	306.78912	59.11013	100.0000

[illegible]

Signal 2: DAD1 B, Sig=230,4 Ref=560,80

Peak #	RetTime [min]	Type	Width [min]	Area [mAU*s]	Height [mAU]	Area %
1	8.051	BB	0.0799	2388.59473	464.90762	97.2225
2	8.889	BV	0.0747	38.12525	7.84358	1.5518
3	8.992	VB	0.0739	30.11237	6.06468	1.2257

20c

```
Acq. Operator   : Bene                              Seq. Line :   7
Acq. Instrument : Instrument 1                       Location : Vial 7
Injection Date  : 05.04.2024 14:42:53                     Inj :   1
                                                   Inj Volume : 5.000 µl
Acq. Method     : C:\CHEM32\1\DATA\24040501\24040501 2024-04-05 14-24-42\ZORBAX1.M
Last changed    : 28.09.2021 15:34:30 by Flo
Analysis Method : C:\CHEM32\1\METHODS\ZORBAX1.M
Last changed    : 28.09.2021 15:34:30 by Flo
Additional Info : Peak(s) manually integrated
```

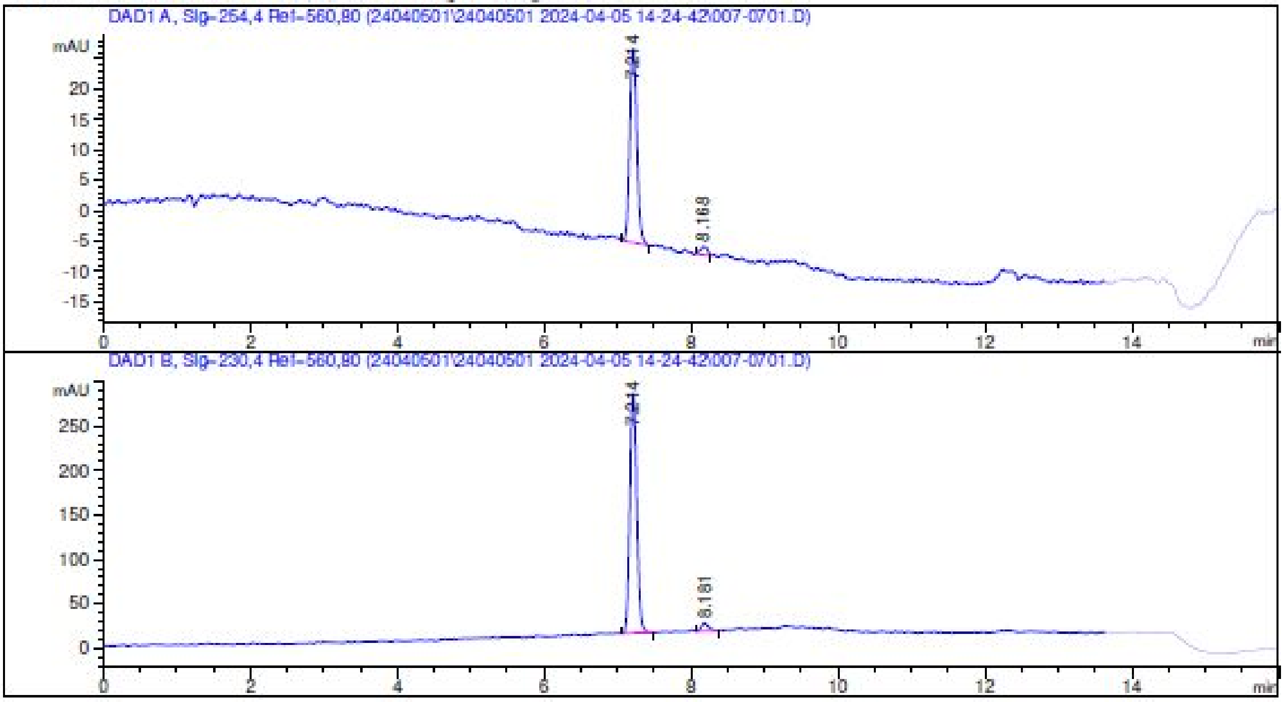

Area Percent Report

```
Sorted By             :      Signal
Multiplier:                  :      1.0000
Dilution:                    :      1.0000
Use Multiplier & Dilution Factor with ISTDs
```

Signal 1: DAD1 A, Sig=254,4 Ref=560,80

Peak #	RetTime [min]	Type	Width [min]	Area [mAU*s]	Height [mAU]	Area %
1	7.214	BB	0.1029	210.74124	31.96292	95.6841
2	8.168	VV	0.1168	9.50568	1.35620	4.3159

Signal 2: DAD1 B, Sig=230,4 Ref=560,80

Peak #	RetTime [min]	Type	Width [min]	Area [mAU*s]	Height [mAU]	Area %
1	7.214	BB	0.1013	1735.00562	268.89020	96.4335
2	8.181	VV	0.1018	64.16803	9.15562	3.5665

20d

Acq. Operator : Bene Seq. Line : 5
Acq. Instrument : Instrument 1 Location : Vial 5
Injection Date : 05.04.2024 13:52:18 Inj : 1
Inj Volume : 5.000 µl
Acq. Method : C:\CHEM32\1\DATA\24040501\24040501 2024-04-05 13-34-09\ZORBAX1.M
Last changed : 28.09.2021 15:34:30 by Flo
Analysis Method : C:\CHEM32\1\METHODS\ZORBAX1.M
Last changed : 28.09.2021 15:34:30 by Flo
Additional Info : Peak(s) manually integrated

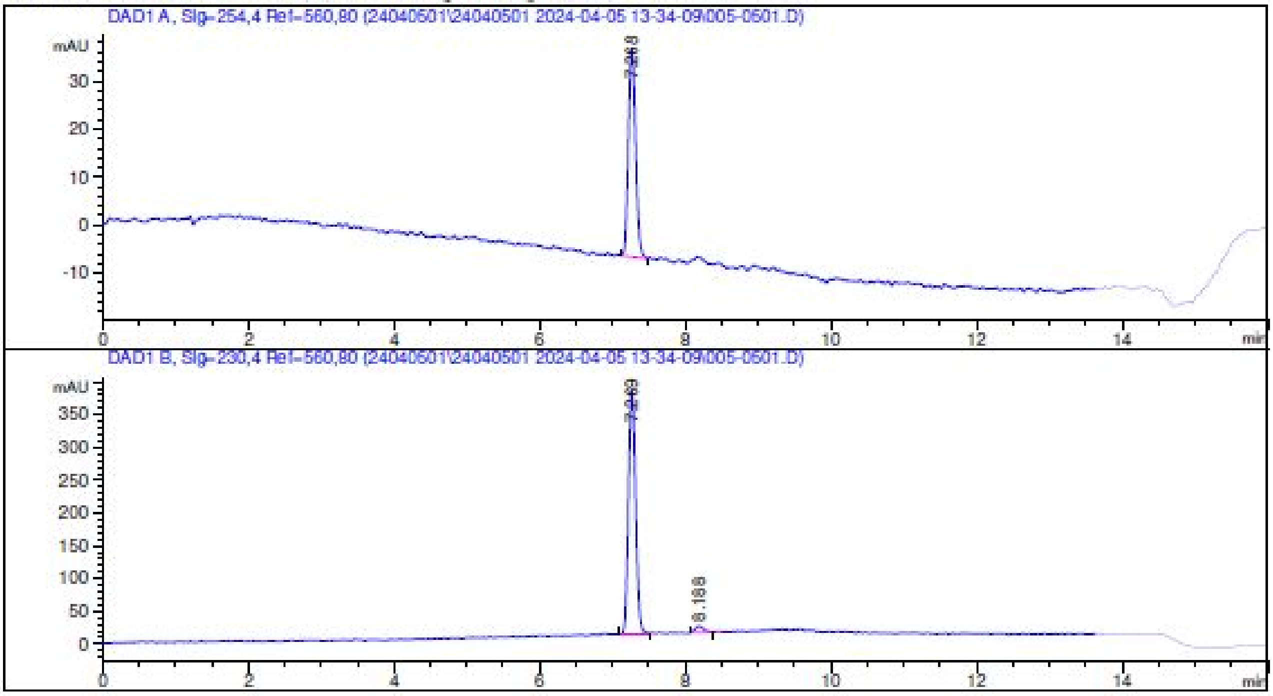

Area Percent Report

Sorted By : Signal
Multiplier: : 1.0000
Dilution: : 1.0000
Use Multiplier & Dilution Factor with ISTDs

Signal 1: DAD1 A, Sig=254,4 Ref=560,80

Peak #	RetTime [min]	Type	Width [min]	Area [mAU*s]	Height [mAU]	Area %
1	7.268	BB	0.1077	296.80966	43.47493	100.0000

Peak #	RetTime [min]	Type	Width [min]	Area [mAU*s]	Height [mAU]	Area %
1	7.269	BV	0.1077	2543.60693	372.50122	96.9363
2	8.188	BB	0.1102	80.39072	9.74867	3.0637

20e

```
Acq. Operator   : Bene                              Seq. Line :    6
Acq. Instrument : Instrument 1                       Location : Vial 6
Injection Date  : 05.04.2024 14:25:47                     Inj :    1
                                                  Inj Volume : 5.000 µl
Acq. Method     : C:\CHEM32\1\DATA\24040501\24040501 2024-04-05 14-24-42\ZORBAX1.M
Last changed    : 28.09.2021 15:34:30 by Flo
Analysis Method : C:\CHEM32\1\METHODS\ZORBAX1.M
Last changed    : 28.09.2021 15:34:30 by Flo
Additional Info : Peak(s) manually integrated
```

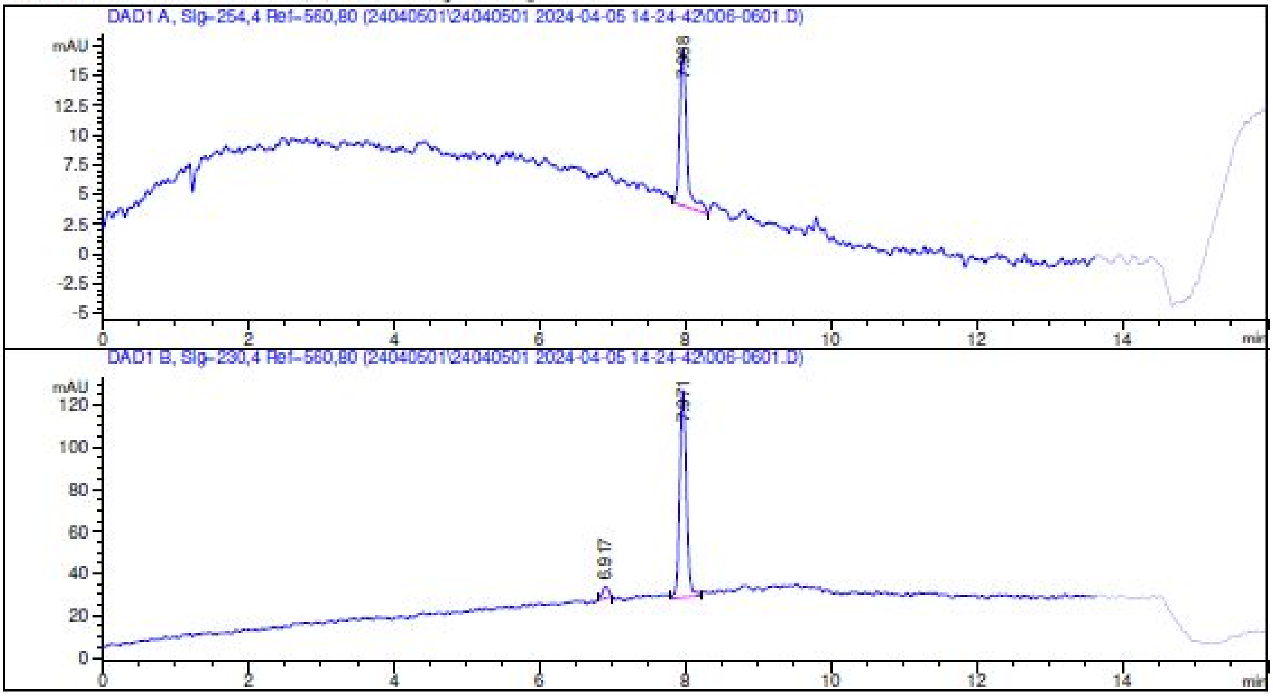

Area Percent Report

Sorted By : Signal
Multiplier: : 1.0000
Dilution: : 1.0000
Use Multiplier & Dilution Factor with ISTDs

Signal 1: DAD1 A, Sig=254,4 Ref=560,80

Peak #	RetTime [min]	Type	Width [min]	Area [mAU*s]	Height [mAU]	Area %
1	7.968	BB	0.1027	89.36568	13.24736	100.0000

Peak #	RetTime [min]	Type	Width [min]	Area [mAU*s]	Height [mAU]	Area %
1	6.917	BB	0.1005	32.16281	5.33374	4.8302
2	7.971	VV	0.1072	633.70667	98.56995	95.1698

20f

```
Acq. Operator   : Pascal                          Seq. Line :   6
Acq. Instrument : Instrument 1                     Location : Vial 6
Injection Date  : 28.04.2023 13:26:40                   Inj :   1
                                                Inj Volume : 5.000 µl
Acq. Method     : C:\CHEM32\1\DATA\23042801\23042801 2023-04-28 10-38-28\ZORBAX1.M
Last changed    : 27.06.2019 11:09:07 by Gregor
Analysis Method : C:\CHEM32\1\METHODS\ZORBAX1.M
Last changed    : 27.06.2019 11:09:07 by Gregor
Additional Info : Peak(s) manually integrated
```

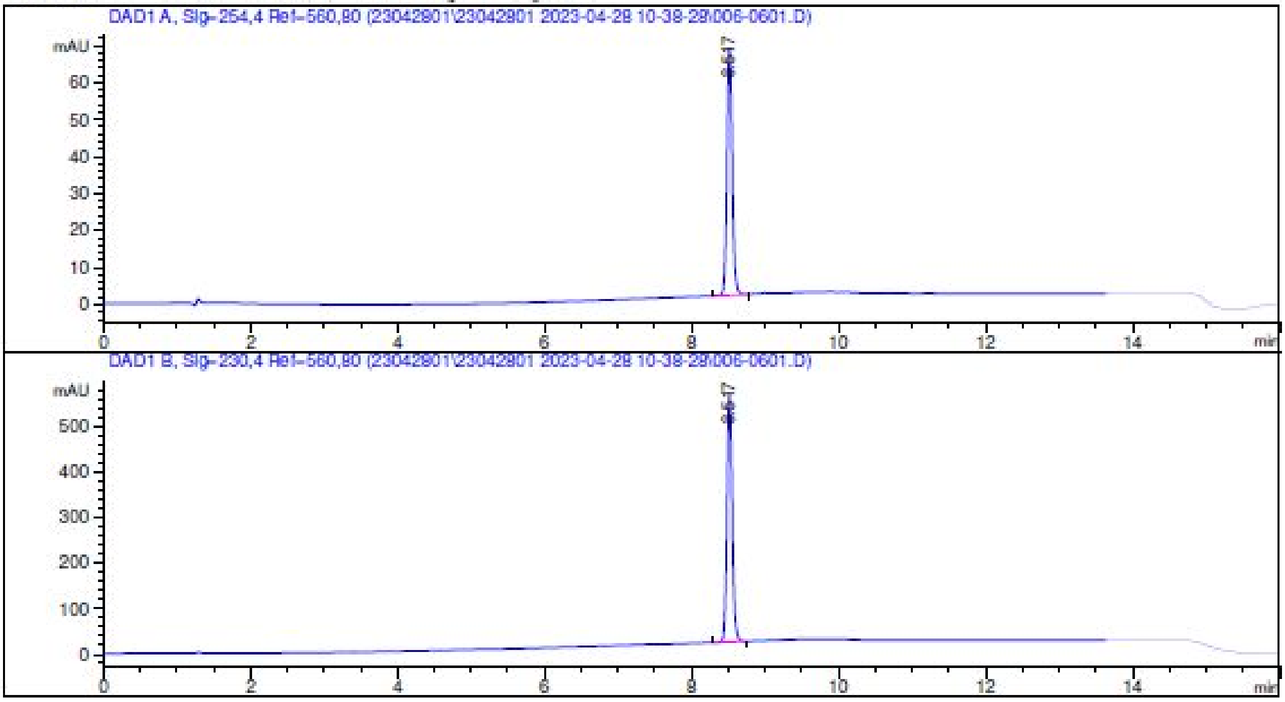

Area Percent Report

```
Sorted By             :      Signal
Multiplier:                  :      1.0000
Dilution:                    :      1.0000
Use Multiplier & Dilution Factor with ISTDs
```

Signal 1: DAD1 A, Sig=254,4 Ref=560,80

Peak #	RetTime [min]	Type	Width [min]	Area [mAU*s]	Height [mAU]	Area %
1	8.517	BB	0.0782	334.09561	66.95851	100.0000

Peak #	RetTime [min]	Type	Width [min]	Area [mAU*s]	Height [mAU]	Area %
1	8.517	BB	0.0778	2706.02515	546.56647	100.0000

20g

```
Acq. Operator   : Bene                               Seq. Line :    5
Acq. Instrument : Instrument 1                        Location : Vial 5
Injection Date  : 09.08.2023 11:32:09                      Inj :    1
                                                    Inj Volume : 5.000 µl
Acq. Method     : C:\CHEM32\1\DATA\23080901\23080901 2023-08-09 11-31-03\ZORBAX1.M
Last changed    : 28.09.2021 15:34:30 by Flo
Analysis Method : C:\PROGRAM FILES (X86)\CHEMSTATION\1\METHODS\DEF_LC.M
Additional Info : Peak(s) manually integrated
```

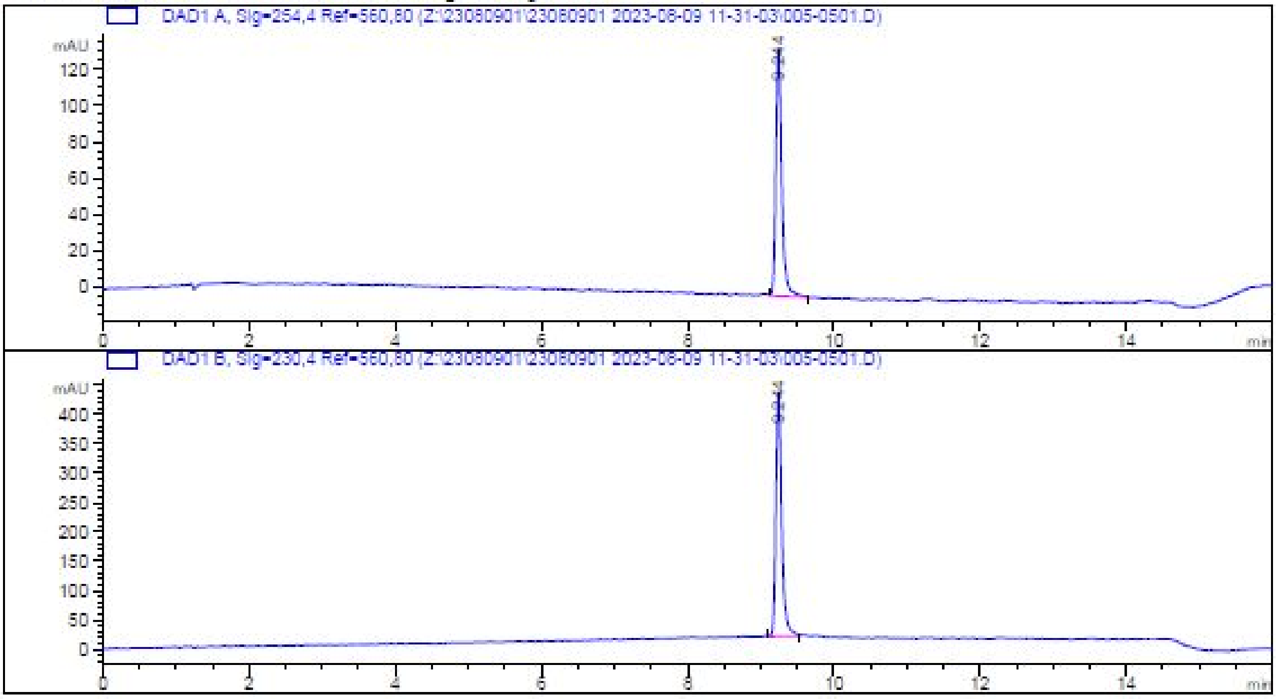


```
=====================================================================
                         Area Percent Report
=====================================================================

Sorted By             :      Signal
Multiplier:                  :      1.0000
Dilution:                    :      1.0000
Use Multiplier & Dilution Factor with ISTDs


Signal 1: DAD1 A, Sig=254,4 Ref=560,80

Peak RetTime Type  Width     Area      Height     Area
  #   [min]        [min]   [mAU*s]     [mAU]       %
----|-------|----|-------|----------|----------|--------|
   1   9.244 VB    0.0857  773.09656  137.21986 100.0000

Totals :                   773.09656  137.21986


Signal 2: DAD1 B, Sig=230,4 Ref=560,80

Peak RetTime Type  Width     Area      Height     Area
  #   [min]        [min]   [mAU*s]     [mAU]       %
----|-------|----|-------|----------|----------|--------|
   1   9.244 VV    0.0850 2321.99170  416.81140 100.0000
```

20h

```
=====================================================================
Acq. Operator   : Bene                            Seq. Line :    7
Acq. Instrument : Instrument 1                     Location : Vial 7
Injection Date  : 07.08.2023 14:13:19                   Inj :    1
                                                 Inj Volume : 5.000 µl
Acq. Method     : C:\CHEM32\1\DATA\23080701\23080701 2023-08-07 13-55-07\ZORBAX1.M
Last changed    : 28.09.2021 15:34:30 by Flo
Analysis Method : C:\PROGRAM FILES (X86)\CHEMSTATION\1\METHODS\DEF_LC.M
Additional Info : Peak(s) manually integrated
```

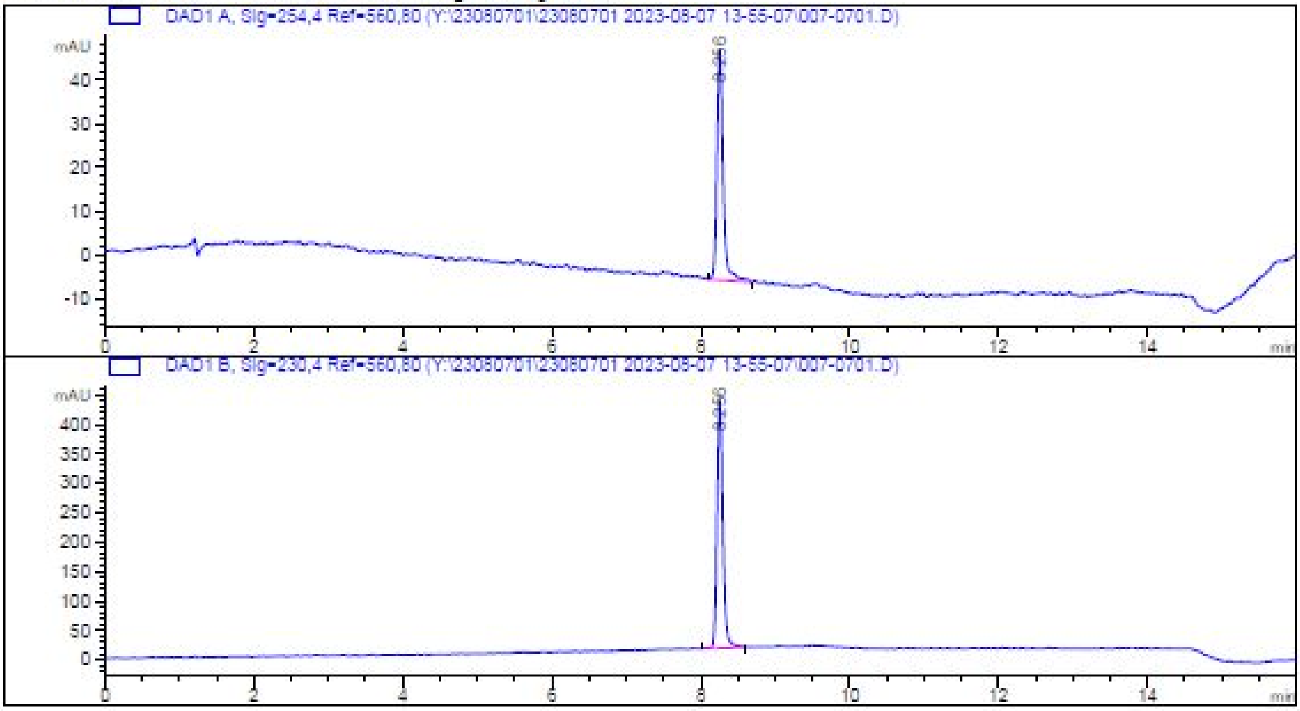


```
=====================================================================
                         Area Percent Report
=====================================================================

Sorted By             :      Signal
Multiplier:                   :      1.0000
Dilution:                     :      1.0000
Use Multiplier & Dilution Factor with ISTDs


Signal 1: DAD1 A, Sig=254,4 Ref=560,80

Peak RetTime Type  Width     Area      Height     Area
  #   [min]        [min]   [mAU*s]     [mAU]       %
----|-------|----|-------|----------|----------|--------|
   1   8.256 BB    0.0868  303.05643   52.95160 100.0000

Totals :                   303.05643   52.95160


Signal 2: DAD1 B, Sig=230,4 Ref=560,80

Peak RetTime Type  Width     Area      Height     Area
  #   [min]        [min]   [mAU*s]     [mAU]       %
----|-------|----|-------|----------|----------|--------|
   1   8.256 VV    0.0834 2303.88623  424.07605 100.0000
```

HRMS spectra for selected compounds

8i

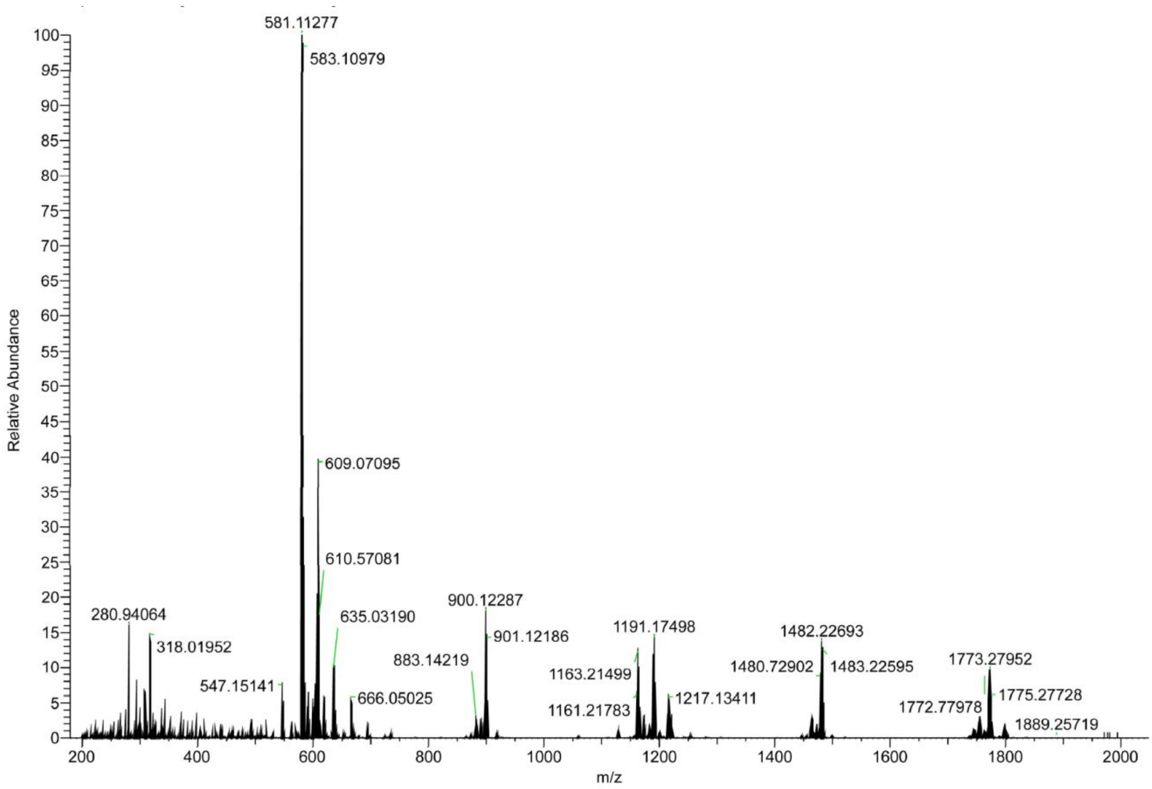

8j

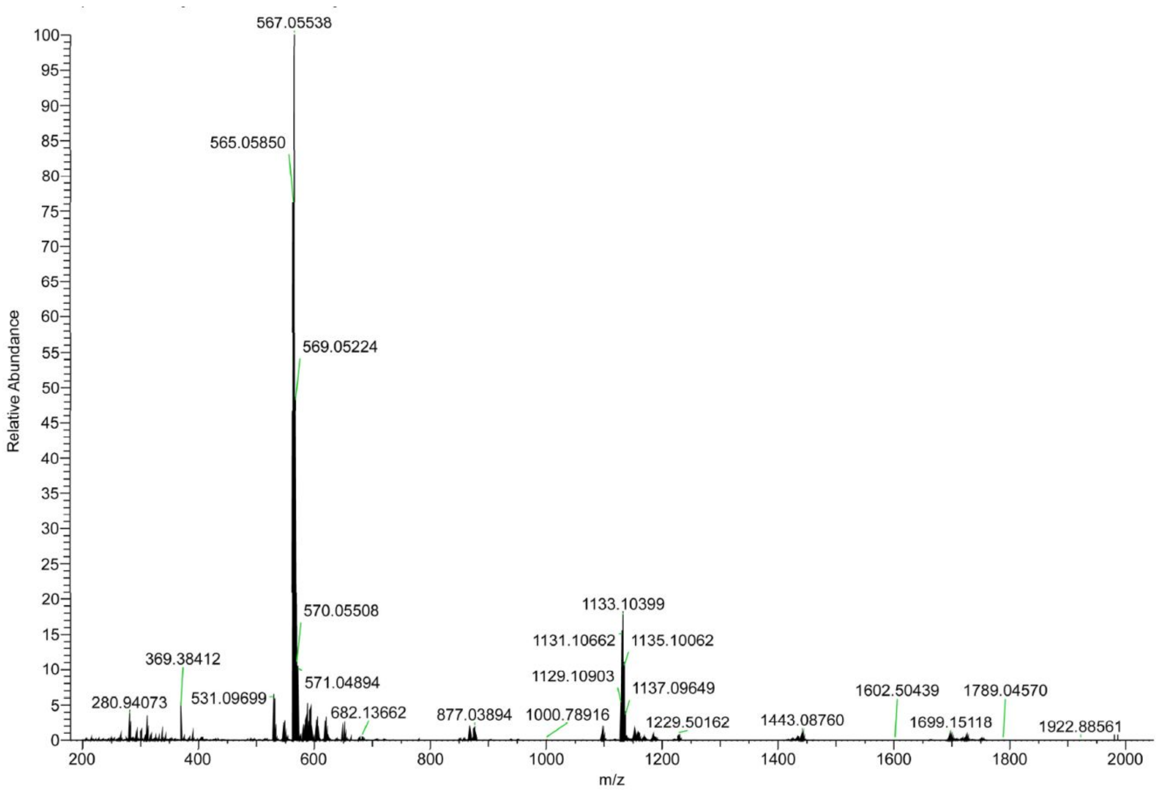

8k

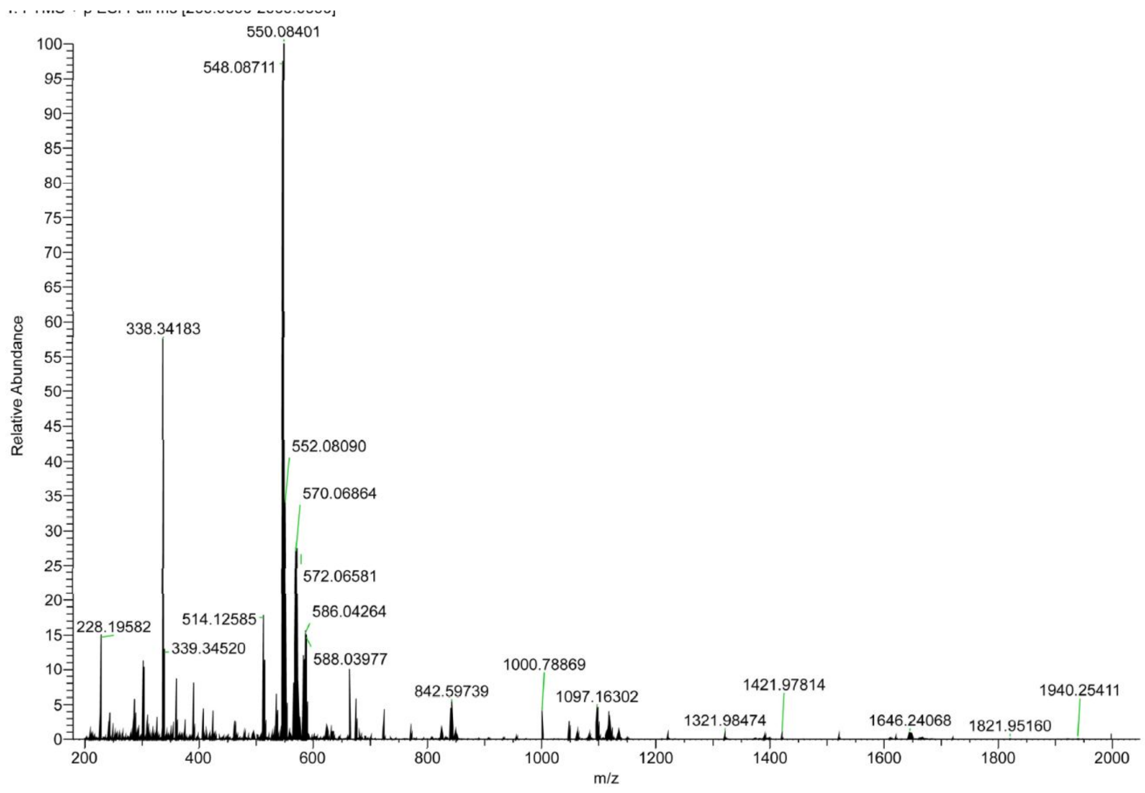
550.08401
548.08711
338.34183
552.08090
570.06864
572.06581
586.04264
514.12585
228.19582
339.34520
588.03977
1000.78869
842.59739
1097.16302
1421.97814
1321.98474
1646.24068
1821.95160
1940.25411
Relative Abundance
m/z

14b

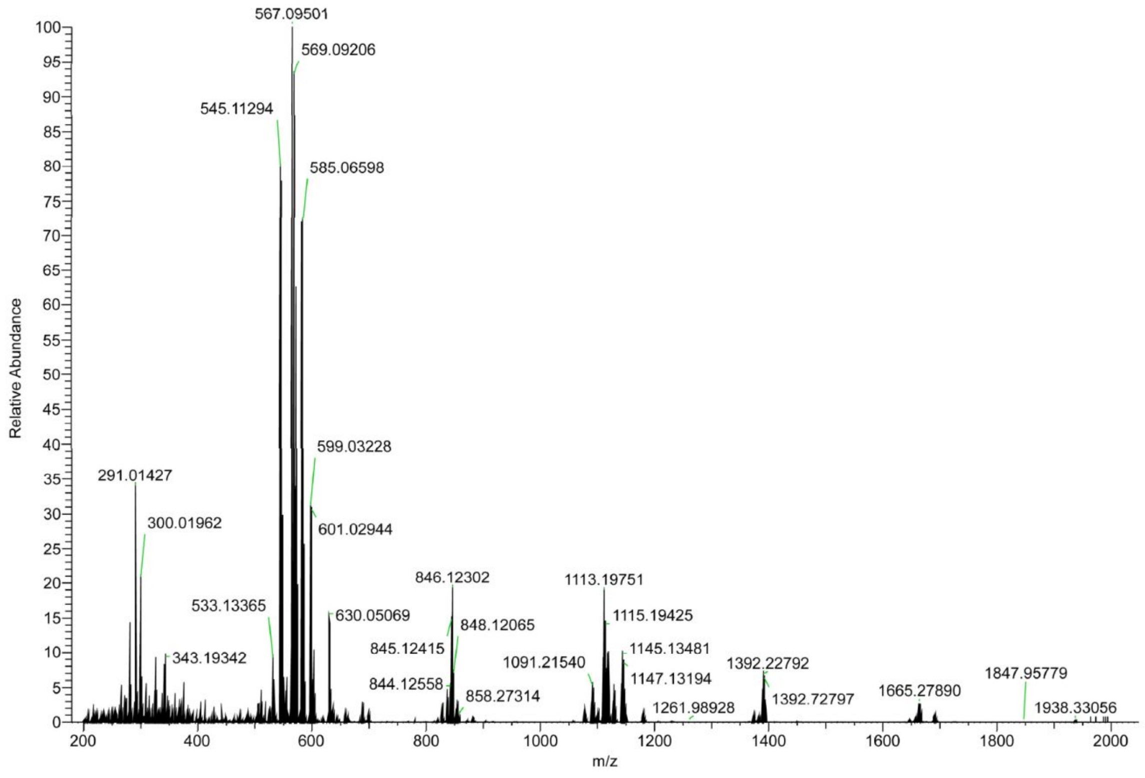
567.09501
569.09206
545.11294
585.06598
599.03228
291.01427
601.02944
300.01962
846.12302
1113.19751
533.13365
630.05069
1115.19425
848.12065
845.12415
1145.13481
343.19342
1091.21540
1392.22792
1847.95779
1147.13194
844.12558
858.27314
1665.27890
1392.72797
1261.98928
1938.33056
Relative Abundance
m/z

14c

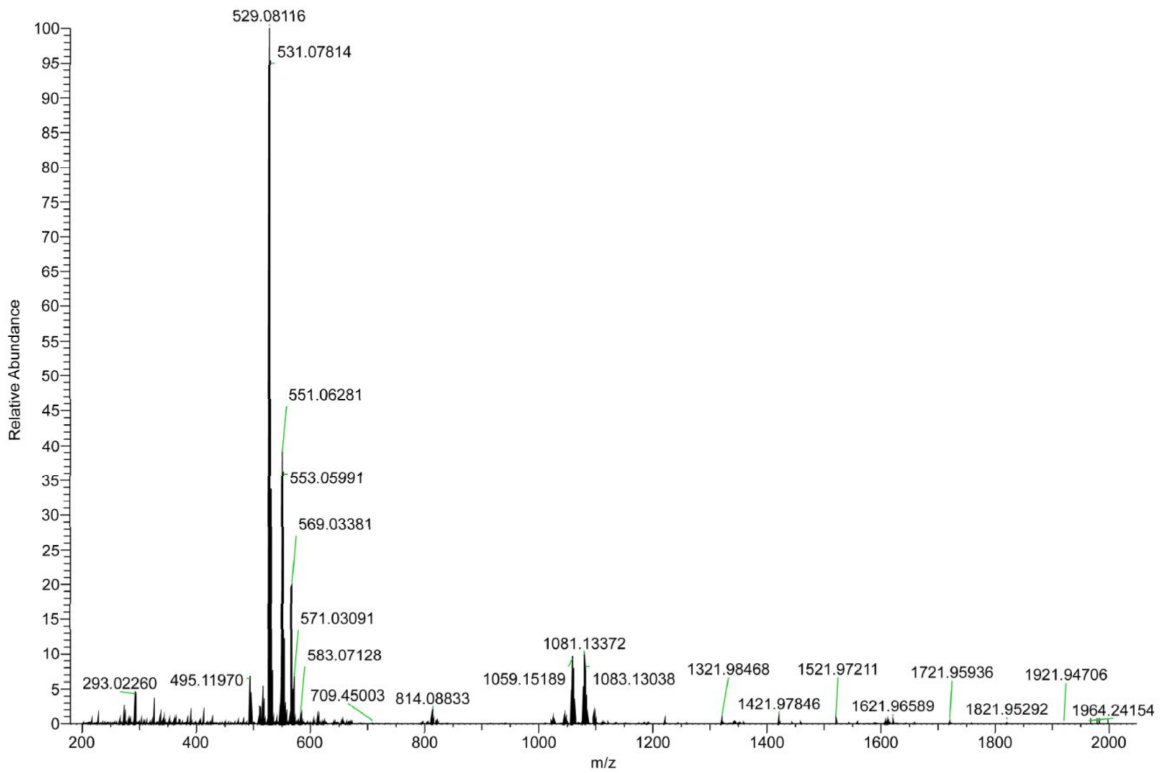
529.08116
531.07814
551.06281
553.05991
569.03381
571.03091
583.07128
293.02260
495.11970
709.45003
814.08833
1081.13372
1059.15189
1083.13038
1321.98468
1421.97846
1521.97211
1621.96589
1721.95936
1821.95292
1921.94706
1964.24154
Relative Abundance
m/z

14d

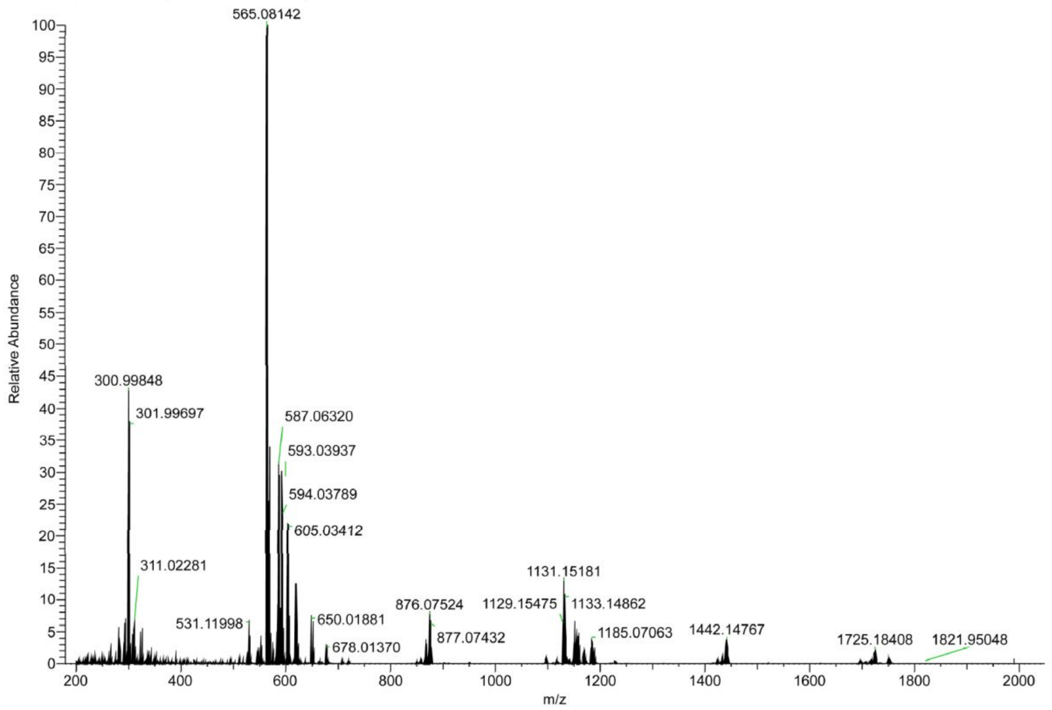
565.08142
300.99848
301.99697
311.02281
587.06320
593.03937
594.03789
605.03412
531.11998
650.01881
678.01370
876.07524
877.07432
1131.15181
1129.15475
1133.14862
1185.07063
1442.14767
1725.18408
1821.95048
Relative Abundance
m/z

14f

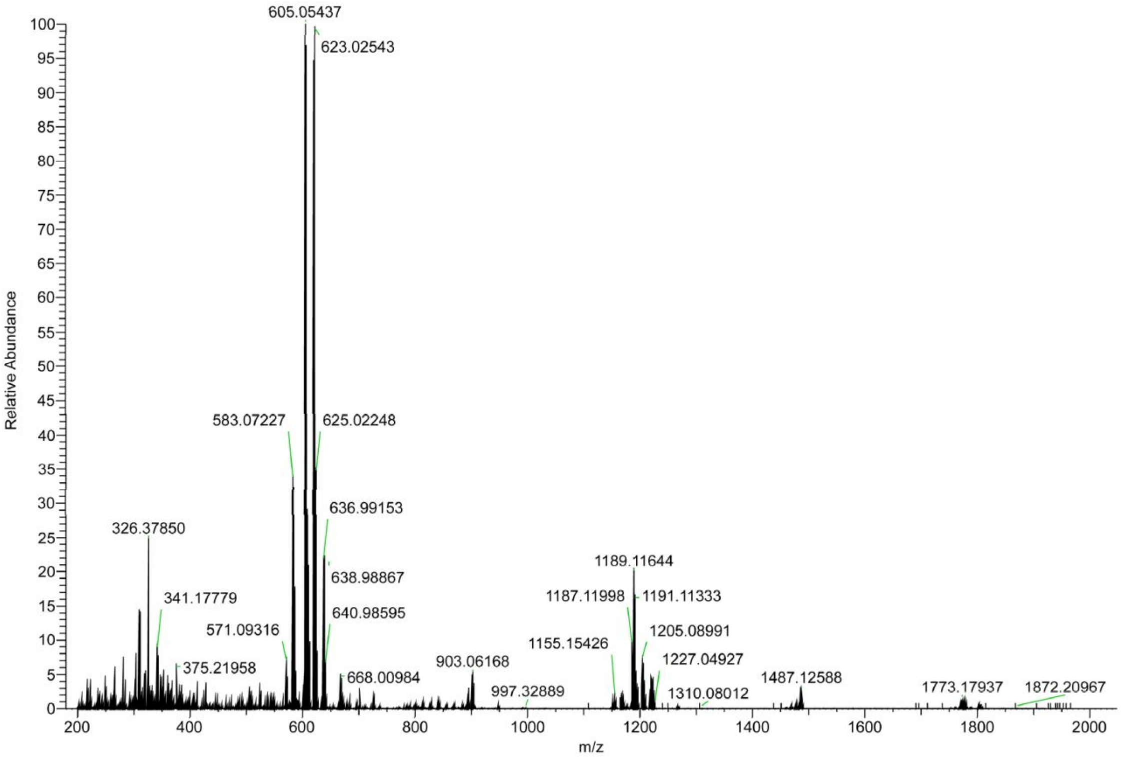
605.05437
623.02543
583.07227
625.02248
636.99153
638.98867
640.98595
326.37850
341.17779
571.09316
375.21958
668.00984
903.06168
997.32889
1189.11644
1187.11998
1191.11333
1205.08991
1155.15426
1227.04927
1310.08012
1487.12588
1773.17937
1872.20967
Relative Abundance
m/z

14h

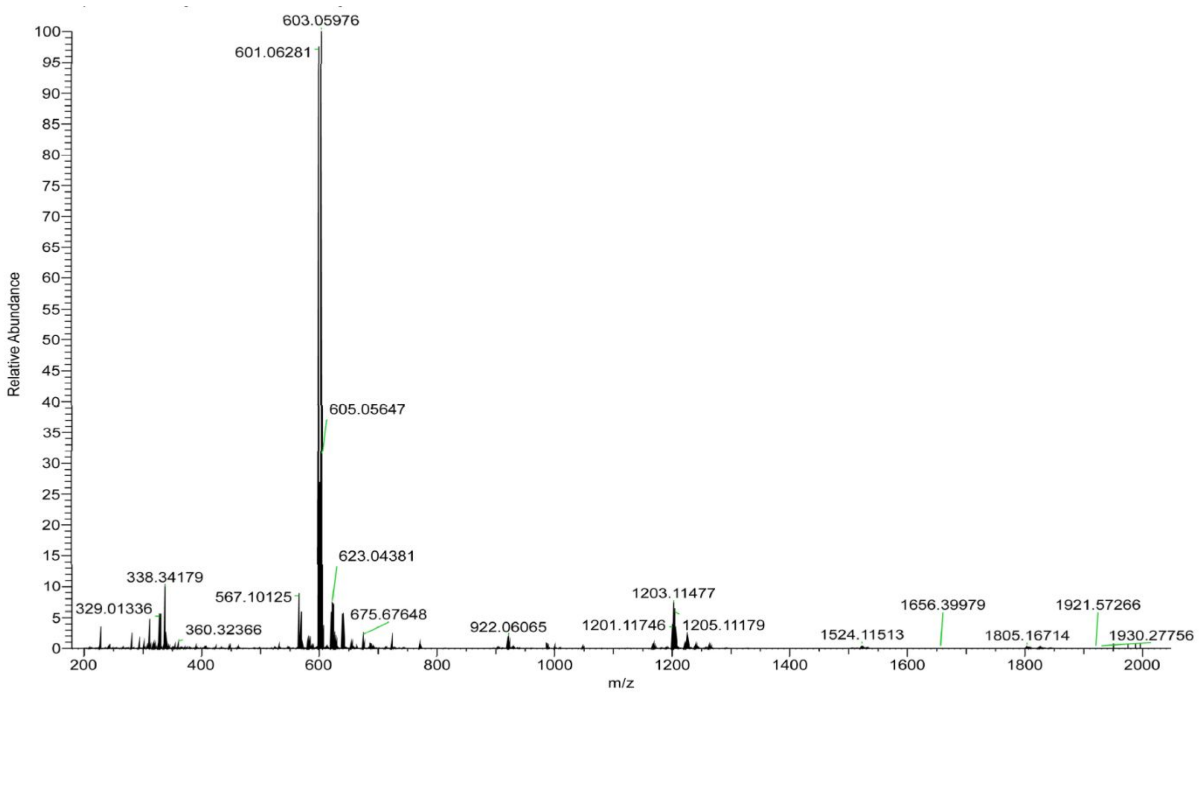
603.05976
601.06281
605.05647
623.04381
338.34179
329.01336
567.10125
360.32366
675.67648
922.06065
1203.11477
1201.11746
1205.11179
1524.11513
1656.39979
1805.16714
1921.57266
1930.27756
Relative Abundance
m/z

14m

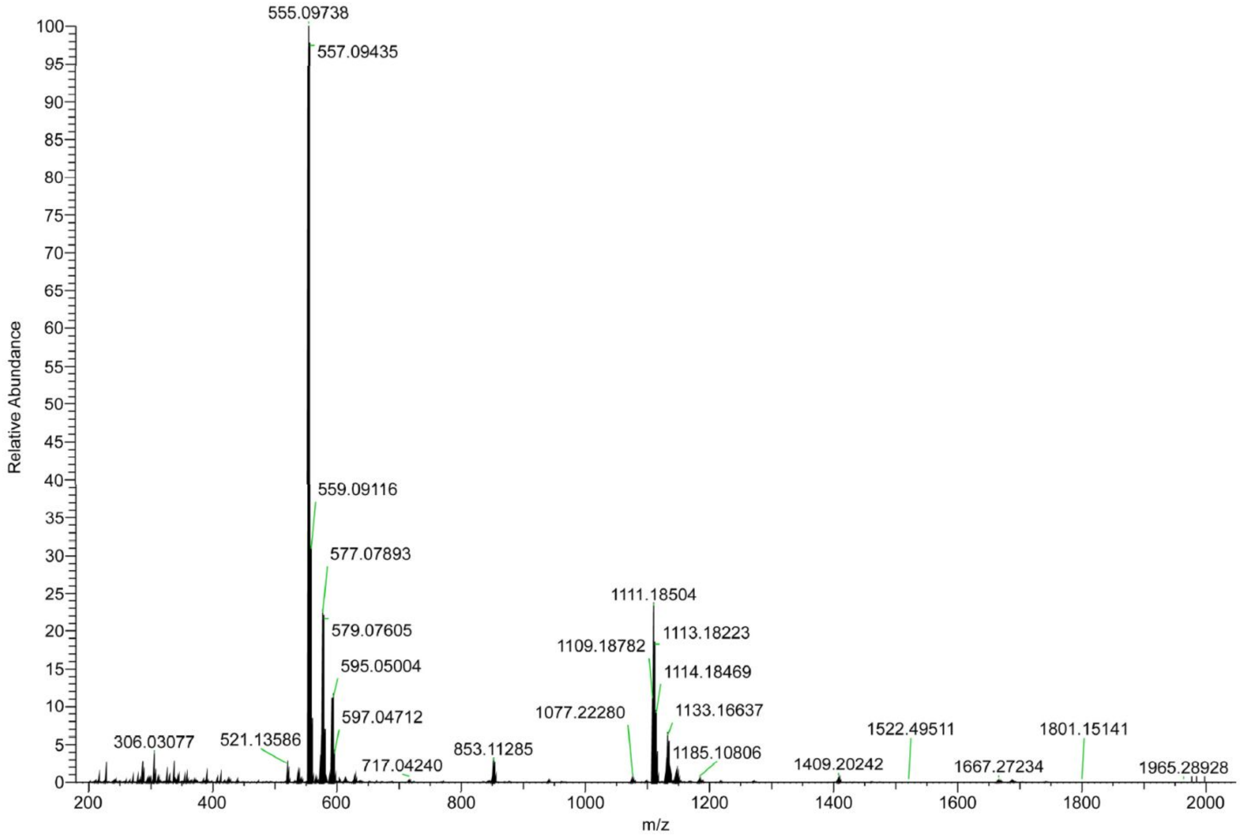
555.09738
557.09435
559.09116
577.07893
579.07605
595.05004
597.04712
306.03077
521.13586
717.04240
853.11285
1077.22280
1109.18782
1111.18504
1113.18223
1114.18469
1133.16637
1185.10806
1409.20242
1522.49511
1667.27234
1801.15141
1965.28928
Relative Abundance
m/z

14q

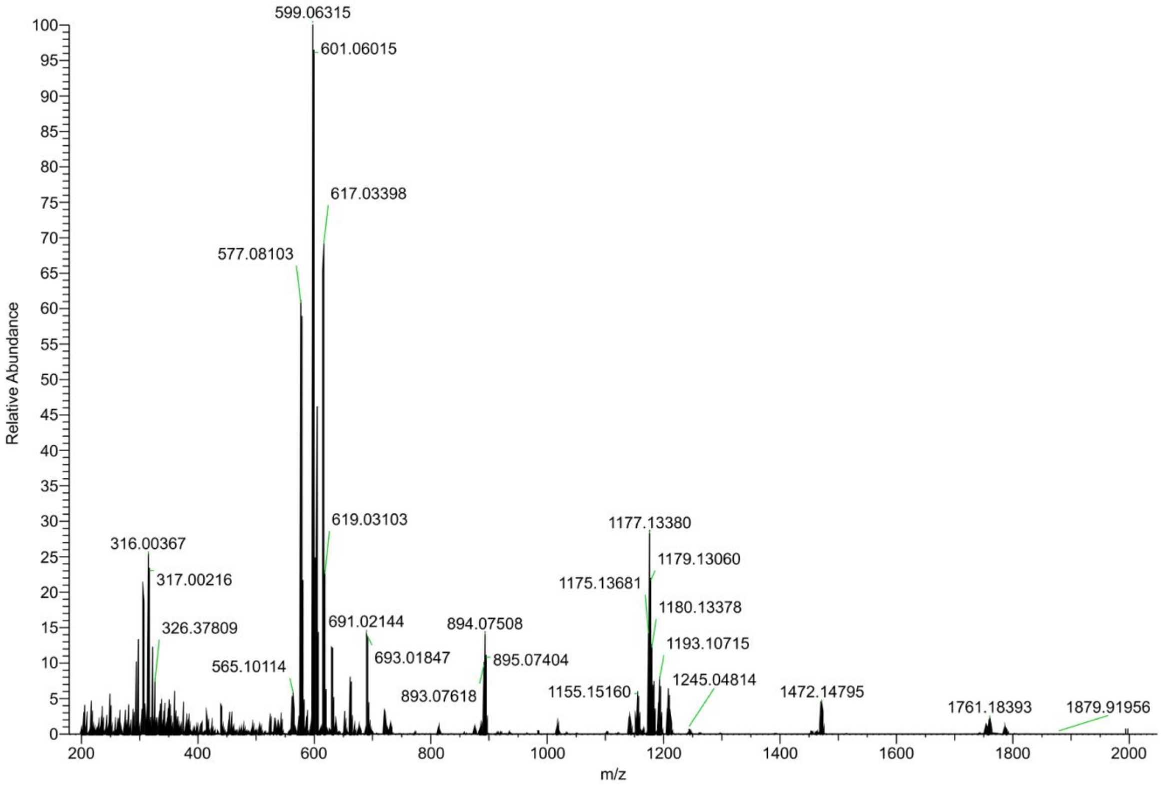
599.06315
601.06015
617.03398
577.08103
619.03103
316.00367
317.00216
326.37809
565.10114
691.02144
693.01847
894.07508
895.07404
893.07618
1177.13380
1179.13060
1175.13681
1180.13378
1193.10715
1155.15160
1245.04814
1472.14795
1761.18393
1879.91956
Relative Abundance
m/z

14r

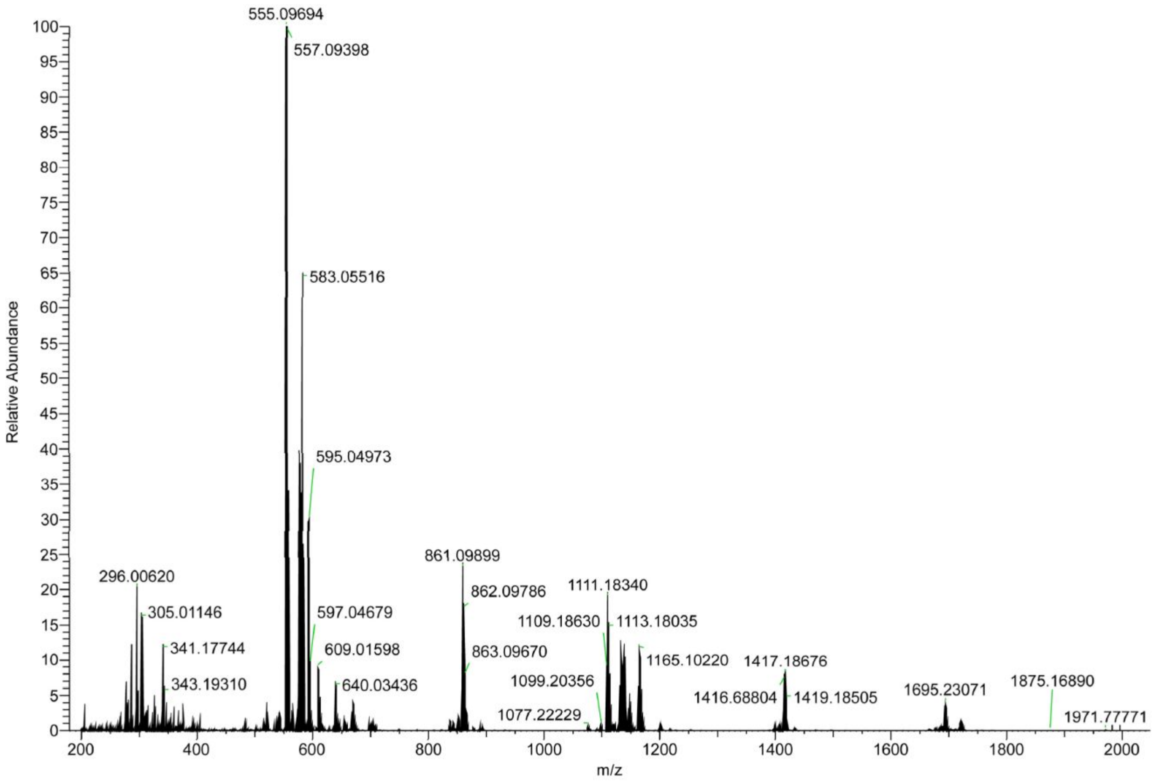

555.09694
557.09398
583.05516
595.04973
861.09899
862.09786
1111.18340
1109.18630
1113.18035
296.00620
305.01146
597.04679
609.01598
341.17744
343.19310
640.03436
863.09670
1165.10220
1417.18676
1099.20356
1416.68804
1419.18505
1695.23071
1875.16890
1077.22229
1971.77771
Relative Abundance
m/z

20a

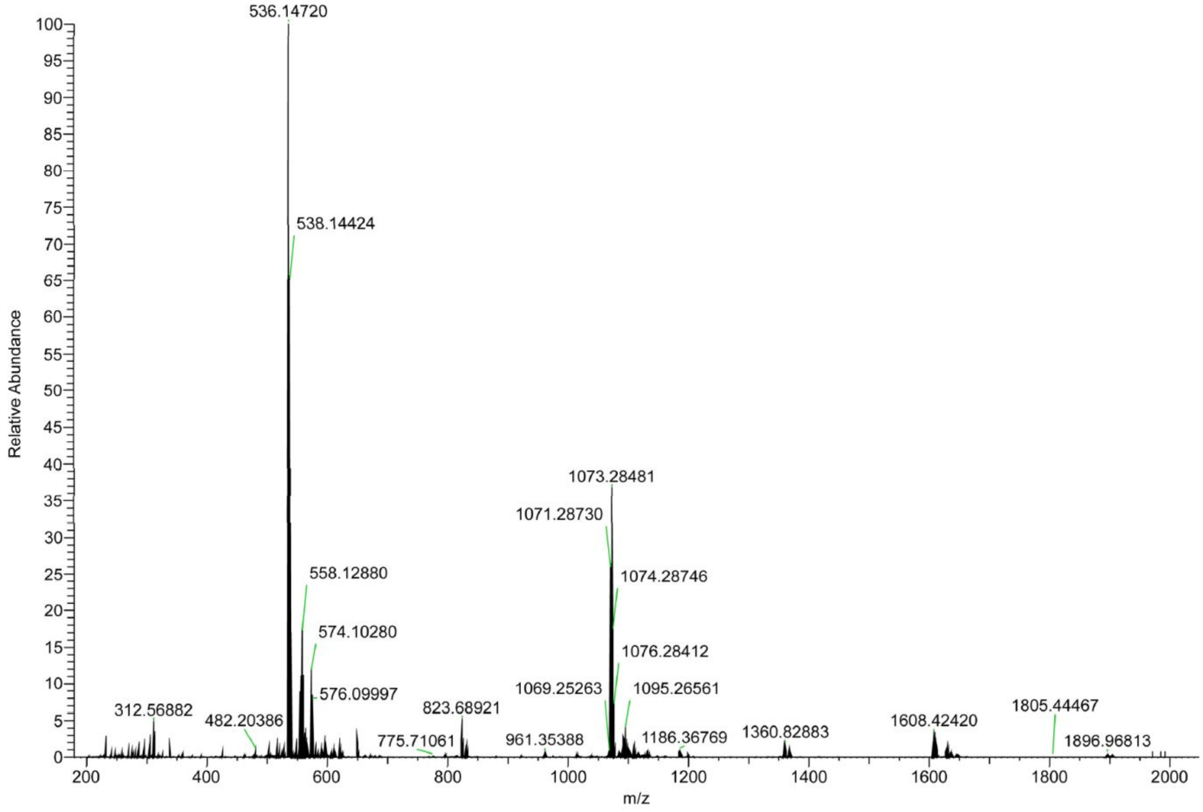

536.14720
538.14424
1073.28481
1071.28730
1074.28746
558.12880
574.10280
1076.28412
576.09997
1069.25263
1095.26561
312.56882
482.20386
823.68921
775.71061
961.35388
1186.36769
1360.82883
1608.42420
1805.44467
1896.96813
Relative Abundance
m/z

20b

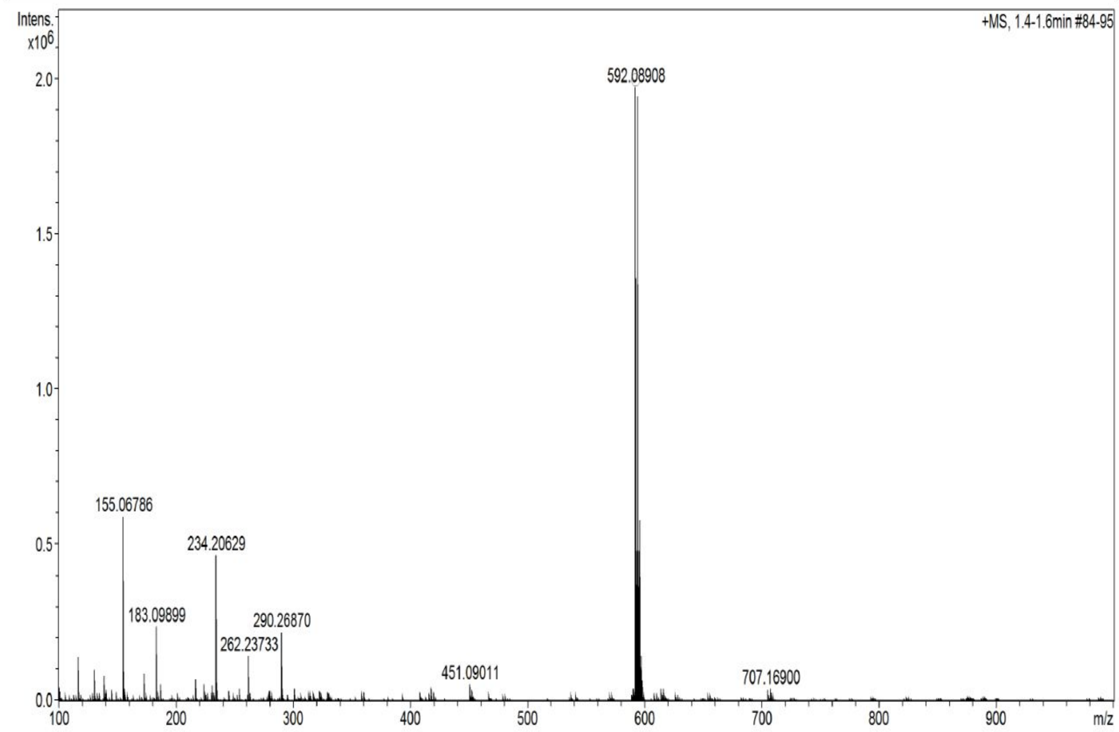

	Meas. m/z	#	Ion Formula	m/z	err [ppm]	mSigma	# mSigma	Score	rdb	e⁻ Conf	N-Rule
[M+Na]+	592.08908	1	C24H26Cl3N5NaO5	592.08917	0.16	30.4	1	100.00	12.5	even	ok

20c

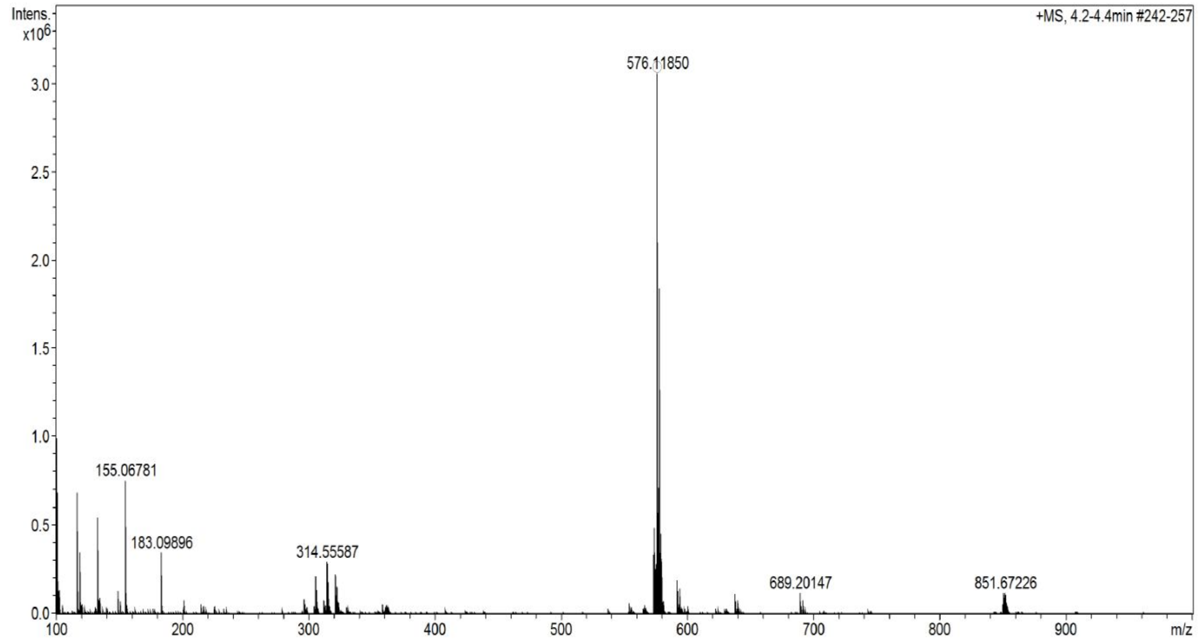

	Meas. m/z	#	Ion Formula	m/z	err [ppm]	mSigma	# mSigma	Score	rdb	e⁻ Conf	N-Rule
[M+Na]+	576.11850	1	C24H26Cl2FN5NaO5	576.11872	0.40	47.0	1	100.00	12.5	even	ok

20d

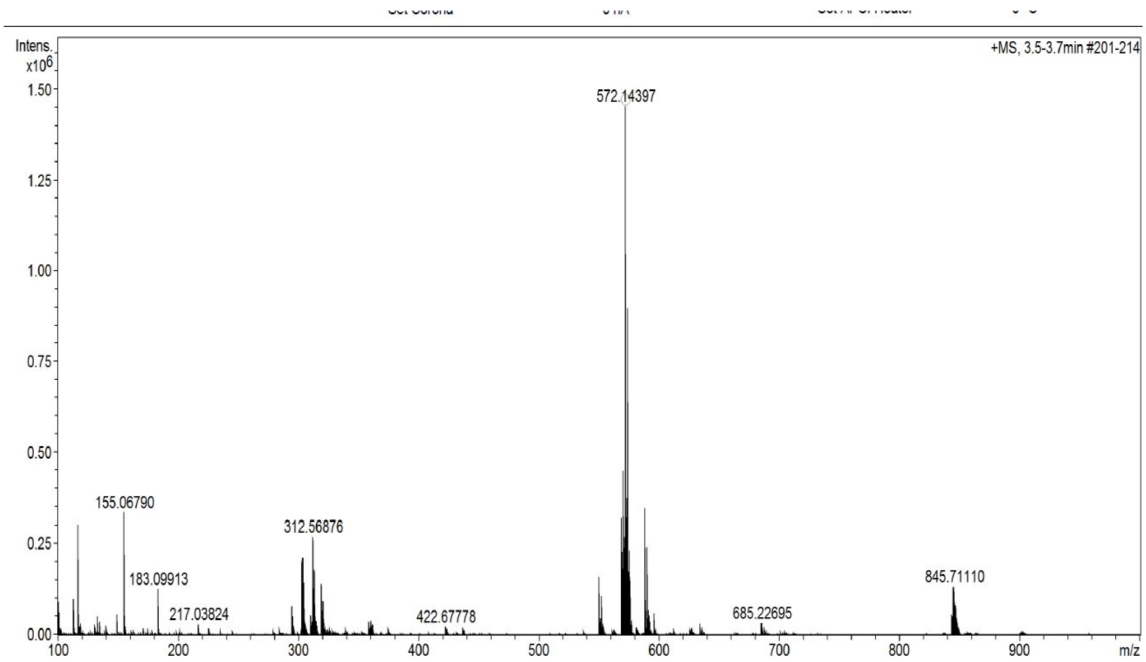

	Meas. m/z	#	Ion Formula	m/z	err [ppm]	mSigma	# mSigma	Score	rdb	e⁻ Conf	N-Rule
$[M+Na]^+$	572.14397	1	C25H29Cl2N5NaO5	572.14380	-0.31	39.3	1	100.00	12.5	even	ok

20g

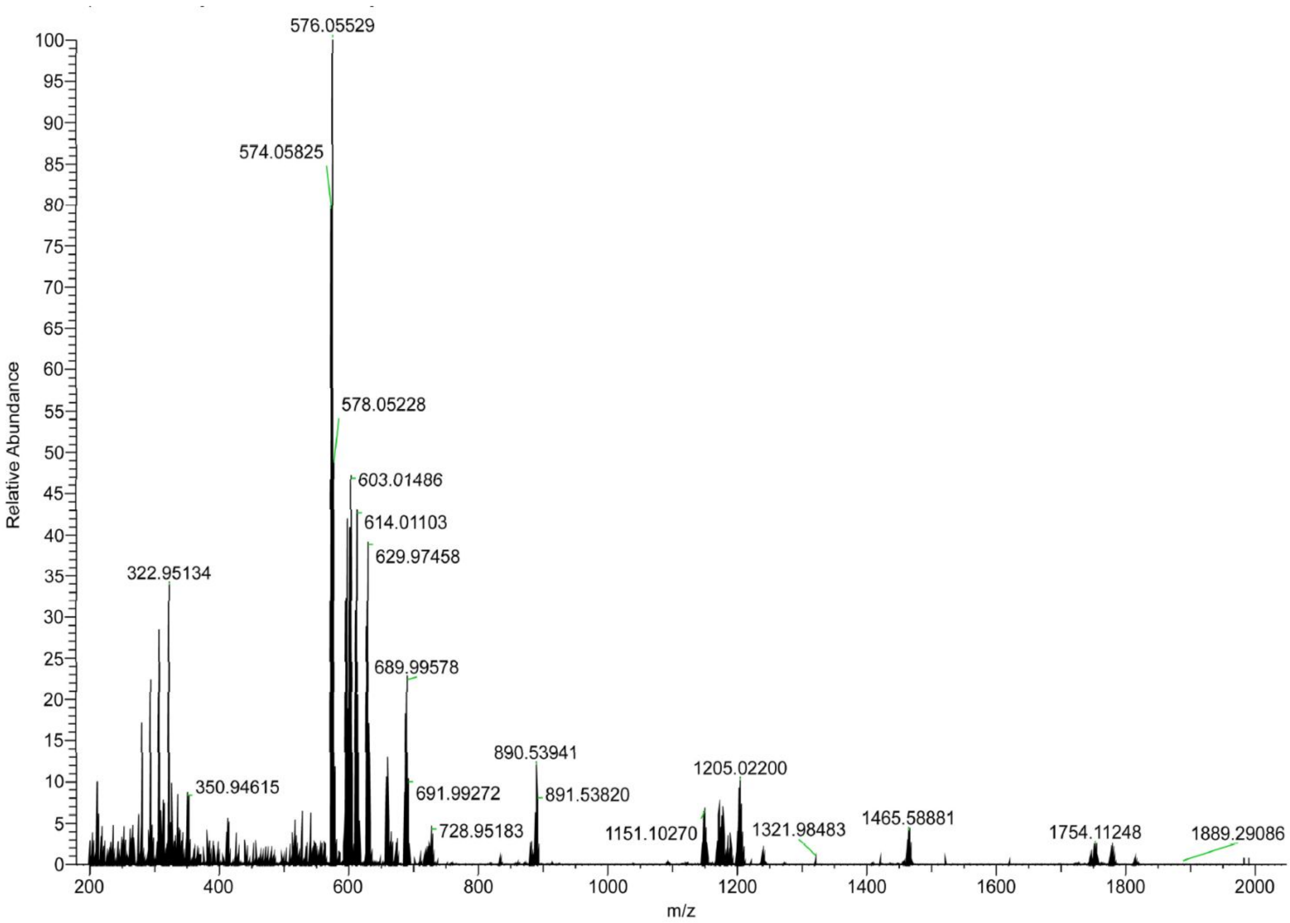

20h

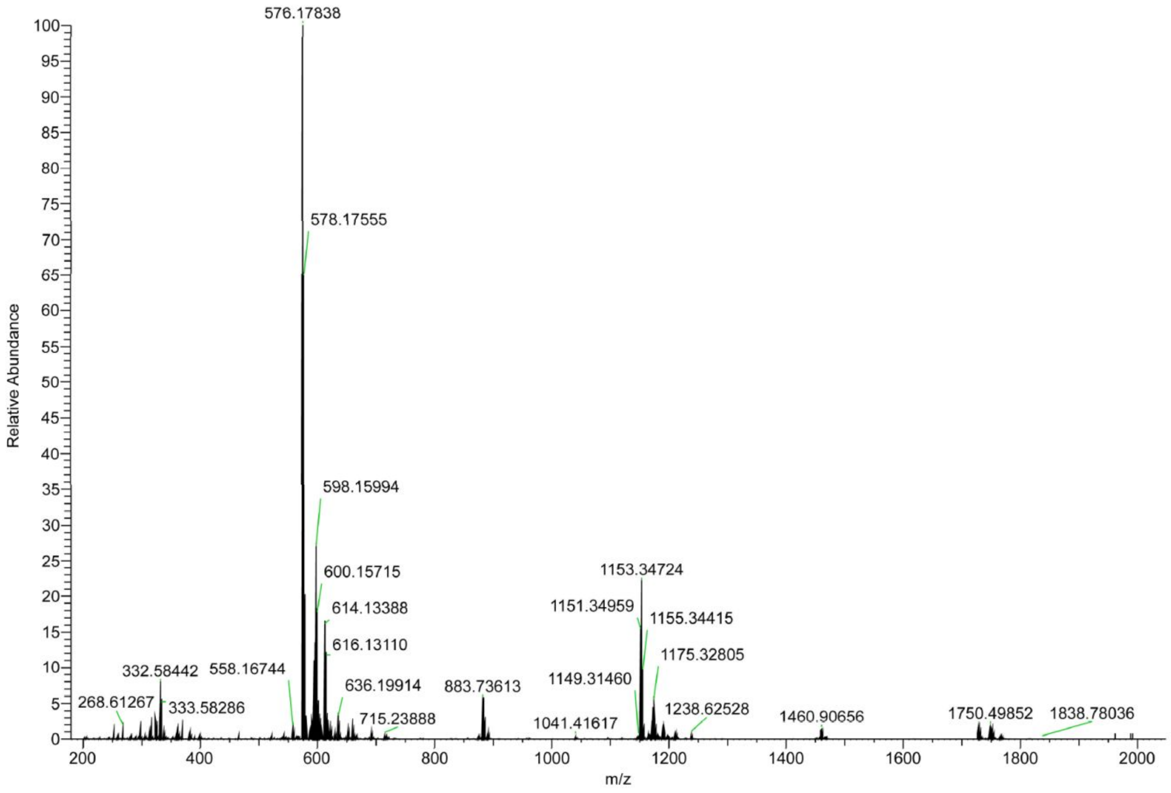

576.17838
578.17555
598.15994
600.15715
614.13388
616.13110
636.19914
715.23888
558.16744
332.58442
268.61267
333.58286
883.73613
1041.41617
1153.34724
1151.34959
1155.34415
1175.32805
1149.31460
1238.62528
1460.90656
1750.49852
1838.78036
Relative Abundance
m/z
